# The Malarial Host-Targeting Signal Is Conserved in the Irish Potato Famine Pathogen

**DOI:** 10.1371/journal.ppat.0020050

**Published:** 2006-05-26

**Authors:** Souvik Bhattacharjee, N. Luisa Hiller, Konstantinos Liolios, Joe Win, Thirumala-Devi Kanneganti, Carolyn Young, Sophien Kamoun, Kasturi Haldar

**Affiliations:** 1 Departments of Pathology and Microbiology and Immunology, Northwestern University, Chicago, Illinois, United States of America; 2 Department of Plant Pathology, The Ohio State University, Ohio Agricultural Research and Development Center, Wooster, Ohio, United States of America; University of North Carolina, United States of America

## Abstract

Animal and plant eukaryotic pathogens, such as the human malaria parasite Plasmodium falciparum and the potato late blight agent *Phytophthora infestans,* are widely divergent eukaryotic microbes. Yet they both produce secretory virulence and pathogenic proteins that alter host cell functions. In *P. falciparum,* export of parasite proteins to the host erythrocyte is mediated by leader sequences shown to contain a host-targeting (HT) motif centered on an RxLx (E, D, or Q) core: this motif appears to signify a major pathogenic export pathway with hundreds of putative effectors. Here we show that a secretory protein of *P. infestans,* which is perceived by plant disease resistance proteins and induces hypersensitive plant cell death, contains a leader sequence that is equivalent to the *Plasmodium* HT-leader in its ability to export fusion of green fluorescent protein (GFP) from the P. falciparum parasite to the host erythrocyte. This export is dependent on an RxLR sequence conserved in P. infestans leaders, as well as in leaders of all ten secretory oomycete proteins shown to function inside plant cells. The RxLR motif is also detected in hundreds of secretory proteins of *P. infestans, Phytophthora sojae,* and Phytophthora ramorum and has high value in predicting host-targeted leaders*.* A consensus motif further reveals E/D residues enriched within ~25 amino acids downstream of the RxLR, which are also needed for export. Together the data suggest that in these plant pathogenic oomycetes, a consensus HT motif may reside in an extended sequence of ~25–30 amino acids, rather than in a short linear sequence. Evidence is presented that although the consensus is much shorter in *P. falciparum,* information sufficient for vacuolar export is contained in a region of ~30 amino acids, which includes sequences flanking the HT core. Finally, positional conservation between *Phytophthora* RxLR and P. falciparum RxLx (E, D, Q) is consistent with the idea that the context of their presentation is constrained. These studies provide the first evidence to our knowledge that eukaryotic microbes share equivalent pathogenic HT signals and thus conserved mechanisms to access host cells across plant and animal kingdoms that may present unique targets for prophylaxis across divergent pathogens.

## Synopsis

Microbial interactions with host cells frequently involve utilization of pathogenic effectors that cause virulent infection and disease in the host. How these eukaryotic pathogenic effectors appear in the host cell is largely unknown. Recent studies have identified the first host-targeting (HT) signal for a eukaryotic pathogen in the human malaria parasite Plasmodium falciparum. Bhattacharjee, Haldar, and colleagues show that the *Plasmodium* HT-signal is conserved in the biotrophic oomycete Phytophthora infestans that caused the Irish potato famine. Like its malarial counterpart, the *Phytophthora* HT signal is present in major, known, virulence proteins, and predicts a pathogenic “host-targeted-secretome” of hundreds of putative effectors to colonize the host cell. Since *Plasmodium* and *Phytophthora* belong to distinct evolutionary groups, the study establishes for the first time that different eukaryotic microbes can share similar strategies in delivering toxic proteins to their hosts. This may present shared targets for controlling vastly different infections of both animals and plants. The present work has implications for agriculture and human health, since *Phytophthora* species devastate a wide range of food and commercial crops and *Plasmodium* species cause malaria, which kills more than one million children each year.

## Introduction

A wide range of microbial pathogens causes disease by secreting proteins into their plant and animal host cells [1–3]. Bacterial effectors are known to carry leader sequences that enable their transport through specialized machinery dedicated to the pathogenic process. These leaders and their associated secretion systems are shared by many bacterial species, suggesting that conserved mechanisms underlie virulence and pathogenesis across a wide range of prokaryotes [4]. In contrast, little is known about leaders used by eukaryotic pathogens to target virulence determinants to their host cells and whether leaders can be shared across diverse pathogens is completely unknown.

**Figure 1:**
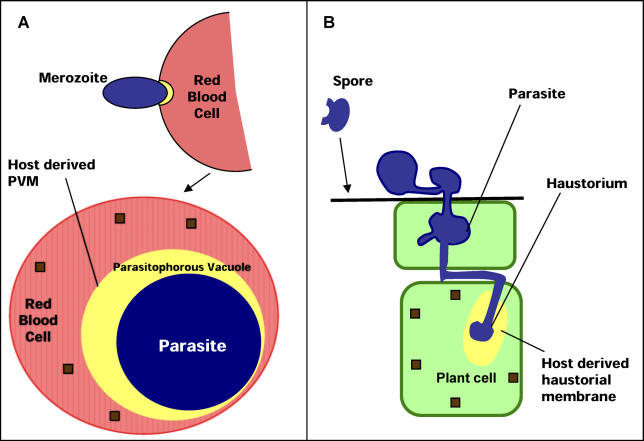
Schematic of Intracellular Infection of *Plasmodium* and *Phytophthora* Parasites (A) A human erythrocyte (pink) infected by P. falciparum (blue). Invasion by the extracellular merozoite stage leads to formation of a host-derived PVM within which the parasite resides. Proteins (brown squares) secreted by the parasite must cross the PVM to reach and mediate virulence and structural changes in the erythrocyte. (B) Plant cells (green) infected by P. infestans (blue). P. infestans parasite colonizes the host intracellular spaces and forms haustoria (yellow). The host-derived haustorial membrane must be crossed by pathogenic effectors (brown squares) released into cells to mediate virulence and plant hypersensitive responses.

Recent studies show that the human malaria Plasmodium falciparum and other plasmodial species encode a host-targeting (HT) leader that can be used to define a host-targeted-secretome (HT-secretome) involved in blood stage infection [5,6]. P. falciparum is an apicomplexan parasite, which causes devastating disease, with a death toll approaching two million children a year [7]. During its blood stages of infection, the parasite invades mature human erythrocytes and develops within a parasitophorous vacuolar membrane (PVM) derived from an invagination of the erythrocyte membrane [8] (Figure 1A). Malarial proteins synthesized by the parasite and exported to the human erythrocyte induce virulence, antigenic and structural changes in the cytosol and membrane of the host cell, leading to many disease pathologies including death [7].

*Plasmodium* is a eukaryote, and, thus, proteins exported to the host cell must cross the parasite plasma membrane as well as the PVM. Proteins with an ER-type signal sequence (SS) are recruited into the parasite secretory pathway, the SS is cleaved, and proteins are released at the plasma membrane. Subsequent export across the surrounding PVM requires an HT-signal [5,6] that is present downstream of the secretory SS and revealed upon cleavage of the SS [9]. The HT-signal is detected on ~400 P. falciparum proteins that are predicted to extensively remodel the erythrocyte during infection. Further, it is conserved across plasmodial species [5,6]. This novel signal, as well as its cargo, may provide targets for new drugs and vaccines against malaria.

Oomycetes are deep-branching eukaryotes that include the biotrophic oomycete Hyaloperonospora parasitica as well as the notorious plant killer *Phytophthora,* which causes destructive diseases such as potato late blight and sudden oak death [10]. These pathogens cause arguably the most destructive diseases of dicotyledonous plants, and they remain difficult to manage. They encode for highly polymorphic proteins containing an ER-type eukaryotic secretory SS, suggesting that these proteins may be effectors released from the microbe into the host to induce disease (virulence function) and/or to elicit host defenses such as hypersensitive cell death (avirulence function) [11,12]. Importantly, allelic variations in these genes result in rapid adaptation in host response genes and host cell death during infection [13–15].

Further, these gene products are specifically recognized by the products of resistance genes upon transduction into the host [13–15]. Thus, genetic, evolutionary, and functional data provide a strong basis to infer that these gene products are pathogenic effectors delivered to the host [11,12,16]. All of the ten known oomycete avirulence (Avr) proteins, including AVR3a of Phytophthora infestans as well as ATR1 and ATR13 of *H. parasitica,* are perceived inside the host cytoplasm by cytosolic plant disease resistance proteins, resulting in hypersensitive cell death [11–16]. Oomycetes establish intimate association with host plant cells through structures such as appresoria, infection vesicles, and haustoria (Figure 1B) [11,15–17]. These structures penetrate plant cells, although they remain enveloped by a lipidic membrane derived from the plant plasma membrane (Figure 1B).

The ER-type SS of oomycete Avr proteins presumably directs the proteins beyond the pathogen plasma membrane. Recent studies identified a highly conserved N-terminal sequence motif RxLR, positioned within 60 amino acids downstream of the ER-type SS in multiple oomycete effectors, including AVR3a of P. infestans [14,15]. Here we show that *Phytophthora* leaders containing the SS, followed by the RxLR motif and an E/D rich domain further downstream, can efficiently substitute for *Plasmodium* HT leaders in driving protein export out of the malarial vacuole and into the host erythrocyte. This equivalence in function when combined with equivalence in both position and specific sequence requirements in the HT motif suggests that deep branching eukaryotes belonging to distinct groups such as heterokonts *(Phytophthora)* and that alveolates *(Plasmodium)* share conserved pathogenic secretion strategies to access host cells across plant and animal kingdoms [18].

## Results

### Comparative Analyses of Leader Sequences in *Plasmodium* and *Phytophthora*

**Figure 2:**
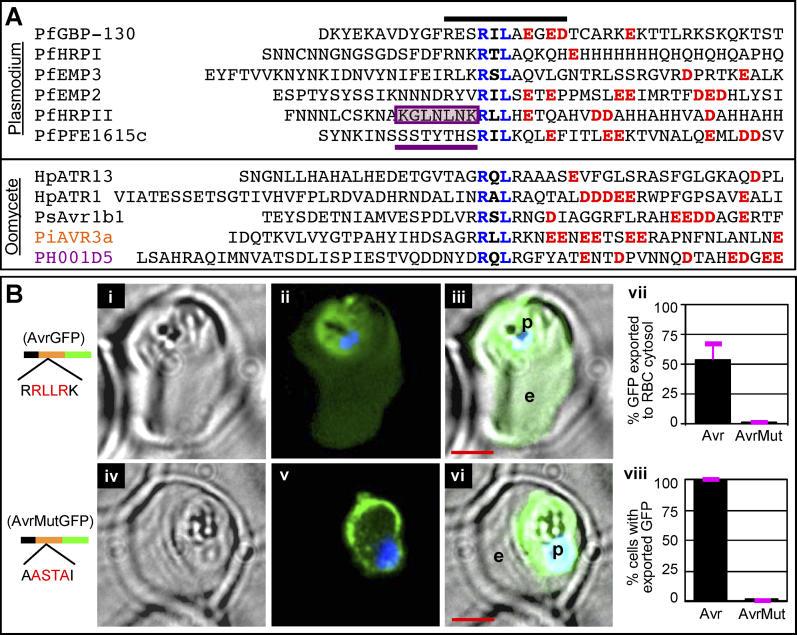
Conserved, Plant Pathogenic Oomycete Motif Functions as a Host-Targeting Signal in P. falciparum–Infected Erythrocytes (A) Sequence alignment of six effectors from P. falciparum HT*-*secretome (upper panel) and five Avr proteins from the oomycete pathogens *Phytophthora* and *Hyaloperonospora* (lower panel). Each sequence contains an 11 amino acid region (black bar) centered on RxL residues (blue). Negatively charged residues (E or D) downstream of the RxL are colored red. Seven residues upstream of the RxL are underlined purple and boxed in the case of PfHRPII. (B) Live cells expressing secretory GFP chimeras of AVR3a (residues 21 to 69) with no change (i–iii), or where RRLLRK was replaced by AASTAI (iv–vi), where (ii) and (v) indicate fluorescence images, (i) and (iv) corresponding brightfield images, and (iii) and (vi) the respective merges. Constructs contain SS (black) followed by indicated sequences of AVR3a (orange) and GFP (green). For quantitative analyses, two hundred fluorescent images were analyzed as described in Materials and Methods. Fraction of GFP exported to the erythrocyte cytosol is indicated in (vii). As expected, all parasitized cells express and export the transgene (viii). Standard deviations are shown in pink. In all cases: p, parasite; e, erythrocyte; nucleus is Hoechst-stained (blue); scale bar represents 2 μm.

Our prior studies have demonstrated that several hundred secreted plasmodial proteins carry the HT signal immediately downstream of the SS within a vacuolar translocation sequence necessary and sufficient to mediate export of GFP to the host erythrocyte. Shown in Figure 2A are multiple sequence alignments of vacuolar translocation sequences (~40 amino acids in length) of six soluble P. falciparum proteins known to be exported to the erythrocyte. Each contains an HT motif that is an 11 amino acid sequence (black bar) centered on RxL residues (blue). The HT motif allows multiple residues at almost all positions. Nonetheless, it can be recognized by its confined core where the most common residues are RxLx (E, D, or Q) [5]. Similarly, five ~40 amino acid sequences downstream of the ER-type SS of four Avr proteins and one unknown protein from the oomycete pathogens *Phytophthora* and *Hyaloperonospora* contain a conserved sequence motif, just downstream of predicted SS, centered on RxLR residues (blue) and frequently followed by two consecutive negative residues (E or D) within an ~35 amino acid region (Figure 2A, lower panel) [15].

The oomycete RxLR is similar in sequence and position to the *Plasmodium* HT-motif, suggesting a related function. However, predictive algorithms for the plasmodial motif [5,6,19] do not recognize the *Phytophthora* RxLR. We therefore tested for functional conservation and to do this we expressed a green fluorescent protein (GFP) chimera of a 50 amino acid sequence from AVR3a of P. infestans containing RRLLRKNEENEETSEERA fused downstream of an ER-type SS (see Materials and Methods) in *P. falciparum.* The AVR3a sequence mediated the export of GFP to the host erythrocyte (Figure 2Bi–2Biii). Green fluorescence was also detected in the parasite, presumably due to continuous expression of this protein by the constitutive *cam* promoter. Nonetheless, an average of 50% of the GFP signal was found in the host erythrocyte (Figure 2Bvii–2Bviii). This was comparable to 40% export of GFP chimeras of P. falciparum proteins to the erythrocyte, as previously reported [9]. Replacement of RRLLRK by a different sequence of equal size and similar polarity AASTAI quantitatively blocked GFP export to the red cell (Figure 2Biv–2Bviii). Instead, the GFP transgene concentrated in the periphery of the parasite consistent with its accumulation in the parasitophorous vacuole. These data indicated that export to the erythrocyte was dependent on sequences containing the RxLR motif. They established that the oomycete leader can replace a P. falciparum leader in vacuolar translocation to the host cell and that the RxLR is functionally equivalent to the plasmodial HT-motif.

### Predictive Value of the RxLR Motif in a P. infestans HT-Secretome

**Figure 3:**
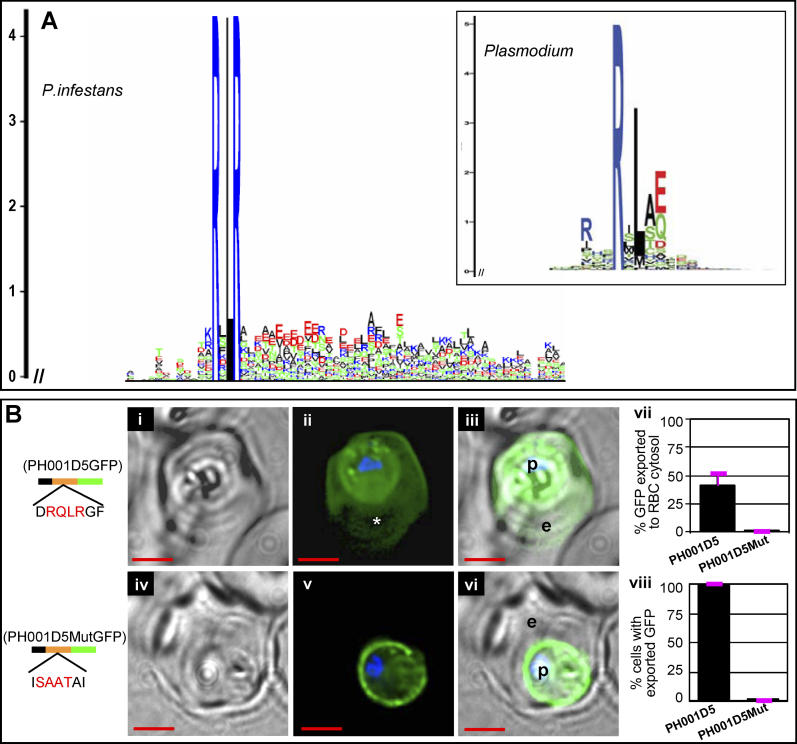
HT Motif Has High Predictive Value for *Phytophthora* Effectors (A) Sequence logos derived from 59 predicted P. infestans secretory proteins and the P. falciparum HT-secretome (boxed inset). Amino acids are represented by one-letter abbreviations and color-coded as follows: blue, basic; red, acidic; black, hydrophobic; and green, polar. Height of amino acids indicates their frequency at that position. (B) Live cells expressing secretory GFP chimeras of PH001D5 (residues 19 to 88) with no change (i–iii) or where DRQLRGF was replaced with ISAATAI (iv–vi), where (ii) and (v) indicate fluorescence images, (i and iv) corresponding brightfield images, and (iii and vi) show respective merges. The asterisk (*) in panel (ii) indicates intraerythrocytic loop structure that excluded GFP. Constructs contain SS (black), followed by indicated sequences of PH001D5 (orange) and GFP (green). For quantitative analyses, 220 fluorescent images were analyzed as described in Materials and Methods. Fraction of GFP exported to the erythrocyte cytosol is indicated in (vii) in a culture where all parasites are transformed (as expected) and export the transgene (viii). Standard deviations are shown in pink. Export of green fluorescence to erythrocyte is quantitatively blocked on replacement of the P. infestans motif (as indicated in the bar chart in vii [standard deviation show in pink]). p, parasite; e, erythrocyte; nucleus is Hoechst-stained (blue), scale bar is 2 μm.

We have previously shown that the plasmodial HT motif has high predictive value in identifying unknown proteins and defining an HT-secretome with hundreds of putative effectors [5]. In the present study, bioinformatic analyses indicated that RxLR occurred in P. infestans proteins containing an ER-type SS*.* Figure 3A is a sequence logo graphical representation created from 59 sequences that correspond to the subset of P. infestans–candidate effectors that contain at least 100 residues after the predicted SS cleavage site, highlighting the conservation of the RxLR motif. The height of each letter representing the amino acids indicates their relative frequency at that position, and the height of the stack of letters at any position represents the information content (measured in bits) of the sequence at that position [20].

To test the predictive value of this P. infestans motif, we selected a hypothetical protein corresponding to cDNA PH001D5 from the P. infestans–candidate effectors (Table S1). PH001D5 is known to be expressed in planta during infection stages (T. D. Kanneganti and S. Kamoun, unpublished data), but has no homologues in the NCBI non-redundant protein sequence database (see Materials and Methods). A GFP chimera containing the first 55 residues from PH001D5 fused downstream of an ER-type SS was transfected into P. falciparum (Figure 3Bi–3Biii). This construct exported an average of 40% GFP from P. falciparum to its host erythrocyte (Figure 3Bi–3Biii, Figure 3Bvii–3Bviii). Despite a slight reduction in the efficiency of export compared with the Avr3a transgene, these levels of export seen in stably transfected cells are nonetheless comparable to export of 40% of GFP chimeras of P. falciparum proteins to the erythrocyte as previously reported in the literature [9]. Moreover, when DRQLRGF in the motif was replaced by ISAATAI, transport to the erythrocyte was quantitatively blocked (Figure 3Biv–3Bviii). Rather, GFP concentrated in the periphery of the parasite consistent with its accumulation in the vacuole. These data demonstrated that export to the erythrocyte was dependent on sequences containing RQLR and that the oomycete RxLR motif is likely to have strong predictive value. Further, in conjunction with the results of Figure 2B, they suggested that P. infestans leaders are equivalent to P. falciparum leaders in translocating proteins to the host cell, and both sets of leaders clearly share the RxL.

### Contribution of E, D Downstream of RxLR in Phytophthora Sequences

Prior studies have indicated the prevalence of E/D/Q at the fifth position of the P. falciparum HT core [5,6] and validated it for one protein (called GBP 130, [6]). Nonetheless, our results from Figures 2 and 3 show that P. infestans sequences lacking conservation in the fifth position also quantitatively export the GFP reporter to the erythrocyte. This shows that while E/D/Q at position five of the core is important for GBP130, it is not required in all proteins. A comparison of the P. falciparum and P. infestans logos highlights the shared core of RxL between plasmodial and oomycete effectors (Figure 3A). It also suggests enrichment of E/D residues downstream of RxLR, as manifested by the height of red amino acids in this region.

**Figure 4:**
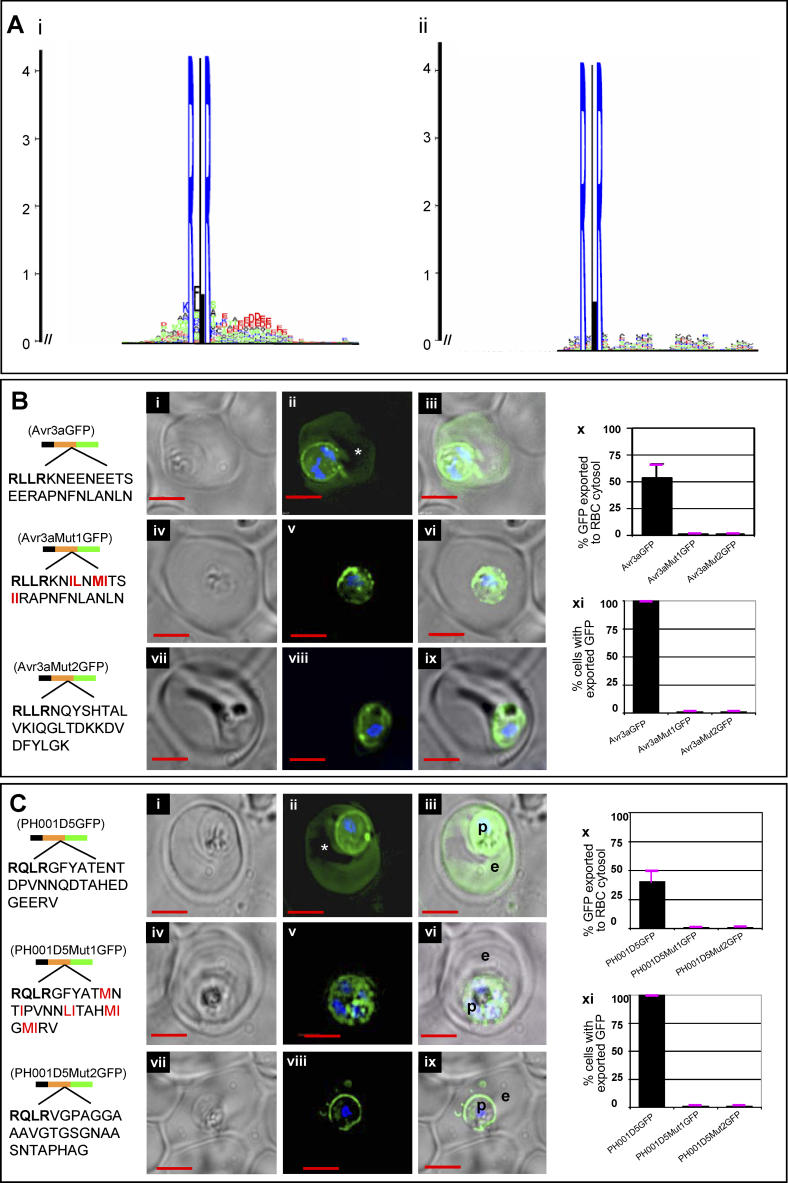
Comparative Analyses of Secretory and Cytosolic RxLR–Containing Sequences in *Phytophthora:* In Silico and Functional Evidence for the Requirement of Downstream E, D Residue Sequences in HT Consensus (Ai) Secretory: logos generated from sequences containing RxLR in the first 100 residues after the SS cleavage site for predicted secretory proteins (~10% of the SS set). (Aii) Cytosolic: logos from sequences containing RxLR in the first 100 residues of predicted cytosolic proteins (~5% of the cytosolic set). (B) Live cells expressing secretory GFP chimeras of Avr3a (residues 21–69) with no change (i–iii), replacement of D/E residues downstream of RxLR with hydrophobic residues (iv–vi), replacement of downstream sequence KNEENEETSEERAPNFNLANLN by NQYSHTALVKIQGLTDKKDYLGK found downstream of RxLR from a non-secretory *Phytophthora* protein AAY43422.1 (vii–ix). (C) Live cells expressing secretory GFP chimeras of PH001D5 (residues 19–88) with no change (i–iii), replacement of D/E residues downstream of RxLR with hydrophobic residues (iv–vi), replacement of downstream sequence GFYATENTDPVNNQDTAHEDGEERV by VGPAGGAAAVGTGSGNAASNTAPHAG found downstream of RxLR from a non-secretory *Phytophthora* protein 83742 (vii–ix). (B and C) Panels (ii, v, and viii) indicate fluorescence micrographs; (i, iv, and vii), brightfield images; (iii, vi, and ix), respective merges. Constructs contain SS (black) followed by indicated AVR3a or PH001D5 wild-type and modified sequences (orange) and GFP (green). Fraction of GFP exported to the erythrocyte cytosol is indicated in Bx and Cx. Standard deviations are shown in pink. Export of green fluorescence to erythrocyte is quantitatively blocked on replacement of the regions downstream of the Avr3a and PH001D5 motifs, as indicated in the bar charts in (xi). Standard deviations are shown in pink. p, parasite; e, erythrocyte; nucleus is Hoechst-stained (blue); scale bar is 2 μm. In both (ii) panels, the asterisk (*) indicates intraerythrocytic loop structure that excluded GFP. Note, in the case of PH001D5 (Cviii), a small amount of GFP is detected in tubovesicular elements in the erythrocyte. However, export to the erythrocyte cytoplasm or periphery is never detected.

To examine this further, a logo of secretory RxLR-containing sequences of *Phytophthora* species was created from the 59 protein sequences of P. infestans shown in Figure 3A as well as 176 and 147 protein sequences from Phytophthora sojae and *Phytophthora ramorum,* respectively (Figure 4A, see also Table 1 and Tables S1 and S2). This logo demonstrates that RxLR in secretory proteins is conserved across species (see Figures S1 and S2): the reduced number of P. infestans sequences is likely due to incomplete genome sequencing. More than 1,000 proteins in each of these species contained just an ER-type SS. Hence, RxLR may be valuable in prioritizing ~10% of secreted proteins as host-targeted effectors (Figure 4A). RxLR is present in ~5% of cytosolic (i.e., non-secretory) proteins of *Phytophthora* species (logos for which are shown in Figure 4Aii and Figures S3 and S4), and in organisms such as Saccharomyces cerevisiae it is present at ~1% of sequences (unpublished data). Thus, RxLR is clearly enriched in secretory proteins of *Phytophthora*. Further acidic residues downstream of RxLR are enriched in the secretory set but not in the cytosolic set of RxLR proteins from *Phytophthora* (as noted by the height and prevalence of negative (red) residues in Figure 4Ai but not in Figure 4Aii, and in Figures S3 and S4). The logo suggests that the prevalence of acidic residues peaks at nine amino acid downstream of RxLR but can be detected within ~20–25 amino acids at frequencies above background.

**Table 1:**
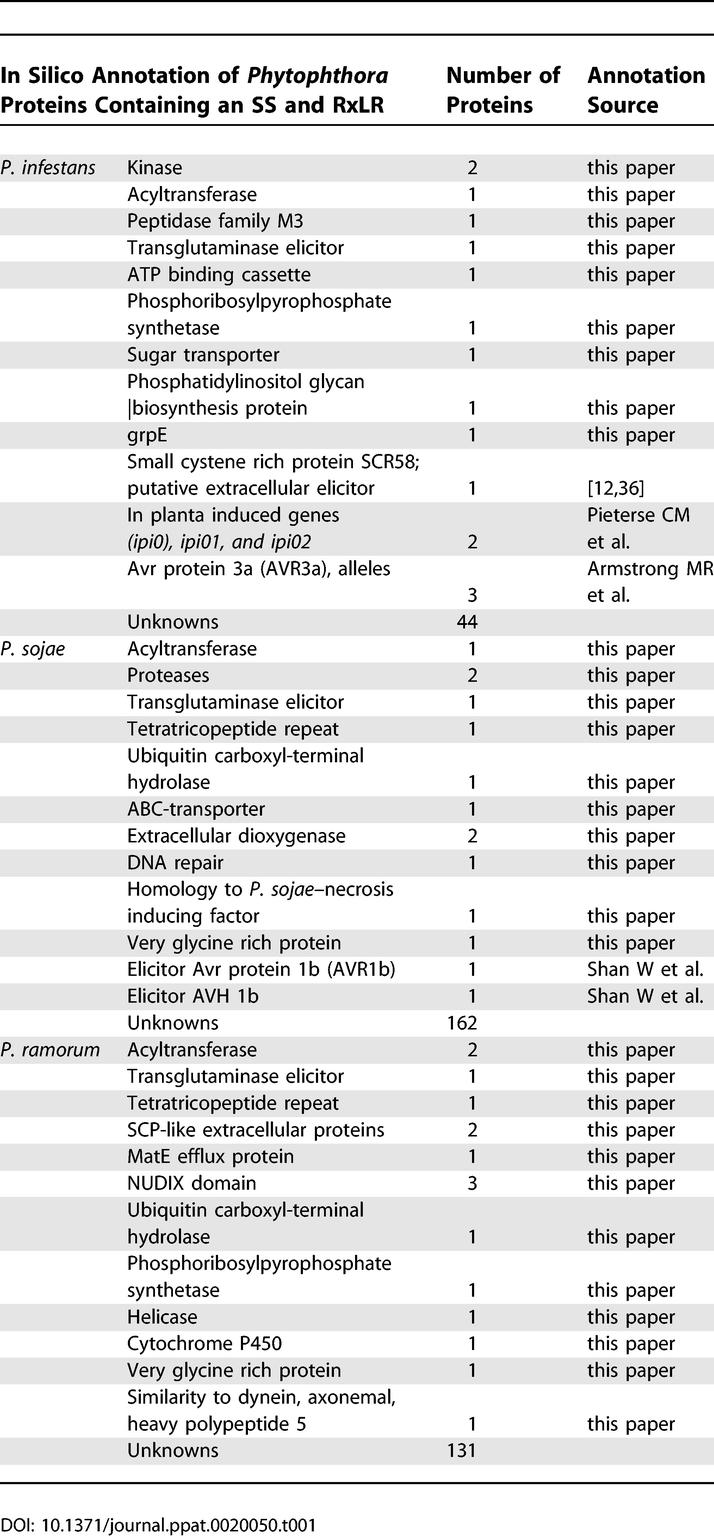
List of Proteins in the Predicted Phytophthora sp. RxLR HT-Secretomes

To test the functional significance of the downstream sequences, we replaced D/E residues in this region with neutral amino acids in both AVR3a and PH001D5. Thus, in AVR 3A we replaced six Es in 22 amino acids. Because of the importance of E/D and Q in the RxLxE/D/Q plasmodial motif, in PH001D5 we also replaced a single Q residue resulting in alteration of seven D/E residues and one Q residue in 25 amino acids downstream of RxLR. As shown in both AVR3a and PH001D5 (Figure 4Bi–4Bvi and Figure 4Ci–4Cvi), these substitutions blocked export of green fluorescence to the erythrocyte cytoplasm. In the case of PH001D5, a small amount of GFP (5%–7%) was occasionally detected in a tubovesicular structure emerging from the parasite, but despite examination of more than 200 optical images per sample, export to the erythrocyte cytosol or periphery (either the host membrane or structures called the Maurer's clefts) shown to be mediated by the HT motif [5] was not observed. Thus, at least a subset of E/D residues downstream of RxLR is also required for export to the host cell. In addition, this function cannot be provided by two randomly selected sequences downstream of RxLR derived from the cytosolic set (Figure 4Bvii–ix and Figure 4Cvii–4Cix). Together, these data suggest that in secretory proteins, sequences downstream of RxLR are conserved for their function in HT, and E/D residues in this region have value for HT. The logo suggests that the E/D residues are distributed over a longer stretch of ~20–25 amino acids in *Phytophthora* sequences. Thus, the consensus HT motif which appears in a short sequence of 11 amino acids with a five amino acid core in *Plasmodium* may extend over ~25–30 amino acids in *Phytophthora* sequences.

### The Linear, *Plasmodium* HT Core Motif Is Dominant but Not Sufficient for Protein Export: Role of Flanking Regions

**Figure 5:**
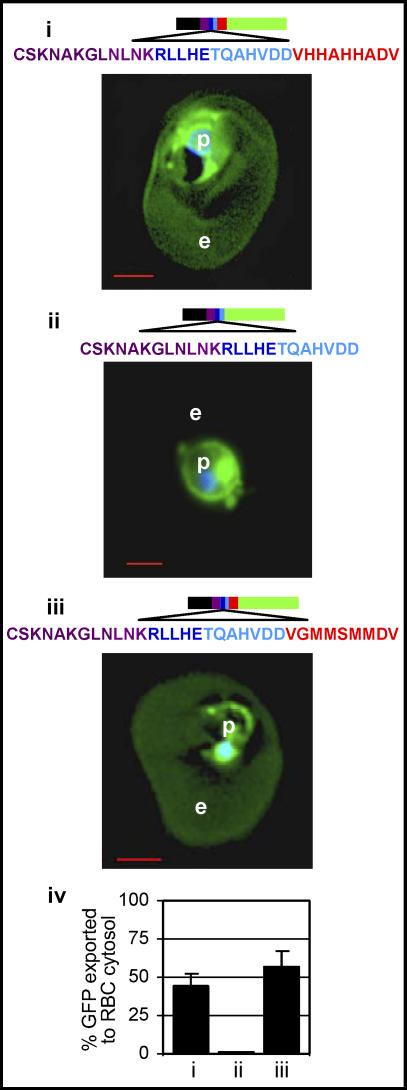
Requirement of Sequences Downstream of HT Motif in Protein Export to Erythrocyte Cytosol (i) Images of live cells exporting a secretory GFP chimera containing the five amino acid HT core (blue) followed by 16 amino acids downstream sequence from PfHRPII (red). (ii) Removal of the terminal nine amino acids (VHHAHHADV) blocked export to the erythrocyte. (iii) Replacement of VHHAHHADV with VGMMSMMDV restored export of GFP to the erythrocyte. For quantitative analyses, two hundred fluorescent images were analyzed as described in Materials and Methods. (iv) Fraction of GFP exported to the erythrocyte cytosol is indicated, and all parasitized cells export the transgene (as expected for stable transfections; unpublished data). Standard deviations are as shown. Constructs contain SS (black), upstream region (purple), followed by sequence containing core host-targeting motif (blue), the downstream spacer region (red), and GFP (green). In all cases: p, parasite; e, erythrocyte; nucleus is Hoechst-stained (blue); scale bar is 2 μm.

The *Plasmodium* HT motif is clearly necessary for protein export to the erythrocyte [5,6,19], but its sufficiency is not well understood. We therefore examined the contribution of P. falciparum sequences flanking the RxLx E/D/Q (Figure 5). We reported earlier that 16 amino acids TQAHVDDVHHAHHADV placed downstream of the five amino acid HT core is needed for export of GFP to the erythrocyte cytosol (Figure 5i, Figure 5iv, and [9]). Deletion of the terminal nine amino acid sequence VHHAHHADV inhibits export to the red cell (Figure 5ii, Figure 5iv), consistent with our prior results on the requirement for the region (~16 residues) downstream of the HT motif [9]. Moreover, replacing VHHAHHADV with VGMMSMMDV has no effect on export to the erythrocyte (Figure 5iii, Figure 5iv), consistent with the fact that swapping of regions downstream of the HT motif between multiple *P. falciparum–*secreted proteins has no effect on HT [9]. Together these data indicate a requirement for approximately 16 amino acids immediately following the HT core region of PfHRPII (P. falciparum histine-rich protein II). But there is a high level of flexibility in specific sequences used, suggesting it may not be specific residues per se that are required, but instead this downstream region may serve as a spacer between the HT motif and its cargo.

**Figure 6:**
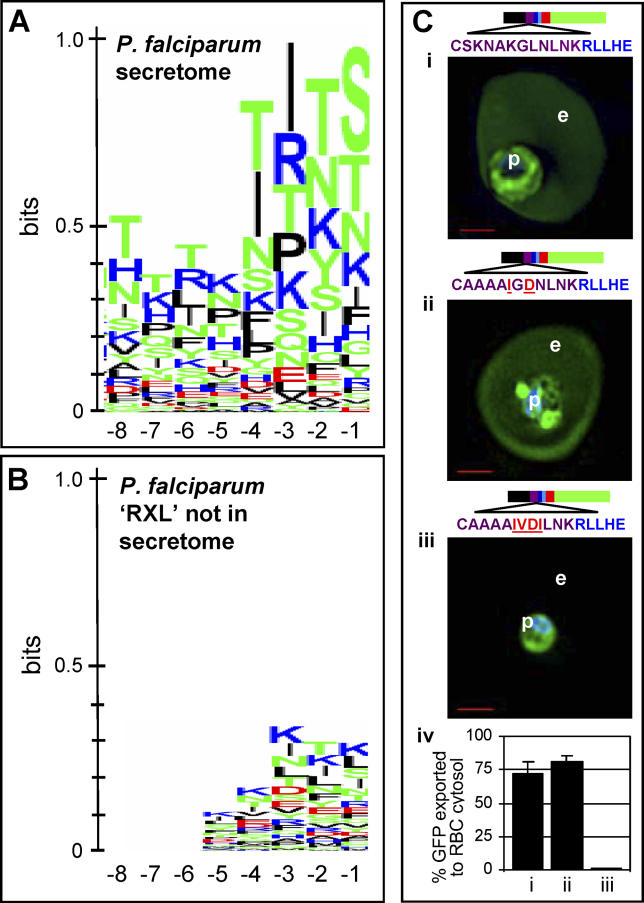
Analysis of Upstream Region of RxL Motifs in the P. falciparum HT-Secretome Logos derived from eight positions upstream of the P. falciparum HT core. (A) Logo was derived from sequences in the predicted HT-secretome. (B) Logo was derived from all proteins containing an SS and RxL alone, but excluded from the HT-secretome. Sequences were aligned without gaps using the RxL motif as an anchor. Amino acids are represented by one-letter abbreviations and color-coded as follows: blue, basic; red, acidic; black, hydrophobic; and green, polar. Height of amino acids indicates their relative frequency at that position. (C) Live cells expressing secretory GFP chimeras of wild-type (i), and mutations in the region upstream of HT core of PfHRPIIGFP IGDN (ii), IVDI (iii). For quantitative analyses, 230 fluorescent images were analyzed as described in Materials and Methods. Fraction of GFP exported to the erythrocyte cytosol is indicated in (iv), and all parasitized cells export the transgene (unpublished data). Standard deviations are as shown. Constructs contain SS (black), upstream region (purple), with indicated point mutations (pink), followed by sequence containing HT core motif (in blue), downstream region (red), and GFP (green). p, parasite; e, erythrocyte; nucleus is Hoechst-stained (blue), scale bar is 2 μm.

Our analyses of upstream sequences in the logos of the predicted P. falciparum HT-secretomes revealed that some amino acids are more frequent in the region upstream of RxL (purple bar in Figure 2A), and we were thus interested in examining the contribution of this region further. This HT-secretome was previously predicted by presence of an HT motif [5] identified by the pattern searching programs MEME/MAST [21,22] and HMMER (http://hmmer.wustl.edu/, [23]), as well as by the presence of a linear PEXEL motif [6] (MEME/MAST and HMMER HT-secretome available at http://www.haldarlab.northwestern.edu). Sequences were aligned without gaps from the RxL, and represented as a logo. The logo in Figure 6A was derived from eight positions upstream of RxL in HT-secretome proteins, while the logo in Figure 6B was derived from all predicted secretory proteins containing RxL alone, but excluded from the HT-secretome predicted by MEME/MAST, HMMER, and PEXEL. Comparisons were also performed on the upstream region of the predicted HT-secretomes of *Plasmodium knowlesi, Plasmodium berghei,* and Plasmodium yoelii versus the upstream region of sequences with SS and RxL alone. Results similar to those in P. falciparum were also observed for these species (unpublished data). The height of basic and polar residues in HT sequences in Figure 6A relative to those found in sequences containing RxL alone (Figure 6B) suggested that they may play a role in HT.

To experimentally verify the role of the upstream sequences, we mutated the upstream region of PfHRPII, an exported protein of P. falciparum. As indicated earlier, in GFP fusions, SS followed by ~40 residues of PfHRPII is sufficient for protein export into the host cell [9] (Figure 6Ci). The presence of alanines at the extreme N-terminus of the leader, and replacement of KGLN in regions −4 to −7 (relative to the HT core) with IGDN failed to block export of green fluorescence to erythrocyte (Figure 6Cii), suggesting that some substitutions are tolerated in this region. However, IVDI in −4 to −7 quantitatively blocked this export (Figure 6Ciii–6iv) despite preservation of LNKRLLHE of the linear HT motif. Thus it is possible to disrupt the HT of this sequence by changing from basic to acidic amino acids in the upstream region; confirming that there is information content in upstream sequences that are needed for protein export to the erythrocyte. Together the data from Figures 5 and 6 demonstrate that the 11 amino acid HT motif and its core are necessary but not sufficient to define the complete vacuolar translocation leader. Rather, the HT motif functions in conjunction with upstream as well as downstream sequences, suggesting that an ~30 amino acid region containing a short linear motif is needed for protein export to the host erythrocyte. This size corresponds well to the size of the amino acid region, reflecting the HT consensus motif in *Phytophthora* species needed for export.

**Figure 7:**
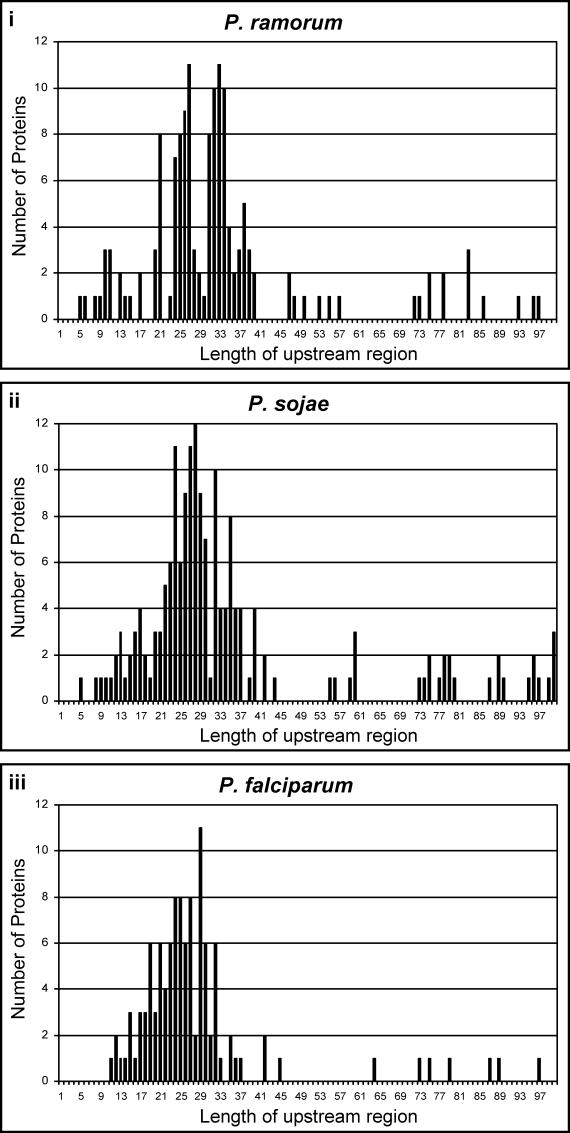
Positional Equivalence of HT Motifs in *Phytophthora* and P. falciparum Histograms plotting the lengths of the upstream regions of proteins in the HT secretomes of P. ramorum (i, 147 sequences), P. sojae (ii, 176 sequences), and P. falciparum (iii, 112 sequences). Sequence distribution data from P. infestans is not shown because the complete sequencing of this genome is still under way.

The positional distribution of the HT motif is also conserved between *Phytophthora* and P. falciparum (Figure 7i–7iii). Notably, although this motif frequently occurs at ~25 amino acids downstream of the SS, it is seen at considerably shorter and greater distances, and, as we have previously demonstrated [5], in both cases it functions in vacuolar translocation of GFP. Conservation of positional distribution of the HT motif between *Phytophthora* and P. falciparum reinforces their shared structural and functional features.

### Comparative Analyses of *Phytophthora* and *Plasmodium* HT-Secretomes

Functional equivalence of their HT signals led us to further analyze and compare *Phytophthora-* and *Plasmodium*-associated HT-secretomes. A prominent feature is their large size, comprising hundreds of proteins, suggesting that like *Plasmodium, Phytophthora* may secrete large numbers of putative effectors. We identified 59 P. infestans (whose genome has not yet been completely sequenced), 176 *P. sojae,* and 147 P. ramorum proteins, all with predicted signal peptides and RxLR motif (Table S1). Subsequent analyses using BLASTP for homology searches against the non-redundant database as well as the St. Louis Pfam database of protein families yielded annotations shown in Table 1 and Table S2 (see Materials and Methods). Several *Phytophthora* proteins had putative functions that could be related to transport or effector activities. These included proteins with similarity to ABC transporters, efflux pumps, proteases, kinases, acyltransferases, and transglutaminase elicitors. Note that a P. sojae transglutaminase elicitor has previously been shown to induce plant defense [24]. There may be enrichment of some cellular functions in the predicted HT-secretome compared with proteins with an ER-type SS (from which the HT-secretome was created), such as the kinases and the acyltransferases, but the precise significance of this is unknown. Like the *Phytophthora* HT-secretome, the P. falciparum HT-secretome also contains an ABC transporter and multiple kinases [5], suggesting related effector activities between the protein sets.

## Discussion

Our results support the idea that although *Plasmodium* and *Phytophthora* are divergent eukaryotes, they share leader sequences with equivalent HT information. This provides the first evidence that equivalent HT leaders may function in infections of eukaryotic parasites, implicating conserved machinery for transport. This finding will facilitate the use of comparative genomics to study host-targeted effectors in eukaryotic parasites and the possible development of new targets for therapeutics against them. It may also signify the eukaryotic counterpart of Type III secretion systems of prokaryotic pathogens.

Although *Phytophthora* and *Plasmodium* belong to distinct eukaryotic groups, recent studies suggest that they may not be as phylogenetically distant as assumed. Phylogenetic analyses indicate that the alveolata (that include apicomplexans such as Plasmodia) and the heterokonts (that group oomycetes with diatoms and brown algae) are sister groups [25]. Similarity in pathogenicity mechanisms between apicomplexans and oomycetes has been noted with regard to secretion of protease inhibitors and attachment to host tissues [26–28]. Our studies support functional conservation in the targeting signals these pathogens evolved to colonize diverse hosts.

The apparent conservation of transport motifs between divergent eukaryotes may reflect convergent evolution or an ancient molecular mechanism. With respect to the latter, a short motif such as the ER-retentive KDEL is well-known to be preserved across phylogenetically diverse taxa and recognized by conserved machinery [29]. However, short sequences tend to be very susceptible to mutation, resulting in loss of function. In this context, it is interesting to note the prevalence of positively charged arginine and lysines immediately surrounding the shared RxL core (blue in Figure 3A) in both P. infestans and *P. falciparum.* This enables redundancy of the much-conserved arginine and may signify a microbial strategy to preserve function despite point mutations and changes in host environment encountered by these signals. In contrast, the E/D/Q in the five amino acid linear core of the plamodial HT motif is not positionally conserved in *Phytophthora* sequences. In PH001D5 which is exported, the first E/D occurs seven amino acids downstream of the conserved RxLR. Further, the logo suggests that in *Phytophthora* sequences multiple E/D residues prevail significantly downstream of the RxLR, and we show that at least a subset of these must play a role in export.

We did not analyze the functional requirement of *Phytophthora* sequences upstream of RxLR, but even in their absence the data suggest that the HT motif in these plant pathogens is not a simple linear sequence but more likely a small domain. Further, our data in P. falciparum that sequences upstream of conserved RxL and peptidic spacer region downstream of the motif are needed for host targeting, suggests that here, too, the complete functional transport signal is larger and approaches the size (25–30 amino acids) of a small globular domain. In contrast to linear domains, globular domains tend to be ordered (alone or after interaction with target molecules), largely conserved amongst species, and have stronger binding affinities [29], and thus could be conserved across divergent eukaryotes such as *Plasmodium* and *Phytophthora*. In this case, the variable yet constrained flanking regions may allow the evolutionary plasticity possibly needed to straddle distinct host barriers, such as crossing the parasitophorous vacuole for P. falciparum proteins and translocating effector proteins across a host-derived membrane during colonization of epidermal cells and the formation of haustorial structures for oomycetes.

All oomycete Avr proteins that are recognized inside host plant cells by cytosolic resistance proteins contain the RxLR motif conserved at their N termini followed by C-terminal regions that are highly variable and under diversifying selection. Moreover, the N-terminal region of AVR3a including the RxLR motif is not required for activation of plant defenses when the gene is directly expressed inside plant cells (J. Bos, T. Kanneganti, and S. Kamoun, unpublished data). These results suggested a function of RxLR as part of a shared pathogenic leader that is distinct from the C-terminal effector domain [11,12,30]. This is supported by our finding that P. infestans leaders containing RxLR motif can mediate host translocation of proteins in *Plasmodium*. The identification of conserved leaders enables discovery of new virulence effectors of the putative HT-secretomes. Our studies with *Plasmodium* revealed that erythrocyte remodeling involves many unique proteins that in the absence of functional assays cannot yet be annotated.

Further, it is vastly more complex than previously realized and may involve heat shock proteins. In addition to the known *Avr* genes, predicted *Phytophthora* HT-secretomes contain ~30 annotated proteins and ~400 unknowns, suggesting that host remodeling by eukaryotic plant pathogens is also highly complex. Approximately 35% of the predicted HT-secretome of P. sojae is homologous to that of *P. ramorum,* suggesting that some effector functions are conserved between Phytophthora sp. yet many others are species- and/or host-specific. Early comparisons suggest significant differences in effector classes between *Plasmodium* and *Phytophthora,* which may reflect colonization of vastly different host cells. Nonetheless, some predicted effectors, such as those with similarity to ABC transporters and kinases may be related between *Phytophthora* and *Plasmodium;* suggesting that related proteins may have evolved effector functions and pointing to candidate components of secretory machinery. At the present time additional similarities cannot be ruled out because it is expected that our predictions of both *Plasmodium* and *Phytophthora* HT-secretomes are comprehensive but not exhaustive.

Sargeant et al. have proposed that the export signal is restricted to *Plasmodium* on the basis of having developed a predictive algorithm that failed to detect pathogenic proteins targeted to the host cell in other microbial genomes [19]. However, this and two earlier algorithms are limited because all three focus on a short linear motif in conjunction with a secretory SS [5,6]. Our data demonstrating that *Phytophthora* HT- motif is equivalent to the *Plasmodium* motif in export GFP to the erythrocyte suggest that plasmodial effectors remain underpredicted and that there is a need to develop a more comprehensive tool. This is especially important if K can replace R in both the first and fourth positions of RxLR. Importantly, our data show that in RxLR-containing sequences significant variation of the RxLxE/D/Q HT core can be recognized and exported by the plasmodial vacuolar translocation machinery, and it is possible that structural studies are needed to accurately predict the critical molecular characteristics of the HT leader.

In addition, studies are needed to establish whether similar or distinct pathogenic secretion signals and associated HT-secretomes function in other intracellular eukaryotic animal pathogens such as *Leishmania, Trypanosoma,* as well as additional apicomplexans *Toxoplasma* and *Cryptosporidia*. Similarly, secretion leaders utilized by haustoria-producing fungi remain unknown, although host-delivered effectors, such as Avr-Pita, AvrL567, and Uf-RTP1 have been described [30–32] and do not contain an obvious HT signature defined here. Emerging genome sequencing and functional genomics in eukaryotic pathogens should reveal the degree to which eukaryotic pathogenic host-targeting signals are conserved across species and whether they span the same host range as the functionally equivalent leaders of *Plasmodium* and *Phytophthora*.

## Materials and Methods

### Plasmid constructs.

Constructs containing *Avr3a* and cDNA PH001D5 along with their respective motif replacements used for transgene expression in P. falciparum 3D7 parasites were assembled in pBSSSGFP [19] and further subcloned into pDC1 [9] at the *XhoI* site. In all constructs, the P. infestans sequences were placed downstream of the ER-type SS of PfHRPII to ensure recruitment into the plasmodial secretory pathway and cleavage of the SS (unpublished data). cDNA PH001D5 is designated as *1D5*. Both *1D5* and *Avr3a* genes were custom synthesized after codon optimization respective to P. falciparum codon usage by DNA 2.0 (Menlo Park, California, United States of America). The translation of *1D5* and *Avr3a* sequences yielded LSAHRAQIMNVATSDLISPIESTVQDDNYDRQLRGFYATENTDPVNNQDTAHEDGEERVNVATVLGKGD and IDQTKVLVYGTPAHYIHDSAGRRLLRKNEENEETSEERAPNFNLANLN, respectively.

1D5 sequence was excised as a *BamHI* fragment and placed at the *BglII* site of pBSSSGFP in three-step PCR. Briefly, PCR1 used HRPIIXhoIF and 1D5KR as forward and reverse primers respectively, while PCR2 used 1D5KF forward and GFPTXhoIR reverse primers (sequence of all the primers are listed in Table 2). PCR3 with products of PCR1 and PCR2 generated SS_Kpn_1D5GFP, which was subsequently digested with *XhoI* and placed in pDC to form pDCSS_Kpn_1D5GFP. For 1D5* constructs containing replacement of DRQLRGF with ISAATAI, we used pDCSS_Kpn_1D5GFP as the template. PCR1 used HRPIIXhoIF and 1D5MUTR as forward and reverse primers respectively, while PCR2 used 1D5MUTF and GFPTXhoIR as forward and reverse primers. The products of PCR1 and PCR2 were used generate SS_Kpn_1D5*GFP by PCR3 which was then digested with *XhoI* and placed in pDC1 to generate pDCSS_Kpn_1D5*GFP.

**Table 2:**
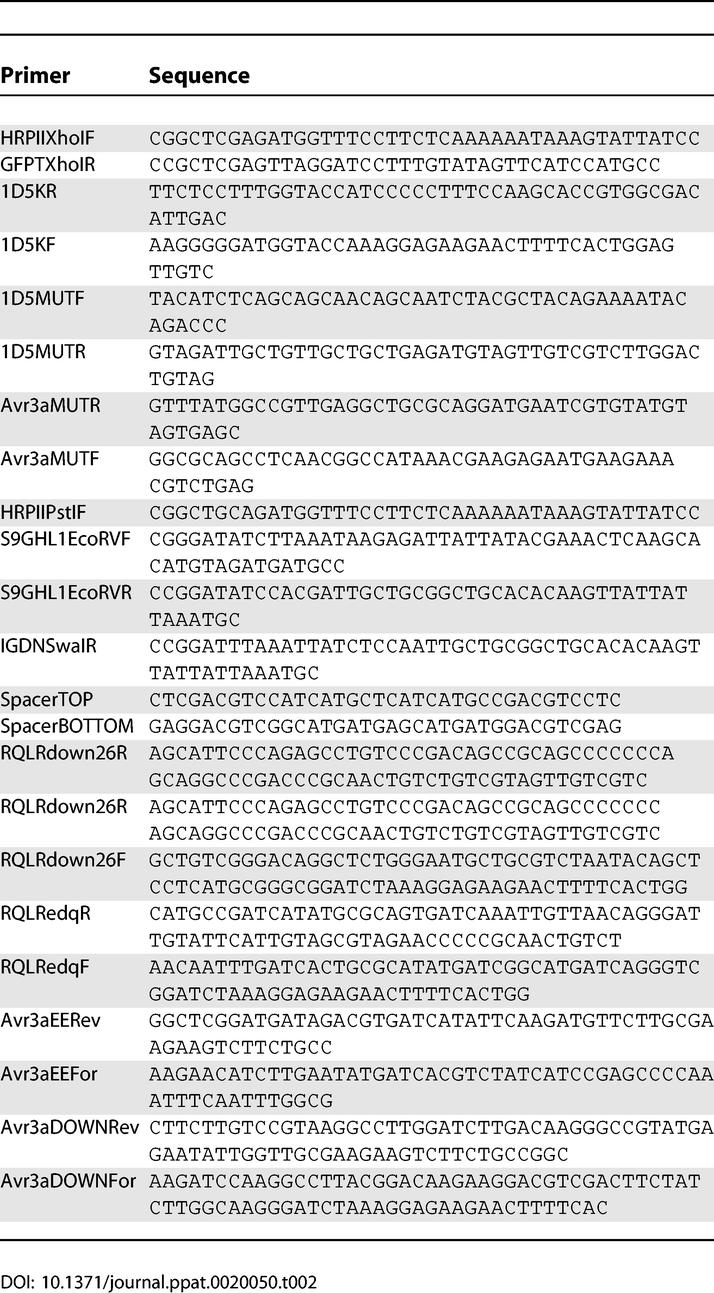
Sequences of Primers Used for Cloning

The codon optimized *Avr3a* fragment was excised as a *BglII* and *BamHI* fragment and inserted at *BglII* digested pBSSSGFPmyc to generate pBSSSAvr3aGFP, subsequently cloned into pDC1.

For Avr3a* construct containing replacement of RRLLRK amino acids of *Avr3a* with AASTAI used pBSSSAvr3aGFPmyc as the template. PCR1 used HRPIIXhoIF and Avr3aMUTR as forward and reverse primers respectively, while PCR2 used Avr3aMUTF and GFPTXhoIR as forward and reverse primers. The products of PCR1 and PCR2 were pooled to generate SSAvr3a*GFP by PCR3 which was then digested with *XhoI* and placed in pDC1 to generate pDCSSAvr3a*GFP.

The construction of plasmids pDCHRPIIDomainIHis.reg._57–124_GFP and pDCHRPIIρDomainIHis.reg._57–124_GFP has been described earlier [9]. pDCHRPIIρ DomainIHis.reg._57–124_GFP was used as a template for two separate set of PCR reactions; one set with HRPIIPstIF forward and S9GHL1EcoRVR reverse primers; and the other with S9GHL1EcoRVF forward and GFPTXhoIR reverse primers. The PCR products were assembled in pBluescript SK (+) to form pBSHRPIIpreHTIVDIHis.reg._57–124_GFP that was further subcloned in pDC1 to generate pDCHRPIIpreHTIVDIHis.reg._57–124_GFP. For the construction of pDCHRPIIpreHTIGDNHis.reg._57–124_GFP, a PCR product with pDCHRPIIDomainIHis.reg.57–124GFP as a template HRPIIPstIF and IGDNSwaIR as forward and reverse primers was used to replace sequences upstream of RxL thus forming pBSHRPIIpreHTIGDNHis.reg._57–124_GFP was further cloned into pDC1 to generate pDCHRPIIpreHTIGDNHis.reg._57–124_GFP.

The construction of pDCHRPIIDomainIGFP and pDCHRPIIDomainImin.his.GFP has been described earlier [9]. An *AatII* site was created in pBSSSHRPIIDomainIGFP sequence just upstream of *BglII* site. Complementary oligos: spacerTOP and spacerBOTTOM were annealed and inserted at *AatII* and *BglII* site to form pBSHRPIIDomainIspacerGFP which was then cloned into pDC1 to generate pDCHRPIIDomainIspacerGFP.

### Construction of PH001D5 downstream variants.

In all cases pDCSS_Kpn_1D5GFP was used as a template. The replacement of the GFYATENTDPVNNQDTAHEDGEERVN region downstream of RQLR in PH001D5 with VGPAGGAAAVGTGSGNAASNTAPHAG, derived from a non-secretory protein of *P. ramorum,* was done as follows. Two separate set of PCR reactions with HRPIIPstIF and RQLRdown26R as forward and reverse primers respectively for the first set, and RQLRdown26F and GFPTXhoIR as forward and reverse primers for the second set. The products of these PCR were used as template to reconstitute entire region with SS, 1D5down and GFP which was cloned in pBluescript SK(+) and subcloned into pDC to form pDC1D5downGFP.

For the replacement of all E, D, and Q residues downstream of RQLR in PH001D5, a similar strategy of successive PCR, with HRPIIPstIF and RQLRedqR as primers for first set and RQLRedqF and GFPTXhoIR as primers for the second set, was used to finally reconstitute entire region with SS, 1D5edq and GFP by third PCR. The region was cloned into pBluescript SK(+) and subcloned into pDC to generate pDC1D5edqGFP.

### Construction of Avr3a downstream variants.

All cloning and subcloning steps to change the region downstream of RRLLRK in Avr3a used pDCSSAvr3AGFP as a template. The replacement of all E residues in NEENEETSEERAPNFNLA of Avr3A with NILNMITSIIRAPNFNLA was done as follows. Two separate set of PCR was carried out with HRPIIPstIF and Avr3aEERev as forward and reverse primers respectively for the first set, and Avr3aEEFor and GFPTXhoIR as forward and reverse primers for the second set. The products of these PCR were used as template to reconstitute entire SSAvr3aEEGFP, which was cloned in pBluescript SK(+) and finally subcloned into pDC to form pDCAvr3aEEGFP.

The replacement of the NEENEETSEERAPNFNLA downstream of RRLLRK in Avr3a with QYSHTALVKIQGLTDKKDVDFYLGK, derived from a non-secretory ribosomal protein L53 (>gi|66270185|gb|AAY43422) of *P. infestans,* was done as follows. Two separate set of PCR, with HRPIIPstIF and Avr3aDOWNRev as primers for the first set, and Avr3aDOWNFor and GFPTXhoIR as primers for the second set, generated products that were used as template to reconstitute the entire SSAvr3aDOWNGFP. The PCR product was cloned in pBluescript SK(+) and subcloned into pDC to form pDCAvr3aDOWNGFP.

### Transgene expression in infected erythrocytes.

*P. falciparum* 3D7 parasites grown in culture were transfected with the indicated plasmids by standard procedures [33]. Briefly, 100 μg of maxiprep (Qiagen, Valencia California, United States) purified plasmid DNA was loaded in 4 × 10^9^ uninfected red blood cells by electroporation [33]. The DNA-loaded erythrocytes were washed and infected with percoll purified schizonts. Forty eight hours after tranfection, cultures were selected for stable expression of transgenes by placement in 1.3–2.6 uM methotrexate and stable cells lines were obtained between 2–4 weeks after drug selection, expanded and frozen as described [9]. In the resulting cultures, all parasites expressed the GFP transgene. For visualization, cells were harvested and nuclei stained with 10 μg/ml Hoechst 33242 for 5 min. After washing thrice with glucose (11 mM) containing phosphate buffer saline, cells were mounted for viewed under fluorescence or brightfield/DIC settings.

### Deconvolution fluorescence microscopy.

Fluorescence microscopy and digital image collection of live infected erythrocytes were performed on an Olympus IX inverted fluorescence microscope with a temperature-controlled stage and a Photometrix-cooled CCD camera (CH350/LCCD) driven by DeltaVision software from Applied Precision (Seattle, Washington, United States) as described in [34]. Briefly, bright field and fluorescent optical images were taken through cells, and DeltaVision software (softWoRx) (American Dynamics, San Diego, California, United States) was used to deconvolve these images). For quantitative projections, a minimum of 200 optical images containing fluorescent parasites were subjected to the “additive” method of data collection. Here, each ray collects and sums data from all of the voxels in its path and scales it down to an appropriate intensity. The data can be used for comparison of intensity in various structures within the image data. Fluorescence quantification was carried out with a 60×, NA 1.4 objective, and polygons were drawn to delineate the whole red cell (mapped by corresponding DIC image) and the vacuolar parasite. Total fluorescence intensity, area, and pixel numbers associated with the parasite and the entire infected red cell were determined. The amount of GFP exported to the erythrocyte was determined by subtracting total fluorescence associated with the parasite from that associated with the infected erythrocyte. To confirm these measurements, a second “volume” method of quantitation was used. For this method, a minimum threshold was set to construct polygons around the GFP signal in all cells expressing fluorescent parasites in multiple fields. These polygons were then applied to the GFP signal in all optical fields and used to obtain intensities for parasite-associated and -exported GFP signal. In all cases the data obtained was within 5% of the first method, confirming accurate computation of the GFP signals in parasite and erythrocytic compartments. Use of a constitutive promoter results in continuous GFP chimera expression associated with the parasite [9].

### Prediction of the *Phytophthora* HT-secretomes.

To predict an HT-secretome for *P. infestans,* we selected available sequences [35] containing: a) an N-terminal ER-type SS; and b) the motif RxLR in the first 100 residues after the SS cleavage site. SSs were predicted with SignalP version 2.0 using the three criteria of Torto et al. [36], except that a hidden Markov models probability cutoff of 0.9 was applied (http://www.cbs.dtu.dk/services/SignalP). The same analysis was performed on the P. ramorum and P. sojae genomes to predict their putative HT-secretomes. These genomes were downloaded from the Joint Genome Institute (JGI) of the US Department of Energy (http://genome.jgi-psf.org/ramorum1/ramorum1.home.html for P. ramorum and http://genome.jgi-psf.org/sojae1/sojae1.home.html for P. sojae)*.*

The first 100 amino acids after the SS cleavage site of 59 P. infestans open reading frames were aligned without gaps using the RxLR motif as an anchor (only sequences that contained at least 100 residues after the predicted SS cleavage site were considered: aligned shown in Figure S1 and sequence IDs shown in Table S1) and analyzed using a program that generates a sequence logo, a graphical method for displaying patterns in a set of sequences as well as information content for each sequence position (http://www.cbs.dtu.dk/~gorodkin/appl/plogo.html; [20,37]. In the logo, the height of each letter is proportional to its frequency at that position; the height of the stack of letters at any position represents the information content (measured in bits) of the sequence at that position [20].

The P. falciparum HT-secretome and logo has been previously described. The set of sequences used to generate the logo were derived by using the pattern identification program Multiple Expectation maximization for Motif Elucidation (MEME) and the Motif Alignment and Search Tool (MAST) [5].

### Analysis of the contribution of sequences flanking the RxL to host targeting.

To compare the HT flanking regions between secretory and cytosolic RxLR-containing *Phytophthora* proteins, sequences were aligned without gaps using the RxLR motif as an anchor. The secretory set comprises 382 sequences from *P. infestans, P. ramorum,* and P. sojae (sequence IDs shown in Table S1, 59 sequences from *P. infestans,* 176 from *P. sojae,* and 147 from *P. ramorum;* alignment of these sequences is shown in Figure S2). The cytosolic set contains 1,681 sequences from the P. ramorum and the P. sojae genomes. The cytosolic set was generated by subtracting the SS set from all the RxLR-containing proteins in the respective genomes, then removing any hits that had a positive maxS score in SignalP 2.0 to eliminate sequences with ambiguous SS (371 sequences were removed). The 1,681 alignments were used to generate six sequence logos, one contains all the sequences together (Figure S3 shows logo in [Ai], and alignment in [B]), and the remaining logos contain four groupings of 382 sequences each and one grouping with 153 sequences (Figure 4Aii shows one of these logos, and Figure S3ii–Figure S3v shows the remaining logos). Logos were generated as described above (http://www.cbs.dtu.dk/~gorodkin/appl/plogo.html; [20, 37]).

For analysis of the region upstream of RxL in the P. falciparum HT-secretome (Figure 6A), we used all secretory proteins that are candidates for secretion into the host. This HT-secretome was previously predicted by presence of an HT motif [5] identified by the pattern searching programs MEME/MAST [21,22] and HMMER (http://hmmer.wustl.edu; [23]), as well as by presence of a linear PEXEL motif [6] (MEME/MAST and HMMER HT-secretome available at http://www.haldarlab.northwestern.edu). Sequences were cleaved at the first residue before their RxL site, and then aligned from the end of the sequence without gaps. Logos show the eight positions upstream of RxL. The logo in Figure 6B was derived from all predicted secretory proteins containing RxL alone, but excluded from the HT-secretome predicted by MEME/MAST, HMMER, and PEXEL [5,6].

The comparative analysis was also performed on predicted proteins from the rodent malarias P. yoelii and *P. berghei,* and the monkey malaria P. knowlesi. Their HT-secretomes include proteins identified by: 1) MEME/MAST [21,22] using the MEME motif generated from the P. falciparum HT-secretome, and searching with MAST against proteins with an SS (Methods described in [5]); and 2) sequences containing the motif Rx(I,L)x(E,D,Q) within the first 100 residues after the predicted SS cleavage site.

### Length of the region upstream of the HT motif.

For the P. ramorum–predicted HT-secretome (147 sequences, Table S1) and the P. sojae–predicted HT-secretome (176 sequences, Table S1), the number of amino acids between the SS cleavage site and the first R in the RxLR motif was quantified and plotted in histograms (Figure 7i and 7ii, respectively). The *P.falciparum*–predicted HT-secretome was selected such that it contains 112 sequences [5] that include one member from the RIFIN and STEVOR antigenically variant families, to avoid over-representation of these families that together contain more than 180 members. The histogram shows the distance between the SS cleavage site and the HT motif (Figure 7 iii).

### Annotation of the *Phytophthora–*Predicted HT-secretome.

All proteins in the predicted HT-secretomes (Table S1) were annotated using the following: 1) the Pfam HMM library of global alignment models (release version 18, http://www.sanger.ac.uk/Software/Pfam) [38] was used with an E-value cutoff of 1e^−10^; 2) NCBI BLASTP was used to search against the NCBI non-redundant protein sequence database (nr). The E-value cutoff was 1e^−25^. Default blastall parameters were used with the exception of the filter that was set at soft masking. Table S2 outlines the details on the resulting annotation. To assess the similarity between the HT-secretomes of different *Phytophthora* species, the relevant datasets were compared to each other using BLASTP, with default blastall parameters, except for soft masking. Proteins with E-values below 1e^−25^ were considered homologous.

## Supporting Information

Figure S1 Alignment of P. infestans Secretome Alignment of 59 P. infestans sequences containing RxLR in the first 100 amino acids after the SS cleavage site. Alignment was anchored on the shared RxLR (bold) and shows 50 amino acids before and after the RxLR.
(13 KB PDF)

Figure S2 Alignment of Sequences from the Phytophthora sp. Secretomes Alignment of 147 *P. ramorum,* 176 *P. sojae,* and 59 P. infestans sequences containing RxLR in the first 100 amino acids after the SS cleavage site. Alignment was anchored on the shared RxLR (bold) and shows 50 amino acids before and after the RxLR.
(30 KB PDF)

Figure S3 Logos Derived from Phytophthora sp.*–*Predicted Cytosolic Sequences Containing RxLR (Ai) Logo was constructed using 1,681 P. ramorum and P. sojae sequences containing RxLR in the first 100 amino acids. The logo for the secretory RxLR proteins was constructed with 382 sequences. Thus, to provide a direct comparison between secretory and cytosolic sets, we derived logos from randomly selected subsets of the same size as the cytosolic set. Thus, (Aii–Av) show logos derived from subsets of the 1,681 sequences.
(43 KB PDF)

Figure S4 Alignment of Phytophthora sp.*–*Predicted Cytosolic Sequences Containing RxLR Alignment of 1681 P. ramorum and P. sojae sequences containing RxLR in the first 100 acids. Alignment was anchored on the shared RxLR (bold) and shows 50 amino acids before and after the RxLR.
(113 KB PDF)

Table S1 List of Protein IDs in the Predicted Phytophthora sp. RxLR HT-Secretomes (19 KB PDF)

Table S2 Detailed Annotation of the Phytophthora sp. HT-Secretome (28 KB PDF)

### Accession Numbers

The National Center for Biotechnology Information (NCBI) (http://www.ncbi.nlm.nih.gov) accession numbers for the P. falciparum proteins mentioned are NP_700633, NP_472949, NP_472948, AAD31511, NP_703354, and CAD51681; for the five amino acid sequences discussed: AAW63768, AAX51201, AAR05402, CAI72345, and cDNA CV917954; and for P. infestans hypothetical protein PH001D5, ID E7.6301.C1.

## Abbreviations

Avr: avirulence
GFP: green fluorescent protein
HRPII: histidine-rich protein II
HT: host-targeting
HT-secretome: host-targeted-secretome
MAST: Motif Alignment and Search Tool
MEME: Multiple Expectation maximization for Motif Elucidation
Pf: P. falciparum
PVM: parasitophorous vacuolar membrane
SS: signal sequence
