# Supplementary material for: The Malarial Host-Targeting Signal Is Conserved in the Irish Potato Famine Pathogen
Source: PLoS Pathog. 2006 May 26;2(5):e50. doi: 10.1371/journal.ppat.0020050 (PMC1464399; doi:10.1371/journal.ppat.0020050)
Supplement: Figure S1 — Alignment of 59 P. infestans sequences containing RxLR in the first 100 amino acids after the SS cleavage site. Alignment was anchored on the shared RxLR (bold) and shows 50 amino acids before and after the RxLR. (13 KB PDF) [file ppat.0020050.sg001.pdf]

Supporting Figure S1: Alignment of *Phytophthora infestans* secretome. Alignment of 59 *P.infestans* sequences containing RxLR in the first one hundred amino acids after the SS cleavage site. Alignment was anchored on the shared RxLR (bold) and shows 50 amino acids before and after the RxLR.

> --------------------LSAHRAQIMNVATSDLISPIESTVQDDNYD**RQLR**GFYATENTDPVNNQDTAHEDGEERVNVATVLGKGDEAWDDALMRLAYQHW
> ---------------------------------LSTNTGVQAANLVGPAQ**RLLR**KHYTAAENDDDSEARALNTEKMKTMLKAGMTVDDYAAKLKLTDKIAAAAN
> --------------------SPGADAVLTGAVSLGFLQLVGADQSVIEQP**RFLR**DGKIAEGDNEERVNAQKEAAAKVLDQVFKTKSLSPLDKLEKTSNLAVIRH
> -------------------IIDAQQVAPSEVSSTDAIQSIYAAQLVRSGK**RFLR**TPTAEQLDDELGQVDEERRMEILTKQLSKLASFTKNTGALKKVDEAVAAA
> ---------------------VSSNLNTAVNYASTSKIRFLSTEYNADEK**RSLR**GDYNNEVTKEPNTSDEERAFSISKSAEYVKMVLYGFKLGFSPRTQSKTVL
> ---------------------VSSNLNTAVNYASTSKIRFLSTEYNADEK**RSLR**GDYNNEVTKEPNTSDEERAFSISKSAEYVKMVLYGFKLGFSPRTQSKTVL
> ----------------------------IDQTKVLVYGTPAHYIHDSAGR**RLLR**KNEENEETSEERAPNFNLANLNEEMFNVAALTKRADAKKLAKQLMGNDKL
> --------------------LTTTVADTAQTATSILTPVLAGEPNKHVAT**RSLR**THPIDDSDDGEERLLNGMTDFFKYHAGKMSPEQLYKYLNLKGLGQEAYKH
> ----------------------------------AALGPPKSSEGTHETA**RLLR**LNAVPQPVETGNQEERTINFASIKKIVPGTSAFKNAQALKASQKAALKAQ
> ---------------------------TRELNMRAAPSDSTRVVDYATTE**RLLR**AHSSDKEEQKEEEERAISINFSSLEKILKNVTSAKTTELQGMLKADEALG
> -------------------------------AVAETSNDINTMNNNQEFA**RSLR**NTEERSIAAILAEAGEEDRAAWRINYRAWYKAKLTPTQVKTVLGVSQAEM
> --KKQQRT**RVLR**KQLTNLRFDLSVAEIETETERILAQMKRVDDEIAALSPSAVTFENTAQKI
> ----------------QTPPGQADKSKLIAHDVLMKTTSLSETTIATSSK**RFLR**LYDAEVRDTVRGDNDVDREERGSSPLLSKVDDLIHKVFKSNPEQAQIKAW
> -------------------------------TTDAQLSDARAVRASFNTK**RALR**SHTKATDHGEERAYKPSLSVVESLNNWMQRASKNILPDDVILVMASKAMT
> --------------------VSTEANGQVALSTSKGQLAGERAEEENSIV**RSLR**AVETSEDEEERDLLGLFAKSKLKKMMKSESFKLKRFGEWDDFTVGYIREK
> ------------------------EQAAAAKELRLNSFVHRSFDAHIHAQ**RVLR**DRRSVDEERGLPTVIEKTKTLFSTKVTDKTLQRWAANKKSPQHALIRLDL
> -----------------------------------SDQNSNVASITSQVQ**RLLR**THHATIKVNADSEERFLTEPPLTTDEMMAMMKAGKSKNAYAFELGIAGQM
> --------------------------ESTVVMNNRNFDSINVPISDDITS**RNLR**ASGEERAYAFVDKIKSLFSRPGISQKVESLQKNPAMVKNLEKAALSQKGS
> ---------------------FTNADDSQLLSKVSPDFAANDMTYTVSRK**RLLR**VAGPEDDDATTDEEDRGLGSIVDVIKRSDAAEALQKLSKASVKKVKQTGN
> ----------------------MEVKTRSGNSDLLVTSSADNIDSSNTAK**RSLR**QVAKASQESVDYLSSKSKYVAAEERGWLTKLFGEKIHLERASEAAYTTLL
> -----------------------------------YTGAAVATQSTYEVK**RMLR**LQVEEVAEEEMADDSECGSLEMAEDDSECGSLEMAEDDSECGSLEMAEPD
> ---------------------------------LVSSKPAMLPPPEELSQ**RHLR**SHDTPVLVDDYNADEERGLDNAKMKSMWKDGWSADSYAPKLDIADDIAHA
> -----------------------NVLHVPTQVTKSHAVSPDAQFVVAMGR**RSLR**TSGEANEERTRLNTLLLLDDVTEAEMSSIKKLASTFAKLENRNDGAADLF
> ------------------ASADSNERLVRAVYSTVRSRDLATDDGPKHTK**RFLR**GESSKIVNLKQEEGVFEERKGVSQKLTKALQAIKARYLKWEQKVLVPAFK
> -----------------------RSATEHAQLMVSQSELDQPTRWNVADK**RLLR**ANDGTNAAEEERGMADIATKMKTWTQSLKTHVGSSKPFQIAAQKWRNTKV
> --------------------SDSEKAAKISNDQVLSGRQLIDTVAKDNKK**RLLR**AYKDAEDDSEDSKNVKPTADSKHADESEDSEDSQEERFSLIQTSNQPRYY
> ----------------------------IDQTKVLMYGTPAHYIHDSAGR**RFLR**KNEENEETSEERAPNFNLANLNEEIFNVAALTKKADAKKLAKQLMGNDKM
> ----------------------------VDQTKVLMYGSPAHYIHDSAGR**RLLR**KNEESEETSEERAPNFNLATLNEEMFDVAALTKKADAKKLAKQLMGNGKL
> --ESVAEG**RRLR**ADAAPVPVNKDNVAKLAGGFLEKLKTNTALTKAANTIKNSNADEAAVRKA
> LRVPWYQDPSRCSGGVRDRLFLRAIRLHGIQRQQGAVLRFRSVRWFGCQR**RQLR**SLLGCPKCPTTMVLQHRRLRLLLVHPVPGEQVRPQVGAHPLDHPADVPAY
> PTQTSETEFQIAEAFETTSNENETTLLDDVVVSASLESEKTVPLPVETKP**RDLR**PVKASTTEHHTESHSLTLPYGGALVAVEATALLVGGATIAVLAISKIKSR
> VAPDAAVDAEQSQPRTNLRGLLNKIVSNDPTQAPVRVLAATWEDGNVGDA**RKLR**RISEKIVGGETVVMFNDRRLRIADEMESAVVNFDVRSNPNCVVSVCGEMS
> --TQGYKMVA**RFLR**LLLHTYFRKIVVYGLNNFPREGPVILCPNHPNMLVDAILVMTEAVSHGRN
> KSNLLRARPSTWQSLYDVQQSNSSVVENDYARGIIVCLHNGIVAMGVSLI**RELR**CLGNTELIQVYHCFPHEMSDESRALLTRNDSKVEIVDVCTDILAKKGPEN
> HRLLALAATMVRRTRAATRPRPLLVTMARSIRMPRLRVPAATTARRTRVA**RKLR**PLLATMAPRTRITVRRLRAVSTSRSSSTSRSSSTSRSKLLSRPRTKRLSR
> LKRRARELGVDAANGDEKQPMNELSDSDDGDAHTEFVPLMVASEKHDVLE**RKLR**AKTSEIRFHFDLSKKMELAEMAELLFNHRGIVAFYACITVYLYGDLAIYA
> ---EDK**RDLR**DLEFGVPHDRKLVDPMSPFFPSDKHPCTHAGMFIPGCHELKIFSGSSHFE
> LYQLRGRHPEMGWVAFFREAYTQVAAAAAQREANNQNRAAQEDARSDTFN**RTLR**TIQVMPTEEFKTPGELKEVTIAELKQRLERRDVDLAGCVERQELVDLLVK
> --------------------------GVTFNVEHLTIPRFTRPVEENLIK**RQLR**VMDDNERGFPSGPSLEKIEAMFQSLTNKITTKSKPSQRDVPDEKLPQLLG
> LDVQVGAVALGALQDLVCATYLSAALWGVDRWIKQQQQAEAEEDESIQKP**RDLR**QRRARQKATVVRFFASFLLFAFMSVPFVADQLLVRIRDMRFNFDLVKMAI
> YTPVELNAPLNASHPAYGAKPNDDCAKPIIPVDPNEAHAKSMTVKNDVAY**RKLR**VMEDSSYSDIDDLSSYFGEQLEVGFKVLKGQFPHATAPNTPWPSSYWPTF
> ------GSGGVKSCDYGQEMFRSGTQVYFLMTIGALVVVMFTVRVLVFDV**RKLR**QPKVRTFVIFDSVWLMFSFASAVAVAAAPVGTAVCSGVDKDIMILLEEVC
> ---------------------------------APMLSASFSSPAASRRA**RRLR**RSKTSAKAADNSSTDPRTPSILADINELSRNGLARRVEMPTGLLERLASV
> ------------IREHGQKKANAAFAMDSEFMTKKQGSRQKVAVNREFYR**RFLR**LFKVIVPGPFTAEVGFAALVAVMLVARTSFDIVVLHTFTAVERAIISRSQ
> --DK**RDLR**RPPKAPRTFNTRAFETALRTPGTGWFHDKFRCDKKSFLRIYRLVHAAWGR
> ----------------------AEAADLTTFPEITSIDHTATTGNDQRAE**RRLR**KRDTDGDASELNQDLQDEARTPQWLAKIVGNLDDVEKAVTKVSKEQSVTN
> LSTQEGMVQRFKMMRARFGRPGGIFGEQILAGNLRTGRLSLDDAKSTSDA**RSLR**DLTPMIGKELELGTTHIGRYLCGWVAVDDAFFGIASSSLILEDVTGYLVE
> -------------------------------------STTAIRDSRGLTR**RRLR**AAALTIEDDKEGRALPTSALTNAAKSVTSKLTGYAQLPVWLITKKSPKEV
> EHALLLKFVADPPTMTMQRLKGLTTGSLPTFLDIKDNMAASSQIVEDNLL**RQLR**AQQRGVVFMGDDTWESLYSREFTRKFAFDSFNVKDLHSVDRGVTAHLFPE
> --**RILR**SLGTYPPSTLINQQVVGVVTTDTQQSTTTVTTRAWKSGSYSASQLGDYKS
> ---------------QTTDRSVGTLVDCPSVRSQDSPCLWAGENGQVVDS**RSLR**ELFIERNYVAYSDKESYGRNLQEHMTYIEDVSMYARQVGHDFSYHMGVND
> --------------------ESAVPDSAATQALQHYDDRLALEEVALVED**RALR**LAHFLATMLAGVPLRKLRARSKTPAWRLFKLDSIASELRWSSSTSTKTNA
> ---------------------------------------ALETWYSSGTH**RGLR**QLTVPVLDERNRFQTCRQFFDNIVKFCPELEFLDGYKKTLNEMDKLVCRD
> AKRSGDQKEAKKLLHLTVRPLDDVKRAQLYQTAARTQVLEVFVDAELTRE**RLLR**CLSGEEAVDPGKKLLDKAFATLERAQNSRDLETAIKLYEEAERVFW----
> --------TCAVPDRRRALPSTEQHLHQQQSDSMSGNQHQVLLAGEYAQR**RPLR**HKSPLAAFTSQLEAQQPPPAKELVHVQYEQQEQEPEQPEKQQPEWPGQEG
> -------------------------------------VPSASPASTSRSG**RNLR**QKMVDELKNVKLRKSPAGKDKNSTRGTPPPLELNEDVDDEEDDVVQDPQA
> PLSADRPTTYSDKLDQLNVIFNVIGTPGEDDIGSLGEVKQYLRKLSKKEP**RDLR**EMYPGAPADSLDLLKQMLSFNPESRISVDKALAHPFLESVRRSQSETVEG
> SQSLAQPGWRLMFGFAGILGALQIALTPLLSESPRWLLNHGEEKEAEHTL**RRLR**QTDDVFDELDNISAASFSESGDVQGVGDVLRDKKI---------------
> SQTPARFADEKPAEETAAKTEAEEEPPLIDAEKLQKQVEELTTQNKDMND**RLLR**ALADAENVRRISRVDVNNAREFAISKFAKALLDVSDNLKRAHESIDVATL
