# Supplementary material for: The Malarial Host-Targeting Signal Is Conserved in the Irish Potato Famine Pathogen
Source: PLoS Pathog. 2006 May 26;2(5):e50. doi: 10.1371/journal.ppat.0020050 (PMC1464399; doi:10.1371/journal.ppat.0020050)
Supplement: Figure S2 — Alignment of 147 P. ramorum, 176 P. sojae, and 59 P. infestans sequences containing RxLR in the first 100 amino acids after the SS cleavage site. Alignment was anchored on the shared RxLR (bold) and shows 50 amino acids before and after the RxLR. (30 KB PDF) [file ppat.0020050.sg002.pdf]

Supporting Figure S2: Alignment of sequences from the *Phytophthora sp* secretomes. Alignment of 147 *P.ramorum*, 176 *P.sojae,* and 59 *P.infestans* sequences containing RxLR in the first one hundred amino acids after the SS cleavage site. Alignment was anchored on the shared RxLR (bold) and shows 50 amino acids before and after the RxLR. Note: this data set was not hand curated and therefore contains a few extremely short sequences. They represent no more than 5% of the total set and have no effect on the resulting logo.

```
> ---------------DVADYESLSTAAQTAKFDPPGVARSFADHDDMASKRLLRSTGTIIDKDDSVDSEERAITIPGLDQLKSLVKKGSSKVDKLIELLKYKFW
> ----------------------DPTSAYRGSKLTTPGAQTQVLQDNTATKRLLRAYTDDEKRGISIPGLESVLKVFPSSKTKQLQGLLKADETLGNAFKTLGLS
> ------------------------------AAEIETTPPDCSDNYGIPTKRFLRTRKSLANDEERSVGTSAVESVLKSTVIDQQIEAVLQKAKSADDAFKRLKL
> -----------------SADSAQIKLSTTGTGTVPAFRSLAGASSEVGGKRSLRTAKEDEADDEADDEADDEADDEADEERGITVFSSGTSAKKLRGFLKKNSI
> --------------------VSGTEKAKLMPVSAVDTRLLTAGQDGGADKRFLRTKTTTEYDGTEVEERAITVGNTLEKAKFYYWKTMGKRPGDIYQKYFKMVD
> -------------------ATDAEHTRIVAMMTAETSVSTYGGQNDGADNRILRAEGTVDDTELSAEDEERESGDFVENVKFYYWLKMGNTPADIYQKFFKGVD
> -------------------------------------------QTSTESARLLRGTVLDSSSSDSDLDSASGSDLDSTSDSDVDSYSGSTSDIINVCPVVVNFS
> -----------------------------AVTASDLSNSIDLVQNNIVGSRSLRIRVATADADDSDDEERAGLESLKSILKSSATLEAKANQLIAKNKELFQQV
> ------------------------------EKSIRSSIITNVAYGRASRGRLLRKADADEANAIDTEERGVLSSIKNWGNMQYWLAVGKSDDQVKKALKMEGLT
> --------------------------------------------VDNTRTRLLRQESAAFANKPAETGGTRKDSTNPLRRRDQALVSAHRVYDPVSGLACSLVG
> -----------------------------SLEASAHPKTTTEPDVGDINTRQLRVTDEEERGILTGVKMAVKTTYWAQMQKSEDFVKKALKLDGLTKGAMKANP
> ---------------------------FELPVYSNDRSITAQQVREENHTRRLRGSTSPDEEERGLLMSIKMAVKTTYLAGTGKSDDIVKKALKLDELTIPAMR
> ---------------LSAATNADQTADISAMGSPDLVTSLEIGNGAGDEKRFLRSHRAEEDDDDSNENENENEEEERAGGANLFTTSKLNEMLSGVKTFDRFAR
> -----------------------------EDIATSRDQKAKTEVADDGTHRHLRGSKKTGGTVAVNEERLALPGVKKAVSKLRSMFSKNPNLAKAVKQKTPGRP
> ----------------------------TIEPDQSSISKPLDTGSAASHERLLRIVKTVNEDDEGDREEERGGFNFGSLTKLFKSSSKTKKAAQVALAAEKQKN
> ------------------DTDSADIKQPLLLPSATSSRMLPIDQNDSVARRFLRTDKMDHEDGDATHDEERGVGIVQSELESLLKVPSHVESMARQAIKGSSIL
> ---------------------------GPVSGPSTASTLVNDFQPEGSSKRFLRSYDMSDLNVNEGAGEEERGIDLTKLDDVINVVKGDKVMDAAKLDDVINAA
> ---------------------------------ATSTSARSINKFESTFKRALRSETKTNVDTSEEEERIVPAPTWLTKFRVWKLKREAGFQLSKTPKQLQKEA
> VGGPGVVAPGVSVGVPGVVGVGGLPYGTDVSVGANALGVGVANTASQKTYRNLRSE------------------------------------------------
> -----------------------------------------LSTEPDQEKRLLRSHKTADVDDAQNEERGIGIGWIDDLFPSEMIKRLKSQLEYRRTLFTSWNS
> ---------------------------------EKSTRSSITTNVAYGRGRLLRKADADTANTIDTEERGVWSSIKNWGKIQYWLAVGKSDDQVKKALKMETLT
> ---------------------------------EKSTRSSITTNVAYGRGRLLRKADAAKANAIDTEERGIWSSIKNWGKTEYWLAAGKSDDQVKKALKIDKLT
> ---------------------------------EKSTRSSITTNVAYGRGRLLRKADAAKVNAIDTEERGVLSSIKNWGKMKYWLAAGKSDDQVKKALNMEGLT
> --------------------TTTSDHTKTSKLTFLGVNSISADQTNGASQRRLRGQELETEGDEEEERGVSTNLLKLDDVFKLDDVFSTKTLRNMIHKATRDAK
> ---------------------VVEVDQTQISKVASANNVRSIGVAPTDVKRFLRRKKTTTNEGTADEERGIKEVASKIDDLLPYIRQVNAKSIDSAIHLLRYQG
> ----------------------TPAVAHSTFTTSTLGRPAIADQNNEPAKRLLRTDLTVEKGEGSDEEERLYIPGLETATNYLKSMIGPTVASPQQLKAWLVGG
> ----------------------------LADSTLTMTRSVTAGRDAISAERFLRTEPTADVNGEERIFKVPSRLTNLFKPGTSSKVTSEAKVASKVITPSVASN
> ----------------------TISSKIKAHNFEAVARPLADNRNDVLPTRLLRTNDLLNQDTEERLYGLEKASEAINSAASKIKMNLNIRTWVKEGKNADNVF
> ----------------------------GSVKTMTTDAEGARSLTSDRYKRLLRADSAVDEERGITSALKSLPGVDKAKKLLLKQQLAAYLKKEKSADDVFSKL
> ---------------------------------QADDVSYSGSGSDENDSRFLRGPDESDEDSEFLSGLDESDDSSELAFGLDPGNDIRDLAGVEDGSFGNDLT
> -----------------------------APTATDAKSVKVVGPGPTDGNRSLRVREASNDNNNDADVNDEERTFNLKQFLGLEPSTNLFAKSKLKKMLKDENF
> --------------------------AVESKQVQLSQMTTANRIESEPDSRLLRAEVDGQERRLQSKYSLTYIKQLVNDKQFRNNNFQSWKDLSVSDITLAKSL
> --------------ASAVTDSEDTKLSQLTSEDIEALTHLLAVESDVDAKRFLRGDTETDLTTAGSDTDALDAEAEERGIVPSSVTNLINKVKNGWSKFKTKAL
> ---------------------------ASTDKKQMAQLDSDIGFLSRDSERLLREGTKKIADDDDDELAADDELAADDEERAIMVKIRGSLSKLRAKYLKWKMN
> ---------------------ASTDKKQMAQRDSEIVFLPRELADNKDGERFLRTQNEKTADGDDDELDADDEERGVMMKIRGFLSNLGAKYHNWKVKLLTPRF
> --------------------LSADTDKGVMVSTMASPDSGLLDVGHNSGKRFLRVHETTNEDDGSEERLSGANMFNTEKIKQAMADSNYAKTLFRRWKRNGFEG
> --------------------LSADTDKGVMVSTMASPDSGLLDVGHNSGKRFLRVHETANEDDGSEERLSGANMFSTKKIKQAMADSNYAKTLFRRWKRYGFGG
> --------------------LSADTDKGVMVSTMASPDSGLLDVGHNSGKRFLRVHETANEDDGSEERLSGANMFSTKKIKQAMADPNYAKTLFRRWKRNGVEA
> ---------------FAVTSNDKHQQVLSTSQALRNRDLADDNTVYANEKRVLRTRQTSDDEDEDAKLKTEDSDSDDEEEERGLTSTLDYPKYWRWFRAHMTPY
> NCDVSVLTEIANTADASTCKTTCNFVVPIESTESSRLADWESFCASDACQRVLRDLSTIHECSIDGASLHQSVVNRIEDDCAKLTRALRAADGSHVHSSGMDMG
> -------------------------LPATTESETDRVSTVAAERSVDSLKRFLRAVPDSDEGEDEERASSLLAKEFVQKMKTMVLKKELLAVAHADEATKLAQV
> -------------------ATEIEQSKMAVPETPAWVIPLAAEGNDGAAKRFLRSETTIAEEEADEEERVMPGTAAAEKLIPKLQKVLSNKQLVSQKEETQFAL
> -----------------------VSAVTEQTSSKVAPPMLSGVQSNVETKRMLRTEEAASEDINTEERRLVNVDLIIKDAVVSLKQAVKWKAEFLVWKLANKKS
> ----------------------LLPATTDQTAFKIATPLLDGEQNNVGGRRFLRTGDAASDNVPNTEERRRLVNIDLILKDAAISVKKATAWKAQFLWWKMINK
> -----------------------VSAVTEQTSSKVAPPMLSGVQSNVETKRMLRTEEAASEDINTEERRLVNVDLVIKDAVVSLKKAVKWKAEFLVWKLANKKS
> --------------------VSTKQNQISETSPEIVARSLAAAQDDAAVKRNLRKYVEEDDLDSLDSLDSLDSFDETEERAKVINVKQLKQLAKTKNAHLPVKF
> -----------------VSPVADPNQSKLFTVHTPDVARVLNVDDEIAKPRFLRAEGNADTDDEERALPASLTKLTDKLKPVAEKVKPLSKKLTPYAEKLTDKA
> AQQAIGQVRDLANANAAANNELNAALQSLLDSAQTFAETDNNGASIFNGIRKLRTEHDK---------------------------------------------
> ----------------------------FQLGSGGRVMWENNCDFTGNDYRSLRGIPAMCGDVCADDSTCTHWSWTNYNSGTCWLKKGARSAKVSKWATNCGYV
> FVADSEGRGRAVEASGLAEIGDRLVAVNEVSLEGLTLQKAVGELQMAELPRTLRFQTHDGRCIQRPAAAIKESVIEAETASAVIYDPTNEENFDYVVSSIGEKS
> ---------------------------STDVGQAELPKTINNDAVNTSHKRHLRSEFAEDLIEWPERIEEYIEHHTRVREIFRAWCMEGKEPKDAMEEAEDGTP
> EETKDKPLEEFVEGQMKKPVGSKQMKMILVLTVLSCFVLSLAPAHAEISQRRLRREDDYTTPEPESPLDVFLTAIQAVEKPEDPRELRSDDDGQEAIENFVKTI
> ----------------------------ASVSDDVKILSAGQAFRVHENKRFLRTTPHRKQTESEKEAGPVFMEHKLEKALSNPKKTKRLYQSWYTRGITAKEV
> -----------------------------ASGATLSKLSTANVESIGGGHRFLRKHKVEDDDTGVDEEEEERGWLASKTVMAKFAVAHTTNNMGEMQKILREVQ
> ------------------------------VSVASESNVAKPKPLDRALGRQLRGFQGKRSLRTFVEATKDDDDSEERVFNEFAKKMAQAAKNKLTSNNQATTD
> ---------------------VSVASESNVIKPKMLDRAQGHQLRGFQGYRSLRTFVEITKDDDDSEERVSNEFAKKMIQAAKNKLTSNNQATTNKLFKSLQVE
> ---------------------VSVASESNVIKPKMLDRAQGHQLRGFQGYRSLRTFVEITKDDDDSEERVSNEFAKKMIQAAKNKLTSNNQATTNKLFKSLQVE
> ---------------------VSVASESNVAKPKTLDRAQGHQLRGFQGYRSLRTFLEATKDDDDSEERVFNEFAKKMAQAAKNKLTSNNQATTNKLFKNLQVE
> AVRSIGGNAVPPNGAATTEGKMVTVTTYDGNGLVQRVGKWWKRTFGGKDARRLRHE------------------------------------------------
> -----------------------GQNPVTRHTHMNNHSYSFQSIPDSKSHRALRSMDTPKQGPIHNGKQKHPHHDDPQQNDKRAHRALLGADGIVSIQRTLGQA
> --------------------ATDADNTKLSQMTTSDTIQSIDAAQAATTKRFLRRYKVTEEDEESEDDLENEESEDDTEDEERTIPNIKVLDDVVENVAKSKVT
> -----------------------GQNPVTRHTHMNNHSYSFQSIPDSKSHRALRSMDTPKQGPIHNGKQKHPHHDDPQKNDKRAHPALLGADGIVSIQRTLGQA
> -----------------------------TPTDSNQAKIVQATLPEGLSQRLLRTYPTTYEDDDSEERGDFTKAEKALFKAMRGNGKTADDYAEELGIAAAMRE
> --------------------LSAGADKGMIVSTMASPDSGLLDVGRNNGKRFLRVHETADGDDHSEERLSGANMFNTEKIKLAIADSKYAKTLFRRWKRYGFES
> TTQDSGDDDSSSDEDDSASNGQEERGAGVSAGTAKKLKKQFSGEYDNVLNRFLRHLFDKMTPSNRRLPDPSKVPAHLRPLRLNADG------------------
> -------------------LSANAKSDLTTLTMTSSKKIQSLAAGQSDGKRFLRSHKNIDFDGEERGVITNPIRRDMLSSSAYKKEVFDNWLLHNKSSATIRDY
> ---------------------ANAKSDLTTLTMTSSKKIQSFAAGQSDGKRFLRSHKNIDFDGEERGIKTSPIVSDMLSDSAYKKEIFKNWLKHNVSSFTARDY
> -----------------------------TTSTVQLKASRVWSSKPQNDRRGLRSNEAVGLENDSVADNDNGTADESGGSEERAIDPAVIAKFEAKMDDVKVEA
> ---------------------------TSISPQPQLVSSAGVGRSTENNNRLLRSREEGGDVDEDSGDQERGLYRSVSRLFSAGDDLKKIEADIVELKAETSFL
> EDISIPIPIIGDALFEDLSNSSTRRSLQAAYPPNGFQTEMLNAVNKERRARGLRELCMNKKLQNAAQGHSNDMAAKDYMAHTSPDGSTMAQRIKDAGYDRTASG
> -----------------------NPITVESSVATPNTLNSNDFAQNDMDSRSLRIRETVDADLEDNSDDEERAGITEVAAKLTKSSSLKSLLDEVETLKTQAHH
> ----------------------------------DSYSAAVSSESTYEAKRMLRIQVEEAAQEEMADDSECGSLEMAEDDSECGSLEMAEEDSECGSLEMAESD
> -----------------------ASVDQTKISMMTTAGVTKAESRPLVDARSLRGYKLSEAEDDERTINVSASIDDVIKRLNAGKIALDYVVNLKKLDKKVLRA
> ------------------------------TANLLVSSVVTADENDIVPDRHLRATNEDDIVPDRRLGATEEDDGNEEERGIATSGLENLAKTTAKKTQQQWWL
> ---------------------AVPADMNSNMAMPYLPTSLATGKNSVSPKRLLRTVDYEEEEDDSEEERGILSGLESITGTVKTKILNAKLRSWLKKGQTTDDV
> ---------------------QTKISAVTSHSSPNWGEELVRDRNGANNGRSLRVGTNTVEERVTWLEKLGKNAKVRWWLETEKTDDYVLKALNLKGLTGSDLT
> ---------------------QTKISAVTSHSSPNWGDELVRDRNGANNGRSLRVNANTAGATWLENLGKHAKVFWWLETEKTDDYVLKALNLKGLSGSDLTKN
> VALVAKSWYHVVDELVARYHRDTMELTFKFGSRVEVLAVRQQVQLRGRAVRDLRVRMGKSDGSRFVTGVWWWMEDREIPWDALFAHMRGLKRLDLRCMPLESCH
> -------LPAATSADQATAISSTGSSTGSSTGSSVLITSHEIGNGAGNEKRFLRSHRVQEDDDDSNDDDDDEEGEEEERAGGANLFKTSKLNEMLSGIKEFKRY
> ------------------ATASGPTTGDSIMASPDLVVSLGTAQDVGGETRFLRKHKDEVSEEEDDDDEEEEERKKGPGFFDSQRLEKMLGNRIYRSKWFNRWK
> --------------LPAATSADQATAISSTGSSTELITSHEIDNGAGNDKRFLRSHRVEKEDDDSKEDDDDDDDEEEEEERKKGPGFFDSQRLEKMLGNRIYRS
> NLDLLAKLQQNGIVALQNVPNYAQLRQEYLRTAANCAVQAQHKGAEFLLHRQLRDGTNRYTVSLESGHQLSDTLAQSQELLATCPEYVQIHSAFSAAVELAVAS
> -------------------------------------QTTPNAPALRHEARGLRLNADTPTTEDADEHRKVHHHVKKVKKIAVPVPVEVPQFIPVPVSVPSTVV
> ---------------------------------------AERRVNGDLTHRDLRVETSAKMSVSDEIEANLRDDNLGASEDSWDHWENHFENPLPRSEPCSSGS
> -------------------ASGDADQAKLSMVTSTDAAVPVRALDTVNGKRLLRSYYEADKDDEADEADEADEYDAAEEERGVMTPAQIRKWTARAEEWVERGK
> ---------------------ISTTKDSNHVAIPNVASSDIARSLEDNTGRHLRRVDNEERTFKEFGGALKKFLEKRTPFTDAWKQRRADKFKLKAKKRYDRMQ
> ---------------------------YRGSKLTTPEAQAQVLQDNTPTKRLLRAYTDDEERGISIPGLESVLKVFPSSKTKQLQGLLKADETLGNAFKTLGLS
> ---------------------------------------------YMIVARFLRLLLHTYFRKIVVYGLNNFPREGPVILCPNHPNMLVDAILVMTEVISHGRN
> ----------------------ATSSDKFSFVTSTDAQSVEATQLKRGDRRFLRTTKMEQEVDDDDDDDDDDDDDDDSQAAEDEERAIPVLGKSISLPASLKNF
> ------------------------DDNEPKTPAPTSSASSRDNDPTIQEIRGLRNAGMKLNDARDFKGSIEKLRGAITLLHGRVFGEARRTVTEPADISQDAAL
> VKSDALQTEQTQPRTHLRGLLDKLVSNSEPTPVPVRQLVAAWEDGKLGESRQLRPITDKIVGGETVVVFNDRRLSPEAKMESAVVAFDVRSSANCVVSVCGELT
> ------------------------------------------------LNRKLRIARGLAPLTGPPGVWLLGNMPVYTKNRDRIYHFLEELLKEHKGRMKMPWH
> -----------------------------------------EAPLAPADARLLRQQVAESVTTDSSSSHSSSSSSSGSGSLNATATINTEASAAETTQHEGPTT
> -------------------------------------------LPASLSCRTLRREQSAASPLLRVISAAFPSHSRCLATQSSDDSDATGAPGVESPGEKFVMV
> FCDLWYFLRMGLNSLTPAARGPRRHLFATSKVQGRIWLMDIDRNGHCNNARFLRECGFGRRDLWQLNGVWEVVTAARGNLVVGSQTVRYRRELSFGQAYTMESR
> SPKLKLNTTLPVEVQYVLYEKALTWRDLGGTLQRLVLWDKGYVVTSSNTTRELRVRCGMNLDDVVVSRDEFQDLHDCPSTSCTDPVSSESVLHGTICADSQIKQ
> ---------------------------NSKATASNFARSVFHSPNDAVAQRLLRTDKAENAAEEERAISVAGVKSGLTKTKTWLQSKWAWIKAKWAGRKLVKQW
```

> ----------------EPATVHVGQPTSPKIAIRDLVDSVAAAPSNADDG**RRLR**AHRLVNGETEVGTARDEERTNLDPKAEKWLASLLTKSDAHLAGLARAPKG
> ----------------------TSTAANSKATASNFARSVFHSPNDAVAQ**RLLR**TDKVEDAAEEERAISVAGVKSGLTKTKTWLQSKWAWIKAKWADRQLVKQW
> -------------------------------------TDSNTGKPETSEG**RLLR**GLQGNFVGTTRNDDESEERAIIDAMKQAARAAKKKFYQTATDKLFKKVKI
> -----------------------AVDTTTKLTNIATSASIADLQTQSNGQ**RYLR**KRETDETDETDNEDRAFDVKKLGSTVKSFFTSKDKKLLKLLISGANVDDL
> -----------------------------ALASARTSDRSFEVISDKSIS**RHLR**PALKTEVDDGTDEDRMMTTFKIWWMKFVATIKNTKLAKKHETMLKSWIQN
> -------------------------ATATRDVTTVLARSPSTGPKDIPIK**RFLR**RHKAANVNENAEGEERGFGITVLDDIARNVGTAWDDISRNSKIRSMLSAQ
> ---------------------DVDQTKLSKMTSPDAAGLSVHAIGAANEK**RRLR**SHYDEDEDEDEDEDEDEDEDEEERIKGVDLFKEWKLDQMLSGIKEFKRYK
> ----------------------------VSTATESEQNTFSAVTAPRKNA**RLLR**TTEMEKDDDLDSDDEERVNFSSITKWFNPVDDVATAAAKKLQPQQVAKLS
> ------GSGGIKSCNYGQEMFRSGNQVYFLMVIGALVVIAFTLRVLVFDV**RKLR**LPSPRALVMFDAVWMLFTFACAVAVAAAPVGTSVCSGVDKDIQTLLEEVC
> ----------------TSAVSNSDKVNVAKLDSGIASLPRALAEHNDQIK**RSLR**RHDDEERQYGTQLIDDVITKVDDVVTKATVAEQKLPSKWTSALKKFRLAN
> ----------------ASAVSNSDKVEVAKLDSGIASLPRALAEHNDQIK**RSLR**RHDYDELAEVDSGNGERMGGAQLIDDVITKADDVVMKATVAEQKLPSKWT
> -----------------VVTDTAGQTKISHISASDVLQAANVAANAVPSN**RRLR**AARATRPDDDNDDDDDDDDEERGFSVSADTVRNMLSSKSFRRSTFAEWDQ
> KVPEPVNGRETPTETEDPRLVAARLARDKQQEAEAHAETVRNFPRGEAEP**RTLR**DHSCMTRVLAGASGYCEVEDEVTGESFRVGRRYCNSIKEEAVFRCSDAVG
> --ANSEAT**RRLR**VDEAGGETRRRLQSKWGVWLAVIKTNLAHLPIEDRLYEQLKRIKEKMDNA
> ---Q**RLLR**SEASTTSDSTVSTDFAALSSGSGFTPGNVAAGSSFGALDHEGSTSGNRPA
> --HSGAALAEEK**RDLR**DLEFGVPHDRKRVDPFSPFFPSDKHPCTHAGMFIPGCHELKIFSGSSHFE
> ----------------------ASRTTLSQLSTANAVDPASARETIGNDR**RFLR**KHKVVEDDDVYGTEDDEDEERGLSDWGKAAADMAASAKALAWFKLNYPGV
> ----------------------TSTASGTTLSQLNTANAVGPTESIGNGR**RFLR**KHEVVQDDEDGEETNDEEEERGIVDSAKALKVMAQLARAQTTDDMARIIS
> ----------------------------------AVNSQTTQVRQEVSTA**RLLR**VRKSSSEDEERGGISTAGVEALISSLKSTVSSEQLSTLAKKSESTDDVFK
> --------------------------SHEVADSKLVTSRINAAQNTLTSK**RFLR**ADSVTGEDDEERAISFKSIPGIEAIKNVFSKKVTLKDLLKYADRKKSSEY
> -------------------------------ATNSKLSASSSIVAEKNVA**RFLR**TSTVADWGNGERGVSIRVTPGVEAVTNKKGTLKNLLKYANRKKSSKSKLK
> -------LPAATSADQATAISSTWSSTGSSTGSSVLITSHEIGNGTGNEK**RFLR**SHRVQEDDDDSNEDDEDDSDEEEEEEERNKGPGFFDSQRLEKMLNNRIYRS
> --------------------------SHEVADSKLVTSRINAAQNTLTSK**RFLR**ADSITGEDDEERAISLKSIPGFEAIKNVFSKKVELKDLLKYADKKKSSEY
> ------------------------------SVATPDTLNSNDFAQNDMDD**RSLR**IREAVDADLEDNSDDEERAGITEVVAKLPKSSSLKSLLDEVETLKTQAYQ
> ---------------------------------EKSTRSSSIPDVGYNRG**RLLR**KTDTAKANAIDTEERGIWSSIKNWGKMQYWLAVDKSDEQVIKALKMDGLT
> ------------------------------ASVVNAKLATSHSTTSHNTK**RFLR**TVNTADENDEERGINFKAIPGVEAIKNVFSKKVTLENLLAYADKKKPSDY
> ---------------------------YRGSKLTTPEAQAQVLQDNTPTK**RLLR**AYTDDEERGISIPGLESVLKVFPSSKTKQLQGLLKADETLGNAFKTLGLS
> ------------------------------ASATTSSKFLPGDQVDSVGE**RLLR**TETAVDKDDSSEERGFVLPGFAQNVAKLAKSRVQNVQARVKQFRVESKFR
> ----------------------ITSSKTSTLSSPAAARSLFVEQHNIVSK**RLLR**AETTADEDEEEEEEGENEEEDEERGITLPSSATEKIAKLAMSSDEIARIQ
> ----------------ASAVSNSDKVEVAKLDSGIASLSRALAEHNDQIK**RSLR**RHDYDELAEVDSGDGERMGGAQLIDDVITKVDDVVTKATVAEQKLPSKWT
> ----------------GTLASSSHRVPTSSITSLDAEHKFPANYFTVDGR**RSLR**ASKSIDDEERVIPAISKWMKTTFWLNTGKSDEHVRKTLGLEGLSGASLKA
> ----------------------ITSSKTSTLSSPAAARSLFVEQHNIVSK**RLLR**AETTADEDEEEEEEGEEEGENEEEDEERGITLPSSATKKIAKLAMSSDEI
> ----------------------------SATSSYQIKTTNLASGGEGPSK**RVLR**SVDAAEADDKDASAAAEERGFQSAVLVCMEVAFTDAYIAMKDGSVVYVKR
> ---------------------------LNINPQASQNLANAATDTHSNSV**RMLR**ADATTGESIDEERGIADIAKKLGTKAKGFMKDRYHWLYVHWKSSAMNQDE
> ---------------------------YRGSKLTTPEAQAQVLQDNTPTK**RLLR**AYTDDEERGISIPGLESVLKVFPSSKTKQLQGLLKADETLGNAFKTLGLS
> ---AQTTPTL**RGLR**LNADTPTTEDADEHRKVHHHVKKVKKIAVPVPVEVPQFIPVPVSVPSTVV
> --------------------ATDADNTKLSQMTTSDTIQSIDAAQAATTK**RFLR**RYKVTEEDEESEDDLENEESEDDTEDEERAIPNIKVLDDVVENVAKSKVT
> -----------------------GQNPVTRHTHMNNHSYSFQSIPDSKSH**RALR**SMDTPKQGPIHNGKQKHPHHDDPQQNDKRAHPALLGADGIVSIQRTLGQA
> ----------------------------LADSTLTMTRSVTANRDAISAE**RFLR**TEPTADVNGEERIFKVPSRLTNLFKPGTSSKVTSEAKVASKVITPSVASN
> --------------------DSADIKQPLLLPSATSSRMLPIDQNDSVAR**RFLR**TDKTDHEDGDATHDEERGVGIVQSELESLLKVPSHVESMARQAIKGSSIL
> ---------------------------FESSVYSNDRSITARQVREENHI**RRLR**GSTTPDKEERGVLASMTAPAITTYLAKTGKSKDVVKKAMKLDQLSEEAMK
> ----------------------------ASVSDDVKILSAGQAFRVHENK**RFLR**TTPHRKQTESEKEAGPVFMEHKLEKALSNPKKTKRLYQSWYTRGITAKEV
> --GKEVGL**RILR**VVETGGHIMFLVMVFMPFGQLKMVAASKVLAVTSALQLKMLALVARQRHQ
> ---------------------------------EKSTRSSITTNVAYGRG**RLLR**KADAAKANAIDTEERGIWSSIKNWGKTEYWLAAGKSDDQVKKALKIDKLT
> --ARER**RSLR**QSLREIWSLVHKRGLSNVLLCRFMGGVCAGVSATAVNPVAFYYAGVQPLN
> ---------------------------------QADDVSYSGSGSDENDS**RFLR**GPDESDEDSEFLSGLDESDDSSELAFGLDPGNDIRDLTGVEDGSFGNDLT
> ----------------------------------DRNPDQLTHFTITGER**RYLR**ADRAAAQGEPASGKKRFSDAKLKKTLNRVKLPPVSMKNRMWNKMLTPMFE
> ----APLELNPYTPVELNSPLTASHPAYGAKPNDDCVKPIIPTGPNDAAY**RKLR**EMEDSYSDIDDLGSYFGGRLEVDFTTLKNQFSYASAPNAPWPGSYWPTFQ
> EAGDAATETSSAQQQETTLPCGLSNNGNMCFVNAVLQCLAANPSFLESVD**RALR**MRAQLHVTRQPDDAHVQKLLVTETLVSLLRGISPVESEEMEVEARGAGQD
> STFKIPLKIDGESTCNINKRSQVTELMRKASLIVWDEAPMAHRHAFEAVD**RTLR**DVLDNEAEPFGGKVVVLSGDFRQILPVVKGGSATETIDACLKSSELWPLF
> -------------------------------TEYSDETNIAMVESPDLVR**RSLR**NGDIAGGRFLRAHEEDDAGERTFSVTDLWNKVAAKKLAKAMLADPSKEQK
> -----------------------ATSATPSAAAHVAEGTFASPATDVGVS**RLLR**GPDEDDNDSSESAAGLEEEDDEDSSETAFDLEADDDDDSSELLDPELDSE
> -------------------------------TEYSDETNISMVESPDLVR**RSLR**NGDIAGGRFLRAHEEDDAGERTFSVTELWNKAGAKKLAKAILKDPSKEQK
> --------------------------------FSSESSAKLSIQNAARGT**RFLR**TAALETTRDDEERGVTMNFAGHFRGNKATKKLLNLWYKTGESEASVAAKL
> -------------------------------------TLTSPEPKHVQAT**RRLR**GAPKTGEIDRSATHSQDTKLDSGAKLTSKLKRVKVPAVTMENEMWNKLLT
> ---------------------ASTTEASKVASPGVLSATGLAVHMSGIGK**RFLR**IREAKDNDDLEGEDRGLVTLVYEGGRNFFKKMTQSAPSVNKVDDQLALVK
> ----------------------VQESTETQLSRMLAIPTIDQKQAASARS**RFLR**EHKATAEENEVDNESEENEVDNESEESDGSEERTGVNKDDELEASLIRVW
> ------------------LITTRTLNHAAISNVASADIVHSIDAIEGNGG**RMLR**KAKEDPAFEEDNEERAGGAFTKWLKHKLEYLKVSAKKRIPRTYENELHRN
> --------------------------ISSDQTKSALATGSGAALEENNLG**RLLR**SLEKVEDDVADEERGFLSGLKKVLGIKGKNPDFAKKNLKMALKDQNSRNE
> -----------------------------VTTSNQVKLTPTTIDRGLGDR**RLLR**SNGANIDAADDEDEERAWLIDMVRNFLARNDFDMTKLSKMMDNEAYKLKM
> PSAWHQPQSLHRQEVNGSNVTVLQPSKDDYQRGFILCLHNGIVAMGVSLI**RELR**CLGNNEIVQVYHCFPEEMSDENRALLTRNDSRVEIVDVCTEILAKKGKDN
> ----------------------SIRSPTENVPGDQSSLHSRAEQVEVSTR**RLLR**ASYNFADADTKAKDAGGEERVGVGVILAHYVQEGASTTAKAAKKAAAVVE
> ------------------------QSSATATVSDPASAIHSPNEAQHTGN**RFLR**TPVSAGGESEGDERGLDWFTSNAAKALKTQKAKWIKNAQIYDDLIMQKRT
> QAQQALQQILSLADANAAANNELNAALQSLLDAAQSFASADDNAASIFNG**RKLR**TEQDK---
> DDTSSAAAAPSAELQDAALPCGLPNNGNLCFANAVLQCLAAVPVFLESVD**RALR**MRSQLHVVRQPDDAQAQKLLVAETLVSLLRGVSPVEEEDVELELELEAEA
> FVADSEGRGRAVEASGLAEIGDRLIAVNDVSLEGVALQKAVGELQAAALP**RTLR**FQTHDGRCIQPPPTAIKESVIEAATASAFTYDPSNEETFDYVVASVGDKT
> MDGAPAIGRLDIVQRLHHERDEGCSAATWKCWSGSTGSTRTCVGGCRSSR**RPLR**MATWTLSSSC--
> --DDTH**RSLR**SSVTTQDAEAEERVDLKGAAGKFSSLVREKSGNVLEKYPSVAAMLGKNPA
> -------------------------LPASDQLQRVRVVSSDLAPSAEAHT**RRLR**SSNVMDEEDGGATDEDEERLLNPSATAKLKAELEAINMKHNFRDWFQVKK
> ------------------------------------QSARRRLDSTLDDA**RSLR**IHFKLKRASMSMYGQLEFDVLANPVVSADKSTVRYDGYATFMEGGTNHTI
> ---------------------LPADQAAVSRATSLTEAFSVVDKEGVEEK**RRLR**FKSTNKGKEDDANEEVEERGGGFNLDLTGVGNLFKAKDPQGIADAAKKAA
> ---TEGDAEK**RLLR**SHKNHKKAASAAVAEEERGMFDFLKNVDDLPMFERMKLQPSYKNHIFESW
> --------------------------ITDESQPRDATIVDAPLTGRGANA**RYLR**TSTSIIKAPDAQLPSTKAAIASSVTKEEEERKISTGLSKLRQKLSKRFHD
> NYNYNSIARPLHPISPFYQRGENFDWGVSRQRGVLVCMHDGVLDIGLSLI**RELR**CLGNQELVQVYHCGQEELSKRSMDLIFRLDKRVELVDVCSDLSTRGVVSP
> ------------------------ADQTQLTTFTRGIDARPIGSTEVDSK**RFLR**RRKHSEDEAAEDEERAGSMDDVVAKLAGLVPTLSKVRKSTVAKGMAYLQQ
> -------------------LPAHTADFDSALSSLTLPGESHATQTVIAAK**RSLR**ALKTTNSPRDEEERDISGLAKKVASWASKTKLTNILQKRMQVKAEKRVQL
> ------------------------------------DAGRVLETTTNEHA**RHLR**TAVASVVDLPDDEDERLLGYNTVQLWRMRRTANKLMNGKLTTQKEAALKK
> ----------------------NPLAASKDSQLMAPGPLAQKLENGILTT**RYLR**VATTDEEEDEERGISAKIPGLEKISSALKSSKTKTKELQALLKADESLGK
> ---------------------------SKESHLTAADALTHALQNETPTK**RFLR**VSTTNKDDYGEQRGISVEKISNVLKSSKTKELEALLKADDTITNAFKKLK
> ---------------------------LVPTTKLDRTVEHPINAAQKNAE**RFLR**TDNAAVLDDVEEERAFANIKGIARTQDEATEWLEHWLGKRLSIEKVANEL
> ---------------------------LVPTTKLDRTVEHPINAAQKNAE**RFLR**TDNAAVLDDVEEERAFANIKGIARTQDEATEWLEHWLGKRLSIEKVANEL
> SATVSGSGSVAEIIEALSVSASASGSGSVSFAEAFKMLSPGSSRPSFGSA**RTLR**SSRESSVADVSVSVSSSASSNSSATVTVGSGASYELATPLTTKSSVVPVY
> --------------GHSPSAGYFPDHLHIEPGVVAESAIIVGLVVCLYGF**RLLR**AMVFACGFLVCGLLVSAALENTFGLKAWVLAASWIGFVVVGIAGGCVALA
> ----------IEIRNLTQRSASLLCGDNSPYSPSIVNVSDIKYHSDHKYY**RTLR**SMPSLQPPLFHGNHAALCRNQIQLKRKYRTFSNCLPISGRKDAERCSGAD
> --------------------ESASTDTTMIATDSVSIRSLTRMDTGENQQ**RFLR**TDEGDARDDEERGWKEVVQTVREKIARAKFPAWYASGKTPEKLVEELQLV
> --------------------------------PPNVKTVLSAEQHDIPVK**RLLR**PGNPAGKEDEERGINFSSVPGFEKLANLLKPKPGLKKLLKWADAKKPPET
> --------------ANGVHVGDNPISNRAHETNHLVTADPVVDERTWVPF**RALR**SMDSPKQGPHDDGKHLSHDPMKGSKHNSPHRALGPAHVTPAPTTTTTKAT
> -------------------AGQESTKAALFLGSSQDLADSVICLSGQSTC**RSLR**ELEGEAEDLANYSILKPLNETSGDKEMVCFGPCCARCYQYRSRRLEEVVD
> ----------------------------AAPTSPPVFQQGHPELGDNSER**RLLR**SFRTDEERAASLAEKLKKLLGLSKTAATNAVDDFAEAIIQKMLKSDKFNR
> ------------VHPTAQLTPRTEAEVADRQRFLSHSHRLLAACSNSEAG**RKLR**EQSNLRIAAKLQELRASRRRLDAATVSAATHKTNLANVSVSTYPENLFGD
> --------------------------------------ESVGPITVPYPA**RLLR**LHELPTDAEERGITINTKQIDDWLEKGDTADDIFKLLSLHMKESNILANP
> ------------VHPIAQQTAQTEGELADRQRFLNHSQRLLAACSNSEAG**RKLR**EQSNLRIAAKLQELRATRRRLDAATVLATAHKTNFTDVIVSTYLEYLFGE
> ---------------------LSAATEIEQAKSSTLLSSQSIVVHEGAKG**RSLR**AVDTEDESSELRGWWTSAKVRWWLESEKSDDYVKGALKLKGLEGDALLNH
> ---------------------------ADTKQTQLLAAAHSTEATLSGNT**RFLR**SHKTQKMEAAEDEERSVNELAAKLDDVLNHVKSVKDLSLNKAMHHLQMAR
> ----------------------------------TSGSSFATIRDDTPAE**RSLR**ISGISASDEERGGPVALEKVKTQLPTSTITEKTLARWAQNNKFPKKALIR
> --------------------------ALTLPQFMINSPKLTGSLLTAQQQ**RFLR**TPYTEDDSIEERGISTVVEKTKALLSSSKVSEQTLERWVRNDKSPKKALI
> ----------------------------ASVATPRLDRSLAAAQRDVTAR**RFLR**IQEGGEERGIATTVEKAKSLFTSSKISQKTLERWVKNNKSPAKVLTRLQL
> -----------------------------ASATSTLKFSPVSLSNDHPEK**RLLR**VETKTGKDNEEERAIAGAAIPNQSKLSKVKKWLSKMKGWSLSKFKEWIKE
> -----------------------------------SADSTNLQTNNVRAG**RNLR**QQPTATQGDDEDRAFNTALVTEGVTNWLKASKVTWTDDQLKALVTKGTSA
> --------------------------------STDSKIVSSGQKKDSFSN**RALR**NGATTVNGEERTFNLEMLAGWANALTRSNVDEQLQKMLAQKTSAEQAFKL
> ---Q**RLLR**SEASAQTDSTVTTTDFTTLGSMGGSSFTPGNVAAGSSFGALDKEGGSMND
> -------------------------------STLAVARSEIGAPSAVYSE**RLLR**SEPQDEDTFEDRAFGLNWLRLRWLRLGAAKAKTTDVISARESKWIEAWAN
> -------------------------ATTTKVVQTASVESANEAHTDNNGT**RFLR**KRAVDNSDDLCTEERGPLTDVLKKATARFSPRQKALKLLKEDADDLVAQG
> ---------------------------EGVARRAQHLENDPHSDPGMNAR**RHLR**GSASAVSKAATSTNPEDEERAITQSLDKFKALFKKSPDLTKLKGQLGTKA
> ------------------------DVAATLSKTTSSYLFADAPDAAGKGS**RFLR**SDVAEDAKRNNQLGEEERANAFTEYLKTIMAKLLGNPKKADLTRTFPSPP
> -------------------------SVNKDVTLSKSTRSDLVHSANAGSV**RLLR**REDQNDESEDGERGLSRAVAEKAANAASKLTKSKSFLSEKAAVQKFARSK
> --------------------------VPGKKNGTATISDSGLNGKLRDNQ**RRLR**TRAHAEERGFSVTALAEAMSKKPSNQKQVTQLFTKVEKGYSKANMNSEQA
> --------------VQSTDRSLGALVDCPAVRSADSSCLWAGVNGDVVDS**RTLR**ELLIEHNYVSYSDREAYRRNLQEHMTYIEDVSMYAKNIGHEFSYHMGVNE
> ---YKIVA**RFLR**LLLHTYFRKIVVYGLNNFPREGPVILCPNHPNMLVDAILVMTEAASHGRN
> -------------------------------LSTATIFNQVEKTTSLGSQ**RLLR**THPDYKVADDEAETEERGPLSREQMKALVKEVGIDRRKVKADFNHLLRHE
> --------------------ALPSATASNIAEVENEASPAIADSTHADGG**RMLR**RVEKNEIDEERIDWSKVWAKLTSKKFTKGQNMSPTEQHKWLIKQAKKLGM
> ------------------------------GIAEQSKLASAPTSHGNISN**RFLR**SHETMEEKEEDPADDEERFLKIPKLARFKGDFAPNKLKRMLKDEDFKLRM
> ------------------------QPTPVKLESDDVLPSNYAETGANTGK**RFLR**GYETVAGEKDSIDDEEERFADVLKILGFKSDFASRYLKNMMKNKDFELKM
> LYQLRGRHPEMGWMAFFREAYTQVSAAAAQRETNNQNRAAQEDARSETFN**RTLR**TIQVMPTEEFRTPEELKDASIAELKQRLERRDVDFAGCVERQELVDLLVK

> ---------------------------AERSEHTQLSTLTSVNAAPMKNA**RFLR**SARTEEEENEEERKIPSYLTHPLLAPEKQVVAAAKAAEKATKLTKYDDEI
> ---------------------ATELKQTQLPKVTAADAVQSVDSISGKLT**RFLR**GVKTVEEEKDESEEDSEEERSIQLKTVDDILADMEAAESNMFVHLSSYDR
> ------------------------------YFVTESKLSLNSEPSDIHFN**RLLR**VVDGEERGRLGGVAVPFLEKAKTVLLPAVSTAKLSQWVTKGKPVNEVFVR
> ---------------------------EFPSVASLDHAPLARKRVSVDGG**RQLR**SVPDSSETEENGVWSWIVKHYQMKAWVANGTPPKQVMKELGLLGLRGNAL
> ----------------------------ASTSEQTTRSTMTSPGVVGDTP**RFLR**KQEAHEERGPQLQKIDDLLANPAAAKAQTVAKLSKIDSAIWKEINSLPSA
> LDAEQPVTVTLADGSTRELTAAEFEQLARERSEQQAKLQPQEEESTRWKR**RELR**DIQDQDPQFALHIDLAKRTFKELQRHGYAQGYALTGFSEQDARPRKRQVT
> -------------------------LSAGPGKAAVSSVVTRSVANAGECT**RFLR**GHRLADGLDDSEEEERGVRIPVKSKMLNNESYRQEIFDNWLKENVSSMRA
> -------------------SDADADADAPNTPEPTSSASARDNDPTIQQV**RGLR**NAGMKLNDAKDFTGAIEKLRGAITLLHERVFGEGRHAVSDPAEISQDAAL
> --------------------------KSSAMVSALHSEAVEAPVSSDATR**RRLR**GTVVVDEERVLGLEKLANSVASIFKRKPGLIQKVEKLQSSPSAVKALEKA
> ------------------LATAEAPNHAAIVNVASADNVHSLDTTAEIGG**RMLR**KVKEDTVSKKDHEERGPGAILERQTAFVKKLFSRQNAIVNRAQGAFQRQN
> ---------------------------------VTGAADDVKILATTSET**RFLR**AADTKTADDTSTINPKGEFIEHKLQRALTNPKKTQRLYKTWYNKGYTAKD
> ---------------------------TRVPDDANLQSVNAPVQTVTRSR**RFLR**TADTDIVYEPKVHNPGKKQVFIEDKLQKALTDPKKNKKLYARWYNSGFTV
> ----------------------------STGLTTTNLAEDVQLAPELGAL**RSLR**GASQDGSKGDEKKPKTEHGDKKKDDEKKKGSDNKKDGHKKDDKEKEKKEK
> -----------------VHVDDNPIAHHSHDTNHLIPSEPVADERTWVPF**RALR**AMDSPKQGPNPHDDGKHAHPHQDLHHDSKKDVKQAPHALGQAHVTPAPTI
> -----------------VHVDDNPIAHHAHASDHLVTSEPVADERTWVPF**RALR**SMDYPQLGHLSDYTHKPRTPTSLDKLTAIKNAHRALGQAQATPAPTVPVT
> LRALAALPTSQPSANEKASWAPLRALKTMEGDQISTSQPAVDEKASWAPL**RALR**TMDNAKQQQQQHPQNHHPNGKPVPKNGVKHH-------------------
> ------------------------------SSEFIQTEHVQPISDQSIGH**RHLR**YTKSTSTAEAEAADEERGIKITDLVILENLAKFDDWVGEGKTAEKLYKEL
> ------------------------------AVHTQEGLNSKIPTPDSEST**RLLR**AHEVADNGKDAGLPADDEERGANAGWVSRLADIDRKTWKMRKLDMKLAEK
> -------------------------GTAVAPSKPKVVNRVDLDKSTGNDH**RFLR**SHKVDYDNSDGYDASDDAEEAGEEEEEEEMGWVVTLKANAVLLRLSMTNS
> ---------------------------ANTKVESSDAVRALADQNNVDVE**RLLR**IVEETEDVEDRAVGASVLQKLKDAVMKIKFSSWYNANMTPTEVNTMLIAQ
> ---------------------------ANTKVESSDAVRALADQNNVDVE**RLLR**IVEETEDVEDRAVGASVLQKLKDAVMKIKFSSWYNAKMTPTEVNTMLIAQ
> ------------------------ETQLSQTIPELVARTLADAPINDAVK**RSLR**RHATLDEEERGGGGYNTKMLVDLAKSKNNKLPGIIAKLSKSGQKTVINTW
> ------------------------------APTAHQNQVQGAIDNKQSGP**RFLR**VDKTADDEERAGEVYHLSKSEKIILVQCIEPTSLPHRSAMESDPQKAWRV
> -------------------------------TENDVTRSLTTGRNGGTKQ**RLLR**STHAGEERGAVSTVGNLAEKAKFRFWKLIGDHPGVIHEKWFKNMDAKDIL
> ----------------------------------VPASADISQLTSEQNK**RSLR**GRQAVVHSDGEKEERRLVNVDLAIKDAVKATNKATVWKAQCAAWKLMGKT
> -------------------------VPDSKVSTSDDSLRTLIDDIHVSSK**RHLR**AADNDEERAVPILGKLTSYLNQKTLDLWLFQGKDPKEVFRILKLVGLKGR
> ----------------------TPASLLTTTDIQRLVHSTVVSQRNLPTT**RLLR**VHKLSSLDDEERGLPVSGLEAVANSVKSTVSVEKLREWLKRGESTDDVFK
> ----------------------TPASLLTTTDIQRLVHSTVVSQRNLPTT**RLLR**VHKLSSLDDEERGLPVSGLEAVANSVKSTVSVEKLREWLKRGESTDDVFK
> ---------------------------------AEALPHADKVEISSMPN**RHLR**SHEPVADEERGGHGLWVSRLAELDQSLWKLQKLGMAGGVKVWLHFGKDLV
> GGWSAAPVTPDTRAALVRALSKTSVCVNSILSARSQVVAGTNYEFQIDGC**RGLR**GGDCSSACKSPRRFSVSVFDQPWTHTTIATKISDS---------------
> --ASTANQVPSS**RRLR**TEVKKLPASYIQGLMHNDDQLKTALNAWKQGKVTTKGLAESLDLSQNKIK
> ----------------------------------DTKTQAGTTHNELLNR**RALR**LHGATTETRASEDDEERIFSVSKIIGLKKASESAPAVEKALKKVVSLEKT
> PNDSDDFVLAGSDDSDNDDSTEAVPYDDIDDDNDEDDDGISDDEYVAADS**RRLR**AAAITPKPTTAPPKATTAAPKATTATPKATTATPKTTPPSPTKPTATNST
> PNDSDDFVLAGSDDSDNDDSTEAVPYDDIDDDNDEDDDGISDDEYVAADS**RRLR**AAAITPKPTTAPPKATTAAPKATTATPKATTATPKTTPPSPTKPTATNST
> ---------------------------------------RNRAPGKLLTA**RLLR**SLKDAEVYGVQEERTGLSAVAEKTSASLKKLKNKVLALGKKTKQTKKLEN
> ------------------------------TLNGVSQAVSIPADEKQSAH**RLLR**SHKSGNVADEEEERSFGSAKDPDAVSTKLGLASVRGLADNLDVGQKWFKA
> --------------------------------------ASASSELVASNA**RALR**SHKTPSVLAAGDEERAAEERAAEEKNLDAKILAKILSDHAYAKQVFRSWL
> -------------------LPEDTRLSKVPIAEHHFLLGGQSGQDGGHVN**RHLR**AGMKNQGSKLASSEERGPIDEVIAGTKKLWGKVKPNHQQAADELFASLKI
> --------------------------VSIDHNIVQPLAQPEPATVSEEEP**RRLR**SSSSPDCGCLSFPAVNAAGETSEGLKGNWGSEGCESAPLGSRVYGRATWH
> ---------------------------------------ASDLTTTDSGK**RFLR**STESNQETEALDSADEERRVNPFPNLKLKIPEIKLDKLKVPFTKKAPVTA
> ------------------------------AKKHPKSRDEHEETVRDKNK**RFLR**LTSEEDTDNVTPGAEDDEEERDLIIPTIDRPKYHRWFHAHMTPYDVKQVL
> --------------------------------ISESTLAYPGHELIENRK**RALR**STQYEKGGEDGSDDLESSKDAADSDDEEERDLIISTINRPKYWRWFHAGM
> -----------------------------DPKSSKLTSADAAALNDGNNG**RFLR**KRDAELDTNDEDKAMAELESMTERAGAAKTNYKLFYKAKMTPTQVAKAIG
> AYHCSAAHVAQAYRHLSETSECLESNCGRFLEEENHHYSNSNTKSRSRTR**RRLR**PAFSFSDSNATNQSAVVGDVHAASEDDKDVTIISVVSCASVTDVTLSIGD
> ---DSAPLVDQG**RNLR**AEQGGLAKADDLIASAAKRNDNAAYFFQTLRNKAILTKADKLEEAGKTVK
> -------------------STSSVTKTIEHDFPAAILSDANYNNGALGAK**RVLR**AHEIPDKENEDRMFNWAKSNAWSFKDKSTSNVLGKIKFDPKVGEGLDSSR
> -------------------------------------ATRLHQPTADAAQ**RSLR**ATNLDEKRGILPDLTKVKGAVAGVKSRIANARLIHWLNDQKSPDYAFTKL
> -------------------APESATAKVNPAKSPSHLRSIATNGRYVPPS**RLLR**SAELPADEERAMKLRLVRWLNRSQQTKISTVDTKQVKKWVKKDDSMASVF
> ----------------------VPESTPIEISSPKSPLSVTSLGRDDPHA**RLLR**AVDDEERAITFPGNSRLVKWLSPSTEAKLPRADKLNVKKWVKNHDEMDDV
> --------------------------DSTPTYGRKGVVALGAHRGDTTGA**RLLR**GVENENEDDASDEDRLYLPGLETVSNYIWSLSGPKVTSKTIDGWLKKGES
> --------------------------SVTDNSIVNAAEYPTVVQNDAPFQ**RFLR**AATDEDEERAISFNSIPGVGKVKNIVDKQKAAQWLKKGKEADDVFTKMKL
> ----------------------------LSSATDSDLALPLAGAQDVAAN**RLLR**ADGDDEERAFNIKTIPGLDKIKSIPGVEKFAGLFKTKITPGTLLKWAKKG
> ----------------------ASAATESKMATPDFRAAGRSIATDQRPQ**RLLR**SHKTIEKADDEKEERVFSIKFLDDFLENNQLKGWLKDSVSARDAIKLTKL
> -------------------------------HQSTTTTQLWTSKLDTPIK**RLLR**AHRTTDDEERGITASGVETLANTLKSTTNIDDELKALVAKGESADDAFKL
> --------------------------ESAATTDRKLITASKLETRQSNGE**RFLR**TSKLTADQDDEDSDEERVFGLKLKSANAEQIKDWLLFRNSADDIFTYLKL
> --EAALPPKS**RLLR**RQVQQNDDADAETTTQLAASNHSSSSDSASSSSSGSLEADATVSTEASAA
> ---------------LSITNSDKKQVSVLDSGKDATSRMLTADYELTNVK**RSLR**GESEKTKQDEERMGNFLVKIKGSIQKLKDAYRAHIEKQFVKLDLIDKKTP
> GVLALRRWSDFVLLKRVSSCNSGSDAWEVAGELQSSNVSWLKIAEVMLAL**RTLR**RRHCCH--
> ------------------SSATTVSDKSTLIQDAAPVHSELSVAVYDSSK**RNLR**SDGVASERDAKNEERGFTPTGVKEAAQMVKTKNSHATHTLYVLNGNSPGW
> --------------------ALPSATASNIAEVENEASPATADSAHADGG**RMLR**RVEKNDEIDEERIDWSKVWAKLTSKKFTKGQNMSPTEQHKWLIRQAKKLG
> --------------------ALPSATASNIAEVENEASSATADSAHADGG**RMLR**RVEKNDEIDEERIDWSKVWAKLTSKKFTKGQNMSPTEQHKWLIRQAKKLG
> NFDLLARLQENGIIALQNVPNFSNLRSEYLRTAADCAVQAQRQGAEFLLH**RELR**DGTNRYTVSLESGRRLSEALDQSAQLLAVCPEYVEAHAAFSAAVELAVAN
> ----------------------------LSTTNANQAKIIKGTSPGGHSP**RLLR**AYQPDDEGDSPEDRTLSKAQVTKILNKLGKDVTWDHVMRNPALFQRYQKK
> ------------------------------DATSMSTTVGERDEVGAAAN**RRLR**GIDTADELDDEDSEERGAVDLSKHADEVFKVLKLDDGVEDLLANPIMKEW
> --------------------------LPTTNSKTFVASAPSLDQAVTQTH**RHLR**AYEDGVNSEEKGFEGITKLAKMLSPNKQEAADKLFINLKLQKEAPDLFGS
> -------------------------LQPGQIALSETESVGAALQHDSAAK**RSLR**IRESTEDYQDEDDEEEDVDEEEEEEEERGWLKSLKFLKWLKKGMNPKHVSK
> -------------------------------------TDASVETESKSAV**RNLR**VQISEQVPAIPAPAGGPTSPRPQSPLQHARHPSSPKAEPQRGNGHESRHG
> --EAVAES**RSLR**AQQNIGAAAAAFKNYKSPNIATKFVEEMTEVGIRGTAARMAAGAKESAKL
> ------------------------------VTTDSVQAKITTVARGGPSQ**RLLR**SYSKPAEENDSADSEKWGFTTEELDDDSEERGLTSGQIESLTKLANKWGH
> ---------------------------SSGVKIPEASVQQGHFHPNNQPT**RYLR**GNENSIWNEDDAANTADEERAITNIDSAFTKSASFNKLKYFTKGEAELNK
> VVGVSGLGVYGTGYGYGPGVYGGVYGPGYGYNGVAPGGATASATANANAY**RKLR**SEEQ--
> YGPGVYGPGVGVYGPGYGYNGYGYNGVAPAAAYPSNGGGATASASATANA**RKLR**SEEEE---
> GPGVVSPGVGVGVPGVVGVGAPTTVVNNGYPAQPSNGGATASATATANAN**RKLR**SE--
> PGVVGVGAPGVVGYNGVNVGAPTTVVNNGYPAQPSNGGATASATATANAN**RKLR**SE--
> ------------------------------IAASPENMASKLSALGVNDK**RSLR**GMKSTDGPADEQRGDLTKIAQKFKAALSKTSIGQKIQAMQLKKIAAREEQ
> --------------------------------APPEGTVSKPSAPGVSGK**RGLR**STDDAADEERADTTKFAQKLETALSKTKLGQKVQESKLAKIMKREALVKI
> --------------------------ESVVKVNALQADAVEAPVDSAITR**RRLR**GTVAEEEERILGLEKLANRVTSLFKTNPGLAQKAENLQKSPSAVKALEKA
> -----------------------------KTSPPAIAHSFIAAKNDAPTT**RMLR**NENRAEGDDEEERAIGDAIGGAITGLASKVQLKFDLRNLLNEGKSADDAL
> -----------------------------VLRPTEAGQVEVANRLRTSPK**RLLR**AASTIIDDEERAIIPVGEQTLLKFSKIVGKKGMQTLAKRLEGKAWEVGKK
> SSRGGFLRNRVAKDSAFLQRSRDFDSSVSRDKAILLCIHNGVLAMGVSLV**RELR**CLGNEELIQVYHCGPEELSDESRELLFSIDDRLETVDVCGDLVQRGVIKR
> ------------------------------LESTNLARSLQVNEHVGNET**RSLR**IHELENEDENDEEEENEGDGEEEERAGGANLFAESKLKQMMSGVKLFKRF
> -------------------------SEAAISTMGSPDSVLSGKNNVNRHA**RNLR**AQKDDEDDDEDDDEDDDEDDDEDRVLTGMKHGGGLTWQKLEDILTNTGEI
> --------------------------EAKLLSEAAPGLAADAVISGESRE**RFLR**VADPEDDDLAAPADDGKTEERAPKFKSLNEIHKKLDEEDMVHVSKILGNM
> -----------------AADSAQTKISVMRSEGPIRSLNAGEDDGDASYG**RFLR**AHKENDVSSSEERVIVLTEMLNKANVKQAAKAILKDPSIEAAVCKEWNAK
> ---QRSPNTPAL**RGLR**ESADTPATTEDNEDWNHHDHHHHHVKKVKKIAIPVPVEVPQYIPVPVSVP
> -------------------------------------VDTTARTHSDITQ**RQLR**VETSPEAIETDLRDDELGASEDFWGSWDNHYAQAQSDPLSNDTSSASADG
> FCDLWYFLRMGLDALTPAPRGPRRHLFAVSKVPGRVWLMDIDRNGHCNNA**RFLR**ECGFGRRDLWQHNGVWEVLTAAGGNLVVGSQTVRYRRELSFGQAYTLQSR
> LHEDASTNPTTTTTATATTTVPTVSRQQDAGLQTQTQTVQTQAQQAEEDA**RRLR**ALEDQPLRREDPRESLLNEAMRAKSFAIAPLADRICCGRWYVAAFLLATL
> --ESANGVSH**RLLR**SASSPEELRGLKAQAVDESDDSEDLTEERGGNGLSSAAAKWKKIRTSVRL
> ----------------------------DETGVTNVNAVHSPNVLAGVDK**RFLR**SHHTEDGEAKLSNYDNEERNGLFGANTLSNMGKDTILRFQMFTKWKANGY
> ----------------------------NSDFLPTKLSHVVADSTNDGSS**RLLR**SGKATGNDSEGDSIGDEERAFDDPAITQYLSLFKDWARNKETPESVYQYF
> ----------------------LSSGEERLTKVTASDFDGLADGDSLARK**RRLR**VDSTGIAEEEEERAFTLIIKLTNRFTKDPAKLEKIAKNKKYRQWLKEDEN
> AIVTNLESFCADESCGHVLEALQDIGECSIDGEGLRQSVVNPIKAVCSAT**RALR**AADGSHQHGSTMEISLGSMEMEDSHDATSSSTATESSEMEDSHDIASSAS
> ---EDVEDVQIS**RHLR**QEFAAKPGETGGVTDSTNPLRRRDQALVSSHRVYDPVSGLACSLVGECVA
> -------------------STSSVTKTIEHDFPAAILSDANYNNGAPGAK**RVLR**AHEIPDKENEDRMLNWAKLNAWPFKDKSTSYVLGKFEFDPKVGEALDSSR
> -------------------ASAKLSSSASAKIDSDIPVRGLVAEYNHVGK**RKLR**GNELVAEDDDDEEERGLPVSSLLNRWKSSLQEIQRTTSYKIKKYLVEKFF
> -----------------AVAGASEDAKVSTMASTMASPDLDVLAVSHSAT**RFLR**GLESVGEVDEDRLAGDKLFHAAKLKRAMDDSKYAETLMKRWKRHGYDIEK
> ------------------------EVDSKTALAAEVPAAIRSLESDTPAS**RLLR**TGTVTSADNEDRGFTESATLLVYPSSQTKLKQLLETATNADDAFSSLKLN
> ----------------------TSHSKITVASTPVSYDVVDPNQVGGQTP**RHLR**ALGIEDDDSSVERAFPELNSMLKIVRPDKQDKAKNLFVRLKLHESTSNLF
> ----------------------------DSRTVVTPSFRTSLRNDIPPTA**RFLR**TGTTTDEDQDARTLNLIIARLESLTNRLKSASQLKVKELVNSGQNIDDAF
> ----------------------ISAPAQNKLGQVAPAGVNDAVHADITTK**RSLR**GLEETLDAADEERGLPELATKLRTWVSNSKLVQAAAKQGQTLDQKLRVAK
> ------------------------VSTDFQLAKVAPATVNNAVHADIGDK**RSLR**GLESTRVDADEERGMTELSAKFKTWIASLKTWISNSKLVQMATKQTQTLN
> ---------------------------------EGYSTAAVSTNSAYQAK**RMLR**LQAQEVAEEEMADDSECGSLEMAEDDSECGSLEMAEDDSECGSLEMAESD
> ------------------SSGFESTMGIVDTNRSVKLDPSVQHIPLDPAL**RHLR**PPPPRVPDDAKLFVGLSVFRDGYRCATTIFTGFTRASNPQNIIFGVVDQV
> --------------ANGVHVGDNPISNRAHETNHLVTADPVVDERTWVPF**RALR**SMDSPKQGPHDDGKHLSHDPMKGSKHNSPHRALGPAHVTPAPTTTTTKAT
> --------------------------------------TKPSTEATAVSK**RLLR**FVEAADEEERRIDFSPEKLRKMLGDETYRLKKFGKWDSDGHTFDGLKHYL
> YGPGYDYNGYGYGYNGYGYNGYGYNGVAPAPAYPSNGGGATASASATATA**RKLR**SAEEQ---
> -----------------------------KSVAVDFPVTIHDSQDAIPTK**RLLR**SSEDSEERVGFDTLTGLAKAGASKVTGTTVLDTWLFHNVNGVQVLAKLQL
> -------------------------ADTTIANVAPSYSLHGPDQAHGHTK**RQLR**CHSLLAVDEDAEERGIANNPSVAKVLYKLTPNKQKAAEKLFKRLKLGKNT
> AREHLGDGARVGDHAHGALHAGQITARHHRRRLVVDAALEARRTPVHELD**RALR**LDGGHGRVHVLGDDVAAVHQTRRHVLAVARVALGHHGRRLERAVGDLGHR
> GVYGPGVYGPGVGVGVGGYGAPAAPAAYNSYPSTNGGGATASASATANAN**RKLR**SQG---
> PYTPINTNFPLTASHPAYGGKLKDDCVEPVIPKDPNQAKAESMVVTGSMY**RKLR**GMEDASNTDVAKLENYFGTSMELDFNTLKEKYGAASAPSSPWASSYWPTF
> --------------------LSAHRAQIMNVATSDLISPIESTVQDDNYD**RQLR**GFYATENTDPVNNQDTAHEDGEERVNVATVLGKGDEAWDDALMRLAYQHW
> ---------------------------------LSTNTGVQAANLVGPAQ**RLLR**KHYTAAENDDDSEARALNTEKMKTMLKAGMTVDDYAAKLKLTDKIAAAAN

> --------------------SPGADAVLTGAVSLGFLQLVGADQSVIEQP**RFLR**DGKIAEGDNEERVNAQKEAAAKVLDQVFKTKSLSPLDKLEKTSNLAVIRH
> -------------------IIDAQQVAPSEVSSTDAIQSIYAAQLVRSGK**RFLR**TPTAEQLDDELGQVDEERRMEILTKQLSKLASFTKNTGALKKVDEAVAAA
> ---------------------VSSNLNTAVNYASTSKIRFLSTEYNADEK**RSLR**GDYNNEVTKEPNTSDEERAFSISKSAEYVKMVLYGFKLGFSPRTQSKTVL
> ---------------------VSSNLNTAVNYASTSKIRFLSTEYNADEK**RSLR**GDYNNEVTKEPNTSDEERAFSISKSAEYVKMVLYGFKLGFSPRTQSKTVL
> ----------------------------IDQTKVLVYGTPAHYIHDSAGR**RLLR**KNEENEETSEERAPNFNLANLNEEMFNVAALTKRADAKKLAKQLMGNDKL
> --------------------LTTTVADTAQTATSILTPVLAGEPNKHVAT**RSLR**THPIDDSDDGEERLLNGMTDFFKYHAGKMSPEQLYKYLNLKGLGQEAYKH
> ----------------------------------AALGPPKSSEGTHETA**RLLR**LNAVPQPVETGNQEERTINFASIKKIVPGTSAFKNAQALKASQKAALKAQ
> ---------------------------TRELNMRAAPSDSTRVVDYATTE**RLLR**AHSSDKEEQKEEEERAISINFSSLEKILKNVTSAKTTELQGMLKADEALG
> -------------------------------AVAETSNDINTMNNNQEFA**RSLR**NTEERSIAAILAEAGEEDRAAWRINYRAWYKAKLTPTQVKTVLGVSQAEM
> --KKQQRT**RVLR**KQLTNLRFDLSVAEIETETERILAQMKRVDDEIAALSPSAVTFENTAQKI
> ----------------QTPPGQADKSKLIAHDVLMKTTSLSETTIATSSK**RFLR**LYDAEVRDTVRGDNDVDREERGSSPLLSKVDDLIHKVFKSNPEQAQIKAW
> -------------------------------TTDAQLSDARAVRASFNTK**RALR**SHTKATDHGEERAYKPSLSVVESLNNWMQRASKNILPDDVILVMASKAMT
> --------------------VSTEANGQVALSTSKGQLAGERAEEENSIV**RSLR**AVETSEDEEERDLLGLFAKSKLKKMMKSESFKLKRFGEWDDFTVGYIREK
> ------------------------EQAAAAKELRLNSFVHRSFDAHIHAQ**RVLR**DRRSVDEERGLPTVIEKTKTLFSTKVTDKTLQRWAANKKSPQHALIRLDL
> -----------------------------------SDQNSNVASITSQVQ**RLLR**THHATIKVNADSEERFLTEPPLTTDEMMAMMKAGKSKNAYAFELGIAGQM
> --------------------------ESTVVMNNRNFDSINVPISDDITS**RNLR**ASGEERAYAFVDKIKSLFSRPGISQKVESLQKNPAMVKNLEKAALSQKGS
> ---------------------FTNADDSQLLSKVSPDFAANDMTYTVSRK**RLLR**VAGPEDDDATTDEEDRGLGSIVDVIKRSDAAEALQKLSKASVKKVKQTGN
> ----------------------MEVKTRSGNSDLLVTSSADNIDSSNTAK**RSLR**QVAKASQESVDYLSSKSKYVAAEERGWLTKLFGEKIHLERASEAAYTTLL
> -----------------------------------YTGAAVATQSTYEVK**RMLR**LQVEEVAEEEMADDSECGSLEMAEDDSECGSLEMAEDDSECGSLEMAEPD
> ---------------------------------LVSSKPAMLPPPEELSQ**RHLR**SHDTPVLVDDYNADEERGLDNAKMKSMWKDGWSADSYAPKLDIADDIAHA
> -----------------------NVLHVPTQVTKSHAVSPDAQFVVAMGR**RSLR**TSGEANEERTRLNTLLLLDDVTEAEMSSIKKLASTFAKLENRNDGAADLF
> ------------------ASADSNERLVRAVYSTVRSRDLATDDGPKHTK**RFLR**GESSKIVNLKQEEGVFEERKGVSQKLTKALQAIKARYLKWEQKVLVPAFK
> -----------------------RSATEHAQLMVSQSELDQPTRWNVADK**RLLR**ANDGTNAAEEERGMADIATKMKTWTQSLKTHVGSSKPFQIAAQKWRNTKV
> --------------------SDSEKAAKISNDQVLSGRQLIDTVAKDNKK**RLLR**AYKDAEDDSEDSKNVKPTADSKHADESEDSEDSQEERFSLIQTSNQPRYY
> ----------------------------IDQTKVLMYGTPAHYIHDSAGR**RFLR**KNEENEETSEERAPNFNLANLNEEIFNVAALTKKADAKKLAKQLMGNDKM
> ----------------------------VDQTKVLMYGSPAHYIHDSAGR**RLLR**KNEESEETSEERAPNFNLATLNEEMFDVAALTKKADAKKLAKQLMGNGKL
> --ESVAEG**RRLR**ADAAPVPVNKDNVAKLAGGFLEKLKTNTALTKAANTIKNSNADEAAVRKA
> LRVPWYQDPSRCSGGVRDRLFLRAIRLHGIQRQQGAVLRFRSVRWFGCQR**RQLR**SLLGCPKCPTTMVLQHRRLRLLLVHPVPGEQVRPQVGAHPLDHPADVPAY
> PTQTSETEFQIAEAFETTSNENETTLLDDVVVSASLESEKTVPLPVETKP**RDLR**PVKASTTEHHTESHSLTLPYGGALVAVEATALLVGGATIAVLAISKIKSR
> VAPDAAVDAEQSQPRTNLRGLLNKIVSNDPTQAPVRVLAATWEDGNVGDA**RKLR**RISEKIVGGETVVMFNDRRLRIADEMESAVVNFDVRSNPNCVVSVCGEMS
> --TQGYKMVA**RFLR**LLLHTYFRKIVVYGLNNFPREGPVILCPNHPNMLVDAILVMTEAVSHGRN
> KSNLLRARPSTWQSLYDVQQSNSSVVENDYARGIIVCLHNGIVAMGVSLI**RELR**CLGNTELIQVYHCFPHEMSDESRALLTRNDSKVEIVDVCTDILAKKGPEN
> HRLLALAATMVRRTRAATRPRPLLVTMARSIRMPRLRVPAATTARRTRVA**RKLR**PLLATMAPRTRITVRRLRAVSTSRSSSTSRSSSTSRSKLLSRPRTKRLSR
> LKRRARELGVDAANGDEKQPMNELSDSDDGDAHTEFVPLMVASEKHDVLE**RKLR**AKTSEIRFHFDLSKKMELAEMAELLFNHRGIVAFYACITVYLYGDLAIYA
> ---EDK**RDLR**DLEFGVPHDRKLVDPMSPFFPSDKHPCTHAGMFIPGCHELKIFSGSSHFE
> LYQLRGRHPEMGWVAFFREAYTQVAAAAAQREANNQNRAAQEDARSDTFN**RTLR**TIQVMPTEEFKTPGELKEVTIAELKQRLERRDVDLAGCVERQELVDLLVK
> --------------------------GVTFNVEHLTIPRFTRPVEENLIK**RQLR**VMDDNERGFPSGPSLEKIEAMFQSLTNKITTKSKPSQRDVPDEKLPQLLG
> LDVQVGAVALGALQDLVCATYLSAALWGVDRWIKQQQQAEAEEDESIQKP**RDLR**QRRARQKATVVRFFASFLLFAFMSVPFVADQLLVRIRDMRFNFDLVKMAI
> YTPVELNAPLNASHPAYGAKPNDDCAKPIIPVDPNEAHAKSMTVKNDVAY**RKLR**VMEDSSYSDIDDLSSYFGEQLEVGFKVLKGQFPHATAPNTPWPSSYWPTF
> ------GSGGVKSCDYGQEMFRSGTQVYFLMTIGALVVVMFTVRVLVFDV**RKLR**QPKVRTFVIFDSVWLMFSFASAVAVAAAPVGTAVCSGVDKDIMILLEEVC
> ---------------------------------APMLSASFSSPAASRRA**RRLR**RSKTSAKAADNSSTDPRTPSILADINELSRNGLARRVEMPTGLLERLASV
> ------------IREHGQKKANAAFAMDSEFMTKKQGSRQKVAVNREFYR**RFLR**LFKVIVPGPFTAEVGFAALVAVMLVARTSFDIVVLHTFTAVERAIISRSQ
> --DK**RDLR**RPPKAPRTFNTRAFETALRTPGTGWFHDKFRCDKKSFLRIYRLVHAAWGR
> ----------------------AEAADLTTFPEITSIDHTATTGNDQRAE**RRLR**KRDTDGDASELNQDLQDEARTPQWLAKIVGNLDDVEKAVTKVSKEQSVTN
> LSTQEGMVQRFKMMRARFGRPGGIFGEQILAGNLRTGRLSLDDAKSTSDA**RSLR**DLTPMIGKELELGTTHIGRYLCGWVAVDDAFFGIASSSLILEDVTGYLVE
> -------------------------------------STTAIRDSRGLTR**RRLR**AAALTIEDDKEGRALPTSALTNAAKSVTSKLTGYAQLPVWLITKKSPKEV
> EHALLLKFVADPPTMTMQRLKGLTTGSLPTFLDIKDNMAASSQIVEDNLL**RQLR**AQQRGVVFMGDDTWESLYSREFTRKFAFDSFNVKDLHSVDRGVTAHLFPE
> --**RILR**SLGTYPPSTLINQQVVGVVTTDTQQSTTTVTTRAWKSGSYSASQLGDYKS
> ---------------QTTDRSVGTLVDCPSVRSQDSPCLWAGENGQVVDS**RSLR**ELFIERNYVAYSDKESYGRNLQEHMTYIEDVSMYARQVGHDFSYHMGVND
> --------------------ESAVPDSAATQALQHYDDRLALEEVALVED**RALR**LAHFLATMLAGVPLRKLRARSKTPAWRLFKLDSIASELRWSSSTSTKTNA
> ---------------------------------------ALETWYSSGTH**RGLR**QLTVPVLDERNRFQTCRQFFDNIVKFCPELEFLDGYKKTLNEMDKLVCRD
> AKRSGDQKEAKKLLHLTVRPLDDVKRAQLYQTAARTQVLEVFVDAELTRE**RLLR**CLSGEEAVDPGKKLLDKAFATLERAQNSRDLETAIKLYEEAERVFW----
> --------TCAVPDRRRALPSTEQHLHQQQSDSMSGNQHQVLLAGEYAQR**RPLR**HKSPLAAFTSQLEAQQPPPAKELVHVQYEQQEQEPEQPEKQQPEWPGQEG
> -------------------------------------VPSASPASTSRSG**RNLR**QKMVDELKNVKLRKSPAGKDKNSTRGTPPPLELNEDVDDEEDDVVQDPQA
> PLSADRPTTYSDKLDQLNVIFNVIGTPGEDDIGSLGEVKQYLRKLSKKEP**RDLR**EMYPGAPADSLDLLKQMLSFNPESRISVDKALAHPFLESVRRSQSETVEG
> SQSLAQPGWRLMFGFAGILGALQIALTPLLSESPRWLLNHGEEKEAEHTL**RRLR**QTDDVFDELDNISAASFSESGDVQGVGDVLRDKKI---------------
> SQTPARFADEKPAEETAAKTEAEEEPPLIDAEKLQKQVEELTTQNKDMND**RLLR**ALADAENVRRISRVDVNNAREFAISKFAKALLDVSDNLKRAHESIDVATL
