# Supplementary material for: The Malarial Host-Targeting Signal Is Conserved in the Irish Potato Famine Pathogen
Source: PLoS Pathog. 2006 May 26;2(5):e50. doi: 10.1371/journal.ppat.0020050 (PMC1464399; doi:10.1371/journal.ppat.0020050)

Supporting Figure S3: Logos derived from *Phytophthora sp* predicted cytosolic sequences containing RxLR. Ai. Logo was constructed using 1681 *P.ramorum* and *P.sojae* sequences containing RxLR in the first one hundred amino acids. The logo for the secretory RxLR proteins was constructed with 382 sequences, thus in order to provide a direct comparison between secretory and cytosolic sets we derived logos from randomly selected subsets of the same size as the cytosolic set. Thus Aii-Av show logos derived from subsets of the 1681 sequences.

Note that in these subsets we detect a low level of polar residues downstream of the RxLR, however the height of these residues is much lower relative to the secretory set (Fig 4Ai), and negative residues (red) are not enriched.

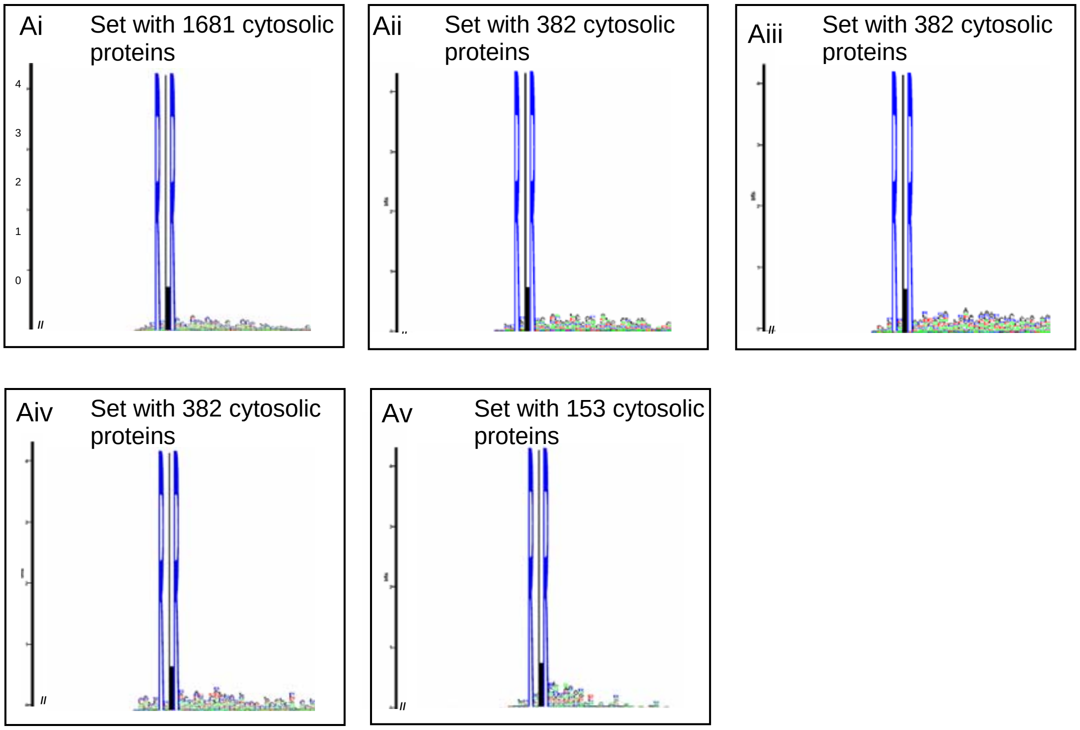

Supplement: Figure S3 — (Ai) Logo was constructed using 1,681 P. ramorum and P. sojae sequences containing RxLR in the first 100 amino acids. The logo for the secretory RxLR proteins was constructed with 382 sequences. Thus, to provide a direct comparison between secretory and cytosolic sets, we derived logos from randomly selected subsets of the same size as the cytosolic set. Thus, (Aii–Av) show logos derived from subsets of the 1,681 sequences. (43 KB PDF) [file ppat.0020050.sg003.pdf]
