# Supplementary material for: The Malarial Host-Targeting Signal Is Conserved in the Irish Potato Famine Pathogen
Source: PLoS Pathog. 2006 May 26;2(5):e50. doi: 10.1371/journal.ppat.0020050 (PMC1464399; doi:10.1371/journal.ppat.0020050)
Supplement: Figure S4 — Alignment of 1681 P. ramorum and P. sojae sequences containing RxLR in the first 100 acids. Alignment was anchored on the shared RxLR (bold) and shows 50 amino acids before and after the RxLR. (113 KB PDF) [file ppat.0020050.sg004.pdf]

**Supporting Figure S4: Alignment of *Phytophthora sp* predicted cytosolic sequences containing RxLR**. Alignment of 1681 *P.ramorum* and *P.sojae* sequences containing RxLR in the first one hundred amino acids. Alignment was anchored on the shared RxLR (bold) and shows 50 amino acids before and after the RxLR.

Note: This entire data set was not hand curated and therefore contains a few short sequences. They represent no more than 2% of the total set and have no effect on the resulting logo.

> -----------------------------------------MLSSLRRST**RVLR**ASSSAQSLLKAPATASQLQRRTFLTSPRVTSYVMARAKVLHLAARSFLAY
> AAPRGPEPGNKRKRKRLSKSKSSSAPASPSGVDAQLQTLENWHRALTVDV**RELR**REGARVRLELEKVDDTAEAAVEKAESCAAQAKAATQARDAAQQREDKLSK
> ----------------MVATLQQELHALRVDREQERETAKNLQKFLAGQV**RELR**TASAQQPSGSVSAQEVGSAPMVRPDSGRTAAHSDRSSASGNAADAIHQKP
> -------------------------------------------------M**RPLR**NNHGDTSVSAATFAELVRKCGLSKKRLHQQLYPMEQGRSSPTDEFPIAVA
> ASRAPTKKRRRLSPPRDSSEELICALTGSSPAPPRPRAANGAALTATHEI**RLLR**SQVAEMEEQLRVLQRQWAQHLPDQRTLAIAQRSAREKHGAVQTEAAHNEL
> ILESKAKLVAGDDVRDKFRTKLSTYLKRLGVKVVVGERLTERLSGNSFEK**RTLR**TDKGTEIESDVQLLCGGFSPTTELIQKLDASLVTPEGFVKVNSKLQLDSD
> -----------------------------------------------MLS**RPLR**SFAPLRRLLSSSAIAANEPTSLWQDVYNLDATHCHDPLVSEGDLQVVLDV
> -----------------MRMLSSVPGTSLDKLPTGVSFDAPKPREVAPFL**RSLR**RMLENESDEVLRWTPDGRAFEILDMARMQDEVLPKYFKHRKYTSFQRQLN
> -MATAPARCSSRALCAKLQPPRTTSAMAPRISAAFFSCGWSARGGSAQTK**RVLR**AWGSRHPNVASGQALRPLLPSHDTNANSWAAVSIAGSDTTSACDGICASG
> HYGECKPASKVFLACMHDEEGMKKTWTGETNESRMEVEGSMATERTVQSL**RCLR**QRERLAKDGCYVLGLSHRELPSHWTNEQVVAFAVNRGAVDESLSTLGLTL
> RVAVVTRVDDSPEEDFPIDVDTREVIPKDMMMKRVLDRFGVPVEAEVRTW**RKLR**TYNLVSGSFRAPSRAIALKEALENAVKASVEALHDLLPRVREEIVPESPL
> AQEQGVAGYDPNEYWRIDADAYRIFMELAPHAQGESVELGQLPELCHQVG**RPLR**DDHEQFRLMQELDTTNAMVILRHDFVTWLLNEIHAEERARIEAAPRPIPD
> GASIRPTKKRRRLTEVPDTNEELILTLKGGRPRGRRRDPNKTPLTATHEI**RRLR**AQVVSMEEELQGLQTKWAKQLPDEHTLVTAHRSAREKHAANLADEAHTEL
> EATAQATKKRRRPPHTNEDIIRTLTGSADTRPRGRARATNKAPLSATHEI**RRLR**AQVASMEEELGELQFKWAEQISDSRTLASAQHTAREKHEADQTEATHNKL
> SSSSDIADRNTKRRRKVSNAQTAVVCALNGSRRPQRRPRAANKRGSAHEV**RMLR**AQVASMEEELRGLKLKWTATLPDENTLAAAYRATLKKYAVIQTETTRREL
> AAPRAVLTPSSAWQEDRYSYDAYDPEVPAMMDSQGEELLPRPSLLAFRPS**RMLR**VSQQQHQNAVYKQQLLRAIGRESDDSSMRSPARASDTSDDDWYEGSDAGS
> LSGTPEAREFLKDLQREYRTILEYRSTLQDVAVAANKVQETQRRSKAAKE**RRLR**QEREEQAIAVEEEAAKRSTAVATI--------------------------
> -------MGLKKWLGLDFSASQPVGHSVKGAGEEDEPLAFGRPEHQPPRR**RQLR**AETSSSHRRHTAWEARNSTPAPTRRSAPLQSPPDVYAQQPERRFSSTSTD
> ------------MAGDRIASTLADDARWHARLAALLAASGKFESAASHYR**RALR**GLEETTGGAPEDDAQRQRRRLQWQFELAATETKRGRRQDAIVLYEGILQV
> -------------------------------------------MGVDGFL**RSLR**DAVDQTHLQQFKGQTLVVDALSWLHKACYGCAFELSTGKNTDTYVQYMLR
> --------------------------------------------MAPRVD**RRLR**LAFIRQLPWAQQVTELAMGSALSMQKFLATPLAAQWKFPFVVNPRFCRRR
> ---------------------------------------------MTSHN**RRLR**STSVSFGVGSIPRPNFDAEPPTSIRNVTLQRRSSSPRVPSQWRRADSVME
> ------------------MSSANQDEEPQDEQDDSQEEQQDEEEAARQEG**RRLR**TAITEASDAREELQQRNAKLQRRIATLLQKTQEGASAADTATAPRREDKA
> -----------------------------------MHQFVRARPLGGQQL**RRLR**ELREAGDNNVRERHLKRDGGVSAPLQQPGDALVSRTVDAIVYERQQDRDG
> PSLQSLNLSNNALRDVCHLQLLPCSLTQLDLGANRLATLPLELGIWLPEL**RLLR**VRSNALESLRPLTGCLQLQTLDAGDNRVALVSELRFLESLGQLRQLLLDG
> MAAIVEDPPLELTQRTRAMDRELKPWALYDLSGAKSPGSLEEMMGYFRRF**RALR**GKSLTGVSHDALQRSWCAMIVRWNRMHQEGSNFVAWLTSREEVVGEHSLS
> DGFYQILMRESDVPLTAVSTPSGMLWEWLVMPQGLKNAPATFNRMVSHVL**RPLR**DFAPSYFDDIFVHSHAEEGLSAIDVHLRHLRQVFQVMRENKLYANLKKCV
> ----MESRLAASGAFYGRLIPQTTDEEQVAAALAALEAASDAWAPSVTRN**RSLR**NAVLGESRFYRVLQEAKLYAIDFGGVCEDEDAAEMDEMRRRAPSLAESDY
> SEDEHDRFLLAIKEFPHGPWASIAQAVGTRSVRQVQTHTQKYYEKIMRRV**RGLR**KDRKTWARVEHRIDDDVLHFCEFMKSMGSNKVMVTSPTAIHRVVSPPSST
> GLWSGEEHDRFLEGLKLYPHGPWKKIAAYVGTRSPRQVQTHAQKYYEKVG**RRLR**GLRKDRKKLVRPEHRLDEDMVQLCQLAKDRKDPSGNVDPLLASRLRAPLS
> ECLGLKKEDKKVFRCTFPGCGREFQLKGNLKRHANIHNGDKKFACKFCGK**RFLR**KADMEVHYRVHTGEKPYRCKYEDCGKCFARRSDLLSHERTHTGRKPFACA
> ALFDAADERKPDSVYVITSYWADCKPRPAGFGVLLRPVTRFWLQETLRLG**RWLR**PDSHLFFQPPDRPSDLQLQYPAGFQDAWQTQTELDDDGDVTMQDPARPAG
> --------------------MLPELQDLTPHCDINKADVGEPGVTTEVQD**RKLR**NILKHHRPIFLGDGNAAPAPACGVVCDLDVGDIKPVDQRPRSVAPHVAIK
> --------------MRDPRETLSIPSDDLSMDQVHAAWLAAGACGDIETM**RHLR**AQHPSWENFHLTTLGASALHTAAWEGSLGIVELVLEAGQDPDTRDDGGLT
> ---------------------------------------MVLDRTRRRRQ**RALR**VIRSPTRQEHQQFLSETPLLQPVIVLQTAVPAEPQDQIVGPSPSPAPPPS
> -------------------------------MNQQEPILRTSMDFRDAFE**RKLR**FVLEFGLCPNLGKNLTPASSAATIDLLLDSDDGFQDLMSDDEEHETLLAQ
> VQREEEERVEMLDKIRKQNVKDKYELGNAATRLDQELKEQQLQWHKKTEV**RRLR**QMPTDEQEVALQAAESSNNNNRKTGSIYAMENQRLAMLMKDSKLRQRVKN
> -------------------------------------------------M**RALR**DLDGNNTAATDVVAASVAVQWLEMAQKVKSMEGEAKDLRTLIQVFRDSLQ
> PTTSSTWNFVQMNMTLLPIARPLPVSVTHRFQEGAALVGVKPCTPTQKLH**RRLR**QNTSNISPRRHDVDCNSVRAATTTSPTSSRGLPPTPRSACGAKRKHSETW
> QPIEEGDFVWVSDESGVCTSAVVVRTGFLFDEESEQEQHVTVVHTEDGEE**RALR**DQPLVKLPACHHPKQLLPLELHDLSNVPLMATNGDAGRTETFTSEQLENV
> ISPPRPSTAPAPNASNTPAAGAPDSIQTLTNGLEDSDNESGDDDVRGHKR**RRLR**RGAAAAPASPSLSQLANGDDADDVDDFIVGDVQQDEQDDDYGEQAEELDE
> AGLVGRFETEHAGYRERNYGPAFQPPATSFGFSLPKPLRPTTADTSPKSS**RYLR**EIDRRTILARLDQGEKQSALAKEFRVTRAAICNLNKHRELVLSRQHEDPL
> YLGKYIEDIQLEGLGLFGGDVVLNDLEIKRHVLRESLEIPSSFDFSRGFI**RELR**IHIPWTQLLSQPIEVKLYTIELILTAKSERGAAADAAIDAAEDKIEQPKS
> DNAQTHKEMLRALSSVRVDRQSAEVLQRLHATEFALLRGEHKDIQVSKRR**RELR**DKWRLKQSDAATLEDVQRRYPKLFEGVRNLYH------------------
> TFMGVGLSVYSPEWMEEGKRLPYVRGIGVIAVHTDNGELMKLIEERKRIK**RELR**KGVGNRGDLQDHGDDIDQEEEDVVEDKGDEDLFDFEDGEATDDGQALVES
> ----------------------------------MSRTAQCKTTGHAPAA**RILR**RVREDATDMALLVTSTPADCWKFVSESSDCRLYELTGESLPHCISTVDTF
> ----------------------------------MSRTAQCKTTGHAPAA**RILR**RVREDATDMALLVTSTPADCWKFVSESSDCRLYELTGESLPHCISTVDTF
> LGLPPLASAVLVLRNNSSLGALPHVISAVSQCVDYSVEIPLARACSFGSL**RLLR**RVWDGSEELAASIGGLSRRRHSCWSPWQFLRSDCHYHRAQFSQGVVEAVR
> ------------MRRARSSSDAGGVFFRVPCALLVEFLDLRGSINLLNAS**RWLR**YDRVLTATALEKTRYAQHFHQKCAADWWHLTPAWKADGRLVESDWDEEEE
> ---------------------------------MARKHARGEEMPAEPKR**RRLR**CDAASHGTFDEVLQVAGMAQRRRRPRRDGSKQSVARVDLAMVTADAVGPA
> ---------------------------------------------MPLLK**RLLR**PDQLDDASATATAEQLFTSSIYAQEMRSQRQQAAALCSAPLVSMSSLVTR
> ---------------------MSAPSKSNSLRLSPMAQLEKAARKLTMYS**RALR**TQLARLREEVAAEKQAVLTSEDDVSESSARLQEIEELMAKLQLDIDALRV
> ALGGFVLDEQGEEDLLREALQTVRDQGFRMQRAVDTGDQPAVLKHAAEVL**RELR**TSLLSPKNYYQLYMQVLDELRHFESYVDEQQQAGASMRVLYERVQSSGNV
> ---------------------------------------MTHGRVSRAVA**RLLR**PFHRHQKEDDASAKRPRGSLTSTTTSSSTRSTSSSSSRSSGVFRSSGGVS
> ----------------------------------------------MLEI**RELR**PLLAILNEDDKPLEQVASMFLHTFTKNDHFKVGCTICILLQDNLLTSSQR
> -----------MSSPLSMLSAELLGSVPADASEQELQDADWAAVSAALKA**RRLR**TQEELTELAAALDPEEPPASEHTLEEHGDVLAIHTELKFLQPQLAVAEQH
> ---------------------------------MNSPPKSAASSSSGLPL**RQLR**WRALPPVDVLDECHWLVTLDARNANRARKARQGEHHKPQLPGETPTKELS
> FISVAGLRVDGRRSEEVRRIRSRFGLFSRVDGSSYLEQGNTKVVAVVYGP**RELR**TAPTTSGAAAVGTGSGNAASNTQPRATVNCEFTQAAFATSERKPQRSGDR
> RQIKVSSRLAPQIYLGHISQPADTSAARPVVVKCAGQLQRQGPWTEWQAA**RQLR**RVAHPNVVRVFDAFVQRSLVFVVMEHCARGDLLSHLVATQPESRLEQHHA
> -------------------------------MATPASGSNQRAPVDDEAA**RKLR**IRAAFDMFDKDKKGCVIQEEVSTIMRYLGAYPTEKDIIKKILPEMQEDEP
> ----------------------------------------MQAFLPAPLL**RSLR**RFAGPDAAEDSEDASSYSILTRGAVVGTQRSVTSSGDDVSVTQQIAVWGL
> ---------------MDAGEGYRGRIPFAPLRLTFEEQERCQELTLNLLD**RTLR**SYDERDATTHGTPRHHSSLDSARWKHHKTQDDASLYSERDHGGRRDLHMP
> SSDSSQKPHNPDSKTAHTSSSATSASTPSTDAETSGKDPNSSDKAGAAMD**RALR**AQEEARRLQFALAQQQRKTAFDMELEALARVCIRVANARTDCEMRQALDK
> ---------------------------------------------MQVLA**RRLR**AAVRLRRVSHLAPSASSPLHHFSSSSDANPSVLDGPEGLLQFANRAMERV
> LSQRDVEAEFGKFGPIEQCAVKKGYAFVHYEQLEDAELAVQEMNDKELGG**RRLR**VAFAVSHGTQRRYDGPPPPAQLAPPGPPPPPPQRAQPPLLHSPRFPVNAS
> ----------------------------------MQTLVEGQTLLRDGRY**RFLR**RIGSGTFAVIMRALDVQQQRHVAIKCVRQRELNALGEREADVLRRANVRD
> RQWLNDNALNFYLQFLTQTSASSDVLLMDAAVVSCLLHQCEDEDEYQDLA**RGLR**LAQRRLCIVPVTDNDALGGDCSHWSLLLFQDGTFRHLDSSAGHNKRAAQR
> AVRYREGDEVKSPFGPGRVRSCGDRKTHVEVALAGGGVLYARRGCLLPEL**RMLR**CGEFPRDTKVMILSQEAVEGVVLDYIVSDQQYQVLIEDGDEDHHSELLPT
> --------------------------------------MESLSSTASADL**RVLR**SPQLSPRPHQVMFQAVHQLALDVGAALVQQLAAQPPARRGVGIFRRHSSA
> -----------------------------------------METRLSLRR**RLLR**IEDTDASVASASASSALVFNGSNQSRSSAGRRQQPQEFDDEDDEGDGEGD
> TLQSHFDGGGAPTPRFDVQQEYPLFVTWEIEEKGGTHLRGCIGTLAPTNL**RNLR**DFTFKSALRDHRFDPIGPQELHRLHCSVSLLVDYQDADSYDDWEIGVHGI
> ---MLALQRHAARSGAQQVARFSAAATQEAASNVLQLQVAVREGEGSRAS**RRLR**KQGFLPGVLYGEGEDGSDERVLVSLETRTFERVHRKLWTSVENQVFQVQV
> -----------------MSSSSASLARGALAVLRLGRNSSASLCSRGNNV**RLLR**RAPERHGGVELAREMVSTTHAKKLVWRDVDSEESYDLPAVKQLDKQDEEL
> SDAQSIPLSPSRLESPMFRERVNALMQQGDTYAKRLDGERRRSHDLDLAL**RTLR**VEHFQTRKALSDATNAQLCAATTELKPLRTLENRLDKVLTRYNEVCNTNK
> ------------------------------------MASSTQSDKTKRHW**RVLR**NALLRSTSSGPPSSPADATGSATASSIATEFFPLYLARPVDTAQVQIRSP
> ELWDGHLIPFLSLDEAVALAELNRFFYDIVHKQIQIQAEASLVCTIPELL**RVLR**KWHNLRNVAFQPVGDGPEIHESIHYNVNAAGGLIVDHQDNQPAPEPEPAQ
> ---------------------------MARTIDPSESMTPGSPSLAQQKQ**RWLR**AREEVRQAQIVTVKQGLSLTPSLYFGTRRCTYSTVVSVVTTALPLPRFDP
> ----------------------------------------------MASK**RVLR**ACKLQGLSLHADALRRLTDELTNESSLQLEDVIYAIKNNIDRSKMTTSVV
> AAVLRPSQLVFIQSYQHETIEKAKSGELSLKRLAGYTLAEAYRSQYRASE**RHLR**ALLAEACTELPSFPEIFTHVLRAKAPALVASFSGARVIALRLSCVVLDAA
> ------------------YLYRRLETQMLPSPKDHEVIDVASNGPALIEG**RGLR**SLTLDGADALGHYDDGNGASQASEIGEEPLEDEDDAQATEGEEHAYRRGR
> AFHQLLRRFSRYYYILRHNARSGSATVLLRRIDGAEHALLGVRLPPSTLS**RYLR**QEEEVLSNALEGFAPARISWSSPARQVSLEKVVEAHEPLLKHTFGFIDGK
> AFHQLLRRFSRYYYILRHNARSGSATVLLRRIDGAEHALLGVRLPSSTLS**RYLR**QEEEVLSNALEGFAPARISWSSPARQVSLEKVVEAHEPLLKHTFGFIDGK
> -------------------MASALASCKSVMASYRCLGEGLQKETSLNVE**RHLR**VLVTLIKPRSDTAITCTRAYIEVSERSDWSQLHSQVLCDKLRHSAAMVVP
> ------------------------------------MSTPAVSTQPAVLR**RFLR**AQSVSSICQDLMPRLMQQLRRLGRPEEGNNASSVPLEHVTAFLDAILLAH
> ------------MIGDFEYVQQRVALQVDVMRRHVTGVAEVVLAPRAAEL**RVLR**LHARQLRVRTVHIDGVQANFEQLNFLGEVVDESYRDLATFDLFYRGAIVA
> ---------------------------MAAGTRSRRAVDLLPSEAQASES**RTLR**HMSFVEMLSAVSPRSSSSSSSWVAPPAAAWTREEINMIQEMFLSDEKAIQ
> VGPERPPSLTKKQHPNRQKKTSKIRALSTERSRQCRERQKLYSENLEAVV**RALR**TEVYDLKILRSLRQEQSLQLRGSVSGSLAKIIREYMTVFRHGMTAETVEA
> ----------------------------------MLTTENLPPQVAQRVA**RELR**KLVIQPLDGICYLPQDEEQLSEIHAEIRGPEDTPYEGGYFTVKLTLTESF
> ----------------MSHRFPIQEAVFPKLQLNESAKDKLINTALMQLT**RALR**DYDKYQKWQASRPPSLRHQLHPAMWKPVKTMEQLTVYRRVPADDAVVTAP
> ---------MAPGGRAWRKENDAGNRRLSVTSSEDVPVSGAKVPFEELLA**RELR**KSEGNGAKSTVTATGKVSKKPFLKRGARGWWMQRPGAKQKVVKHKLVSKV
> YARAEAAKHTLEAEMEALAAELTSGNNPGLRGPLVDSEGFPRADIDVHRV**RQLR**HDLAVKRTDLGDLMKEIETLLPLVFEVKSGQKQEETARNVGNAETAMQQL

> -------MELAYEGRSEPLEFSPPEQRQRVLLLRNASDVAVLFKVQCTAP**RKLR**VRPALGILAAAETASIQVHLSPQECTAATCKLLVLGRIATQTLPLRAAAV
> LPGFQRFPLNSDAPVIIPRNSAVVPTPTVSSACAPANASGDWRLSGNDEM**RHLR**SGTVVLIERMLRNHQPHDVLNHVPENTDWRSKTFQLALKIERLMFKAAKS
> AQASFTHSQPDTMSVTVDTPHNDVRYFDSTWLQIGQDLRTQRWKNVVNID**RMLR**NHRPHEVLTHVEQNTNWYAKTFTLALKVERLIFESAVSRESYLDEKTLRG
> VVAATGLQNTLPVLSGTPSAGCAATPTIPSVVGVVEVPTGGTWRQAGDEM**RRLR**SRHVILVERMLRNHQPHDVLTQVPENYDWHSKTFTLAIKIERLMFTTATS
> ---MAPVVRSTTASGSAIYESERAVQEYLQFHYGSAETRTRNGHWLRGWP**RYLR**AEQAVGIDFSQHFVNAAKQMKERGEMQYEAHIQGDVHEVRRAKLPEGAKP
> MTIQQPWVGVKNAVQQGRAVPRSVPALYRGLGVSLSSIAPVSAIRFAING**RLLR**VLSGTDSAPTDQVKVLCSTVSGATAAVITGPAELVMTLQQNNGRSFGATV
> -MRDQGKTARTHGGAGSGFLTRPRGMWEADYRFAVAARLNQLDTHSVLKR**RRLR**AHDQCRQPGCSRAETLAHVLNHCAGTMDAVRGRHDDALKHIERALHASSP
> -------------------------------------------------M**RALR**NQVQSELARVGSFSLHHIDRQDNAHADRLANRALDLRRTVIECGIHCDGV
> ------------------------------------------MNAAPREI**RWLR**RIMTELGDAAVRALTHVEADEDDDSDLDRLLRRAMLLLRSRAINDQLSGA
> RRAVVTRVDGGADVDFPITVDTEEVIPPDMMLKRVAHRFSIPLKAGGSKW**RKLR**TFDLVTGTFSAETRASALNKALEGAVAAAFDAVRDGYRDASEEIVPETPQ
> -----------------------------------MDGDQRGDSAADLFS**RVLR**GTEQSSTSGASSVNSSAAVGGFFVCLDVPTATEFGVDYEVFRTGPKFQGV
> ---------------------------------------MAARTDLEARL**RALR**EGKRPPAAAPAPPAAPAPAASFSWPDVPSSPVQKQVSVRQRVPVGGEYQP
> ------------------------------------------------MG**RTLR**RPRRQLGLEAPAKEHLAQRIADVVALSLQQHGRESGTGGLPTGKQGLLAS
> IFSEAGSVVNFRLVTDRETGKPKGYGFCEYADGATALSAMRNLNGYEING**RNLR**VDFADGGDKSGGADRKRHDNGNQRHGGNSGGNFRNGGSEGGPPSMVTGEM
> RKLAAKKKKNLTPKSPLRCGCCGGKEDKEGKCGCTIEPRVNSKHAADIYE**RQLR**WKNANETRHSQQRQLKETLALTECTFRNPFYQKAWNSLDDRVSRSTEDGG
> CVTKGKGVKVVARVTHQSAKPTPASPRSAGGRAEAVKYATTNTTNLARVT**RLLR**FVMQEVLGPMKALPTPNTPLSPTLKAADAPASQADKKKSKKKKKAAKK--
> -------MERSTSPARTAAGLSPLEQQKQRALLAKIAQFETRGAILTTDA**RTLR**SQLHDSASGQWQAVEVRLQQLARNWNPYQDATSAAKLEKKKLDFYQLLE
> MAAIVEDPPLELTQRTRAMDRELKPWALYDLSGAKSPGSLEEMTGYFRRF**RALR**GKSLTGVSHDALQRSWCAMIVRWNRMHQEGSNFVAWLTSREEVVSEHSLS
> EKMDQEAKIHDRDFKRAALEHKHQEDALQARNDELQRNVEDLEDHVRALD**RRLR**AGSLNVPRVMNFLNRGKTRVAGNWGRLKGLLELAKDNQPVPDAWATQILV
> PKVSEVFKHKVFALLDDAANDKMDIPQPVHTLVGKLAGRLLGVFNANKRA**RVLR**QEKGDVQKLKVAIAAVCGQETALKVEQFMLRLVDAQVEADEAKRLNKTKN
> ------------------------MVRIRASRRTAPAAAATHDVDFPHLW**RQLR**AAGWTAKRPSGLANDWTYTSPDGSSHFIGEAAVVAHALTSGLLNENEQDD
> ------------------------MVRIRASRRTAPAAAATHDVDFPHLW**RQLR**AAGWTAKRPSGLANDWTYTSPDGSSHFIGEAAVLAHALTSGLFNENAQDD
> ------------------------------------------MYSREKLE**RLLR**QAKVTTQSIQKVSQWMLNHRQHLPEMVGVWRDLLFEGEAEHQIVYLYVAN
> ERKAVLVMNKIVEMGGLAPGGLNFDAKVRRESTDLEDLFIAHVGSMDCFA**RGLR**QAAKLLDKNELHQLRQQRYASWKSALGERIEDGKATLQELAAYAQEAGEP
> RSRSRQLLRSMFIQTEPTLNPLSVKFFPGHAVLDDRFTTGVSLCSRGNNV**RLLR**RGPEQHGGVELAREMVSTTHAKKLVWRDVDSEEGYDLPAVKQLDKQDEEL
> -----------------------------------------------MQR**RELR**PSQELLSLLDYLNDVVGLSARYVFQQQLTPPQLLQTPQSELRRVFPELCG
> -------------------------MPRARPDRYWKYSHSPVGLSRLLQE**RELR**GNARVIDAACQGHGSLVRRLQCEAVLAGHEGCVNTLQWNESGKLLASGSD
> ---------------------------------MVRKKVLQDGIVSDKCF**RLLR**LKHEIQSDAKVLKVVVDEVDVLRLCRSLGMRLSYEGTPVAKDSFDFGLFN
> AELQKMMESLSWVHEGVGSSRGKSRRRAHNDRHDEVEFLRIKVQQMEEEL**RALR**LQHLDTMVPMSVSPGPAATLAFSSQAFGFPVSSPQTPETPPIWDLAARQQ
> ---------------------------MKYLEEPQLSWLNSVLGSYEVGD**RVLR**GKLETYSCKKAGSDKRLAKSLELYYQQEMEAAADLLGSSPLGPLAAPATR
> -----------MAADVSARVHLVAEKLQQKAQDAQRKGNENAARALSASV**RDLR**EAMALISEQRHLLARRRGEGDDEEDDADAHVQELATRLTRVDSMLGKKAD
> ----------------------------------------MSIQLEEDLR**RVLR**LARAEAKANKLKGSGKGSNISKVHQPEHRHRHDQYLTGSKGERPTLLTAQ
> GEEQEKREASSKKRKLEDSTAAQQEQETTAGEEELQEEEAESLLSWPDMR**RRLR**ELAEPITLFGEDMKARMVRLRRAEIDAATRHEDDLGAGHDIRNRFVGGDG
> -----------------------------------------------MWK**RPLR**DVVVRCHVGNLRVSGVMARNAVDEVARRWRLPLSSEGTKLWGEYMAGTTM
> -----------------------------------------------MLA**RALR**SSALSRAASRRFIGGFAADASAPTVAEKVSHILVVVSAFGCSVNVVLVDY
> -------MATMALAVQQQQQRAEMASYLYEKHLTHQDGVAGTLSIRLVAA**RNLR**AAASMFWVRTCNPYVIFRVGKQSVRSSTIPGNDNPTWRREFLEVKLPKLP
> LTEIHVELQLLDRNGALQDNATLRRNATVKKFALAVNHFSSYLQKYNDTN**RFLR**FFKRGEMEEERQQIVTEIDQLFRMLGLATSVTVISSNASAAKNAAKFLSK
> ----------------------------------------MLNGLSEPVE**RSLR**SLFYSMNDDFAAFGIDTEEQRSDDENEGSPVHDAPPTTNDSFLSRLDPVF
> VVQLVIRPKRALYDVHELGPERFQVAELSVDPTQDDVTQAKRKDFDVFNE**RGLR**VRCSHWQLFAQGSNSPTATPCLVYLHSNIGSRLDALRVRDAALKRGFSVL
> ILTDGMLWDARIPTRALYKFDKLSNVGCGFFHPNGNEVIVNSAVWDLRTY**RLLR**MVPALDKCCIKFSPSGSVLFAYYPYEGGDYLPIRKSKLKSWFRVLDARDY
> -----------------------------MERARIRRRRQQFQTQLEVQT**RELR**ELLTVQLTYEAKGASTQTRQVLKAQYQLGELCAEFDEFVLASMYFQQVLT
> ---------------------------------------MTNSSAMRRRM**RPLR**ATPEALTAQATQHVPQLVVRPTNRGGETLQVAMGGTAVERSMVNVGSIAV
> DPPPLLRKLYDLGVPVEIQRLARFANMSPDEAARIVQHAYRCYRGREFMR**RILR**SRIKKLWDATSGRYYYYNITTKESSWEKPRLLLNDDDDDDNQSPKAVSHE
> DRVRRGLCSESKEAQKHVGQLFGGPRSGGVSCLAGSSLMKVLFDDLRSTD**RTLR**IAAARVLCLMAYENLTNQQLIIRSQAGGDEEQLGVTVGWVHAFAAPQSLR
> QEMKKEQKPFDGSASAVSTCPTGSNRSVEPVLDSESFSRSLSSSCLSFAS**RRLR**IRRLHARVPLVSVTSLYNRLQTSGVVLVDCRKGVEFSAGHLPDALHCPHV
> -MNSVLSSLTWGTWDDAEWTPAASNIDAANNFIPVAVSEVGKPVNASQNQ**RSLR**KLRKKGLQAPKNSQLWFEELLERVEGTSFGQAKATYRCNPLRCLHASEES
> AAGKRVGGKDAAGKLPVTATELNMESDKITRTSQIDTNPPVSTTPMASSG**RVLR**PRVQVKRDVNFVADNERSSSGKSDGCDVVDEDEYESPDRNGANSDEEVLS
> ------------------------------------MDMRSKAYPALPEG**RGLR**LVLPRVGDLRFRPQVPAVFAQKLYIHADPRRRFWYARFQLKRKFIIMSTQ
> SHESDNGVTEPLLPAHSKGRSNSLSSSANGVYDRNTAEHVERGDVHVKRN**RPLR**DSYLKQLGYTPLIFKVNSFLWRKMKKIGSFIASMSPMRARKPNDDLLSDA
> --------------------------MAEKRRKSTCTKRKEEIGDLKAEI**RKLR**SELEVKAELKGLEAPVKRGKSPPFESEVLENETLASVIQQQQLEIARVHS
> SSSPLYNSSRDARDLSSDTSPPLRPLGASGRPQFAAAPAFPRPPFPQDER**RVLR**TQISPRTTHDAAAAADNLLSLIQRNRPGRPPAREVAGRVPHVLRPRDEYD
> ----------------------------------------MGAGERLAMP**RFLR**ESVRERAPDVVPSSSSSSSASPSPASPSAGDAESSMVEELQRRKAAFLAH
> -------MVLKKLLGTAQDMWRMRRMPTQVRAKDTRLAFTDIIGDESQKG**RWLR**TGPILELMDVLAGTISSRVSLGPTATISFDRVDLVKPVFHGDLVRVEGEV
> ----------------------------------MDHSWRSASLQQKFCD**RALR**LSDTSDGDLGVIKDTVVVTHSMGGLVMSMALATGKCSFGEGASWVALSSP
> ------------------------MSSSNWGAWGTKLNVTSIVSQGLEQV**RSLR**EDVEKSFDQVVTGAPGSRPLPPSLGDQVEYTAGRNEANKTSNEAVEPDLN
> --------------------------------------------MEAPVK**RHLR**GATSRRNLLEGPPSPSLTPRSIATGTVRGQSQRKLQPAASRDNAANPDES
> ------------------------MQAPTHDDPTDLWACARTPETAPALA**RLLR**DAAPPDDRNDYGETALHVAAAHGNEEAVQLLLHSGADLLAADWTRFGKKF
> ------------------------------MLRHVLAASPASVRLQGQHL**RRLR**GHSFSSVPVLQRGDGCKALQVGETYHNFTLERVATVPEYGVDALELRHEP
> ----------------MASSSAALQQSLGESEVFDLVCDYLRTRQFFEAE**RTLR**SEREATGSPRASSPVAANQQDMAPVEDAALPSLASRLEFMLERSYVTQLV
> -----------------MVVPPKLPISEMAVARTPQVMRLASMTYDEHSP**RFLR**HLQELDHPSKKNWTTVERICFEMTGTEKDYVSDVAALVDRFLDPLAAFAE
> HGSIRPSTLQEILQDEAMLKGSDPLQLPKNRSLDECFEMFGLIHQVVVSR**RVLR**RIVIEAVEDFAAENVKYLELRSTPRDLLRDGATRADYVDEVVSALEECRA
> --MGAPSRRRQQHAALVARLAAVKQQQQTLREESQAFAKAPNAPEKAAAQ**RALR**SLRQLTPEVTVLCAQTGAVVERVANANDVAEKMTREVRRLDVIQSRLGVA
> ---------MAPTGAAVAAATAASVAASMTRVVLAPAPPGALACQRDSSL**RSLR**TAIHACHERVVPATKKSKAQRFYELELEDTVLFPEGGGQPWDTGNIDGVP
> -------------------------------MPSDSDDEGRAQLELAAQM**RALR**GKLRVREQEVVALQDQVVAVTTQLRETTDELDCRMSFVADAMTQIGDLEA
> SSISRTHALIRVGALTPQQLMDPSTRPALELVDQKSRFGSFVNQEQIFGT**RRLR**HGDQISFGAKKTVLRVRYQVFVLVASRIQRASREQLNDACQRIGMHLIVA
> ---MQRVAVLSHVKASSVPPSTRSSSVRRIAWHGGKMSSNKEEALQKFYQ**RKLR**AGDHLTAQQIEAMQTTLKLDLQTNPKKKHATHRPHAKVVATGSRTVLKTG
> DHKVMVTSGPQSDRLAFGEYIQKNMKLYELRNGVTLNGPATANYVRGELA**RFLR**RAPYQVNLLIGAVDASPEQKAEPSLHWIDYLGAMVKVNFGAHGYGAHFCL
> -------------------MGNSPLSREFSSRRVTSEEREAMKRFGDEEM**RLLR**ETFKGLASGKDGVSVDKDTFLKCFPMRGLLGERLFEVIDKDGSGSIHYNE
> CIFNADKTAVYYDDTPTRIISERGANNGAKIKGQTRSGRASVLLIVSATG**RKLR**PIIIFKGQPGGRVEEEVKGISNKVVTAVQKNA------------------
> RQHAANRQSGSPPMTPTYTVPRQLKVPIIDAEPETTIGAAVKMKGELSFE**RLLR**IEGEFEGKLNSKGSLVIGTRGALLGNVENMKEVYVTGGKIVGNVNVEKLV
> ADKQPEPGLVRKIDKSTMLLQNALTSEDGCADMVTLNYLHEPAILFNLKH**RFLR**QIPYTYTGAICIAVNPYSWLDIYTKELQEQYLERDRSELPPHVYATSAGA
> -----MDFEALFPPLAPQDEATAQELSSVDINVDDILLMPLEKAVDDGIY**RALR**RWNEGDSALTDKALPYAERVKKYQRIAALALQPDGELNTAEEEREEQEDA
> FHLIMEFVDGVPGSIVTLVTDFQRLHQGVPWSATGALPRAAIQHGDLETL**RGLR**KLATTKTFQLRPELAMDGAARCAIQFGQLEILQYLAHTGLLVENVTSTTV
> ERELQAAQAQLQFSRRDARTLLEFELQQAEDVLRTDREQLKHKLLERVRR**RRLR**VQKRLTEKPPTSNIKQRREVEVAEKKARLEAKMLEKQLQRAQRRTRKSFN
> -----MGEASAASPPEPTSPRGSPVVTTDSAMRLAPSSSPALTSSGGRSG**RNLR**QKVVDELKSVRVRKSPATREKPTRAAVPPPLELNEDVEDEEDAAQGKAKG
> DVWQLKRDSCAAVRAFESQRSAQIDRFQARDRGYWRQFQDEIRRGGDENA**RLLR**FFALRLHADLAYAEALRQTRSALDAPTAQSAAADSTGGNDQLNVQSSVSK
> --MAKGAKAEGAPAPNSEVLSVPILETVKAAQLQNGLRHRDFQRYRQYCT**RRLR**RIRKSVKFTHGKGKQFVNKKVDAETASEARLLYLPLYNAERAWGYAMQLK
> ADRLVKEEWTWARIFNTDAAGSWDSIIFSDEKKWNLDGPDGFQTYWWDIH**RPLR**QTKRRQAGSGSVVVWAGFSAAGKTKLAVLYVKQNSDDYVYTMSEYLLPFA
> ---MACRTPRELAAACMDHIALEGRAGLSVRRLFAAVEPHDDTHVRRHVW**RLLR**SSRGALRFFRGSEASATPPQTPSKKRKRPGPCDNQDNSVEVKREMLQAAA
> ---------------------------MHLDLVTSSVTRVTRADPTAMAA**RELR**PLYKKLLRLAQSLPEPKRQQSLDQIRREFRSHEELTDPKEVSALIQRAQS
> RKVRLSDRELFRRVQRVAVPNGPVDLTGLSPKRGWKRVRGGLTGAMEVQY**RVLR**APTTVAPFLTRQCQVVVGGEIRASVSELLSLLRAPTESESNALLRALYGT
> SSVVTALFGSYDLRNAKQWRDEDMAHREQEIQWLNDDIVRAHQWRVADLE**RFLR**REKLQSEHVVCQARAEQLSTVSEQSTLLCGFIVSAMCNVGVPDDVRASVL
> --------------------MSRVAAMKQPEPVAQDPADGTPVKEVAPFL**RSLR**RMLDTESPRILCWTPDGKAFEIHDMAAMTGYVLPKYFKHRKYASFQRQLN
> -------------------------MSKEQARVLQLSATRTTSSSILAFR**RRLR**SLWASLGESVDVARERESRWEKQRSKQIEIFVSEAEKSYLLLEELQSEEE
> -------------------------------------MTHAQKYRQKIER**RKLR**DRRDSIDSALSESSSQATDSHTREPSMVEPPFDPTEHAVLLEFLDNFHPS
> SIPELDRKDLLIVKDGTVRVAESLDREQQQLDANGEVVKASGTWTKIEHE**RFLR**AMETFPKGPWKAIAEMVGTRTVRQTQTHAQKYREKMARRMRGLRNRNGTL
> ---------------------------------------------MTRKE**RLLR**AKLKKKMADDDKSPSSGANSFEDILQMSDLSELSLLENLRKRYEHALIYT
> SVLSAMTVRHDDDHLVTQRPAATLKEILEKIRANKDQIRELDLKDMAVKK**RKLR**PSGGDLVGRVFQLNRTVLRLLLPDHEIGDAGASSMGNMLRANNTLQHLDL
> WGYPWVQPENTYTVAQAEDLFWRWVGLRGYSAQELDELKEDRLLHRILDQ**RDLR**IRFAHLVSKRKLYSEMEGLKRRAQSSARNERGYGVHASATVPRPSAKKPR
> ------------------------------MPHTQSTARRWDPWTSSQKH**RRLR**RRRPETLGLLTPDRIGDHLVARCMALDDFRVRNLTNGWLPEALAGPRTNF
> LFNSMAAPLHPETYETLGQAWIWPTKQQQVDASTSVAARTASLKRRSGAQ**RALR**KSCSERPLGSPKDLLATGGSVSVLDVDDSAADEDDEQRRQRIMARAEEIL
> ---------MSSVVCPRACPSTPTLLSGCSASDVSLSSSSVFLASASRRK**RRLR**PEMDAESCSDVTEVDPASLSGLGIQAAALGNSTLTVLNLTGNSVHDVQDL
> -------------------MEQRRLAAAESLLAHSLFGFQVHDTAVSERL**RDLR**ELLLATCGERDQIALLSESIREIEEQLQRHCERYLLEPLRRFEQEMELLL
> ---------------------------MFQPLRGQAAFTDATDAICIGSG**RFLR**SVLVPTLRAAGSNVVVAQTRGTSFASACAKAEGKYEVDTIQKDGRVQTEV
> TTLATVAQLLRSNGFNLVRVPLAVAAVAHNIVVDRYKIGNEVALLKVFEG**RELR**YLDVLDYVVHELERFGLLVLLDAHVMQPAGAITPLWYDETQGCPETVVVQ
> -------------MFEKVVESVLEEYVSEWVEGLDSEKMKVALFAGKVEF**RDLR**MRGAALDKFQLPMKMKSGTVGRLSIKVPWKRLTSQAVKIKIEDVFLVVEP
> SATSATSPSSQWTRSIQNQSFEPKPTAYSPHSLEDEDPLMLQQRQCRDKL**RALR**QVRERMQFYPLQPPDKRRPASYSMKISTCTILSERSLHWQSDGPQVSRHN
> ---------------------------MSSLNLEALWPSPPARALPRRRR**RSLR**STLARAVFPRVMEFKVISRESDKHELMPVVYLIDRSPAWRRRVPSLVERE
> ------------------------MEVLQHAAVGAVVAGGGLTAAQSLLS**RRLR**PPTSLALSLGSFVGVFRLLEATGYKRSTRNGHRSPNASQAAAVAAAVALM
> ALRKCRNLSRLTMVNCNLMEISSLESVGASLVHLCLSNQNITQMSGLSAL**RQLR**HLYLQQNRISRIEGLERCRKLKTLWLYDNYLTSVDNLGFCSDLRELWLQN
> --------------------MLDAVSLSEEELTVLFSPASHGASNSMLDH**RQLR**AGRKPSSSQARKKKARGSKASSTKTPSTTVVPARCGYRTGKCQNLQAIKR
> -----------------------------------------MSLSLLQLR**RSLR**GPTTQLTRRWINYGHDMHKEMLNGTAKVSVTEYDEGGFVINDVNMRGGVA
> PAPLKWKDVITAMVAAVGKQSEYEDGVTTPKARLFLLPAEAGGITGELNA**RALR**FGVRENDRFVICLQGEDYQPPSERQTAASTSTAVAVTGGSSARPMTLSTP
> ------------------------------------------------ME**RILR**GGSSSEPRHSEGNALKLPQNVEDTLAKVLPSEDVLDRPEFDAREFINRNF
> ERKATKAYGVSRGPLHQRINGRVPLEARTGTQLDYTTEGADRGVVEMVRY**RALR**RMCVEFEELRGMLQLLDIDRASASTADVVSHYFNNPQRVMKKLDLSDKPN
> FSAQLQCAICLCAYDNPVSLPCNHCFCEECIHRALELKTLCPICKAPAKK**RRLR**YDTTVQELLCATEMLCAAPEDAAAEKQPQSIEMKTTKQTTEKQVTTAPSD
> ------MGDSQNWSSFEPEAPKPAKQPQTTESDDIKSSKGLGALTEPVAV**RNLR**NRLIFGSLVGFVTGATFGGIDAAKTYHKTHGKLAPAALSSVARGAAVTGG
> --------------------------------MRSFLSRCYANSTKRNAV**RHLR**VVEAMQQTNEAALTKAKEQSEARAQQETGALSAAEEQLSGGASFQALQTL
> --------------------------------MRSFLSRCYANSTKRNAV**RHLR**VVEAMQQTNEAALTKAKEQSEARAQQETGALSAAEEQLSAGASFQALQTL
> SYIQLQRENEELRENGRRLQQETELLAAQSKQNKLRDPEDTRCEELEQEI**RLLR**SEKMRTEEAHRLELQKTEARAAHSGATYQQLVAKYQERFEFDPLEAKRAA
> PNAASEPLESLLVQLPEPNARYSDTQGQPTQLMRFGESARSLFMLEKPSE**RRLR**LVIALKQAPRAVDTWLELLRCPLAGNPGKYNSLEAREGAGYTEMCIMLAK
> --------------------------MELGAAAHSTAMCWAAAEGNLEAM**RRLR**EEHGADVNAADYDKRTPLHIAVSDEQLEMVDYLLQCGAYAEALDRWGRSP
> ---------------------------------------------MLSAT**RVLR**RASAVPRSLSACFSSSSYSDNQWRSSPRSRGPNPSRGGRWRDNERRPPLT
> QAAPDPTAAPSAAVIARHRALKEELLHLDQNAWLTKHAKQPRFQFSGEQK**RMLR**QWFDALDSDGSGKISVEELEDPMLSIGIVNDTREIEQIVSKLDKDSNGQI

> ------------------------------------MLARATRVEQTLWR**RVLR**AVRPLAENSTVGCSQQTAAVHTALFSGRNAVSGTQKRRVNVSAFAAAGGA
> ------------------------MSALLLGTEPVTEVKLHPVVVLQALD**RSLR**RAEGQERVIGTLLGRVENGVAEISGSFAVPHLEKGDEVAVGRDFHAHMYE
> ARRVLLRNFSVAPHAADKFVVEFPQEHEGLNIEFNWSLADDDVTPHGDAF**RNLR**WPKLEELAKHDKLAGKKVTIEEVDVSIVFNDFEGLYEKVTEHLSTEPNLY
> ----MAAPSFYEVLGVAATCSADDVRRAYHQAARRCHPDKRSAATEEDEQ**RFLR**VQEAYETLRTEEQRRQYDAKLLQDELVRRREQELVMVSDEVPLADMKREI
> ------------------------------------------------MQ**RLLR**RDKGAPVVCEGDEEDGEDEVSITTDDLPLELDLAAVEQQLAPSPAPVALE
> HNIERMQALATSHSVNLRPHAKTHKTREIAVKQLAAGASGLTVSKPSEAL**RFLR**GEVLGLKSLLLAYPVVQSNKVQEVLKAAQEVGVEFLLTVDSAEGVDAAQK
> -------------------------------MVTRAQKLANREEIAQNLQ**RRLR**EIASKSAAPPSPPASKKRKTDGVPLLLTPHLSKPRTPVISAPKIPAKVGE
> PRGLAANPNSFCAATSDNVVVPDAITKERIVCTTFVPLMEEDAGRLDALV**RKLR**HPMAKIRARALQSLLFKLRERLVRWPDLVPLQNALVPSLLACLEPPLELP
> -------------------------------------------MVDLRDS**RFLR**DVAPPLTAPKRAESPEGTTSSSDDEDSSNNNNHSDSGRPPRSKLNADEVF
> LSTELNSSKYAIDAHNRSIGELGNLSELPKLCSLDVSFNKITSLRNVSTA**RELR**ELKIYNNKLTNTIGLKANSSLEGLQMNDNQIEQISGDFLALGRLKNLWLN
> NVSDNVETEVLQLSESSRAAQEKHAQLLRRIEAEKRARNISVPTSVEEVV**RRLR**QLGEPITLFGERPADRRERLREILSRLELEAEETGFVHQVLADVQKQGPG
> -------------------MTGESSPSAPPPSAPAHPPSSACRPKRQTFS**RFLR**RSPYGERRSRAVAPADSATHAHSGSEGHTRKRRGAGANDLTKRRESGIAH
> ----------------------------------------MACTETPGSW**RVLR**DQAELVKETPVSKEIENVTEQVDKVKLAWGSDSDDSDWSDDEDDSKTQSQ
> ----------------------------------MRRTRSSALAEAPQRL**RSLR**STARNPPLSPPPVASLRRSRRQPTVTKTLIKSEEQDAGQDDELDEDEEQD
> -------------------------------------------MLHVSQF**RALR**RSAPAPVRLLARALSSSGVSQDELLRALDKFQKESASKTVPWFLENMPPA
> -------------MRGNYDCWYRANSPEHYDKLLRGERPLVELTMRQLCL**RSLR**LSSDGSGIPVMRALFAGVYQNPGDMLSFRTLVAKSLSTVIASGSGLSVGR
> ------------------MAFVASSPAASGPVDAPSTPLSQDELRVRQLL**RDLR**VDTGDVVLFDRKCSSMGLYGGAICVCAKFFGQTQWDHNGVVVRAPSLALD
> PERDGGGGMLSGSPTPTREHSTLLREQEAERERLRMDNFNQALRINFLEE**RLLR**VKQGTDFTSEDLENELAQLRIALEEREHELQQRNFSMIRATEAIDMLNAQ
> ---------------MSTPLKALIQERLRAKMVRQGPDYDLDDEYACDSD**RLLR**QPVEVTAYDISSRGDDEDVLVSSSSRGPITRRSNPTQPTYPVRLHTHIDP
> VGAYWSRALVVPSRCRQVSRLWLEKALVLTDGVQLFALMWQLSQPWPWPA**RWLR**ATRWTNAFTLDLFSFRATGAAMGSTSQSFSLWGEMPHYWLYALLWALVPW
> ARSSSSRGAIAHIHGSSINSLRPPGPFNYYWVDDHINVAADIGSNCADIE**RSLR**GAMLTVLGHGAINEAKFTPWCPRQKALGLIFDSQAGTVAMPASKIRVAQT
> WYSSAPLDFGATAVAEIEDAEDVVLEPLPLQQGFVDQKPIEDFNLSQESQ**RNLR**KAGVTHLFPVQTQSFDVMMKGSDIMGRSKTGSGKTLAFALPIIETIQANR
> -------------------------------------MKPRADWFTTSGA**RELR**DSSALDYREYVHRSEDCQSPQFEEDLRQIELDLPRTGESIRLFLLSPEET
> --------------------------------MQCLQRSSRIGITGRLAP**RWLR**AASTSSASSCYTQELPTEPSAAGRGPARVCPIQTPYKPHMTLYTNLLDRA
> LNVAGGDARASAAHERATEEAQDADNAALYEQALRCQQKQQISEAKEIYQ**RLLR**GPSINPRLEYLCNKNLAVMQLEGRSFHEALEFFAVALALDATDVVVWCQM
> VAHFQALQASASAAVVADVFEWLGDAVIGELVGRCLLSQFHCAPLSARVF**RNLR**LAVVANRNLAQAYDAMGYAALRRRSFREDGGVNAGNRTVKMKQRADVVEA
> EEDQVELQKLAEGFVHEAFEEYTDFRCVAGGVLDKERWKAVKTRDGVTSY**RDLR**MVDPERSRVAPKEKVGSSQMTASTKLHGVLAVGTVPGEFNDMAFGLVNAT
> ---------------------MWYCFGRSSDLGYIRKQHVSVSADSTFYL**RLLR**VKTTEEQGLTLVPDKDDFLTCLLHALAAALATQDSPCAALLGQLPELVSE
> ------------------------------MEALVLKSLGKLRKACTRSH**RELR**DMIDAAQAKVTAAGQDGAAEVPFEPFLCACLTRHAKLVAVALDCMEKFLA
> SDVAPASNWVDLRPLSSSSRCVSQSGPRPGLYTARHRLTQELVALKVLDR**RLLR**QRATRRRLRQEVRVLQLSRDHQNLLTLYEVKAVGDRVELAFELARGGDVL
> LNSENEAARFDEALRGYFRDAPPTRKRARGGGTANQPAEIDTEMCLKQLK**RLLR**DAAVPTQSQYIRRLHAMILHQLKRGTKSPTVFAKVSRLHDKYQVLQWFTG
> TAAQELSDAFQAAVTDDEQERNNQLLVHACNCDDAQCRAPEFRALCSHMK**RFLR**ASCWASHSHKWRAYPIATAVVELFTYHALHCQALQCNVPMCRQLRGQYVR
> ---------------------------MHRFAKSIPTCLAVSSRASASSR**RLLR**PQSIGVAFFSSSEPQMGKPLTAEELAERRAQRAARRAATGKEDAKEEKSD
> -----------MAATFVPTPSLSAGEPSPSSSSTGGDARAAPISASRFAQ**RRLR**VVTEPKHSNKMHRTRTLSELILLSPRSSPRITPRHKRFDMIEVSSFFAE
> -----------------------------MSAHTAATASVNGFQLEESNK**RFLR**SYGMSDLDVKEDSSEEERGIDLTKLDDVINVVKGDKVIDAAKVDDVINAA
> SFNHVSAHTSMEDATLALHSLDASLYQIAYNYGATGNGKVYRCVSHCDCP**RRLR**IVRASKKEDAKTATLYNLETTGDHGTKISNKKRKGIDLSVKGDVDALLAG
> PRSIRRAYIRSVWFPIDIASSIPFEYFLKDSAASMSTRKSFKMIKYIKLP**RLLR**LGRFVKYLRRYKRYSSVIITLNAVIFSAHVAGCLWVAILKPCADAVEATR
> VSINPLRERELNLRGCKAPAIENLGVTQDGFDCIDLSDNEIKKLENFPRL**RRLR**MLLLHNNHVSKIQENLSEAVANLEFLMLTGNRIAQLAEVDHLACLAKLDT
> -------------------------MGPKNVSTPTLPTQVLHQLLTPSFV**RVLR**ASPDQFILELQTAEDISTATLIWTYPELTDVLVLHDVYLANFVATPVEDL
> GGLADATRRAKKAELQQLKEEHEQKGKVAEEKKNAEKYKRPRFFERVKVM**RKLR**QAKNRLEQATDKAERSEHEKKFQTYREEMMYIYYYPTSEPYISLFPSTPH
> ------------------------MGVAKKLRVLCMHGYRTNAKIMEDQA**RGLR**KALAPHAEFVFLNGPIEARGPSDDVIETRYAEHKPFYEWGSFIERKRPQE
> -----------------------MRVATKKLRILCLHGYRTNVKVMQDQT**RALR**HVMAPHAEFVFATAPFKARGASDETIERLYKDHAPFYEWGHITRLDRQSD
> --------------MASAIIALPTSSPPKKLRVLCLHGFRTNRQVMETQT**RGLR**DALGPNAEFVFLDGPFESRGPTDEVIERLFGDTAPFYEWWAARYLEKEER
> CTFCGVSYFVFAEVQTLQTTVKQYKKTFRLTVDFDEQEFVRFMERERSVA**RDLR**QQVTELKDDFVQQAATCSTSIRQLAEQNEMHRSERQAALELLEQVQKELL
> ---------------------------MILHPSCQSPTQSMQAARRTGIS**RVLR**ARQRLLVRVSSGRTRPTSSSCVSSTSSRSFRSSSAALASGRRNAADDSEA
> CRRVYDRLRFVCEELQRCSDASSLHESRVLALYGCAISQFLEFLEKNSQK**RLLR**RLIESSRVAERIQEFHEEIDQLFKLLSIVHMAEMSVWRKEWEQDRTLQRN
> ------------------------------------MSPALKRKQPSSAA**RKLR**KSCSLPTCTSPPGTCMHCTCDGRCGRHPAGLCGGRREGSGRGCKREGCTR
> SSFASSAADDGSSRQLHTVHGHVWSARGGSSLGSADDALTLVVTSDLSLL**RRLR**VVHDSFVTLQFQERSHVARLCLLPQAIVNSSDQEQVAAADANAKLDEVGM
> GFTWRWVVEVSTHFALQRQTAHFAHQYAQVLLRRRAAASLDQWDDARLVQ**RQLR**VLGLACVFMAAKIEEVHPPKAADLVAFLAERGGTQLAADDLARLEVDYVV
> CHGCGKTALCEEFEAQGYDILDEAFMDMPSYALHPQSLLMETSWVCSWFE**RVLR**LADRVKPGRKQVFIADRSPFSAVLYSAKGHLLEPVIREQMREVQKFAGVQ
> --------------------------------------MDASDERENDYL**RFLR**GMRTDDATIQASMPDLSTTQSAELDFRTSFVGADGLPEIDHDGAEFARML
> VVERRYSDFLWLHHQLSKQCAGYVIPPLPAKVVGLLQGPEFLEHRRAGLE**RFLR**KVVAHDELQNSNFFQSFLECSIVELTALKAEAQRANSLNAASLSSVVQRT
> HDLVLVFPRRQGGELKKPDDFTLHRFVQIMLGKDPERSQRANKIVVDPFQ**RVLR**TPRCFLDERGCDVVMDTSNDHSEDGEQCRLETQQQLLKNEYEKFVGSAEA
> SPDAVAASVQALRTSFRSGTTRDLKTRLAQLKQLRRLVNENVREIQDALF**RDLR**KPPAETLTMELAPVRGEISLHEEELENWAAPERVWTNVANLPGRSYIQRE
> ---------MQTSRPSSPTKMPSLSAPKSTTTLWTARLVATLWKLAVTRA**RKLR**RHSQGAADQVDIMYGSALDGSTVMCGEDALELDETSIMHEAALSVVASGV
> EHDGATLYVVEVYLQKAQQGLPKSNAKRQLLREQERERPECHVVHRYSDF**RALR**HEIYDVVDVTDDQMHPVWCSYCCRVLWLMTYGSFPSRLPNRGVVARISGW
> --------------------------MSRSRSCAASVRRYLSEAGEKEAL**RVLR**EQLHRQRRVTSDDVRLAVRAVASQGGTVPVPSDFPPTRWVLEFKRIHGFV
> -----------------------MTIQRRSAMSGALVRRYLSEAGEKEAL**RVLR**EQLHRQRRVTSDDVRLAVRAVASQGGTVPVPSDFPPTRWVLEFKRIHGFV
> --------------MDPDALLTSLLDEFLFPWSRLTLPCCVQHRSERLFS**RVLR**STDAARYQFAKWRQFRLAMCRAAQLGQLWMVQQLYERHPKALNGATALAA
> ------------------MTGLSDTVNSDENPPLAELRRLKSQAQSGERR**RQLR**REAQKIESKKPNPSDPEPQEAEMAKPQRKKKRNFFKWLLTPSVATNRQVA
> SDGKPVKARAAKKHGISSALAHRKAQKINKTHRDSQCYSTQLQQRKKAEI**RALR**DQALELDQQVKLLLQRRQQRLRVRGKTEKTPITQIEDDSREWFTKAVGEF
> -----------------------------------------------MEG**RLLR**SKIKVDPGPNYMHATLGIERDRDSDDDFVPDEENGTEEEDQVALVREKPT
> TSPMRSPVRIEFSSTAPALDVVSKRTKSYGNETKTTKRKIKETIRKQRYQ**RRLR**HERETLRRMDMELSRQLIKLQQDAEKQQQGTIAKLVLAKSIWRDLAIQQR
> LPGLPSIKRRRRRRSSSASDATSPAPLASSDSDSGDFLPRRLLPTASLSL**RRLR**HRSLSPTSVRSFNSAMATTMVPAPPYMLGERPTPAHGGSGAPSFRQVPFV
> RRFKSTSPMLASSGGYEAFGSFHTPSTACLFAQEDAKSDVPMVFKSDIAS**RRLR**LLNDSVAIPQNRSLFALDLETWTYLNHGAFGAPTKAAIEAATHWRTQADA
> ACLQRMFRCRKARVRLRQMLASVFEKFYDADSGLYYYLDKRTGETTWEKP**RALR**SDDDIELTAVYDGKEAATSEAEPTGEASALLVVDLDKDENSTSNEEGDKG
> -------------------------------------MARSSVASLLEEN**RQLR**VQLERSDLARRRVEGQVTEMQRRVSFAETTAAQQLRLKEGEWMAEYDRSV
> ----------------------------------MLVITSFPRRFPTASK**RPLR**LAPAFSSASIATRDASPAAAVSASSPRFGARSVHSKAPSSLSSTFATSAA
> AWPSPSSSSSSVSSSSFGVHVPPGLRHFEGRFQNLRGQSLFYFSLYPPPK**RALR**GVVLYLHGAGDHCRRYVPLYERLCEEGFGVITYDLVNHGASDCDAHKTRG
> SRRSRDTRSSAADSTSDGADTDTSSAGQLADTPQRRGRRRKTAEETRKST**RELR**SADEMRHSSRSGEEDDEDEQEDNGGRSRSLSNASNTSSTSASSMLSEPIT
> MELSAYEAKRQRRIEANQRRLQTLHVPRLQPRVRSSVRKKRGVEDAEPVR**RSLR**QRQLRENGPENGSEVFLELDPPPVRLKRARTSPPQPLGLPVTLSSELQSS
> -----------------------------MSAHTAATASVNGFQLEESSK**RFLR**SYGMSDLDVKEDSGEEERGIDLTKLDDVINVVKGDKVIDAAKLDDVINAA
> EEVAARLSIQLILKKKKGEKKKPPKTPKNRPASIERGTFIVTDALQIPGM**RLLR**WWDTRAVHMLSTGGSVESDRIVRREKLTGEQQTQVACPRVVKDYQTFMGG
> HVGDAFIRRGGRVFPSARVASAKPRPFGQRESLTDEADSASDSGALRMMK**RSLR**AARSFLLDDELAVLHTTLDAAGGAVQRLLKVATGAEDDQELQLQDAQDTQ
> DDPDDVFKAALSFVDEFEFAPANAQVQAATSLSVVPLPKEEKRRRRAERK**RLLR**QAGVYSDPNRARNEQTREIAFLREQMEKLQLNLHVLQNHKTRTKTTLLAV
> VDEFSCDEDAAGRPSCVTDNSVRLHGPTTPVVFSPDAHKQRRRDQINEKR**RLLR**KAGVYGDPNRVRNAGRLELLQLREQLEQLQVDVQVLTRMETKERPRSLIS
> SAPSGGDHGSSETTSDDETPMVQSARCKAPVRLSSHEDKIRRRTEVNAKI**RVLR**KAGVYADSNRVRNGRTREIAFLREQMEKLQLDLETLKKRKGEQTTSTRAP
> AFEAALSFVDEYSFPESDAHVQPSAAVVGPQPVEEADENVQRRARANAKK**RLLR**KAGLYDDPNRARKERKLEITFLRRQLEQMQLELKTLQTPQRRQDTGKQER
> ALLIRSTNGRLKHGDIAVAETEGIHPSTIFRIWTRAKTEEARTGRFASPS**RRLR**SGRRPADHTAMLERLQGVDVVERSAVRSVAAPPRSCECTKPLLTEANKVQ
> ---------------------------------MATVGKIDAEGRVLADN**RSLR**GYKRTEAEDEDRAISASLDDVILKLNAGKSSLQHVVDVKALDKAVKKAFK
> DFLQENGLSKEIASKALVVSGQKVEAPSSFSQGIAYVVYEQLFLSVYAAI**RALR**STGCTLPIELWYREEETNTSHPLLQELTSKYGAYLRIIKDPRAVKFYTKL
> APLQLALEDEAQLEAVARIFVQNTVVQYEKFIEHDRRKVDANNWKCIKHE**RDLR**VFLQRRRKDVVRRMRRHQRLRSFRHRSSETAAFSDTDTCSSSIGSRDSSC
> HDGFFPQIALSREEVKYYKRLGKERVAQLVRIIEDADCAYKWTNASSSSS**RRLR**LEPKSGVTCQRANFLDFQPANKAAHASTLLKASVQLSDTRVDEILRAIAK
> -----------------------------MYSMFPMVEKSYRVQITQDIE**RDLR**FLGQSRVDALTSPFAMKQLKDKGGLKLFELCEKEFYTMKAVATVNAPLQE
> ------------------------MYPVDEETFIDYPISIANPIDLTKIQ**RRLR**LGYYRQVEAFLSDVKLISINCETYNIPSSPISQNSRNLLSAILTQAQRHF
> -------MQDVHDVEAKLRELVRSWGVSTFSERIVLQWLDRFLGKDGEDF**RRLR**KWIVMQLNQRRVPDECCEWQMGCPQVIPLLRATPVWDCGNFPWVETLESN
> ------------------------------MATIETSTTAQELQKTLEDP**RYLR**GVSLLKEKRLEEAVVAFEDLLRTLCEAEGKADSLSVAPVYYEYGHALLSL
> ------------------------------------------MALEKDLV**RALR**PLSLGAVPTSAISSLFDTGGTAALREEWVDGVSRHQLRALERVFSKFLRR
> ARAWVEKIAPALQTFLAVEKHLMVPLKFVVPHGDAKWPVEAWGYPLGLHC**RNLR**VIKQQGKKLPFFALDDLEMFDFPWDMRQRKWEVLVLPSLKNFYEINGHSD
> ASGGLGDVHSNGGDIDSLLSSDADVEGKMRRLQEPYERHRAHDLRAECYR**RGLR**PIKKGPNANDNKSGYIDLLRKFDGTSQNGPPNGLPPLREDQMGKRGQDEA
> ---------------------------------------------MPAPR**RALR**PPSPAMHVDSPQYQEVATPTHELELELLADGTMRSRSHSHALSVILGAST
> TSACNGAAMGKRVAHRSNSRTSSSSHRPANNSRSSVGSFDRDGDATRRQE**RALR**RWNMLRTHVRVTRRLSQELAHKHARRFSSAQQSLVDACIGCISAPATKER
> ----------------------MTPIHAQPVKRSSFRPIPDRYETIAEVQ**RDLR**KAGLESSDLVVGWTGERSFGGRCLHEIDPSGVFMNPYQGFVLQSVISVIG
> -MRAATRDGGAGDLHVLQEELKKLGISVVSGRQDTVISVKVKGLAGRALI**RGLR**LADQLLQEKIKHAIKTCTDPATKLFTDPTFGPSSNDPDGAAAMCKAGSTA
> --------------------------------------------MSSYLR**RHLR**PLSRAWRRAPNGLRGLCTADEDKYLELKQLKDIKSEEFYDLQGQKRRYFY
> -------------------------------------MLRSSLRNAARSQ**RLLR**YTGAVPAHAQAPSRIEPLASFGYDFSSFSSRDVRAPLMLPLLPQYSPLLF
> SDYLPTDIEEDEEVPYEHPQSETRGAADGPGCVAMDATADVECSSESPRR**RSLR**TLYVEYWSRTDECKERKWDHLDEIAENYGSAEEHIFATTLKGRTAFLEPN
> DVFQPKQLKKKNKRKNKASAEQQAGPKLKAAWVDEDDEDVEVNLEEQSQL**RKLR**KTEKDAVVDGKELHHRLKSYYQSAHGSTSWADPKNFLGDRPEDSDNSDYE
> EFEQDAHMPGTPPMDAQSRRFWGEKPLPPNPKETEARRLQEQEKLQPEER**RALR**HRAARVTQRLVRCFLARCQLSRRLSAVGRPVELQITRVQGLEMLCDFRNV
> PAPHLNVGVFSKRKGVRYHRDGQVRSCRFCEILRARAEPFLYEDEDVVVF**RPLR**PIVPSHVLIVPRAHIRNVNRLGARHRGLLEQMYRVAEAVMTQELESQEPQ
> DLLMALQSMEPCGNCGAEGAKIPCMSGCGEVFYCSRECNMHHASAHRLRC**RNLR**CSKFNDSESESESDDSDEYETDSSAEYETDSDEEEEEEEEEEEAKPKKKK
> GTRQGFAIYNCEPFGKCFQEVIGGIGIAEMLYCTSLVALVGAGDQPAFSP**RRLR**VWNTKTGSAICDLNFVTAVLAVRMNRQRLIAVLERKIYIFDISTMKILET
> DVALAPNSSVILEPADSLSFHLQPQTAPQAVLTIRNVADDRQIAFKVKTT**RPLR**YLVRPNQGLLGPNGSASVMVILQQKDCDELLRLDPAERQLANDKFLVQSI
> CRMDAAFGDVQPKVELRFKDLSVSADIIVKDVTESAADLPTLPNELVKGF**RKLR**AKKHLVQKQILKNVSGVLKPGTITLLLGQPGSGKSALMKVLSGRFPVKKN
> FLEPWCAVLRGRLTSEGRFSDHSALGEYWYVAVRPPPSLMAILNREASDV**RVLR**EALELLSLVAIVDNEAWKFLLLQCCDLALDDDDTLDGVFEPRFLLRFDAE
> ------------------------------------------MSGFQARW**RELR**KDGWTWKRSEGLSNDMIYIKPGKTKKDVRGVDVFVGEKEIMEYLDNVDLG
> ------------------------------------------MSGFQARW**RELR**KDGWTWKRSEGLSNDMIYIKPGKTKKDVRGVDVFVGEKEIMEYLDNVDLG
> ---------------------MLNRYFELLPFIDADDDDIADSLPAPAAK**RRLR**GLLGELKDVESVSKALQGDHVTLLDARMWFDSLIAARASYARYLAPRAAI
> ---------------------MSTREHDEASVLLRVATTGPGKSEYSFPD**RGLR**FSSRFDGGNMANVQLMPSGAFSIKVAEDAAAFGLSTGYSTWFYFEVERGG
> LEHLTLEMTTNINTRHGLGKRNVLYELHMTYQPSKDEPTTWTLSRAFDEY**RDLR**RRLVKALRLGHRCNAECKWLGNVIKHHFPRASLFCRSCPSGVDGTRVALL
> ----------MPPRAGSRSRRRSSDKLPALRPSGSNQRDADAVAQWDDRH**RRLR**RRKRRELEPLPQLARRCPSSSDSSEVDDSSASVSDEDVRVPPSTITTMRS
> -------------MPALKPKKNEFSSAKEFKTLLVTASKMHKIRVSNEAI**RHLR**LTMNTTIASASLLPEAINVIIASLAERKTRRVGLEDVREWLTPSAYDDDD
> ---------MAVAPSFDPLPASPRASMLPTTNVDAVGIRNGHFVDAQGRV**RLLR**GVNLGGTSKLPIGYQHTDRHCKNSFFDGARSVSFVNRPFPLSEAPAHFAR
> ----MSSALTKFYPEVTGTSLETKRKNIYLWRKRRDTIEKAGKSSLSNVL**RKLR**SAGSAIVLSSETESQFLLSINAYRADGAPVLALMLQLKAQEYASAAGIPR

> ---------MAMPEQPELKKAPTAVTDTSSTSSADSDKPQQLKPKDVDAR**RSLR**ADSLGVDRSSRPHFKEGIRLAEMGKWGALVERVASEPQLARHKDHHGMLP
> ----------------------------------MRCQAVSSAPQASAMA**RTLR**LARQTGSLNLSSRDLCAFPVEVFRLYELLDEDERSWECAVLKKLDLSYNE
> IASPVRAEYSVRELGDRQFSLSTTSEEFVREDFHLQNPRGELLACSFWRR**RALR**DADPIAELVSSSSSSSLSNEEDVTSVVCAVEELMADNASTDSSNSSWSSE
> TYYPNGITLRDSVRKRLGDWTQQQLDQIEIVEEEVSEYVRYGVFAFRVER**RELR**NRSIVVFLREIVEDLLKTPDNDLRPLTEEAFVPRAEPYAASP--------
> ------------------------------------------------ME**RSLR**PLVAACVEGVNVSVLVVGSAKTQKSKVLFVPMPEQSLASAVFQTLFDMLQ
> ------------------------------------------------ML**RLLR**RPLARARHLRPDTFTSLQRTRAISSAFDARPQELSASGQSGRVIFGAKVA
> ADLPAAFASALDAAEARAHERHVAFQGRKQSFVILIRELYALLERTAQD**RRKL**RLLLQELVQRLLDPHGVHAQEDGGTGPLDAAVGSFVTAERETFLRVGGDEC
> -------------------------------------------MLAKQVT**RALR**PSAQTLRFLATSANPAAGSANVPAKSAPKFRKKKTFQFSGKDEEKTAPLE
> -----------------------MRSLDDVQVQVLSEQARRRKSASPGAR**RRLR**SKETEELGAPAVWIAPDWASHPVIESFYGHARRARASSFYEVMCSPMNAT
> ALKVRWTTKEVAMLVEVWADICCAPGAKRLRGEQLNKAIFDQYNARCART**RQLR**RSPTAVNTQRTRMTHFARFVSDFDRAQLSQGRRTWFQLSSEEQLDMDIPN
> DETELRPLDLETIMSSKAAVKFWYLHRPKTMAYVHKNATKMQSLGRAFAV**RKLR**ERHGAEYMADLARQDQARRDADTLKELSPEERAAKELNDAAYDARVEESR
> GCRTIADGSSAAAAQLEQLFERTVQFGENQSGLLLGVAGSYRRSIVTQAM**RNLR**GKFGDFTVVYLNGVILQNELEAFKELTAQLTRATSVKHPVLSYWNMYEYL
> ----------------------------------------MGPPGRRRIG**RTLR**RSRRRSAANGSVVTRATAGAGPLQAIGENTVVELTADDALDGLPPTLASP
> ----------------------------------------MCTPQIQKAA**RSLR**LVPSNASCTLKCGTGFGYCSQVGQGTANEVELLDLREELERKEDKYEVDK
> EDVLKNQFMNFPKGPHVKENLQDLLGDGIFAADGVKWAHQRDVARGLFRM**RELR**DCMTEAITRHTKALHDVLGKVCARNRSVDLHKLLSCFSTEAFAEISFGMK
> ----------------MCENLRDLLGDGIFVADGDQWVHQRKTASNLFTM**RALR**DSMTVVIQRHAVVLYDILQRASDNKETLDLFRLFNRFTIEAFAEIGFGVH
> PLTVQERLSLLEADEVMLKYPSGLLQPFSRQELGRLKKRFVHMIPGEDAS**RELR**VTLVELLQMPELRAQPFAPLVCRTVLDRQTKEAKEGDLVSFAAFVTILAV
> LPQLQHYNAPQNLHQSIYDCVHELEGDWSDLGAMASTQMAPEASRTGASR**RKLR**VVATIPLSATRNVLRVGHEWEFHSILDAQTQLKQNGDLRRKMKKLVGDVV
> MFNKGAVTNAEPPAWKRRPVKLPQDNIKAEVKSQAATTSSETLVDAAVQE**RKLR**EQQERRNAQDTRDAMAKQSSYDLEHDRVYHHAVKQKEAERSAELKAKESL
> TEFSNDNNNMMGQAQASGKVQVGDEVYAVNDNRLEVIGMEGFKAEVATGK**RPLR**VTFRRLLPAVLGGTAMGGNANLEQQGQNAASNFATPAAQPGQMSSTLNLQ
> -------------------------------MTPSDTEKTASRCSPAFQM**RSLR**PVSAKIISYGRENQSHRRPTLYVLRVSRSGAADWYVAKRYSEFRALHDAL
> --MEVQERRNSDIDVDVEAVTKDLQDSLQLSNQDEDRETEGAEAERRETL**RTLR**LAFVGNVDSGKSSLIGTLIKGDLDDGRGSSRQAIFRHKHEVESGRTSSVA
> --------------------------------------MDAEVQQQQALM**RSLR**VFVDALRDIKKTDDGDSVHHEVVEAVRGLCGSSETQQDIQWHDEAWQSGL
> ----------------------MLQFISPTVGPKLDDEAVEIERFRARHR**RLLR**HQAQQFTPSSKVEDLDVDDGRKRKISANWRLKQQQTQVSIQQGDGHWPAS
> --------------------------------------------MDPGTK**RKLR**ICAGTQTLQLALVTGHQSARSLSGIFWALPSSASTDDKKMGKNVGANANW
> PSPRRRWPGGGSRHQPNRPVNRTPRLRLGDKRLNILLANRTTPEDFVSAC**RALR**CKDEISLLAATDMRVWNVALQRRLEAKDERGAAELLALVASFDVNNAIEP
> ----------MLRDLFLAYYQSTNRKPEHVIYYRDGVSEGQFYDILQTEM**RALR**KAFKMISEGYNPPVTFIIVNKRHHMRAFPVNQRDADRKGNVVPGTVIDTG
> -------------------------------------------MAMQSSS**RKLR**LSNPHAELPIKNHKYVKNCTELYMANKRIDKIANFDAFVNLEVLWINGNQ
> IEDENQLFDAIELQPADDEDEDDLPPVKTKRLSSGTAASSDGEALTGDRV**RELR**ERVLEVANRPVSPPAKNHFRRASTVAVPTQGLVPRNESRDDSDSEAVYCV
> ---------MSRLVVAIGHVEFTGGSQVLYVVKVQQDADEWEVRRTYSDF**RKLR**DDVQRVMKDARAHILDDACSRDFARELQDVAFPAKRLFGSKKDHVVKERA
> ETRAAEALAMKDEQMKILGSQNAQLLTSLNTMDDEIGTLKMHKLRLEEEN**RSLR**DQNFELQSKARAAETTLIKAQTGIEERETQVRVLTDHNTELLRLLEHEEA
> -------MTRLHFSRYNEDVDSVLSEDDNAASRGFNQSRGLPALNRSGFE**RSLR**IDNVQVSHIPILGRVDNPLMSSVVIMNGADSGANSPGWRKAEAELEGGGA
> ALQARVHKLLGSRAELEATKALLRTLVTDKASSGASLVQLDATPTLAALR**RNLR**SNLEQQQLALAQKALDGLEGTLAQVSNLATQVDALDDKCDHVHKFLETTK
> -------------------------------------MKDVARDWAAQGL**RPLR**VWHGLMRRFNLDDAATPPLVTVQRLVHNYVVGTLVGSDLLKSVRLKIRDA
> ------------------------------------------MKTRRAHR**RKLR**FSSAKDAAPVATVKAEHAPKRPVKRRKRQHHSRHVSPVPSSTARHPNGLP
> ---------------------------MYSGNAARIYNPQPDPLERGPPK**RVLR**EEQHIGEMVAASKKRVTWRFTLGDSDRTHEVVLVHSVMSYKKATLGDWSF
> AFVPFGPVKSVQIPTEYSTQRSKGFGFVEFEDEADARAAIDNMDDSELFG**RTLR**VSIAKPDKPKLGSNKPVWAEADEVMDDAEEPSQDTHGSRPQA--------
> RPTSSSGLCHQTRCRPDEYASPSETVANGRQRSPTVASACHVHVHNEVHA**RHLR**SRAARRPLADVTAVVATTPDLVQRPVPPNTLPAERIRQPIANGRQRSPTV
> -------------------------------------MMNLRSALSRPLQ**RALR**PRWVSSVQVTRDTGVPDLTITPLAAQKLIEAADRQKAANMMLRVAVEGGG
> REGTPGAVEGSGKVPHGSVLVGMDKFDFVEQPRKTLAEVGAILREAAHLK**RQLR**FRVPPQNYQKPDPAAHENDKSKLLTGAPSSSASVVGGNETGKPTGEWSSW
> ---------------MAKDDAANIHRPAKRLRQEAVWSAGASRELLRLRF**RKLR**GLFDEAQTVADVHEAWGIVATAINENESLGLQADAVTCSDQLTKLRKQWQ
> ------------------------MTQLRVFTLDFVDRLKSKHYMRRRMP**RFLR**PSNGRRSLLRWRRRSERDLVSKLSQWDRHSLLIQSRRCRSVSKVQDARPR
> GNNARHTSYISVDDKIDETLHYDVQKTEESEEVTIEVLQQDQTTPLVSDR**RTLR**EFMKSPQNMQMTFTFGPKSALNKMQLTFSAEWTPSNMQPESTTLASAETH
> --------------------------------------------MASSGD**RHLR**LEEYASSGAAVECEDVSDTPSHVKIRSMRSLSKSDNGGAARVAHSASASH
> ---------------------------MWRQELRWMRHVVRAQDSELRLD**RWLR**RQFPALPQSFLQTQLRKRRIRLQPPPSIDAQPRLQAAQAKSLLREGCVVA
> ---------------------------MWRQELRWMRHVVRAQDSELRLD**RWLR**RQFPALPQSFLQTQLRKRRIRLQPPPSIDAQPRLQAAQAKSLLREGCVVA
> --------------------------MEKLFTKIGAPSYLPTPTEVLDGV**RWLR**ANPAIAAGVMVGLTSYSVAKYYEYGEDSEESDEDNDDNVADSPRATRDEI
> ----------------MPSSLSVDAAAVEDALLRGEWTQALNDSRSMLAA**RPLR**QEDASAMTTDEERVLSVYLQAVFELDLDDEVETAATIARAFAPLPSEIAI
> ----------MVGNTISKKKSEEYLRQRENGFNLSGVHQERLPRYNALLD**RNLR**HHFESRPLQNHLNDLGLVDQRGRIVDLDKQKSKLFIIDQEFKLAEEAERK
> ----MLWPREEFHAAYGQMKHDALQSGGSCSVLILVALDVDALCAAEILT**RLLR**ADMLSYSLLAVRGYEDVLQARETRIRGADGEVRGGGELRSVVLLNCGAIV
> ---------------------------------MFLQPRQTQTELHGYSV**RNLR**RISCELMQFLRGESHEFSRKSLSYQVEFSPFVDNLVAIGTAQYFGIVGNG
> -------------------------------------------MHPQLLH**RVLR**LQSQVPLGRQAARQMAVNGVALARPLQTSALRAITKSCNVEDVHVAETEN
> -------------------------------------------MHPQLLH**RVLR**LQSQVPLGRQAARQMAVNGVALARPLQTSALRAITKSCNVEDVHVAETEN
> FESSEDVILAASGRQCKLLMPQGSRRLQSPASEAAEDEQLHPQHPHRMRK**RVLR**RESSDFHQSAERQRLVPFSRHNNRPMEFVFLSSGEIRAREDCPPGLGLPK
> SAPKPQQVKAEQRAKLVRRNSRVTVDQLLREAFARVDIDMDGKVDALDIQ**RGLR**VAGVKLSADDVGRLMARLDPKNQGQLSYEDFHALHDAFIVQTFRSFDKEN
> -------------------------------------MNQNDSFAQCAVP**RELR**CTYRSKPCPNERARKRNGTLHKLCQTHRLKANKTQRDMQRRLREQRRQQQ
> ------------------------------------------------MA**RGLR**IGAKADVLKNLRALLRVARARSSQDSVRDCKFSQQILAQYRARQHENDRA
> KRQLTSTLNQLVQQKSVAVLYTPTEVERITQMTDMMAQATSDYEADEDWD**RILR**VTDALSSVSNRAVLKESIRYLKLRLGDPSTRVVILALTLTESIVKNCGDL
> RAGSGGSAVAAASTGSTSSNDSVVGLNGPKGYTREELINFWRELSLVAKR**RLLR**IRRESYLTALDQFLVGQNLCCECHDNVIAEWEDHERKRSGSRSLQDVFSV
> RVAVVTRVDDSPEEDFPIDVDTGEVIPKDMMMKRVLDRFGVPVEAEVRTW**RKLR**TYNLVSGSFRAPSRAIALKEALENAVKASVEALHDLLPRVREEIVPESPL
> --------------MTNGQQALRAAQDFDYDVVLVERELAAGDMSGLEFA**RLLR**QSQLKRRLKANSTSSATILCVSSQTAPDDLQLYKDSGMDGCIGKPIGMES
> MAAIVEDPPLELTQRTRAMDRELKPWALYDLSGAKSPGSLEEITGYFRRF**RALR**GKSLTGVSHDALQRSWCAMIVRWNRMHQEGSNFVAWLTSREEVVGEHSLS
> NAQLSCGVIGDATKIVVRSRSSRLFWLVHMSTEMWEFAPDGELYYEKLLH**RLLR**VLVARWSDGSVSHSVTIIAFSRSFYDANQFPDGFDPTRPPFSDPRRQGFG

**END OF 1st SET of 382**

> --------------MGMLNKLQPVLEAARERRDKFLIAMIIQSNDIMAAV**RLLR**LTELGDVPQVPMISHRKCLQISNEINQHKKKLLELNQQLSETLNLSNVVS
> QKGKREHLQSSVSMSSPTAGGGVPEKTVLAKHEDQLAVMARLVLSEKQRI**RGLR**RMRQIRYRKKKDNYALNLEEEARRLREEIEKLAQERRAAYVVVSAGESAW
> ------------------MAALLDRTQKLRQRIEALIRRDAAKRSLPVDE**RALR**RRVDDYYLPMFRWTTEVVEAAQKKQGDAKRCVCIGLSCPQGGGKTTASMY
> PEANPNTMEAVQEKNKALEIDMKEKNRRITYLTHKCEGLYRSQGLAGASF**RCLR**RQWFQLQDELLAAVRTVDPSAVSDEACKEAWQAALEAVDGFGQCRVRAEE
> -----------------------------------------------MSA**RRLR**EATKITPRSAVGTPHKAAETEHEYLRRCVDPVLMPLIESLLLYQPESVFH
> RKSKLDSTGRVCYELKNDPLLLDSFPMDRIMKAMRRLVCDSDTSTRAGAL**RALR**YAISSNGSIKHFVDLNLPVFVVRSLEREQKHLAERVQALKVVRRVMEIDA
> --------------------------------MDTECGALVVSFLTLRER**RELR**AVSRRWLFTSTWLQFAGNNETELQPKKSVWNFRSRAFRHDWQTFIRTACH
> ------------------MNATAPALIPPFRFSTVQQGLYRGAYPTLKNF**RFLR**RLGLKTVVSVIPEPPTSDLADFCASEKIALHHFYAEKFTSDNVTVSPATA
> ------------------------------MSDSGTGGDYKEPSRAIFAQ**RDLR**HFLGSPAQQMILVFVKHLNESVKGKSISSDCEVSPNVQKAVDLVNEINSW
> DHRPQGAAQLAKHLVADTAAAMAASLGVAPFITIVDRAIIENASGARPLG**RGLR**ELSADFVRHPLRFVGKREFHLIFGLYTATYMAANVTDTACEYAEMDNQMP
> ---------------------------------------------MSGMQ**RFLR**LSATEAEAAMKPKLVEGKWKQPLISGRKVAMVKKHALRNGLVGTWEEGKG
> -----------------------------------MSANDDAKGMIAQEE**RELR**RVFEHLGSYRQKKRLALTVNECKERRQRLESSRNNHEVSALLNEKGAKMT
> --------------------------------------MTATRVLSRCTD**RKLR**LLCLHGMYQDAATFSAKTKPLRGATDVDLVFLDGPFTIVPPILARQSKRP
> IIDGWTHVNKRLANVLGVPLVGCTSHRLNLAVRDYLGPHDNILEEVQQLM**RKLR**TLKQASKLRTKTSLAAVLRQDTRWLSTFSMLKRYFLLREFISADDEELGD
> ----------------------------------------MADRVESADE**RTLR**RELLELSKKREALDNSKRRGSGAAVGGVAGRRSVFARLGAEPTRAGGGNR
> DHHAPSSEEETKPTAENDNSSVSSQEGDDEPQPEPYRKTLKEGEPGTLTG**RPLR**LYADGIFDLFHFGHAKALQQCKEAYPNTFLIVGCCSDEITHKLKGRTVMT
> -----------------MGVEDATALQLLRAKQNAYYIQAVQDALQAEEQ**RKLR**LHKTTSKQEAKRLEKQFVRERSADRERLRHIQEDHALLLNAKIAEWKASG
> SEDIPRVPTQFDVEGDADIVVSTEMLLTPQQVERYRKLEKKQLEVWQVVV**RQLR**VWSASSSGAADGEDAVPCRPYCVLVNNLYPLGQVVSKKICDPPEVYPSPQ
> LDGATLAGGKRDNKLVNGHEKDVEEVAELTRALDAGQVDVAVHWLQLLLF**RKLR**KSSRVGRLKTLQTAVAQMLKDQDEEDRLTLRPGLASRTADASCELSYSQL
> LYEYPVTNRPAGSLPNAFVLCWENVLVPTKWMRQELGLKPSVQALHAAKQ**RVLR**TPNLQAALASIEQDLIQLLTSVSTYGPLFIVSEESIQFVEATCKTLFPRL
> MALYAAANGGALGLSSSSSSSPRADQRQDESIEKEAKAEEAPEKKQKKRV**RYLR**DTERHNIVKRIEKGEKQAALAREYGVTRAAICHIKKNRDEIVTRYDLLIQ
> ----MHMLFQSEAAYRRRRSWMPRNGDDAAAAATTTLTLRANNAAGLIGP**RRLR**GRTRNCKTVRREAKAATRRAQRKQKQEIEPEVEDDSDGEEGGEVDNQEEE
> ----------------------------------------------MDTL**RQLR**LDHVALTRHHNAANAALGHHADIVAGLSARAKAAETSAAAAQRLIRKDRE
> ---------------------------------MRRLSTASASTSTAAPA**RALR**RLFQHQPIEELPELKSILAVQNLVAKIPQQPKPHALRENEAYRRWIETYR
> ---------------------------------------------MSGML**RSLR**RVWPGSRDIQDLFSPLRRLKPLASQLPPPSKPDESKPLSNEQRLGDVVWK
> ---------------------------------------------MSGML**RSLR**RVWPGSRDIQDLFSPLRRLKPLASQLPPPSKPDESKPLSNEQRLGDVVWK
> LLWSRASDFVAWLGLGPNAVPDIASIPAQNPPPTKKREPTSIPPNRLAAK**RVLR**PYPPADAAFPTFQNSLDANKRKAASYALGRFSQKVKYVPTSEQLLDAWQV
> TLQSSDTELKRKTPSQWRPQTAVGAEFPLFLQEASFDQVSYGKKLFSDLA**RHLR**SSSDPAQLLQSWNVVKQCQPFVVAKDGVRLFDEAADSKANALGVLQMLPA
> SSSISDESSLASSPSQHHQQRPRFDSKASWADSTRFDMGRADSRASDMSA**RDLR**LTGLGGSHTRPRADSRTSRLSRSSSRSGLRGRQSSFVSNFGVTKGNKPRK
> ---------------MDADSTAGGDVFQLYQQGLTPDHVLQLCYLRRRSP**RLLR**PERTRIEDEFVLPRPELKIASLLPAIDIPLQQLLAAPRVEKASGCVAAGL
> RYTHHGIVFAGARSADAISVAHVWSRKSGLRASQADSKFQLTSLSEFLNA**RPLR**DMRRVQYNSSLVAEASSLLGEVHRTHADAPPVVLARCRFLLGLGQGHFSI
> -------------------------------------------------M**RYLR**AKGIRKALRQFHFLCGIKPPYKVLLDGNFIAMCMQMKVDVRERVPKYLQV
> -------------------MSNIEEARQKLAEDRASIAPWLRAEEYTALP**RQLR**LGVMHLLSEALTTADSPHTSDHMIEDTLALSSYGTQYLTASYPQDKCTLE
> ----------MNELPADFLRGEWLCQREIDLLQRTQSAHQQLTSRLQSLT**RELR**GERYYPLQSSAYGLTLVMVVQTETKQLRVRVYHSYVPPVEASEKAQAAPG
> KGLGTDEQTLITVLGSKSPEVRNLVALRYKELYREPLKSLVKSETSGDFG**RLLR**MISTPLPETEAQILRDATKGMGTTESLIVQILSGRSNEEMNILKRSYFDL
> --------------------------MSSVEQHRELVVDGDVAQALDLCR**RLLR**TDSGLQRVETAHLVLERLRSSNDSSDDANALLRLLGNYVAPTRELTEEIL
> AKETARHKMQDAQKAARDKVDETREAAVAKARGLGANMLHNLRSNQKNAS**RQLR**ENAQELQRSASDTVDGLKKRAQQSATDAASAAQERVRSSVSDGAQKLKRT
> GTWRVGDLSPSSTIADIKRWVFEQHSLAPQQQQLALDQHGAQKTTDTQTL**RELR**VGHGDMLFLDYDGETVATGGVVGTKINADGTLTRVAYHDRADKQSFRPGM
> LAALHRHRGDFAAAGAGRPITRQRSSGFEVERSTVRSAAATCGMAPATLF**RQLR**LGRLRVHTSVTKPLLTEANNIQRLQFALSHIQRDTMLFDDMLDAVHVDEK
> DNPDWQELVRGDVDDDIAVNDDGNLEVADHYEEYDAGVLFPTCVEEVEAI**RSLR**FEPMGDIEAPIDLFERAGGSTMTTLRPAYKHIFELSTSSSFFAYLPVYFW
> RVAYNYDGAHGVRWAGEASAMDTDMATPRVRAVRSDYAEANAMLRQFHYL**RQLR**KFQAERGRARANYLADEMKQ------------------------------
> CRLEAKRKFVEAQRVWARTEPISIPSSGRPHPHLCASVAESPDLSYHADK**RLLR**AGRYLHHEEELDEDDPAFYDNLAFFRSQQGATQQARVLKRSVHPLQNAST
> FRRDLEQLVHDGVLSSRRHAQRSAFRPGVLALTSEPADVQLQQRSKKKLL**RALR**SLREDYGASLALSKEALGFTNSLEDALSFLALQHSSDELPPTLRALPTQL
> QSNRHGKCKVSWYRNSFGDTVPLPEMPPEPLALDPPMVIPPDLRPLGSKR**RYLR**TMQLLADPLPEDDMNRDLQGKLTETSPPRPIGRLLRHQNSMSSKRLLSSK
> ------------------------------------------MALHRGGM**RALR**TLRQCGETPSGGVSGYRLHLKTDETRLREAFSGYGTLEEAKIVTPAHSSM
> -----------------------------MQPAAATRDRAAHLAELYGSL**RDLR**AFSGEDERMRWRWRLHQLRRQQQLEQQDAPEAWAAHMASLLAAMENQIAA
> ------------------------------------------MADGNAYS**RRLR**SRRTRSASMSTSSEEEELEDITSSGGRRYGVYTPEPVQRTLVLRSGGLEE
> QDVQTLKMKVARNHDNDMERRRSSTLRLEPPQSDHRKSTSFVRARTSGVN**RLLR**SSTSSVGGLSNGHSRSSRNRSRGESRSAPTSQAPSENFETALAEPVDEHA
> QRQEMLGGDSWNAVELFYGVTAHLKGDANKWFMTMSDSMDDDDCTFTYLT**RKLR**KKYGRRENSWQIQKRLGKREQQSGERLDAFAGSLTSIGFGKRVSAESYLE
> NSHGVSSALAKFYPEAKGSSLETKRKSFYLWRKRRDTIEKAGKSSSSSVL**RKLR**PAGSATVLSSQTESQLLRWVNSYRADGAPVSTLMLQLKAQEMRAVMHRLG

> -------MDGTLVQTAHSRICGLACMRSSRREKPAAPAAAPHDVDFGHLW**RQLR**AAGWTCKRPTGIQTEWTYKSPEGENVLVGERAVVEYAFESGLLVEDEEHV
> -----------------MADLVPRLMDYVETFPPDGDVDEFKSQLSGCTH**RDLR**TACSRFNLPGVRKQNKKGGFISLLVSYWKGEAVPSSEGNYLSEQPANHDN
> TWIGPLTCVRVVRRSRPHMSALPHVWEAVEAFLDCSPSCSLTQAAAFGSM**RLLR**GISARSGAMVTLDAFYKQAQFSGAMQHAAARGDVAMMQWLLTFQPGGLVT
> AHPNQEKHHTTNPEVTEEELYVGEDGLISVSLMDDKSRLDKAKLLLERRE**RELR**QAAEYGLGLLEANEELQMQVVTLKIQLETETEELSVERDMWQRRTEHAQQ
> QDYDSRMEEQVEDLRDKMRLLQVDRKSNVDLLESSKQTNKDYIRQLKNEN**RELR**KALADLKRSTSSVTGPGGASQVLGQSAAVGGGGLVDEADEIAVAMQQVNK
> --------------------------------------MSVRVQVLGGYR**RLLR**ASRQAFRGDAYALQQARAALRENFVANRDVNDSEQLDGLVKGIDEAEGML
> --------------------------------------------MMLHVA**RGLR**GPRPLASPTRFRSDVLLRTSHVDTLTARGTELRALARQQQKTLLLNRAG
> LASYTTSVQGFAKLNSRAEWEDSLHDKGESKQVQRAVVHLKNQHESQLHS**RRLR**LAELYNREMEEWRDACLANVETPEDRKQNMIQRALALKERRERERQAIVE
> ---------------------------------------MSLMNQADPSG**RELR**LWLLSAATHRGITYYRIAGRFTSSGQTWEVSHRYSDFLTLRDQLVKFLSS
> ----MAPNPAAAPVELRRLNFAFAKVTIGGHLVNRLQHDPVNGDPQQEDD**RVLR**DVDLTLQSGQRVLVVGGNGAGKSTLLSILAGKHLTADDTALVFGRDSFRD
> -----------------------------MASTDLRKALSKYALPSANEL**RHLR**AADPTTSYRASFFRLQVDELLAAAGRKPSAKAAAGLQTLLFQMKEILQSL
> QQRHTQALRDAAESLDVGRATAHSLSLQAEQLGRSERVLDETQATVAMSR**RVLR**GMTWGGWLYNKFSAAPELAGDKGSAEWWTESAVAAAESSCCDSMEDIHDK
> QQRHTQALRDAAESLDVGRATAHSLSLQAEQLGRSERVLDETQATVAMSR**RVLR**GMTWGGWLYNKLSAAPELAGDKGSAEIAMGFICPECRVMFESPEQLGTHY
> ------------------------------MFLVGRRGARAATRPSLAPR**RQLR**PTHKSQISTWPRLAPHQALVAYSRHCADAHARLARDAWTRRNAELLRVFP
> -LVKRSEISAEEKLRVLAWHRANGCNQRHTAAHFQASEPRFRPLNQTTVS**RWLR**SEDKFKKLVLSHQAAIIRDKSVRHPQVERCLCLWLDRLPLNQYIKLTGRE
> ------------------------------------------MKVVVWFR**RDLR**LHDNLALSAALQLMQQQQGVELIPLYVIHRPQIMRCGANRFQFVLESVQD
> LKSAGKGDGSRLEMTYEISDRLLGHGINYRTIGSINARLWTLENQLQNAE**RWLR**SRGLRHYNASKMTEREVLHMCPYYPEIEPLLRPMRSAAISVSEGAPTAAL
> -----------------------MVLPMRRLQPAAMAMARSTTRSGTRVG**RALR**FEHAAQLGAVQLIPTHSFSGRSFSSTPATGYEALAGALMLARLFNDDQIV
> -MATPSRQGRQPPPPAHRSPNTQAASGPDPKKRRKDIATPEAPAKPLNRK**RFLR**FLKSHELKYGLTPVARDEASGDVTLVVCRFCQHFGREQRPDKQRRSTKNI
> -------------------------------MPGAASSRFHAQLVMTDDK**RRLR**RSKRHGELVLGAANQSVQLVYPRLGRYFQRKFEQPLKCVLGRQLLRVYSS
> -------------------------------MGETQVMQVAPKAKPICKK**RALR**KREVDSRWLFNNIHVGEMLVDCRTRELFLGNTVHGAVNVPPPEGSNDGSS
> DSHGVSSALTKFYPEAKGSSLETKRKSIYLWRKRRDTIEKAGKSSSSSVL**RKLR**LAGSATGLSSETEMRAFMHRLGVDVAYNADQTPVVFDYVPRTTLNKRGAR
> ---------------------------------------------MYVTR**RLLR**STMPPEPVEAMPEKEQKKAPLLHTPKRRAAARASAKISGRKASPKQTENG
> -----------------------MVKFRVRKKEEADHLRSEHRMLERVMK**RFLR**TLEKEASLKPMHVGIGAHCTTNRMHELVAETEAVRKQNMALLKVIERHKF
> KAAQLCLSPTDPSEDPALASPVLTGKAAPVTSPNPAKTPLPGLQFAISAQ**RELR**LPSFSSTSFELESGATAASSADEEEEAQQSPDKAAAQHRKKGTRRGTLHP
> -------------MFENRQRVEEARTRAKQGALKDTAKSMVSLFNKKKDG**RTLR**PRVRRTTPLMDVGLAVVLGAVTGVYIFKDTLQRWQVTEGEYVAAEVSKAT
> ------------------------MARAGKGSRAKQLQPHQRRRLSEEGK**RLLR**SALMAGSEGEDGSVDFSVITNRDDSSVESELEGALQWEAQQQQASASGGR
> NFHVEAEYAVDKVLAPEFALPNVNSSQGYPRQGIAMVVYPQLVASAYATI**RALR**EVLNCQLPIEIWFRPDEMRKSPEGLDPLKSLTSDGIVGNLTFEKIDDPRA
> NFRAKAQLVVEKALAPGYALPNTDNSDRSLSQGIAMVVYPKLVASAYATV**RALR**EVQGCRLPIEIWLRPDEMDKEPGALDPLKDLARNKSAGEMTFQAIRHSHA
> -----------------------------------MVVYPKLLASAYATT**RVLR**NVLNCRLPIEIWFHVDEIGENYALLAPLQQLAIFVGDVSFHPMYNPRAKS
> --------------------MLSRGGPFQALTLTNEQQQHCHDCTVQLLD**RTLR**SYDERDGQGEDGRPTTPLRHANLDSSRWKLLKTRSDASLYTERNNSVRRD
> -----------------MPFDGHRGVPFEPLRLTRDQQQQCQDLTFQLLD**RTLR**NYDEREDSVDSHSGAPKLHADLESGRWKQLKTQADASLYVERTCSTWCDL
> AVEALADRLVAETLHASENFASQGHVADPSCWKVIKEKNSIVAYLDISNA**RPLR**RDRFWSEDTVVVTASPQNPTLYPTSGRSFEAFQGQGLAALDDDSEEEQSY
> ------------------MVAIPVQPPHQGANLSEEDQLKFQEMAMQLLD**RTLR**DMDDHDVEPRPHDKQDPRLWKKIHRKDHFTTYTRCDDAPEAAYVAADGGR
> ------------------------MPVDMYINAIGARPPAPIMLLDEEQQ**RILR**DGQHQPTEPDETQQSRPRDGSTSSGLFVPTTPPMLPIPHHIPAQAPPPPT
> ----------------------------MGLNLVSSLQEKTRWVASKLSL**RKLR**SRKRSQKAMEATELRPVALAGASKGFTGASQQCNHFARTPSGAQHEQWTD
> -----------------------------------MAATRSTLSCTWHVQ**RLLR**RSLRSSLPSVDVAALPAASLGVRRSAQSDADYQTSLALQLGSRPSKKKKQ
> AAALTADVPPNQSLYLNNLNDKIKADRMKATLYATLSQHGKILEIVMGRA**RRLR**GQAWVTFDDVPSASNALRAVGGSVLFDKPVVAHFAKEKADVIARREGSFV
> ALRESRCLNSAELLREHWRWEQLFLQATQAMDSRQDDIADQVRVTARSLC**RDLR**ENPVAVEELYHKGTTAHDRSEDLQMLVKALSELTDLTHAQLDKTLEDAKS
> QNLCQREVQFFNQYDQLLTDYMADFELDLSAVRTVLLDDLKPPKDLYIEV**RVLR**DCGEVMTESGLVNLEAHSQHFLRRVDVEQLIRQGLLEQIKR---------
> EKLSREVHQMQLTGAIHTDWLENPKGFLLKPDFKRKAQHVGAGGKKLNSS**RVLR**LHLPGNSYRVRWFVLDGMILRYFKTSTEEQELGAIHLTSVNAVLPSSVAD
> GTGAQGTTNHPKMSYEELLALSMKLTRQNKLMKAQYQKNQSQMATSDADA**RALR**TFLEQELGLDVENSAPGGSIDVEALKEKYRTLSELKHKAETKPVESVNLL
> -----------------------MEDEVYPMTLLTEELKSDEVETRIKAM**RRLR**TVAQALGPERTRSDLLPFLREATEDEDEVLVALAEELGGFVDLVGGDEHA
> ------------MVGGESPTSDSTAANDSRRLHFGGHGAPRDPDESVNTQ**RSLR**TPFVDSTARSDISSATYQLTQSPKNQQIWPPLQEIRRRHLANVQALHGKL
> -------------------MAGDFSATSDEQSQRRRPFIGHGAPREDDSQ**RTLR**SPLLASARVPAARSPASSIASRTHVNSSARSPPSTFLGLRHRHYANIREL
> --------------------MTITKRVQKELVQAVVPADNQALKALRKSS**RALR**AAHKARSAAEKNKGKAWTREEHERFLVALDVFPSGPWKAIAEYVGTKDSR
> GLWSPEEHRLFVDGIKLFPSGPWKDIANHVGTRTARQTMTHAQKYRQKIA**RRLR**NVRMSGKHNLPFLMDQSSRFNALLSSDEQFNDSILATSLAIAAEHELDLL
> TSGEHIRFLEALQLYPHGPWKLVAAYIGTRSTRQAMTHAQKYRQKIERRR**RGLR**TAQAKRPKIQPTILSTPETVEHAPMMSFEQLITAETEETWRQAVDWRLME
> LADHEQRELVLVVRGTASLLDFCTDLCLQNEPFQDGQGHRGMVHAATWLV**RHLR**DDLQELALEYPDYRLVATGHSLGAAVAALSAMQLQDEFPNIHCYAFGTPA
> ---------------------------------------------MNSAL**RRLR**PAATISARHLHALTASAAAESVSPILGQRHKSTAAESASTIAQAEATRGP
> -----MAARVTKLFLQNCASNKVDASKTGKRSASKVKAGENKPSLSKKAR**RKLR**QNSKKVSNKARVLEVARRELEQADRTTENLRKLKSKTSTKTQRLMAKALA
> ------------------------------------------------ME**RKLR**SSRGPAHSHTRHQRRVTPPPLAPPAADPPLDAVADARPTPRRVPLARLLR
> ----------------------MSGVSVLDKTPRVAEETAKYPRAERLGA**RELR**YGTAGFREDAGLLVSTCHRMGMLAVLRSKSAGKIVGVMITASHNAATDNG
> -------------------------------------------------M**RPLR**QVGARLLRPPRTSPRRAAPLFITAESSCIHVTTKIPPSVRFLHAEQAEVE
> --------------------------MASLEAQIKHQVEFYFGDSNFRRD**RFLR**EETKKHEGGFVPFSILFKFKKLAALTVDASVLQAAVADSDVVELNDAKNA
> ------------------------------------------MAVAKRPA**RSLR**TARREQEFDDNDDTAGSGTPLSVDMSFLSNVTSFVDDSGVGHVKVNDTMD
> -------MPLELRGFSADRGRFLLTTARSDAPHVPPRVLERDLVDLQSWD**RLLR**RSSPGLPRLPSKRLRRLMVDNWVRGDRAAGWRLELQKNVSACEGWLNAAF
> RSSHENIVAVLRATNKQHAANVLELRAQVAALTEGNLWLERQLTAKGELV**RELR**EMEKQQQRDNVSVSEVEKILEIKDAQIQTLEREMAQLRQRLARLESDKDS
> PTTSSTWNFVQMNMTLLPIARPLPVSVTHRFQEGAALVGVKPCTPTQKLH**RRLR**QNTSNISPRRHDVDCNSVRAATTTSPTSSRGLPPTPRSACGAKRKHSETW
> ---------------------MDDSATAGDRRGMWSMIKEGYEGLVSTVI**RPLR**AQYAPSELGPKRAQIGDVSVQRVDLKLKNPAGYTLECSWWKPRTPKNGQV
> --------MHHELFLALLGHTGDVVQRRADGFFVRAEASFLSQAQRSVLD**RLLR**LGFAFSALNEFVRRAPRLPSVYLHALAQGVERLLQRYADAVVELEAKTLR
> FERDVQVFVGADGLIQSVQRAAVVDAACVHELPGRALLPGMVNAHSHAFQ**RGLR**GLGETYPKNSTQSSFWTWREEMYKLVGGMSEQQIYDLTRQCFSEMRDAGI
> -------------------------MASAVRSLWPDRKKPRKETPEEARK**RYLR**EILRRELEDKKRVERELHNLVKQINELIQKDRKSLIAYQRRVKGEDPRSV
> ----------------MAPTTRPVYDQDPDALFRGVAAPVTRVRAISSTD**RLLR**PTKASLAGSRAKALTGKEKELAEAREVTRKRRRLARVAKENGRLTIPKSP
> SEQEHEQFLHAMKMFPTGPWRSIAAFIGTRSIKQVQTHAQKYQQKINRRR**RGLR**KQKKKFVRPEHRVDAHATGCIQRVKNFTMQGSPRSPSAFPIGAMSPMSGG
> GIWSQEEHDRFLEAMKLYPKGPWKTIADHIATRSVRQVQTHAQKYQEKVS**RRLR**GLRKSKKKLVRPEHRIDEDTMELCKLVDCAAEKLVAASRKSSSRASGVKL
> AADAKLKQNERERQEIERLNARIAEQTPARGSQLGDAASFDLFALSEASR**RGLR**TCGFTAPTKIQVGALPHALAGRDVLAAAKTGSGKTLAFLLPVLEKLFRLR
> QSVTSHSQQHRGTPVLEDQNYMSAFSPSNNGVYVMETPQQRDGDSAPDGR**RSLR**QLPPVDQNGSFRQRHGVTRDASGGFVTMDNNSTISSSSGNYIDAMSLNNN
> VQPLLVHEVIVISSDDESDGDEDEDTIETNRSGPLHWSEVIDRLLLLAEH**RDLR**RDGSILTKLLQDLGIPQSRGVKLAMHGVCNKLAKLLAFCRDHPRRLRACE
> --------MTTLKILWREKQDMRRVQSAVHQALSVLDGRAIANWNELSF**RDLR**DYVLLVFPELQEPPRELLLYYMDDDDEQVRITNDAELDEAFRLMRELAVA
> NQVVPSNGISSTPPNGVSSTNGSSANASEAADTFPAPLKPNPKPQLRAPP**RRLR**SGSGSGSNTSSSGVRRESGTRRPASANMGIVVGTLRRRNRVTLRTSSLSD
> ------------MLMTTSSTVSLPSVRQPEPPLGRAAADQRRRSVPKITG**RKLR**TPRKFVVTDVPEDELDVLTPSASLSAMSDASEQPRDDDNNNCEDLSASSS
> ------------------MSYLSTSTHFNNWVFSAEELARVRRLQHVKTK**RALR**LERGETQKVEAAAASSRKARSFSALIPRSSTAVRDAFDWNDEVNMDLSTE
> QVRIIGKGSFGVVTQIVRKADRKTLVWKEVNYGAMSEKEKQLIVSEVNIL**RELR**HPHIVRYLDRVIDKQATKIYIVMEYCEGGDLGQFIKRKKREGSYIEEGFI
> -------------------MRAQWEGDLRVFLEEDSEYVHFLEIFEQDLV**RKLR**FKRRARDGPLVDSVAFDRFVETAAQLRYFHVLFQSKLKVVLKPQGSIEGK
> ESDDGTYSDKVHSMMHQKTTRLVVDLNDLREYDSSFMDPAVMGAQDNIVN**RLLR**NPAEFVPPFEEAVKELVFNMDSTYGSKEAEATKDMQFHVGFEGDFGHHNV
> PATPLNVLEFEEYAREYLPKSTLDYFASGSDSMETLKENREAFKRLVLHP**RVLR**DVSSMDTSTKLLGHRIRSPVCVAPSSTHRMAHPDGEIASSSAAAKADTCF
> RKVTSVAKPKRLKAHVQRAIKAKEPKLVENTKNLLVLRGNKTNEEVNELL**RDLR**MIKAPFAKFMGKKNDIHVFDDENKVEFLTQKNDASLFLIGSSTKKRPNNL
> EHVMKIQYDNFLKGDHIYDLLVDLLGDSILIVEGDEWKFHRRVFVNLFSS**RALR**EHMAPIIQKHVRVLQGVLSNAAQSKQAIDFFTYSGRFTLDAFGEIAFGFN
> --------------------------------------------MGDKYD**RQLR**LWGAEGQRRLANTHVLLVGSCATGSEALKNLVLPGVQRFTVLDDQNVTLA
> DGDIVDFVMNMANLYAFPDNSVDAIYGSHVLEHCTHGVGHELEQTLREWH**RVLR**PGGQLFVSVPNLFVLATLFINESIPHQHRMWFMTVIFGGQIDVYDVHKVG
> ----------------------------MISIDCMSDLRIVLLAEKLQYS**RALR**YALRCRCNLKTIIQKKLEATYEKMEKNLFTVRKTSGNKPLTLAEKIIYGH
> -------------------MVIRPDDWARIIFSDEKKWNLDGPDGLQNYW**RDLR**QPPRQTNRRQMCGGSVMVCGGFSTTGKTKLAVLVGRQASEHYTYTVSDYL
> DGVGAKMDSMELEREKGITIQSAATYCSWKDSNINIIDTPGHVDFTIEVE**RALR**VLDGGVLVLCGVSGVQSQSLTVDKQMKRYGVPRIAFINKLDRMGANPWKV
> SVRFADEKPADEKPADEAAAKPEEPKELSESEALQKQVEELTAKNQDMND**RLLR**ALADAENVRRISRVDVSNAREFAISKFAKALLDVSDNLKRAHESIDVEAL
> -----MEWLDIAKDLNAGTIGGVAGIVAGHPLDTVKVQLQTSREASAGVL**RTLR**RVVGSEGTAGLYRGLLSPILSNAPINAAVFGVQGQVVRVLQRDGPLSSTQ
> DKARRLGYKAKQGFVIVRVAVRRGGRKRPNSKGIVYGKPKHHGINELKFE**RNLR**SVAEERAGNKFTNLRVLNSYWVNQDATYKYFEIIMVDHSHKVIRRDPRIN
> QLNVRTGHYTPIACSSSPLKRNLHDYVRYGVINLDKPANPSSHEVVAWIR**RILR**VDKTGHSGTLDPKVTGCLIVCVDRATRLVKAQQGAGKEYVAVVRLHSAIE
> EMLDRVGDGAFGEVSRARSVKTHEIVAVKKIKALFPTWEECLQLRELKSL**RVLR**HENIVLLKEVIRDKEELYFVFEYMQTSLFRVMRNFKTLGKAQIRSMMYQL
> ----------------------------------MDMYTFCTALGKEEAN**RQLR**IHWAIWVTEDDLKEMAGAGVNSLRVPVGDWMFNPYEPYIGCTDGAVKELD
> -------------------------MRLSPREEDHLMLHTAGALAQKRLA**RGLR**LNYTESVALLATQVLEFIRDGKTVAELMTLGAQMLGRRQVMEGVASILDE
> TAMGRPSTPCGLRGDHTAWALQVAPSAIEFSAFERGSCYVATLSVRNASD**RVLR**FRLTPPPHASPFRVLLRGRDLARTENATVSLPPGLSAKYEVAFQVAGVDS
> -------------------------------------MPRKGVSLQEKRE**RILR**IYHESKEVFNLKEVEKLGSKAGVVLQTVKDVNQALVDDVLVDCDKIGSGN
> YRRFVAAPLRLKPRKPSPSPPSTPPACTQQYDASSPWGDSPAFSTRSKEV**RKLR**RRVAKIKISTSDAAIPRDADGETPVNKASDMVSSAGVSSADAADLKSAAE
> --------------MASEYRNCMFGGGHSDDERDAPRPPPAELQSRADAD**RVLR**SFGPSFASAALAFLSEAVDSSVETRPEQLQVLERIVDRAAREAQAGRRQR
> SVSGAENPAFVHDADRCECVGVCSVARVLENCVLQGGVHHTHMHVLTYLG**RPLR**SDCCCYPVGVQWFRAVAEQDDFQVIPGACDEWYTPTADDIGARILAKVMI
> VSAPPLETKKRSGSDIFESSRLAIIKGFQGVGLDRVVDKMQQAFTPDDSE**RVLR**AEWLVVETEEQQLQQLQDAVHSLQQAFKFSNRLRTLVKLANSTLAKCGEL
> -----------------------------------MDLSMILSPALEEEE**RVLR**PPHAPPREPSRKWSAEELQCAVAILQDFQQPLSEPAFDVRGRDGVESLRY
> PLRLDALTSSLDDIGTRREFASILTHFPPDQLASRVFRTSMYLRLVLGEH**RRLR**AQIERGQEAAIKDALEFKRINGDLRREWKFVCAHWHQEVTSALAKANEIK
> -------------------------------------------MADERLD**RKLR**RGEVPPSWGNLDDHTIVYPDSETESVVDDKELDPDYAPPAQEVEDDDDSE
> ------------------MAPHESPRNGYAVVQTPPAVPHDDGFLLNIEE**RSLR**PTWIVYSSVAIAMLEAFHYGWSNSQVNLSTFSNTDDCNARPVEDGTCLMF
> ---------------------------MNLFGRKSAAPARKGPVATAETI**RKLR**EQLESLEKREQHIVKKIALQLQEAKHKSATKDKRGAIFALKRKKMYEAEV
> GGPSNFSGDTGAYAAQLPNGQHFNGHHPSPLQALASAVALPNVFTDAEML**RTLR**RNNGDVMKAALQLTDKHAFRKEMHYQELNVGLPTLQEELRKGYAHLLDGA
> SGEATAASEDSVGRRIHQRANYSVRKKRELIALAQVAGVREVCRMEGVPR**RTLR**HWLDDAEKINSFEGPDSRTSIGRSGRREILPFGREMSDFLEEGQREGREL
> GMWTEEEHARFLEGVKIFASGPWKRVAAYVGTRNVRQTMTHAQKYRLKAA**RRLR**EAQRKQAAARHGLHNAHRGGLDSPLMRRSLQTPTSGGLGLGACSKMRLPC
> SPRAAASSAAASSGPQRRRHSSGGPLKGVVAMVDVRVGADAQIDCSDVVA**RKLR**ELGASTVKRLTPKLTHLVLSHFTPVWKTKIAKWQTGGGSMAAAATRYELK
> ---------------------------------MAEEVLSEERKLILQNR**RELR**KAKKAKAKQFKQEKQQARREKKAEIVAKIRAKREEAQYGADNSQSQDGCW
> PKLMEVLTEACGRLIREGKPPSRLFSVSLLGAVKENPKRFGQPAVLKYLL**RLLR**SKHYIGIKDPSDHGKKPSTVTALGSGHLSASGSTEYFQLSLPVLVTNLLV
> MFNKGAVTNAEPPAWKRRPVKLPQDNIKAEVKSQAATTSSETLVDAAVQE**RKLR**EQQERRNAQDTRDAMAKQSSYDLEHDRVYHHAVKQKEAERSAELKAKESL
> EKHEGKSPLGVEEWAFVQSVQGDEAPDSLQLDAVENDRQMARNQQLTDEN**RKLR**IESRRLKEELEQLAFTKQQEEKETGARISKLEYENKQAGHHEASAQKKIQ
> ------------------------------------------MNLSQREV**RVLR**YTLSHLTYSNRPDLADVPRASSSPPSAEKKRRLSRNDGTVHDDTLRIVAE
> MAAIVEDPPLELTQRTRAMYRELKPRALYDLSGAKSPGSLEEMTGYFRRF**RALR**GKSLTGVSHDALQRSWCAMIVRWNRMHQEGSNFVAWLTSREEVVGEHSLS
> ------------------------------------MAERVRQCPRGKRT**RVLR**HLYFTSGRHYWEVLVERVADPRSVFVGVAPSEKFETGATEQTVGYAATGE
> --------MVSLYVNEAQDDWDDFLPSALYAYNGSRHATHGYQPNELMMG**RKLR**TPAELLRRNRLEHPHSTLQDYHEVLLQDLRKARELAAVALQKEQARQAMY

> ---MDRTSVRVDVTPHSTIEFVGARTVDVVQSTTPDSFRASVFLCASATG**RKLR**SMVAVAGAPGTIVHDELKEDMHYDWDRGYYTVQAKAYCDKRVMIEWIDTV
> EKHEGKSPLGVEEWAFVQSVQGDEAPDSLQLDAVENDRQMARNQQLTDEN**RKLR**IESRRLKEELEQLAFTKQQEEKETGARISKLEYENKQAGHHEASAQKKIQ
> DETELLPAHILENMLAQMLDQPELLKDLADERKLLHREDQREEQLLKNFK**RTLR**ARVRVKKHLEELKHAADENVVVFNCKHSLIQSVVLGYEEKGYVEEDGEED
> HYCELVVADTADSTVVVEALLAWHSRFGIPPVWVSDNGTHFKNEVVAELS**RRLR**TQQTFTPAYSPWVNGSIERVNRDILQVVRAMILSYKISYKDWVYLVPIIQ
> ---MGAGASTKALDEFEAALESSDSTSKLVVTSCDLLVLPPEILERAAHL**RELR**LEHAKLKQLPASFGCLVLLERLSLAGNELEALPLSFHQLQRLEELDLSCN
> ----MAAPSFYEVLGVAATCSADDVRRAYHQAARRCHPDKRSAATEEDEQ**RFLR**VQEAYETLRTEEQRRQYDAKLLQDELVRRREQELVMVSDEVPLADMKREI
> -----------------------------MSAHTAATASVNGFQLEESNK**RFLR**SYGMSDLDVKEDSSEEERGIDLTKLDDVINVVKGDKVIDAAKVDDVLNAA
> --------------------RLYAEVQQREKIEVFCTQATSDSNQAMDAL**RQLR**QDHVALTRHHNAANAALGHHADIVAGLSARAKAAEASAAASQRLIQKDRE
> RLSPDDGTMRDDTLRIVAEALRSENRQLLQDRDVLKAEKKALDERLAQAQ**RSLR**VLEDDMGRVYRQKQKDMETQRQECEQKLQSQNTILGRPAEREGSKELRAL
> TFLKNIDHIEISDTKTVDGVVFYVLDVYVRHQLSRIPTNNKQHLEQKPDF**RVLR**RFSEFETLRERVTAASQQQLMATCPYCDSIRVFMFACYRRPTAFIKLCTG
> EALLLDSHQLLAETEELLASCNTPKAKVASQKSQISAEERREIRNAQAAK**RRLR**YRQKLKDEKEILFLQESELSGELSKLQAAEAEKKRKQAGNLALGAWKAVA
> ----------------------MARIRSSQRRKSATAAAVARDLDFPHFW**RQLR**AAGWTYKKPTGLATEGRYATPDGNGVFVGETAVVAYALEYGLMDEVGDVA
> ------------------------MVRIRASRRTAPAAAATHDVDFPHLW**RQLR**AAGWTAKRPSGLANDWTYTSPDGSSHFIGEAAVVAHALTSGLLNENAQDD
> REAIVLKVDADPGVAKPLDLDSSDIISSTQMLKRLVDRWGNAIVEGGALW**RKLR**TFTLVPGEHEAPTRASTFNRAMQEAVRQAFAAAHSTEHAPREELACAVSE
> ---------------------MWHCFGRSSDLGYIRKQHVSVSADGTFYL**RLLR**VKTSEEQGLTLVPDKDDFLTCPLHALAIALATQDAPCAALLDQLPELVSE
> ---------------MSTPLKALIQERLRAKMVRQGPDYDLDDEYACDSD**RLLR**QPVEVTAYDISSRGDDEDVLVSSSSRGPITRRSNPTQPTYPVRLHTHIDP
> -----------------------------MSAHTAATASVNGFQLEESNK**RFLR**SYGMSDLDVKEDSSEEERGIDLTKLDDVINVVKGDKVIDAAKVDDVLNAA
> ----------------------------------------MYLRLVLGEH**RRLR**AQIERGQEAAIKDALEFKRINGDLRREWKFVCAHWHQEVTSALAKANEIK
> -------------MSSPTAGGGVPEKTVLVKHEDQLAVMARLVLSEKQRV**RELR**RMRQIRYRKKKDNYALSLEEEARRLREEIEKLAQEQRAAYVAVSAVESVW
> ------------MKRREVADAEGEVSEAKDRAQARPQELDEVENRHETCL**RQLR**EADNETAKIQHRMNSLRGQKRDSLAVYGSKIPQLHHLIQQNRHRFAAPPI
> AVPPPWIEIGSIPVDARLVVWVDSNSELTLDSLPLEGSSTLSPANTADST**RPLR**FWRERAATVENIRAQGDASTTKVQSRTSSVAMQTPTVKSLPRVRVLSLRS
> QDVQTLKMKVARNHDNDMERRRSSTLRLEPPQSDHRKSTSFVRARTSGVN**RLLR**SSTSSVGGLSNGHSRSSRNRSRGESRSAPTSQAPSENFETALAEPVDEHA
> EALLLDSHQLLAETEELLASCNTPKAKVASQKSQISAEERREIRNAQAAK**RRLR**YRQKLKDEKEILFLQESELSGELSKLQAAEAEKKRKQAGNLALGAWKAVA
> ----------------------------------------------MALD**RELR**RLLEYANLPETENSSSKDVRPTARGILDRLIGIYHQTCLPSMGVSADMNL
> -------------MSSPTAGGGVPEKTVLVKHEDQLAVMARLVLSEKQRV**RELR**RMRQIRYRKKKDNYALSLEEEARRLREEIEKLAQERRAAYVVVSAGESVW
> AFVPFGPVKSVQIPTEYSTQRSKGFGFVEFEDEADARAAIDNMDDSELFG**RTLR**VSIAKPDKPKLGSNKPVWAEADEVMDDAEEPSQDTHGSRPQA--------
> KKRKSTNQVRREEAEALREQIRALQDQVQELNARAEAAAMTPSDHLHLLR**RSLR**TKNALQELIHDQKLVLAGAQSELIDYQVHHQRLCHGWKRWTPS-------
> ----------------------------------------MYLRLVLGEH**RRLR**AQIERGQEAAIKDALEFKRINGDLRREWKFVCAHWHQEVTSALAKANEIK
> ADLGLALWERRHWVQVAAVETYLRSEAQRLGVTDPSVVALRAAWTEYNKA**RNLR**ADRIRQQMLYRCWEWCIERTGKPRESITEFLLEPTYLQYSFEVIEWAPAS
> -------------------------------------MKRGRVATRTSTR**RVLR**RKNVVVDEEDEDGAEDVSQEEEEEEEEEEEEEEKTPPKKLTRAQAKKQEE
> -----------------EDLFWRWVGLRGHSAQELDELKEDRLLHRILDQ**RDLR**IRFAHLVSKRKLYSEMEGLKRRAQSGARNERGYGFHASATVPRPSAKKPR
> -------------------------------MAIDTYYNKARKQQAAAFP**RRLR**TSKQQAAALSQHNEKLAAELQTSKQHVAALSEHSEQVRVQLEEAKTKAMQ
> EELQDTPKKASGRMGNMNAHAPCCTEVKYAILNTMDHSWRSASLQQKFCD**RALR**LSDTSDGDLGVIKDTVVVTHSMGGLVMSMALATGKCSFGEGASWVALSSP
> SDVAPASNWVDLRPLSSSSRCVSQSGPRPGLYTARHRLTQELVALKVLDR**RLLR**QRATRRRLRQEVRVLQLSRDHQNLLTLYEVKAVGDRVELAFELARGGDVL
> -----------MAATFVPTPSLSAGEPSPSSSSTGGDARAAPISASRFAQ**RRLR**VVTEPKHSNKMHRTRTLSELILLSPRSSPRITPRHKRFDMDIEVSSFFAE
> --------------------LEEGEEVDYGSDTCLREEEEEPPRGRTTDK**RDLR**TPNPFVERSDAVSALTALRTTSSLPSRDEDEPMEANVVSNNLDEVQSQQL
> ALSRAPIHTVTAARSGIEREEIAGDLPRQGSEGQLTDETIRARQADDKTV**RKLR**REKKYGGRAIVVKDELFHIRESDGSLRIVLPTALWPDVLRENHDSIFACH
> --------------------------------------MATTEGASRCGR**RILR**GEICLRKSKSPHSPPTFCVRKPNGKWRMVHAFNKLTAATIPASTPIPRKD
> FERMLEEEDVSVEFLHASFLLDTSGKRLAVTMDRFGFYLATNEADGTFYL**RLLR**VKTPEEQGLTLDPDKDDFLTCPLHALAIALATQDSPCAALLDQLPELVSE
> --MKTDKLKGVPSKKKGDFDEVDSRIQIPVATRMKMQMNRKMNASGGEDT**RLLR**TGIDQYRSLQHLNTSTGRRSSSSSASVDRSVERGTSLMERKQFGAAVPYF
> -------------------------------------------MATLALA**RSLR**ACTRRSAPLSSRAFASVPDAAPKFSVVDHPVTADAIKPEDYFAVVKLAGT
> ----------------MPSSLSVDAAAVEDALLRGEWTQALNDSRSMLAA**RPLR**QEDASAMTTDEERVLSVYLQAVFELDLDDEVETAATIARAFAPLPSEIAI
> AWPSPSSSSSSVSSSSFGVHVPPGLRHFEGRFQNLRGQSLFYFSLYPPPK**RALR**GVVLYLHGAGDHCRRYVPLYERLCEEGFGVITYDLVNHGASDCDAHKTRG
> SSADAPKPRIRRANETLFEGLEEGEEVDYGSDTCLREEEEDPPRGRTTDK**RDLR**TPNPFVERSDAVSALTALRTTSSLPSRDEDEPMEANVVSNNLDEVQSQQL
> ADLGLALWERRHWVQVAAVETYLRSEAQRLGVTDPSVVALRAAWTEYNKA**RNLR**ADRIRQQMLYRCWEWCIERTGKPRESITEFLLEPTYLQYSFEVIEWAPAS
> -------------------------------------MDAAAKKRHKEFA**RMLR**QKEAKHARVKHLPLFREGSPLFRRFGGKSLGDYDQLPRTSFDGPIAVIHS
> -MFDSSFCASVAVVSRREACFRKAWDTNTLVLSASPLLHPVSHAYEQQDA**RCLR**LLQAVALVPSCKSRKLLGTVRQALKMPLVSKTRSQLETSLCPLLKKLQEE
> MAAIVEDPPLELTQRTRAMDRELKPWALYDLSGAKSPGSLEEMTGYFRRF**RALR**GKSLTGVSHDALQRSWCAMIVRWNRMHQEGSNFVAWLTSREEVVGEHSLS
> --------MPERFGLIIDGRIHDSEHFIAVFPCHLLFYELVELLPSVASK**RRLR**QLLGEQKDVESVSKELRGDNVNLLDFRVWFEGLIALKPPYEHYLKASPEE
> KRQLTSTLNQLVQQKSVAVLYTPTEVERITQMTDMMAQATSDYEADEDWD**RILR**VTDALSSVSNRAVLKESIRYLKLRLGDPSTRVVILALTLTESIVKNCGDL
> DGYYQILMRERDIPLTAVSTPNGMLWEWLVMTQGLSNAPATFNRLVTQLF**RPLR**AFTQTYFDDNFVHSRAEEGKTAIEVHLEHLRRVFMVMRANKLYANIDKCV
> ----------------------MARIRSSQRRKSATAAAVARDLDFPHFW**RQLR**AAGWTYKKPTGLATEGRYATPDGNGVFVGETAVVAYALEYGLMDEVGDVA
> KKRKSTNQVRREEAEALREQIRALQDQVQELNARAEAAAMTPSDHLHLLW**RSLR**TKNALQELIHDQKLVLAGAQSELIDYQCWVTRRQLLVDMKDDKLNSALPY
> PQTQSTRAAAPSKHKEGKGDTELRRQRSRTGMQRYRKKIEDHAIALERDV**RKLR**GETQTLQLQCQTLSSGVPTGTTALSVVAEYFRLFRYGLKGSALMNDPDNS
> ---------------------------------------MTHGRVSRAVA**RLLR**PFHRHQKEDDASAKRPRGSLTSTTTSSSTRSTSSSSSRSSGVFRSSGGVS
> ---------------------------MWRQELRWMRHVVRAQDSELRLD**RWLR**RQFPALPQSFLQTQLRKRRIRLQPPPSIDAQPRLQAAQAKSLLREGCVVA
> --------------------------------------MSTSMSRPLDFR**RILR**MKTSMMLRWTWTALDLDDNDGPRQTSFKQAGKRKSRPSGDDDTARRSRPV
> -----------------------------------------MLSSLCRST**RVLR**ASSSAQSLLKAPATASQLQRRTFLTSPRVTSYVMARAKVLHLAARSFLAY
> ---MGAGASTKALGEFEAALASSDSTSKLVVTSCDLLVLPPEILERAAHL**RELR**LEHAKLKQLPASFGCLVLLERLSLAGNELEALPLSFHQLQRLEELDLSCN
> CGFCSTGAMSTSSSTFSGLMVTSASADVAAQKLVAASSMSSSSTSSFRIE**RRLR**VVCSAAVPTGASLRFLRPASMEKSRGEAVLLATLPLELPLAIGDAIAIAS
> ----------------MVATLQQEFQALRVDREQERETAKNLQKFLAGQV**RELR**TASAQQPSGSVSAQEVGSAPMVRPDSGRTTTRSDRSSASGNAADATRQKP
> ETNNTAEYTALLLGTRAAADHGTTTLRVEGDSTLVIQQVRGIFATRSVTL**RHLR**DQVKLELARVGRFSLHHIDRQANAHADRLANRALDLRRTVSECGVHPDGN
> IISLRDEIARLQAQCADSEDRLHAEVQQREKIEVFCTQATSDSNQAMDTM**RQLR**LDHVALTRHHNAANAALGHHADIVADLSVRAKAAETSAAAAQRLIRKDRE
> -------------MPSTKPKKNDISSAKEFKTLCIAARKAYKIRVSDEAI**RHLR**LTMNTTVASASLLPETIDVIFACVAERKKRCVGIDDVREWLTAPGPGGDD
> ADLGLALWERRHWVQVAAVETYLWSEAQRLGVTDPSVVALRAAWTEYNKA**RNLR**ADRIRQQMMYRCWEWCIERTGKPRESITEFLLEPTYLQYSFEVIKWAPAS
> EGEYQKDGVEHKQEVEPGFTFFVGGEEFSHPNSVFAGQSFPDVEAFENFL**RELR**VRKQRRLQEKREQLDKEAQHAQQDHTEEYQLQQQQEAGQVAEEVSNKSDD
> ----------------------------------------------MASK**RLLR**STGTIIDKDDSVDSEERAITIPGLDQLKSLVKKGSSKVDKLIELLKYKFW
> -------------------------------------------MAKAVQQ**RVLR**PALLKDEVLLAQLAAARTERSEPDYDDFLDEVKHAVCGFVQKRVLVPLEV
> ----------------------------------------------MDAL**RQLR**QDHVALTRHHNAANAALGHHADIVAGLSARAKAAEASAAASQRLIQKDRE
> STDREVIDASAVLADSQLKQTGKRTHRDETQSEEERVEQEAAKAEPKKAR**RGLR**EASERRRPAYLDDYVVNAVQATSRVLDRNRKPIRGSDVHIPRNRREMLRS
> RGRRDYHQREVVVIQDEETTDQDSSQSDDDNEEEEEEEAARDEDFGPGNK**RHLR**TQSLEEVTEVPSAKRGRNADHVQVGDGIQRRSQVEGGVRLGV--------
> ADLGLALWERRHWVQVAAVETFLRSEAQRLGATDPSVVALRAAWTEYNKA**RNLR**ADRIRQQMLYRCWEWCIERTGKPRESITEFLLEPTYLQYSFEVIEWAPAS
> ----------------------------------MSGAADNMKKWEAKEG**RFLR**NFSTHNAIINSISINQDGVMASCGDNGSMRFWDYQTGYCFQNDSTKAQPG
> ----------------------------RLGVTDPSVVALRAAWTEYNKA**RNLR**ADRIRQQMLYRCWEWCIERTGKPRESITEFLLEPTYLQYSFEVIEWAPAS
> ------------MIGDFEYVQQRVALQVDVMRRHVTGVAEVVLAPRAAEL**RVLR**LHARQLRVRTVHIDGVQANFEQLNFLGEVVDESYRDLATFDLFYRGAIVA
> ------------------------------MEALVLKSLGKLRKACTRSH**RELR**DMIDAAQAKVTAAGQDGAAEVPFEPFLCACLTRHAKLVAVALDCMEKFLA
> SSADAPKPRIRRANETLFEGLEEGEEVDYGSDTCLREEEEDPPRGRTTDK**RDLR**TPNPFVERSDAVSALTALRTTSSLPSRDEDEPMEANVVSNNLDEVQSQQL
> NSHGVSSALAKFYPEAKGSSLETKRKSFYLWRKRRDTIEKAGKSSSSSVL**RKLR**PAGSATVLSSQTESQLLRWVNSYRADGAPVSTLMLQLKAQEYAAAAGIPR
> ------------------MLPPRGRHSEVFWTRVFLFGASCVAGLIIARR**RKLR**RFHLHILNAKSADCGRLFGLDIGGTLAKMVYFQSADGAAAVQRRQMERQL
> ----------------MYLMVATRPDLAAAVGVLSQFSADLCPTHWQALK**RVLR**YLQATPTHGLEFSREDSLGMCGYSDADWAGDIESRRSTSGYACMMNGGCI
> ------------------------------------------MSGFQARW**RELR**KDGWTWKRSEGLSNDVIYIKPGKTKKDVRGVDVFVGEKEIMEYLDNVDLE
> ----------------------------------------------MDAL**RQLR**QDHVTLTRHHNAANAALGHHADIVAGLSARAKAAETSAAAAQRLIRKDRE
> -------------------ESCADSVAGNALLRGVSGGERKRVTVGEVLC**RDLR**HHQGTSHVLGGSVIVALLQPTPKLVEQFDDILMVNEGHMYFEERGFVCPP
> SDAQSIPLSPSRLESPMFRERVNALMQQGDTYAKRLDGERRRSHDLDLAL**RTLR**VEHFQTRKALSDATNAQLCAATTELKPLRTLENRLDKVLTRYNE------
> --------MELLPADTLKLLSSFLTGHDAFNLSHTSSWGFKHVGDGFAWQ**RRLR**SRGSWQKRFMVAPSLLFQGRQAGNNLPLDSFAYLLHSKDQDAFCLTSLRH
> -------------------------------MPGAASSRFHAQLVMTDDK**RRLR**RSKRHGELVLGAANQSVQLVYPRLGRYFQRKFEQPLKCVLGRQLLRVYSS
> VRFGSSSSATESDGPVGAAHAPRGVEVKKARRLWEPTLQTVRTVANVPHR**RCLR**ARVQPGCTTKRCAIKPGLNACTRGKNPPASTRNNCPRPGLDKKKLAEPGS
> AVEALADRLVAETLHASENFASQGHVADPSCWKVIKEKNSIVAYLDISNA**RPLR**RDRFWSEDTVVVTASPQNPTLYPTSGRSFEAFQGQGLAALDDDSEEEQSY
> IELDDITSEKQLKQREQGQTKDHSAHSDAVLYKIDVPANRYDLLCVEGIA**RALR**IFLEKERPPVYTLAPRAEGPHHITVKPNTKLVRPYVVSAVLRDVQFTQER
> ----------------------------------------LPKLKNDLLL**RALR**GDRTERVPVWCMRQAGRHLPEFRELRDLGYDFFTMCGVPELAVEVSLQPL
> ADVAMTSADDSAGLTSYYEQKIEHLELTVRDETQNLRRLQAQRNELNSKV**RLLR**EELQLLQEPGSYVGEVVKQMGKSKVLVKVNPEGKYVVDVDKTIDITKCTP
> ----------------------------------MDIYTFCTALGKEEAN**RQLR**IHWATWVAEADIKELAEAGVNSLRVPVGDWMFNPYEPYIGCTDGAVKELD
> ----------------------------------------------CIGT**RNLR**DNLSDLRAQVAANQRGVVLMHELCAEYSLPVVTAYMDYIQQAAEIAVRNM
> --------------MSGKGKAATGGRGKKTGKSSTRSSKAGLQFPVGRVA**RYLR**KGRYAQRTGSGAPVYMAAVLEYLCAEILELAGNAARDHKKTRIIPRHIQM
> --------------------------------------------PGTLTG**RPLR**LYADGIFDLFHFGHAKALQQCKEAYPNTFLIVGCCSDEITHKLKGRTVMT
> VAQLKEAGDESFKADASTPYAELWMGTHPNGPSRVIREEGGESPLLSDWI**RSLR**ANETGDLPYLFKVLSVRKALSIQAHPDIKLARELHAKFPEMYKDPNHKPE
> -------------------------------KNRAPAGYAYIEPVMDALE**RELR**EKMNEPHEGKRQCEALWPVHQINWQRSRYVFDMFHKYHKIPREVYDYCVK
> LTINFKGKTLLSDTALKIVAGRRYGLVGKNGAGKTTLLRYMSHYELEGFP**RHLR**LQLVEQESASKLSKDERSVLAVVLAADYERQMLLDEEKELSADAGGDHSA
> -----------------------------------------------MSF**RELR**NLTEMMRALGYPRPISMENFRTPNFELVSDLLYWMVKKYDPSSSVTEEID
> NVLGRTGNTGNVTQVRVEFLQDDKRKIIRNVKGPVREGDILCLLEWEREA**RRLR**--------------------------------------------------
> ------------------------------------FRKILVANRGEIAL**RVLR**SAQKLNMETVAIYSDADAHAQHVQLATEAYRLGPAPASESYLNFPKILEI
> AFMPLVPGVHYAEFNDIESVKKLISDKTAGVFIEPIQGEGGLYPADPEFM**RELR**ALCDKHDALLITDEVQCGLGRTGALFAHELYDVTPDIMTLAKPLAGGLPI
> ------------------------------------------LVGNKSDL**RHLR**AVSTEEAMAFAEKNNLAFIETSALEATGVDTAFQRILTEIYKLMSRKTIQ
> STFKIPLKIDGESTCNINKRSQVTELMKKASLIVWDEAPMAHRHAFEAVD**RTLR**DVLDNEAEPFGGKVVVLSGDFRQILPVVKGGSATETIDACLKSSELWPLF
> IARLELENFKSYGGVHVVGPFHRFTAVVGPNGSGKSNLMDAISFVLGVHS**RQLR**SNQLKDLIHKVPGDAPDSGRSAFVTLVYELKKEVKFTRIISDKGVGSYRI
> --------------------------------------------HWQALK**RVLR**YLQATPTHGLEFSREDSLGMCGYSDADWAGDIESRRSTSGYAFMMNGGCI
> --------------------------------------------HWQALK**RVLR**YLQATPTHGLEFSREDSLGMCGYSDADWAGDIESRRSTSGYAFMMNGGCI
> -----------------------------------------------FVC**RLLR**GLHGLRQTPNVWNRTLHTALQQLELLRLDSDYGLYTQQVGDTGEISHILT
> ----------------------TRPDLAAAVGVLSQFSADPCPTHWQALK**RVLR**YLQATPTHGLEFSREDSLGMCGYSDADWAGDIESRRSTSGYAFMMNGGCI
> IGKGAFGKVMLVRKKIPDGTCNPNAIYAMKVLKKASVFAKNQVEHTKSER**RILR**DIDHPFVVRLRYAFQNEDKLYLVMDYYNGGSLFFHLRKSRKFSEKRARFY
> ----------MLRELFLAYYDNTKRKPEHVIYYRDGVSEGQMFDILQTEM**RALR**KAFKMISEDYNPPVTFVVVNKRHHLRAFPVNQRDADRKGNVMPGTVIDTG
> --------------------------------------------HQQALK**RVLR**YLQATPTHGLEFSREDSLGMCGYSDADWAGDIESRRSTSGYAFMMNGGCI
> EKDPVRGNDSFRSFREILQLAQRERVDFLLLGGDLFHENKPSRRTLYETM**RLLR**THCMGDGAVNFQVVSDQSINFPNFGAVNFEDPNYNVELPIFSIHGNHDDP
> -----------------------------------------SRRAVVWFR**RDLR**LHDNLALDAAIRMQQQTLKAGEGEMALLPIYIVHRPVKQRCGPVRFQFLL

> RYLLKPTALELFIKSTRKNYFFNLRSKDVVQFHEALMARRPLLLKRDPTV**RRLR**HPSSLFRNSPMSNRWVQNEISTFEYLMWLNTIAGRTYNDLTQYPVFPWII
> RYLLKPTALELFIKSTRKNYFFNLRSKDVVQFHEALMARRPLLLKRDPTV**RRLR**HPSSLFRNSPMSNRWVQNEISTFEYLMWLNTIAGRTYNDLTQYPVFPWII
> VFEVRSHYVNLRPVGGGSYGIVCSAEDTLRGRKVAIKKITDVFDDLTDAK**RILR**EMKLLRHLGVHENIINILDVVLIPPNVMDFHDIYIVTDLMESDLERIISS
> --------------------------------------------HWQALK**RVLR**YLQATPTHGLEFSREDSLGMCGYSDADWAGDIESRRSTSGYAFMMNGGCI
> FYAIKTMRVGFDKVSGYRGPGGEMTEKDWLHRCLFLESVAGVPGMVGGML**RHLR**SLRRMKRDYGWIHTLLEEAENERMHLLIFMNLKQPGWLLRALVVGAQGVF
> -------------------------ASIDEPLDLIKLSVAERIYVKCRGD**RELR**GVLQAYDQHLNMVLSDVEETITVQELDPETYEELIKQSKRTIEMLFVRGD
> ----------------------------------------------MINR**RALR**TDEVKMFVLDEADEMLSRGFKDQIYEVFRFLPEKVQVALFSATMPLDVLE
> ---------------------------------------------IPNTF**RELR**ACMTCSLIKTFTQFYDTGCENCAFLQMADNRQRVAECTSAYFEGMIAMMQ
> --------------------------------------------HWQALK**RVLR**YLQATPTHGLEFSREDSLGMCGYSDADWAGDIESRRSTSGYAFMMNGGCI
> STFKIPLKIDGESTCNMNKRSQVTELMRKASLIVWDEAPMAHRHAFEAVD**RTLR**DVLDNEAEPFGGKVVVLSGDFRQILPVVKGGSATETIDACLKSSELWPLF
> LGVIPARLGSTRFPGKPLAEFFGKTMIEHTYAAAVGSKLLSKVVVASDSE**RVLR**VIEGVGGDTVLTGACNTGTDRIVQALHLMDPKELSEYDVVVNIQGDEPGV
> GLVRALNARFDAAKDPQIQGDPYSTLFVGRLHFDTTEETLRGFFEAYGPI**RRLR**LVRDKSDKSKGYAFVEFEHERHFERAYRHAHGRVIDGATILVDFERGRVM
> -------------------------------PQQKGTLRVVCISDTHAKH**RNLR**NVPEGDVLLHCGDFTNRGTHNEVRDFNNWLGALPHKHKVVIAGNHDVCMD
> -------------------RSQVTELMRKASLIVWDEAPMAHRHAFEAVD**RTLR**DVLDNEAEPFGGKVVVLSGDFRQILPVVKGGSATETIDACLKSSELWPLF
> -PKIIYCYGSDGERYKQLVKGQDDTRQDLVIEQVFDTMNQFLMEEKATRK**RKLR**LRTYRVIPLSPIAGVLEWVENTIPWGSYLVSRMSKRLSAHERYHPQEWKH
> MGFKAGMTHIVREVDRPGSKVHKKEIVEPVSIVETPPMVVVGVVGYLETP**RGLR**TLTTVFAEHLSEEIKRRFYKNWYKSKRKAFTKYAKKYQTAPADIENELNR
> ------------------GSGKTYTMKSIYRQAALDLFQQLDEMRESASR**RSLR**ITVGVSFYEIYMNSVNDLLNGRSRVQLMEDGDGAVQLPGLKELPASSADE
> QYYRWEPYLRRSVFEFIRLEDVAYTVNEDANKTQREFFVNFYNFQHVSHI**RDLR**TRSIGELLSFSGTVTRTSEVRPELLFGAFTCAECGGDTTGVEQQFRYSEP
> --------------------------------EFKRKNRNLDPSSSARAM**RRLR**TACESAKRMLSTTTSASVEVDSLFEGVDFSSTVTRAKFESLNEELFKRCE
> -NFTSNLDDPALGPKYTRMLQEVANRERTSIPISLDDMLIFERDDPSFVT**RVLR**NARRYISLFSDAVDENLPPPTRDISDSQDVLDVLRMSRAQELAQQRQTTD
> ---------------------------------------------RRAAD**RHLR**LLQQYFGPKPVYDSSAFRKRFRMTQRLFTKVLIGVVETDDYFEQKADATG
> ---NVVPLSMDHWPNDPNERSRIESSGGFVNFSGLWRVVGQLAVSRSLGD**RHLR**KYVTAEPSVFHSVLGERSSGAFLVVASDGLWETMNNEDVVRFIAEKHRRA
> RCSSGATSSPSVVEIGDVKATVGTPQSPELVPRGYLTEVQQGRPIGQELL**RHLR**WMLQKDLLKQDMFLIGPPGSSRRLLSMRFCELLNKEVEYVAISQDTTESD
> --------------------------SVKMVASAGQPTRLYVKGVFLGYK**RGLR**NQYSHTALVKVQGLTDKKDVDFYLGKKIAYIYKAKSLKNGSQFRVVWGKV
> SKRFLKHVRDTVRPEWVCHAAADCGAGIGRVSKLLLLSMFDHVDMVEQSP**RLLR**GVPQYLGVDETLRGRVRDLYCMGLQDFEPKPASYDLIWMQWVLVHLTDVD
> -----------------------TELMRKASLIVWDEAPMAHRHAFEAVD**RTLR**DVLDNEAEPFGGKVVVLSGDFRQTLPMVKGGSATETIDACLKSSERWPLF
> -------------------------------------APMAHRHAFEAVD**RTLR**DVLDNEAEPFGGKVVVLSGDFRQILPVVKGGSATETIDACLKSSELWPLF
> ------------------------------------------------NQ**RALR**RLRTACERAKRTLSSSAQAYIEIDSLYDGVDFNSTITRARFEDLCGDYFR
> ------------------------------------------------SS**RGLR**VTALKDGLEALARDSQRGVHELLGNLGDAFASSESFWITNQVYVQQATPD
> ---------------------------------PRIGASESSSSKKRMQG**RQLR**AFAKTCRTFFSLSPMYMFSSSGPFTLRKLRLHSVAIALASSVFPVPGGPY
> -----------SASTNSLDVCVRNRQLPPFERLQLAEIEPAVINAAADFA**RELR**ELERKLQDSGKNVLFGDVVDPLEVQGDALGRMWGIVGHLMSVRNSEELRA
> TFDVPKKYQLIKVVGAGTYGEVVAASDVESGTTVAIKKVANAFQDLPDTK**RILR**ELCLLRQLSHPNLIQLYDVPRPERLSYLEDIYLVTDLMETDLHRVIHSTQ
> ----------------SAFCIQAAWKSYTHRRIFRYYRDLLSFRSTGDPA**RMLR**AINPSEASLLDASMGAQVRFRLGGLAFPPTIYYKIFTRKPVCDLGAFSPK
> ---------------------------------SSTDESGASADATSHDA**RVLR**DATDGRLGVAKPVTDCKSLLDVDLSHVTEEGGTTVTSATETTHEGHLYCF
> ECVLKIIAMGLQGKGSYEQDTWNVLDFFVVVSSLVASLPGMPNVSAIRTI**RVLR**PLRSLSVIP-----------------------------------------
> -MVLAELGSKLQGALAKLNRTTVVDDEVFNALLKEICGALLESDVQVKLV**RQLR**DNVKLAVSLEDASSGANRRRLIQKSVVDQIVQMLEPEAQPYKMKKGQSNV
> QVYCRSGAVLIAVNPFKEIEGLYNLQKYHDEMPGWTALPPHVFSIAEGAY**RSLR**RRLHEPGESKKNQTILVSGESGAGKTETTKFIMNYLAESSRDSAATSSNK
> FTLDTCYHLVKPIGNGAYGAVIAVKDVVNGGDNLAVKKITNIFEDLVDAK**RILR**EVRLLGHFSHKNITKLLDLVPPPSRKQFDDMYIITELMETDLHQVIYSMQ
> NSDSKSKFRESVYKLNNEVISSKNLEFWDKLWRIPTVAEEIFTLIQPDDV**RMLR**HQQPENLATMLKQAVSQIVQIVDTPVPKYYQQALNCVRVLTRLVPFILEE
> ------------------------------------------KPSGIRCA**RKLR**VHRRTQRWAQKSYNKSHSVTAMKANPLGGSSMAKGIVLEKIGIEAKQPNS
> EGDVLCVLSQFGEIEDIHLVRDGKTGKSKGFAFLKFQDQRSTVLAVDNLN**GFKL**LDRVLRVDHVLKYKLPKELQEDSD--------------------------
> --------------------------------------TRRGRIVRKVRE**RYLR**DDLSCSLPNCALCSAEDDASAATHVLGTTKTVLADTPRDGVYLLPSMKTF
> ISLIVNEDAAHDCVQKLGDLGVLEFTDLNPELTPFQRRYVNYVKRCDEME**RKLR**YFEVELAKFSITPKPAGSIDQFLAGSADIRYGSQDTAARALDTLERLLED
> ------------IHGDQKRRKGEQKLHEERWQAASNAAHAAAAGSASSSA**RVLR**EVTLETPIRYAPNDPVERWLNALLCLDATNASHRLVAGTPHPRDCELYYV
> ----------ITVELLRKRAEHNEGIISSLEEISLHQEELEKVEVIGTLC**RKLR**ILYLQNNIIEKIEDLTHMKELRYLNLALNNVSKIEGLHSCEFLNKLDLTV
> ------------------------LWEWLVMPQGLKNAPATFNRMVSHVL**RPLR**DFAPSYFDDIFVHSHAEEGLSAIDVHLRHLRQVFQVMRENKLYANLKKCV
> ------------------------------------------------RS**RQLR**TISRRSFLKPPPVPQAQAASSSLHKMLARHALKQLRASSFLANRSFAVLT
> ----------------------LLVTFRSRPNMKHEDDFRLSKAAENQLA**RTLR**NEESGRSRHTGRDDRATTEQVLDPRTRMILYKMLNHGIVTEINGCLSTGK
> --------------------------------------LHEDVAHALTAM**RFLR**PSPIQLHALPVALFGNDVIGQAKSGTGKTAVFGVTAIEHSIRYVEQRGQQ
> IARLELENFKSYGGVHVVGPFHRFTAVVGPNGSGKSNLMDAISFVLGVHS**RQLR**SNQLKDLIHKVPGDAPDSGRSAFVTLVYELKKEVKFTRIISDKGVGSYRI
> -------------------------------EEELQEEEAESLLSWPDMR**RRLR**ELAEPITLFGEDMKARMVRLRRAEIDAATRHEDDLGAGHDIRNRFVGGDG
> ----------------------------------PTVRDLVDELVLSLKR**RQLR**GSYETAKMTTSLLSKVVRIHEQVTWSTAGEILDKIRQLGHMLTKAHAHEL
> -----------------------------------------SRRAIVWFR**RNLR**LHDNLALDAAIHSQQQTLKAGEGEMALLPIYIVHQPVKQRCGPVRFQFLL
> ---------------------------------KKHGSRQKVAVNKEFYR**RFLR**LFKVIIPGPFTAEVGFAALVAVMLVARTSFDIVVLHTFTAIERAIISRSQ
> ---------------------------------------VGFVSPYKEQV**RVLR**QEITRSGIPASVSIEVNTVDGFQGREKDVIVFSCVRSSRRGGIGFLRDIR
> QLAGREMLYFHVEACARVPNLQEILMIGSYDEGLFARFFDSVWRRFNVQI**RYLR**EQKALGTAGGIRFFRDEILDGNPASLFVLHCDVCCSFPLNEMMHFHLKHK
> ------------------------FAGVFGMDDAIQTLRVSLIEPLEDCS**RFLR**FGTTPPKGVLLTGPPGSGKTHLAHAVADEVRRRGLASFVSVQCSDLLTKI
> ----------------------------------------------EAVD**RTLR**DVLDNEAEPFGGKVVVLSGDFRQILPVVKGGSATETIDACLKSSELWPLF
> ------------------------------------------KPSGIRCA**RKLR**VHRRTQRWAQKSYNKSHSVTAMKANPLGGSSMAKGIVLEKIGIEAKQPNS
> NFQIDEKYKFIKVIGRGAYGVVISAENTETNEKVAVKKISRAFEDLVDAK**RILR**EIKLLQHFDHENVITIVDLLPPPSLAQFEDVYIIADLMETDLHRIIYSRQ
> -MTHSFPEPIKLHDDETSSTQSEASDSVPNTFEGPEKNLEIDFKLGVGHP**RGLR**EVPRADLDEMLEAGQCQILSRTSNEHIDAYVLSESSMFIYAHKIVLKTCG
> ------------------------------------------MKVLVWLR**RDLR**LHDNLALSAALHLAKQQGDGGKVELIPLYIVHRPQIMRCGARRFQFVLES
> ----------------------------------------------MLAK**RVLR**LVVERPPTPVLRTRPSLAVRRGSRALGGALAQQVLARGITTPSLANFSSS
> NVSDNVETEVLQLSESSRAAQEKHAQLLRRIEAEKRARNIAVPTSVEEVI**RRLR**QLGEPITLFGERPADRRERLREILSRLELEAEETGFVHQRERQEKQMSAE
> FISVAGLRVDGRRSEEVRRIRTRFGLFSRVDGSAYYEQGNTKVVAVVYGP**RELR**VGPAGGAAAVGTGSGNAASNTQPRAVVNCEFTQAAFATSERKPQRSGDRK
> CGRHGRLRADQQVQAPGRHHEPVAAVQGRPDGAVLGPGGRRRRVRQGPVG**RPLR**EGAPGLRHRQAVGELRPGDPQGRARLRVHRGGRPPELRHGGHDRARPPHH
> QVRIIGKGSFGVVTQIVRKSDRKMLVWKEVNYGAMSEKEKQLIVSEVNIL**RELR**HPHIVRYLDRVIDKQATKIYIVMEYCEGGDLGQFIKRKKREGSYIEEGFI
> ELDDITSEKQLKQRETQGQDKDHSAHSDAVLYKIDVPANRYDLLCVEGIA**RALR**IFLEKERPPVYTLTPRAEGPHHITVKPNTKLVRPFVVSAVLRDVQFTQER
> -------MENHVEHLRAVLVCMRQNKLYANIHICIFGAEEIPFLGCFIGK**RGLR**ADPVKVKAIVDWPVPKNQKDLRKWLGLANYLHKYSENYAEMARPLSNLLK
> -----------------------MEDEVYPMTLLTEELKSDEVETRIKAM**RRLR**TVAQALGPERTRSDLLPFLREATEDEDEVLVALAEELGGFVDLVGGTEHA
> REEMQAKPYRALIGCLLYITTCTRPDVAYIVTQLSRFLENPGQQHWKAAI**RVLR**YLKSTQDTGIAYNGNAKKVEVTAYTDADWGSNLDDRRSVSGIMVVISGAP
> --------------------------------------MAKHKPSGIRCA**RKLR**VHRRTQRWAQKSYNKSHSVTAMKANPLGGSSMAKGIVLEKIGIEAKQPNS
> ----------------------------------------MLRSSQRLVT**RPLR**LPARSRAIASYSEQYRRSLDKPEEFWAEAARDIEWFKPWSKVLDTTRGPS
> -----------------------------------------------MFG**RALR**NPKLMSLPVRQSAQVRRLNLHEFQSLEIMKDFGVATPQGIPADTPAEAKA
> ---MATNPAPPPPVALRGLNFAFAKVTIGGHLVNRLQYDPVTGAPIGDDD**RVLR**DVNLTLQPGQRVLVVGGNGAGKSTLLSILAGKHLTADDTALIFGRDSFRD
> ------------------------MASIDEPLDLIKLSVAERIYVKCRGD**RELR**GVLQAYDQHLNMVLSDVEETITVQELDPETYEELIKQSKRTIEMLFVRGD
> -MAILNPEADSAANLATDSEAKQRQLAEAGYTHVEGAPAPLPLELPHFSL**RDLR**AAIPKHCFERSFVTSTYYMIKNVLTCAALFYAATFIDRAGAAAYVLWPVY
> ---------------------------------------MVLSSVARLST**RSLR**CSRALAARSLATAAAPFDPQVPHYDVVVVGGGLVGSALACQLKSNPVFKG
> ---------------------------------------------MAQDM**RALR**DYVTAMDGRQYESVPEGVVCLLITHSNLKLQMVDIRLDLHGTIGELRHKV
> SQTAARFSDEKPAGAEAAAKPEEPAAELTEAEKLQKQVKDLEAKNKDMND**RLLR**ALADAENVRRISRVDVNNAREFAISKFAKALLDVSDNLKRAHESIDIEAL
> ----------------------------------------------MLPT**RALR**LRNSFVDRLRVELAEIEAAGTYKRERVIASPQGAEISVGGDAVLNFCANN
> YERAVKAKAEIEAEIEAVGAELTSGNNPGLHGPLVDAEGFPRADIDVYRV**RQLR**HALAIKRTDHQQVMRKIEELLPQVFAARSGPKAEETPTTPPKAKQVDATL
> --------------------MGTDIQKIHVANPVVELDGDEMTRIIWKDN**RVLR**PGARAPRRHQRRGDARGRARHQAPPRGHQVRHHHARRGARRGVRAQEDVA
> -----------------MAVLSKAHSAFVARKRSAPNTQRNVAVNGDFFR**RFLR**LFRVLVPGPFSAEVGFAALVALMLAAKTSFDIAVLHLFTSIERAIVSRSQ
> EDVFKNQFDNFPKGPKKSEYLCELLREGIFAVDNEKWYRQRKTASNLFTM**RALR**DSMTSTIQRHLVVLDRIFNRAAETDDTLDLFRLLNRFTMEAFTEIGFGVH
> VFEVRSHYVNLRPVGGGSYGIVCSAEDTLRGRKVAIKKITDVFDDLTDAK**RILR**EMKLLRHLGVHENIINILDVILIPPNVMDFHDIYIVTDLMESDLERIISS
> DLPLAPLGTIPRGDVDRIPEDVVRALDKFIVGQQEAKKAVAVALRTRWRR**RQLR**NDALRAEISPMNILMSGPTGSGKTEIARRLAKISGSPFLKVEATKFTEVG
> MGYKAGMTHIMREVDRPGSKVHKKEIVEPVSIVETPPMVVIGVVGYLETP**RGLR**TLTTVFAEHLSEEVKRRFYKNWYKSKRKAFTKYAKKYQTAPADIENELNR
> -----------MDLLGIRTEEELHGWLVKGVFALGVVTYAATTFITAPYG**RHLR**PGWGPTVNTRLGWALMESPSAVWFAVVYLWGEHRSETTPLIAASLWGCHY
> ----------------------------------MDMYTFCTALGKEEAN**RQLR**IHYEAWVTESDLKEMAEAGVNSLRVPVGDWMFNPYEPYIGCTDGAVEALD
> -------------------------------------------HHEQARP**RRLR**PVHDARHRQRHARRVRVDRRLGPGPALQDQDADQGRQVDRRPAGVELRRL
> ---------------------------SSPSSKPPSLHFFLLHHHEQARP**RRLR**PVHDARHRQRHARRVRVDRRLGPGPALQDQDADQGRQVDRRPAGVELRRL
> RQLMRSKPYRAVVDSLMYLMLGTRPDLAYLVRECSQFSENSGLLHWRAAK**RGLR**YLKDTADWGIQLGGLAWSDQKLDDHLRAYADADFANRVDDRKSAAGYVTQ
> REEMQAKPYRTLIGCLLYITTCTRPNVAYIVTQLSRFLESPGQQHWKAAI**RVLR**YLKSTQDIGIAYNGNAKKVEVTAYTDADWGSNLDDRRSVSGIMVMISGAP
> ------------------------------------------MMRARQLT**RALR**AASKQQQQARRVQEEHKRAPPKTTDKFIGKTGAEIFHETLAEMGVDCVFG
> --------------------------------------------MGDKYD**RQLR**LWGAEGQRRLASTHVLLVGSCATGSEALKNLVLPGVQRFTVLDDQTVTLA
> -------------------MIGCAATPAWILESVDAISTTTNKDTQIQPR**RFLR**SAPKAPDNDRSASSPKPLKVNSGSKLKNKLERVSVPAITMENEMWNKILT
> -------MENHVEHLRAVLECMRQNKLYANIDKRLFGAEEIPFLGCYIGK**RGLR**ADPVKVKAIVDWPVPKNQKDLRKWLDFANYLHKYSENYDEMARPLSNLLK
> ------------------------MSALLLGTEPVTEVKLHPVVVLQVLD**RALR**RAEGQKRVIGTLLGRVEGGVAEVSGSFAVPHLENGDEVAVGRDFHTHMYE
> ------------MSWQDRSRQRASGGGKSTIISLLERFYDPTSSSILLDG**RDLR**TLNLKWLRSQIGLVSQEPVLFAATIAENITAGRTDYTREDVITAANLANA
> --------------------------------------------MLPSVA**RALR**RPSSCHRRPLGALSRRLESTLAPLTQLSEEETMFQDTVARFAADVVAPHA
> CCPQRLLLPLLSPYTLAAPRSALCRAHKMLEKPPTALPPHLPRVHHLRLR**RRLR**ALPDAGSAEMKEVATPIRQGAEGFLRIQYSAIARIAVAIAGLIFFSYALR
> ADVAMASADESAGLTSYYEQKIEHLELTVRDETQNLRRLQAQRNELNSKV**RLLR**EELQLLQEPGSYVGEVVKQMGKSKVLVKVNPEGKYVVDVDKTIDIAKCTP
> -------------------------MRLSPREEDHLMLHSAGFLAQKRLA**RGLR**LNYTESVALLATQVLEFIRDGKTVAELMTLGAQMLGRRQVMEGVASILDE

**END OF 2nd SET of 382**

> --------------MSGKGKAATGGRGKKTGKSSTRSAKAGLQFPVGRVA**RYLR**KGRYAQRTGSGAPVYMAAVLEYLCAEILELAGNAARDHKKTRIIPRHIQM
> ------------------------AVTPRSPTLPRLFRHGFCRC-PPRRV**RPLR**GGRVGHRLVAGGAAGRVHGPDPPGRGELRAHEHGQEPPPGVRRLARGRPP
> -----------------MVKHNNVIPNGHFHKYWESRIKTWFDQASKKKT**RRLR**RKAKAAAIAPRPAAGLLRPAVHCPTIKYGSKVRAGKGFTLEELKEAGINR
> ------------------------------------------------MD**RTLR**LTPEHMAARLEFAERMLLAAQKEKVWELITFSDEFFFNLDGPDGYKHYWR
> DKARRLGYKAKQGYVIVRVAVRRGGRKRPNSKGIVYGKPKHHGINELKFE**RNLR**SVAEERAGNKFTNLRVLNSYWVNQDATYKYFEIIMVDHSHKVIRRDPRIN
> RQLMRSTPYRAVVGSLMYLMLGTRPDLAYLVRECSQFLENPGLLHWRAAK**RGLR**YLKDTADWGVQLGGLAWSDQKLDDHLRAYADADFANRVDDRKSVAGYVTQ
> DGTPLNVLEYEEYAKEYLPKNAFDYYASGADDMVTLQENREAFKRLVLHP**RVLR**DVSNMDTSTTLLGHRVSSPVCVAPSAMHRMAHPDGEIASSSATAKADACY

> RFAGYGNYSEKLRSMMHHKSTRLVVDLNDLREYDGSFTDAAVGAQDNIVN**RLLR**NPAEFVPPFEEAVKELVFNMDSMYGSKEAENTKDMQFHVGFEGDFGHHNV
> DGYYQILMRESDIPLTAVSTPSGMLWEWLVMPQGLSNAPATFNRLVTQLF**RPLR**AFTQTYFDDIFVHSRAEAGQTAMEVHLQHLRRVFEVMRANKLYANIDKCV
> ---------------------MCELDEDGSLTFDGFVMFRELIEDERPLA**RDLR**LLGISLEWEELERVERIKELFHEYIDDPSGNVTAKAAQYLLAEIGFALTS
> TSGGGLVSDVHPNGGDIDSLLSSDADKKPRRLHEPYERHRAHDLRAECYR**RGLR**PIKKGPHANDNKGGYIELLRRNDGSTGPALNSTNPVNINNGALPSLLRES
> AARSVLLSDIQSVLSLARRRHGFLLSGTASQRSKQESPLAVFQSFSLLRG**RLLR**CENMAELSPVNVVKPFLDVIRHEHASSTVTGAALQAVMNFLHTWPWADVK
> VWRKQQQALGHLPHVAALISAFLDSSELWNVPQALDYGYLHLLQRLGATS**RRLR**PLSNRSCFLSAAQRGDIAMLQWLAAYDPEFDVYGAVLDHAAEHGQLHVVQ
> VWRKQQQALGHLPHVAALISAFLDSSELWNVPQALDYGYLHLLQRLGATS**RRLR**PLSNRSCFLSAAQRGDIAMLQWLAAYDPEFDVYGAVLDHAAEHGQLHVVQ
> ------------------------------------------MELQRELA**RALR**PLALGGVPESAITALFDTGGTAAVREEWADGASRRQLRAVERVFAKCLRR
> -----------------------------MATTSEPSATQQELEQTLTDP**RYLR**GVALLKDKRLEEAVVAFEDLLRTMCEAEGKADSLAVAPVYYEYGHALLSL
> ----------------------------------------------MMVR**RALR**SQTSKESPGYRDMGRNHSRDEGFVDVQPEAEDSPDESSPRKKNGRKQHGT
> --------------------------------------------MQEFLA**RRLR**RESEGILTEEEESKSGYDEGIVNAREALLEIESGYSCVPGEFGRSLQFVD
> SRGGGRRGGRGGRGGRGGRGGGRHDGPDFRAAGYNDGTPCSVDESVVGIR**RFLR**PEVGGFHGATKQRYSDFVVREVALDGRVVELSDVQRFAPAAKPQKVSEVF
> -------------MFKKLHSKRAHIGSSAADTREVPPFRASLLGRRQQQL**RQLR**VPRPSSHPLGAPRASLQAVTRKLRPPPDMHGASSLDGSEPPASASASDEA
> -------------------------------------------MAMVAFL**RTLR**PDQARLRQLEAIAADADPADIDTARPWVSMPFHMRDPVDRLRFIHGFAFV
> PVNSTDINQLVEDTVLLLKAMGFRCTSRPNSLLLGDWDPSRASREILKWK**RRLR**VSFGLERGTVGTRQTVAKRVVDTPKTVEDPSRIPLPKTSERKRAEVFRST
> LELDECKEEDDASALAVSSPRYEDECSDDDAVAMEREEAEAAIATLQLQV**RALR**KYKTNYELLCGQLGELNAQIGLQLQRHEADVAALQASIADLQTDKIALEA
> PGRGLVLPAGRARGQAAQDQRAQDLQRQRQPRAGRQRGQARGCAAGQDHG**RALR**GRRGEPHGARERARQGRVYRAAHVRAREREPHGAAAHGQHHAPLLGAPHH
> ------------------------MDNTTTSTNSTTAAADNVAPPAASSG**RMLR**PRKTVVAPTARAGQAKSNRGKGKKPARKARVSEPSEPNTDSTPIGLGGGL
> QSEIVLEGSSVVNDAVDYEDDDVEMVESEASSSKRKESIDSDSLDPHAGS**RRLR**EGDDSDASSSKRSRGESDTPLSAAGALSSPREVAPSSSPRAAQSARDPWM
> ----------------------------MLCGSVAPDRDQLTSLISDEVE**RQLR**ERGSVLEAMREQMAKVLAEFEEMAANVSSKRSWSNSVAKTKPFASPKWTT
> DGYYQILMRESDIPLTAVSTPSGMLWEWLVMPQGLSNAPATFNRLVTQLL**RPLR**AFTQTYFDDIFVHSRAEAGQTAMEVHLQHLRRVFEVMRANKLYANIDKYV
> -------------------------------MHLQLKQKTEETEALATEN**RELR**SLVAGYTKALEEAERRQRDIEAKHLAEIENLRAVHSLELSNLQAKHRQKL
> ---------------------------------------MAPAGGAIING**RQLR**NVNEELNYMYAALGIEKDDDEDEDFVLDEDDEEDNGDEEAEDCYQEEEDE
> -----------------------------------------MLRTALLGA**RPLR**SFAGSAPRTRLPGAPPQSHWQEARTQYHYRARSRRQSRAVSGDVVVLGLI
> SSPKSYMVANASTRKKSVGGSDEAAVSGGSPPVVPSQPQRPRSKEGVDSP**RVLR**PQPPTGISSRTDVDIYRYSQGAEQRMAIRPLFEERRGSEDGERPSSTKRK
> ---------------------------------MTIESFAWRVDRDAVRG**RMLR**ATRDLQEGESVLSERAYGNVVLSQHRATLCAVCLCAADPDICCDDCSKVF
> LTSRLGGPYVQINWLSMTTNSASLDFYNYDLNGGLCVQYRPFDEHQLMLV**RPLR**LRLNQRAVRTARFVLDAYISSASSVFYVYELSSRLYMLFRCFSTATNSIS
> PVHASDPPQQHSNNDDTLQFAELYMSKLLAKLRESNKTPQAAAVELQNAL**RRLR**ELQQEFVAKQAKQNLLDRLAQVDTLEALQKAAASRKAQVEAGYEQEKREL
> ----------MHGFDKVEHWLRKKGLRNFDDNSKAKRKVLKACPYYPELV**RLLR**PSRNASTPKPKTHTRDTTSTEDSECSGNDEPEVVSKRVHTRGKSDTMKRS
> GPQLAPTTQGRCPLHQLVHARARTTADARHLGRLRQDERAADLPGREGLV**RHLR**CGLREADPGGAARVPGRRAGFRQAGQLPHVQREPRCQGAEQDRS------
> --------------------------------MAMARAAARSGPRVAQAG**RTLR**FEHAAQMGAVQLIPTHSFGGRSFSSSPATGLLNDEKIVAASPTEAAEQAL
> ----------------------------MAKPWDVDAAAPGSLTSMGVLV**RWLR**TPGNYKRWETQPKKPLVEEIVAALKKERTSPRSPSSVRHKIFKLQHQFET
> PMLAEWIYATFKARVSRATLARLRNLPSFYFSHVDLSATKRRRVKFPQFE**RELR**EFFFQNEAKTAMADDVLMLKAHELAERLGIDATQLRLSNGWLQSFKKRNG
> RDVYFQIYNLMRRFEPVKKWLEAQGQLQLFCRGELIKQVEAQMQRRYTHF**RELR**PVFDHDGYDTASDEEGEDGDEEADQGSSDEDEYHPTKGASPVDRLPWESE
> PARQRRAQEATGPNPKKRRANTPPPASASAPAAKPQPAAADAAAKPLNRK**RFLR**FLKSHELKFGLAPVARDEATGEVTLVVCRFCQHFGREQRPDKQRRSTKNV
> FCQAKELLVARMLCSECDGDMHLRPPVPGATEDRAKRWRWRCSRKACRKE**RSLR**TGSFFAKAKLKLCQCVRLMYFLASSLPAGLAKSWVAVSDKTVTEWYSYCR
> TGSKPRLKHSKRHGALALDASTQSAQLVYPRVGRFFQRKFEQPLKCVMGR**RLLR**VYSSNGKRSFTCRLLSEEDAAKCSETFQSFGVEVICVGEAMLSTIESNRV
> ------------MDDLPVGLCSECGDDSLYLDRTLLEHFSLHVCVTCKQD**RTLR**DGAFELLSKSRARAEYALPDSSFTGLPHLNQPNPRHEAFAPLQLYLRRTL
> ----------------------------MSTAADSKETKTGVEKSESSLR**RSLR**VAGLPAVVPTETGTSRARRSTAGARTPSAEGEAASSGETTPSAEPEVPLT
> TADSTSDGADTDASTGASHATEAGPPAQRRPGRRRGAAARKTAEETRKST**RELR**SADERHSSRSGDEEEDEEEDDNEDNEGRNRSLSNVSNTSSTSASSLASEP
> AWASPSSSSGSVSSSSFGVHVPPGLRHFEGKFQNLRGQGLFYFSLYPPAK**RALR**GVVLYLHGAGDHCRRYIPLYERLCEEGFGVITYDLVNHGASECDGHKTRG
> MPRKASASSTPELGEPYDEHRYAALRHELSVRGIRFRVGTERVTSSAGFI**RLLR**DWDKAHAVEAAASTSPSPSTRTQTQARRTQKPPPQHQKKAKAKATSKKKQ
> ACLQRMFRCRKARLRLRQMLASVFEKFYDADSGLYYYLDKRTGETTWEKP**RVLR**GDDDIELTAVYDGKEEATSEAEPTNSLQVVDLDKEENHETDEEEEEEAEE
> QSAPAPAQQRHSNQQQEQGSTMSTPSKTNNSRLAAMAQLEKAARKLTMYS**RALR**EQLARLREEVAAEKQAVLTSEDDVSESSARLQEIEELMAKLQLDIDALRT
> ARGGFVLDEQGEEDLLREALQTVRDQGFRMQRAADAGDQPAVLKHAAEVL**RELR**TSLLSPKNYYQLYMQVLDELRHFESYVEEQQQAGASMRVLYERVQSSGNV
> ---------------------------------------MAHGRVSRAVA**RLLR**PFHRHQSKDVEAPTKRQRGSMTSTTTSSTCSTTSSRSSAGSGGFRASAGA
> -MDRFGYYLATYEGKKALACLMWHCFGRSSDLGYISKQHITVSADNVFYV**RMLR**VKATEEQGLPLVPDRDDFLTFPLHTLAVALVMQEAPCVSLLSQLPALATT
> --------------------------------------------------**RVLR**ADEEAGVAAAAACKIRQNISFIVVGGKLIQRKVVVCAKCMGEATQWNAEE
> QTKPQQSKKNKRKARAKANKQEQQGPKLKAAWVDDDDDDVEVSLEDHSHL**RKLR**KTENDDVVDGKELHRRLKNFRNGSLGSVSWADPKNFLGDRQADSDDSDNE
> PRAFCTSPSSISDVDFSRSCNTDKQTSSSSDKTRSDAESGDEQEQAQDGM**RALR**AASGSVDINEALAHPVSSSQSLKRKADEEADGEQHHVIKKSKTELTPAEA
> --------------------------------------MLPVAAPAPQRT**RVLR**GGPIWPSVRGARLLRALTSGGLQEEQEGQEYQEQIADNQFSAEFPYELEL
> --------------------------------ETPCSRHVRQLQCAQRRW**RHLR**RRHQRRRHRRRLGPGQPQQLQHLLRCGSECAVADAHVGQHRAHAPHRGRV
> -SVEEDPRSSKHHDRSLGFEALPGRVLGGPQGRPRPRVRAADAHVPRERD**RPLR**EPAQRRRHGQERQGRGHGPRGRPRLRRRDEAADPGGRERHHRGLEVQDVR
> CSSLCALAETVSTNLLDGQELLQQDDILDELATIQQKKASVVQTIEKVQR**VLRD**LDALESLGGMVQNMNIQRVLTGAANTTTTRMESKAQQDKAALKTAEGEM-
> --------------------------------------QPLDHGQGHVPR**RLLR**LLRRRGRRNPINQSRQFDDLFAKEENQSVKR-------------------
> ARVVPLKWQRQVLTHLKPIYQQMTFCRDELVRGWGLLHPPFYEPNIDSFE**RRLR**FFERSVQLLASAVEECKTKLLEAEALAACRYIQLWVRRLLAKRGFYTKVI
> -MRAAMKDSGADDLHTLQEELKRLGISVVSGRQDTVISVKIKGLAGRALL**RGLR**LADQLLQEKIKQALRTCTDPATKLFTDPTFGPGNSDPDGAAAICKAGAVV
> SLSGAENPAFVHDAERCDCVGACSVARVLENCVLQGGVHHTHMHVLTYLG**RPLR**SECCCYPVAVQWFRAVGEQDDFQVIPGACDEWYTPTADDIGARILAKVMI
> LTASDVAANVSHPKSPWYQRSDKFDETVGRDRGVVTCMHDGVLPLGLSLV**RELR**CLGNRELIQVYHCGQEELSNASKTMLLGADDRLELVDVCSDLVQRGVLKA
> -----------------------------------MYVGQLSMEAVDRTV**RFLR**DRGLSQTQALRTISLQVTMCRYSTELMETKIEWLSNLGLSHDKINSIIRR
> -------------------MPPTSSFKARGTSKGRGKGRYKPTTSSTQAT**RKLR**SVNDALTALPTKVKLPTRQQWLPKYEKELYVRPFNYVEGDVAEAICKFCE
> GLRNALERAGLRLRYNAEVHQQTRDRHVQDPPVQVPEPELTEAEIWGRSS**RQLR**RILNWGRNDDFAGFTPSDDEEEEEQEEQDQVALSWFKPTGIAFPRDRSVF
> ------------------------------------------------MD**RELR**QARQDREGVSTSARLRYKVAAARRGIQTERTERSGDELRPAEKTRTIRKI
> RGEGVGALRKLLAPRLDSKQLNRADVKQLLQGEAKNALTMAEFRLIADLL**RELR**ASDPSTPSLASLLRQCSGVQARAVSTEQQDDEQDEESKEKRAKYLARRRE
> SSSSSSSSSSQWIRSIQNQSFEPKPTAYSPHTRQDEDPLMLQLCQCRDKL**RALR**QVRERMKFFPLRPLDKRRDGYSMKIATSLILSGSDESLDWQEEGDQGVGG
> VGPARPTPAKKQNTATKQKKPGKTRALSTERSRQCRERQKLYSENLEAVV**RALR**TEVCDLEILRNLRREQSLQVRGSVSGSLAKIVREYMTVFRHGVPAPTVEA
> ------------MVNGQWSMVCVCLQLASRSQELRAQQEQECTFSPKTNS**RSLR**AADFAAAAEDEDDGGEHSTVATSVETEAALLHPTESQETSIQMHLARQGR
> --------------MRVRIFDPSAMSSSAWGGWGSKLNVTSIVSQGLEQV**RNLR**EDVEKSFDQVVAGAPGGARLPLARPTPLQEEADKEKAAASEASEPLFGDA
> ----------------------------------------------MLDD**RYLR**GLDESSVRSCNGVRVTERILQLVPNQERFRTTLIAVKHWARVRGIYSNVL
> EADNVSMNVIQACVRGDSSGKSLAAIMDRFGYYLATYEGKKVSADNVFYV**RMLR**VKTAEEQGLTLVPDRDDFLTCPLHMLAVALVMQEAPCVSLLSQLPALATT
> ----------------------------------MVSSAEPGAPAPRDRM**RWLR**ALEMDLSSGADSSDATPEPTKQIPGRERKKEKPLERKAAPPVKGNASGGG
> KALCVVSDVRYVSETLQDDFEVLPLVAVGGWDNETNHVTLHLPVRSTHDE**RELR**REFGREEGDARPCELSTLAEAEHEGDVNALQFVAARGENLLVSASSAGGV
> TRRATKNEWRAYLDSENQALKSKCCEWIEGTIYIVEPLTQEHERFTEEVN**RALR**AQRGFDAYMVSWGSAVTPDMPDLHDYEPDKGYGPAKFLGAPFPPGVQDYL
> ---------------------------MEVTVRVEVHYCTPPDTVLGDVL**RLLR**SCTWAAFMARHIGPRLEKVSPVDASALEALDRQWRATGRYDACSSSSKSV
> TQDSAATAASVAPKPQPPQDSSTTQDAAAQQSLGEEYCAQGMTTLWPDSR**RYLR**NLVLGRECRNVFSLEPNTIFLNHNAYGVAPKPVMQAQAHFVNKMEMNPDR
> -----------------MRLGALALDEQPAAASYYDVQDVEKPRADAAGR**RRLR**APSADSSTTSSDDEDDEDDDDVSEGSDSRLAASDSSVHAATAALGTLQLT
> ----------HVGRPAAELLRLDQRHRGQAARLGGPRRRLEGPPHGRLPA**RQLR**QRLRHLRRLPALRHLRHGPDEDGHPRHQDEPHPVHLQPHPGHRLLLHVRG
> --------------------------------MKDEDDFRLSKAAENQLA**RTLR**NEESGRTRHTGRDDRATTEQVLDPRTRMILYKMLNHGIVTEINGCLSTGK
> -------MATMALAAQQQQERAEMASYLFEKHLTHQDGVAGTLSIRLVAA**RNLR**AAASMFWVRTCNPYVIFRVGRQSVRSSTIPSNDNPTWRREFLEVRLPKLD
> -----------------------------------------------MLA**RALR**SSALSRAASRRFIGGFAAEASAPTVAEKVVNVVLVDYEGNRHVVKGRAGQ
> -------------------------------------------MALSSAP**RKLR**LADPHAELPIKNHKYVKNCTELYMANKRIDKLANFDAFVNLEVLWINGNQ
> IEDENQLFETQPTTDEDDEDELAVRAKRLSSGTAASSDGEALTGDDKERI**RELR**ERVLQDANRASSPSPAAAAAANKNHFRRATTVAVPTTGLVPKRDAPEKDT
> RVEGLEGELRGAINDTHRKFESQDQHAQFLASVSSKHPQAASPGRAVEFQ**RQLR**DMQDAMVQLRSELEADRSARWKSDAAVDAKVDAQLERLNSKMTADKRDLV
> KDAPAGPRPVAPPPVSGPWGNPKPAASSAPPPGFAPSPPKPTSGFSDAHQ**RQLR**HRALFAFRFLVGKAVELRLAASEERYAGVLDCVDPDDFSVVLKNAKRLSS
> SQFIGPSPELSLSAACSFRSTTLLDWIWGCSCPTQHDSAASASTDGWTLT**RFLR**SDEFYSRWQFAEVAHVAVERSDTELLQWLFDHFQDPVVPSDVVSQAVHKG
> ---------MSRLVVAIGHVEFTGGSQVLYVVKVQQDADEWEVRRTYSDF**RKLR**DDVQRVMKDARAHILDDACSRDFARELQELPFPAKRLFGSRKDHVVKERA
> RKIVLSDDCREKRPRCSSAWTELEYEMVLEEATLLREELELLLFVAADLC**RDLR**CDDTPVRSFAAALEQIGELIASSQALTVPQIREMCQQMLAMMQYLCRVHY
> ETRAAEALAMKDEQMKILGSQNTQLLTSLNAMDDEIGTLKMHMLRLEEEN**RSLR**DQNFELQSKARAAETTLIKAQTGIEERETQVRVLTDHNTELLRLLEHEEA
> VVEKELVFGKRSTDAAAARSTAGAAWGKSARKWVAASSSSSAREFGDAMP**RELR**TLVAASFDVFARVQTQKLGAFGVARASREYREALKTCIFALEDRLETHKE
> --------------------------MRAAALQLRRQTPQTAAAMRAVAQ**RQLR**SMSVFSNIKKTVSGKLEERNQLKQGEAYKQQMIELSQKEKFDLNGFYDHL
> --------------------------MDNGTTSTDATPAAAATQQAASTG**RTLR**PRKTAAASTKQAGQARPTRGKGKKPARTAGASRARQQETESVPVRFAPKV
> --------------------LLPPEN-QHVCI-HSCSYQQQG-YS-EGDI**RVLR**-EPRAVRRLRQEELQLVCVDVLEVVRLLRLDCLLQVAHQLVLVNVHRDCA
> --------------------LLPPEN-QHVCI-HSCSYQQQG-YS-EGDI**RVLR**-EPRAVRRLRQEELQLVCVDVLEVVRLLRLDCLLQVAHQLVLVNVHRDCA
> -------------MSGESSSPSVPPPPSASASAPAHPPSSACKPKRQTFS**RFLR**RSPYGERRSRAVAPADSVPHPSEERARKRRGAGANDLTTRRQSGVAHLRT
> ---------------------------MRRHFAQFDDELQDSDQTIATPA**RHLR**PHARLPTLADASDSSSNGRARPTRRRFPASAFFSDTTDDDMSRPMARTSG
> FNAASSDWWAPKSTTGVGPLHQINPVRVKYIRSHVIKHFGHTTDDDPMPL**RGLR**VADVGCGGGILSEALCRIGGTMVSADPGEDNIAVAKKHAAMSRHTSSIDY
> -----------------------------------MAALLRRGGRNALSA**RHLR**ASWQAAGLYRSLHSSSDPPPVSSVQSSPYAQTHTTSSVINFGLGQPSASL
> ----------------------------------------------MALD**RELR**RLLEYARLPETDNASSKDGNPPRPTARGILDRIIGIHRQTCVPSMGVSAD
> --MAVKSSMPQFVARCLDEIGLEGRCGMPLRALFDALDPEQDAAYRRYAW**RVLR**GMENQLSFHVMRPLDSRDDKQVDQKPLIGAEETPRRKRKWKTTLLASPPN
> -----------------MASSVRRGSVGLDGAESQYCDLSGRGLTSGQVD**RTLR**VFSTADTIDLSNNDIDKVPSSIPAEVVTLDISFNVIASLHGIKRLKFVQE
> TSSGRMSNWVDLRPLSAGVLSASQSPPRPGLYTARHRVTQELAALKVLDR**RLLR**QRATRRRLRQEVRVLQLARGHQNLLQLHEAKAVGARVELAFELARGGEVM
> -------------------------------------MAKSRDVNREAVL**RRLR**ARLDRRSRGADADADADAQLRAQVDFYVRLRAALFVNSGSTNVDAVDAVA
> -------------------------MGIRDAMRRSAASSASSAAPASAMA**RTLR**LARQSGSLNLSSRDFQSFPEEVFRLYELLGEDERSWECAVLRKLDLSYND
> -------------------------------------------------M**RYLR**AKGIRKALRQFHFLCGIKPPYKVLLDGNFIAMCVQMKVDVQERVPKFLQV
> -------------------------------------MATAWQLQEQEEA**RRLR**ALLQQQQQEEEETRNLRVGLRQHGAPRWEAVRTDACFVERSRRRKPGAMK
> FFVAGSRESLGLRRSRATFTSHLSMTPRASEDLPPRGDTFCCAMTKSASP**RSLR**EVRQSRGSFVSLSSTISTVSSTQIASPLPQGLRVQGWMYWARNEDSKDDT
> ----------MNDLPMDLLHGDWACQREIDLLKRTQAAHQQLTQRLQAMT**RELR**GERFYPTQTETKQLRVRVYHTYVPPVEASEDTQATPGRWTLRIEGVDASA
> -----------------------------------------MERCGSDTE**RRLR**AMEAAFERERQQRDLLEKKYARLKRRYVRLERSHSRLLMESHGKSNSGAH
> ------------------------------MKPYRAGHTGTLSERYFDQV**RTLR**ASRIAMDVQHTPVPPHVLRSPLKIQPEEFVDTPSSSATTSPAESCASDVE
> GSILPQILRDLTLHGECTIPVDTANIINLKLLPTLQDPAPVFEYQAPVAI**RDLR**ALLENSGALFEHVEDLCAAADCAVHRRRPLLYYGCVTLIDIFLHSNIYTN
> QGLGTDEKTLISVLGSKSPETRNLIALRYKELYRQPLKSLLKSETSGDFG**RLLR**MISTPLPETEAQILRDATKGMGSTESLIVQILSGRSNEEMNILKRTYFDL
> -------------------------MASAVEQHRDLVVDGDVAQALDMCR**RLLR**TDSSLQRVGTAHLVLERLRSGGADDSSDDVNALLRLLGNYVAPTRELTEE
> PPPQPPKDAPPPLPDRGEPEKDSAAEAAASLTAIQKLLRRGRLSFTLLEA**RELR**RKGGSVDARRFTADPYVKIAVGTNPLSAQEKRSKTLKKSGTPVIFHEEVV
> GTWRVGDLTPSSTIADIKRWIFEQHSIAPAQQQLALDQQGAQTTSDTQTL**RELR**VGHGDMLFLDYDGETLGTGGVVGTKINADGTLTRVAYHDRADKQAFRPGM

> ----------MASTFVPTPSLSAGEPSPSSSNGGGEARAAPLSSASRFAQ**RRLR**VVTEPKHSNKMHRTRTLSELILLSPRSSPRITPRHKRFDMDIEVSSFFAE
> MELSEYEAKRQRRVQENLQKLQALGVPKIPQRPRPVARKKREAQQLQPVR**RSLR**QRRQRENDTTAEEAPEELQALDSLPPRPKRVKPEPPEPLDLPVTHPAEL
> ------MSLFALSVDQPPAHFLQPKPSRAQPFKKKRKMKADAGVSEYEAK**RLLR**VEENRRRLQALGLPTLGSATSPQKTALKEEEEQKKKEKKEKKRKREETPL
> -----------------------------------MTVLKTKIVQVTHLR**RHLR**PRQRRGATGRARARARLARTALSPTRARPTPTSSDEGKGSKRKRSSKKKR
> ELTAHVVGQALPVRERVNVHGNLVRLCQVGLCKKKGRTTVDEKFVCKDHA**RLLR**EKDQFVDQSQAAFLVRRDYIRGKCASHLAMPVCNYVGCDRDKVTAKYRGF
> WHEALGSDDEQAAEDELCARVLYAQEEDGHHGEERLLQRLHLAQGLLTFV**RMLR**RRSQDDEAETSAAQWTPEWASVTLSRRRFFVLEVEPQIFMALGVHPTVEM
> ------------------------------------------MSLDDVTR**RWLR**RLVSRALDVEPAVVDQLLTGTKTADNQAEPFAADLVQLFFSAKAEAGSVL
> MAATAPPPLLHVRRQISVALYGEVLECELPGDDEPVAVKCISLTRAAEAR**RQLR**TTREIDNPMQEQRVAELIMANGGHRNVVQPLFHFTHDRRLYLVNELCRGG
> -MAAAAPPLLHVRRQISVALYGEVLECELPGDDESVAVKCISLTRAAEAR**RQLR**TTREIYNPMQEQRVAELIMANGGHRNVVQPLFHFTHDRRLYLVNELCRGG
> -MAAAAPPLLHVRRQISVALYGEVLECELPGDDESVAVKCISLTRAAEAR**RQLR**TTREIDNPMQEQRVAELIMANGGHRNVVQPLFHFTHDRRLYLVNELCRGG
> WTVDASGFGIPGGTLQLVEVPRRRWRRPMELPLPPSPAEAPILQEFSRPW**RALR**LPVPTSSSEEEEEVEESLLKCYYPQYSPPEPTFASEVRMPPFLEPRVAVP
> DHDMMEEVLELLATDSPNDDNSVALSSGNNSNGSVGIRLRLDQRHESRQR**RKLR**DLARGGVSDRESIVLKRPLRKEDEGGILLGAWPRAAGQAEVQEQTQIAEA
> AAVLRPSHETFIQSYQHEVIEKSKRGELPLKRLASQTLAEASRSQYRSSE**RHLR**ALLAEACAELPAFPETFARVLSVRATGFVASFASARVVALRLSCVVLGAA
> --------------MLLGRSRSPGGPRSSVIGTEEFAKPQRSSFFGRQLA**RFLR**RASTVAVEQPSHVPVAPSTRGAVSATSLRRTSAQADVAAIGAVDPQIAFN
> -------------MSLAEDAVLLDKSPFPPLQLSYEEQQHCHDVSLQLLE**RVLR**SYDERLAGITPRHHANLDSARWKLEKTQENASMYTERIRHVRSDLHLPDD
> ---------------MLDRSAPTRLALHPRRKRKFFADLAQLLGEEPPEV**RPLR**GAFFGPELPENRPPASDAAVQTLFVPSHSPLTGDSFESVEFGAPRTRSSG
> --------------------------------------------MWHHSA**RVLR**ATSAQYALQRASLAQLPRRRLNTQRTASPDTISALRDLLGDRLSTSRSVL
> ---------MARTTGTPGSSKRPPGPEDAHVTRAQRDSAESERSRSRQRQ**RELR**HETDRTLRDLPPLPVYNRGVLEAQRAANVARARRDEQEIRLAMDGRHKRT
> RGGRVRGAEGYHKEDVLALLKCVRLVVPTSAEEWDQVLEEYRKTHAEPKG**RSLR**DAHSLRVKFKQLARGFAPGKDVHPDVQEAGTIMELMTTKTADGKLLQRRG
> ------------------------------------------------MK**RSLR**LVTWNVNGLRAVLQRLGQNLQQFLEGLEADIICLQETKMTRSELDQELVR
> SPLRLDSSSGDDTGSADERDGQKMVPSASIWRGDEVPDAENADVKLNNIR**RYLR**AARALYEMQFQMKRPSAILKMKPNAYGLKSRSTFDALAALVSPLRAHQVL
> -------------------------MSRIRDEDDEELDESFQHADIPNTF**RELR**ACMTCSLIKTFTQFYDTGCENCAFLQMADNRQRVAECTSAYFEGMIAMMQ
> ------MAPGRLEPPAWRKENYAGNRRFSVTSSEDAPVSGAKVPFEELLA**RELR**KSEGNNAQRTAASGKAGKKPFLKRGARGWWMRQPGAQQKVVKHTLMSKG
> ----------------------MPHPFEEALELTDAEEQDCYDRAFQLLN**RTLR**SYDDRDRQGQEGQVSSTPLHHSKLDSSRWKLLRTHANASLYLQLQRDHRD
> --------------------MSSRHVPFQALRLSDDERQHCQDRSFQLLD**RTLR**SYDERDGQGDEGRRAAPLHHSNLENTRWKLLKTQADASIYTARHKCALDD
> -----------------MIVDSRREAPFAPLRLTRTQRQHCQDITFQLLD**RTLR**NYDERSDSVDGHVGTPRHHANLDRGRWKQLKTQTNASLYAERASRSWRDL
> -------------------MVAMPVQPPRQAVALSEDQLKFQEMAMQLLD**RTLR**DLDEYDADHRDHDKQDPRLWKRIHHKEHFTTYTRCDDAPEAAYVAADGGR
> -----------------------------------------MAAYRRELY**RTLR**DSRHLVERQLLKAQDRHEQRLGDRVVVQYEIGDPVWVYQLFRNKRGEART
> -------------------------------------MTKSRDVNREAVL**RRLR**ARLDRRSRGADAEADAQLRAQVGFYVRLRAALFANSGSTNMDAVDAVAVA
> ---------------------------------MAEEPLSEERKQILQKR**RELR**KAKKAKAKQFKQEKQQARREKKAEIVAKIRAKREEAQQATAQDGCWLCGE
> --------MSGSLSLASGASSSSSSGSVRLSTGAALAAQVTCLTAGAKVA**RRLR**CRKPVSSVFELLLPPVLTPSLVFIANLDSIFGTAGNYSSSSEAATGTIMY
> --------------------------MDVFANAVNVRPPAAIVLLDEEQQ**RVLR**EGRDEQQQQQQQQQRQRRTPPPLIDDEEDHEEDAPRPDSDGNALFVPTTP
> ----------------------------MGLDIKSVIQQKTRWVASKLSL**RKLR**SKRRSRQAMETTALRPVALAGASRGFVGSSTQCNHFARTPSGAQHEQWTD
> -------------------------MSPLDDEATGARFERLAPEYQAAFA**RFLR**RHYRAGLQQLHRRLRHASTPLAALAEAPEQSSQAFSQRVCAQRLLDFNAQ
> KCAMSWESSTSSNTSSKCAMSWEGSTSSECDMSYTSTTEATSTTSSKNIA**RPLR**VYLDLRESESTDEFASTTESTSTNESTSTTEITPTTIHMSPSSSVYTTSE
> RRRVTHSSSPASTPASTRNSSPVRGTLSSPAPSKELVSVAVDAVGPNGQR**RRLR**AAAHTGTTSSVPHGVQSGTGVLDDGKVLGRYSSHDCALLCEVILGQLKRQ
> ----------------MREQWEAVQAGLRVFLEEESAYVQFLANIEQELA**RKLR**FEARSRDGSLVDSDFDRFVETAAQLQYLHVLFHSKLKPFAEPRARGDVSP
> ------------------CRSCVSSPHSVPSHHAVRRAQDLPPRRRRLGP**RSLR**ARADHVLGRPALGGPGRLLRLGQDALRGHLQVQPGRARRAPGPDQAAAAH
> ------------------MSYLAASTHFNNWVFTTEELARVRRLQHVKTK**RALR**LEREEAQKPEASAASSRKARSFAALVPRSSTAVRDAFDWNDEVDVDLATQ
> RTGLRQLLAAARSSTDNNLQHHPALQALEPTVEEHAVVTQLQALAACLVH**RELR**VLVVKHLRPFLLDLMVRLTDPALALYEQEQEQYRECADSMKRSELLAATL
> -----------------------MTTSSTVSAASSRQAPSEPLLVRTNTE**RDLR**LDAQRRRSVPKITGRKLRTPRKFVVTDVPDEELDVLTPSGAVSDASEPQH
> -------MAASGRWQREYERDWRDYTQRVLVDCFGKFAVQCAPSVTQEDE**RQLR**AVLAAAGIKHVPPNHSRWQAPIPASQPLSEPQDSLISNGSYVYSTPPPPE
> TAPPPAQEGAEKLSNGAPSSNGTLPATVEDADKAPAPLKPKPKPKIRAPP**RRLR**SGSGSGSNTSSSGVRRESGAPRPASANMGIVVGTLRRRNRVTLRTSSLSD
> -MPSPAPRRLTDNTAAARKASSARVFPPPSRLLPLPRLGHPVGATSRTFI**RPLR**VPPAAGTSCTRASEPPAVRRRPSDLAEAQSDTVASFQRLHGMPGPDDDAV
> LTVEACALALRHEELEKDEAALVESEAIAVQNKVLAEKKLMEVGGYECVM**RILR**KALQKAKLEIMDTEDETRELLHEELLLRAQITNMTESLVTTQQSVARLQQ
> AMKTAIHDNFVKLAHSTRRTQLATASKWKRLVAQFRSNVKLDREVSAALG**RVLR**GCEPRWTRRELDLLTRAIEDLGFPALQSPKCASKLHPSFVRLGGSARSCD
> -----------------------------------------MVLAGRWCG**RWLR**RAAGATRGDQRTPSLLDLPSVAQVPPFPTSPHPSIIAMDACLQKFETSMG
> SAAPLPPASADHEVIVISDDEGESSSEDAPWIPLLPWPDVAEKLLLLAKH**RELR**VDQQAVAALLQDAGLPASADVNRALRLACEKLVDLLAFSRGHDRRYQANK
> VQNSEPPAWKRRTAAPPPIKLPGADKPADKPSYAAAPKSSESLVDAAVQE**RKLR**EEQERRAAQESRDAMAKQSSYDLEHDRAYNHAVKQMEYVRTAEQKAKESL
> ---------------------------MAAAALSGSSFVPLCTSSEGSDG**RGLR**LHNAGVDVLNGAKLAARKIALVGVLNVAEDEKTEPERGLFLSSSAASVAE
> ------------------MSSSEDLAPAAAYAPGARVEVDGPPKRPQQTE**RVLR**SLRAWGELSPLLFTGASADGTLRSYVADERAGLLRWQADYKRLFHANATF
> VISLVTLWTNRYVVAKLRVDSNLLSLAQLGMSVVGGLGSELYLVGWTVCK**RGLR**KVLDDGLKDMVLLAGVRILTVLLGLTALKYIAVSFTQTIKSSAPFFTVVL
> FDGTASGVSTCVTGSSRSAEAIADADPVLEEAPSGSHAPSLSSSCLSFAS**RKLR**RRRSHSRVPLISVTSLYNRLQTSGVVLIDCRKGAEFMAGHLPDALHCPHI
> SCCTMNSVLSTLTWGTWDDAEWGVTRSVDTSADIPTTTATAESAESSQKQ**RSLR**KLRKKGLQAPKNSQLWFEELLERVEGTSFGRTKSTYRCNPLKCLHESEEG
> ------MSHEVDNGVTEPLLKGRSNSTTNVVYDRNAAEHVERGDAHVKRG**RPLR**DSYLKQLGYTPLIFKKMKKIGSFIASMSPMRTRKPNDDDLLSDAFPYHQS
> --------------------MRLRGSEHDKLWVTRGERWVPTVIKGPGKT**RYLR**ITNVGEKELVLRQNHQVGMWLAGDRVPRIQGYVSVGSRRYQEWQNLALQA
> RRNSQQPVEPLAPSVAYLANVTVEVTTKTLGRDATTVALTVHDHKSSSTW**RHLR**SYAECRAFQARVLQVLSRGHLCFAECPWLFAYVQRSLPAQRRGHHLFRVA
> GGSTEIDRVGRQDGAEQVEVPCPRVVKDYHAFMGGVDVHDQLRSQRYSLQ**RALR**FKKYYKSLVLGLIDLAIVNAYIVHKAYHKNKESRPLTHVKFMIKLHLQLT
> -----------------------------------------MIYDDDTIL**RVLR**AARAGQDRQDVAAKNDVSMRTAYKWVSAARDNDTWNVSPRKPRGGRRNAK
> LIDFSSTHAFRRWRFRNYVYKFTALGYVAARVVPVPDDSTLVGVGEFSNP**RGLR**SNAVPPLKGIAKELSRRATVRLIDEYFTSKKCIACHSDLAETEFGRRRRA
> -------MPRITVELLRKRAEHNEGIVSTLEEISLHQEELEKIEVIGTLC**RKLR**ILYLQNNIIEKIEDLTHMKELRYLNVALNNISKIEGLQSCEFLNKLDLTV
> ----------------------MVAPVGKTPRHTLTPAEKKKCLEALRRE**RRLR**RAEEKEARARAAAVAPPKTMAAAAANANLASAEAKQAVAEATTAVAEAIP
> ---------------------------------------------MYVTR**RLLR**STLPPEPVEEKEEKQKEVPFLPPPTPKRRAAVRASAKICEVVADASGPAA
> ARLLDAPAFDHKARKHLLLNSIHEAKQSQAPPVLAKRKRRRRPSAQQIRA**RNLR**DADNSLILNLTLDVNDLRQILVAREQFNARSLQTVERFFHLFKDGYPEAF
> TDPSEDPATMALAMASPVLTGKPAPVTSPNPAKNPLPGLQFAISAQRAPA**RELR**LPSFSSTSYELESATPAASSADEEEMSSQQQMQQQQQEKSAAQRRKKGTR
> ------------------------------------------MINWRAKA**RTLR**SVKTLSEKVSGNRTASELRHRLRVWKQFALLSIQVKQFAHRRGLDLMRLV
> EAENLVRETLAANEAFLADGAKFRDPRWRLVRAKDGLNVYHRPRSRQRSS**RSLR**MPRKKAFSPVLLSQSQATDPWGVSQHDHDEASTDDEASASSSSTKDMVRR
> SMVRLRIAKKDESLHMRRERERHEEQVKSHLAVLNRSSLDQDQDIGTSSK**RELR**CAVHRLVLESDTLRTENAELQEKLQQQQHFQFLVHDIAVDVSARSEDDWS
> ---------------------------------------MVSLFGKKKEG**RTLR**PRVRRATPLMDVGLAVILGAVTGVYIFKDTLQRWQISEGEYVAAELRGAQ
> SSSSVYCTPPSCKHHGVHVVVLHLRPAAACGGSAALDDDPVPGRHVEPRH**RQLR**RRGVQHPRRHAQRLLRDHGRVLRRAGGADHRPAEALLAAARPAHRGALLG
> ------------------------MTRAAKGSRAKQLHPHQRRRLSEEGK**RLLR**NALAAGSEGEDGSIDYSIISNCDDSSIESELEGALQWEQLQQQQQSGGG
> ------------------------------MTRQVGKTKTGGEKSESSVL**RSLR**VAGLPAEVVPTESGAPRARGLTKGARTPSAEGEAASSGEAAPSAEPEVPP
> -----------------------------------------MGCASAAGP**RALR**LLERLRPQRSHLRILPLIHYSPPLSFLDVCCRLHGLCVPRRLRGSDHRSA
> INMDPDVDYQCIVGSLQYLVSCSRPDIANAVRTLGKFLACYTKKHFVLAK**RVLR**YLQGTREYGLVWNVTVPWGLQLAAYADADLRNEKDDRRSITGYVLQLNGC
> ------------------------------MARSTLRAKAAPGSTARDDR**RILR**DTETNRFLSAAVPAAAVSKDTGRVVSPQVVRNGLNAAGLNGRSARKKPYL
> -------------------------MSAVDDVAARIRRLEEARAAKISQL**RALR**RELRYNAVTGVDERLDSGLSVAQLDVKVEAGRNMLFKAGFLSGQRAYVRV
> QMAPARADDRCVTAREHPVPVVPRSRVHANVIAAMVTGGAVKEELDGEDV**RILR**VDGVHTEAWNVYEVWASSLAEPVGDRPLFAASGDTRHRQVPTS-------
> APGERLGRGPGHPERGRPRGGDPLRPRPRPDVHADGRGAVRRGGGRQELR**RHLR**GGHHGGARLQHHVRAVRPVHRHVLLPQQAHHDRPGHGQQQQDQLGLQQQG
> --------------------------------------MALRAFLASLEG**RKLR**SASKDPSRFWQDVAEGEKRRYEAAKGENGLLKVQLQTNKKAFKHVKQCLE
> KPKKVPTWLKTKREIQALRQQREELEAFLLLSEMGQTASISSRAFVEASL**RQLR**AKTTALLAKQRRQAAEDENERLKACVNQFEAFRATLAAAESVSGALQAEM
> MTKKSPVTRRKPKSRRGRNRPIHEIARLKQQIPVLESAIHVLWARADMKR**RTLR**ARAENLQQRSKLQRSVDQAMLLESMLQLYQGQKRRVEASLLPTLGAVGRN
> APLQLAAEDEAQLEAVARIFVQNTVVQYEKFIEQDRRKVDGNHWKCIKHE**RDLR**VFLQRRRKDVERRMRRHMRLRSFRYRSSETAEFSDADTYTPSIGSRDSSC
> -----------------MTPYYDNSSLNNLTDAARARIFEVVDEAIQRVS**RALR**VVPVSGNLFAERFCTSRYSTTPPVCHSISDKEYCLEMPIPDDHFAPVRYC
> TRAETKRQRSSSTGDAAHGSDYSEQLYAALTAGALWFLGVKDMVQLLGTC**RMLR**HDRALGVMALSNCSVGVHLLHGCNGDWMDLSLERQQGSSAGNGEESGFWS
> KPRQARAAEKNSGCSPAAQRKASKSNKTQRRVGDSQCYSTQLQQRKKAEL**RALR**DQALELEEQVKLLLQRRQQRQKTRAEKEKSSKTQNEEDDSREWFKTAVGE
> L-EGAPRAGVPGQPRRGHAHRPRGLQAGPDLAAGRRPRLPGEHGHRQVRG**RALR**RPGRAGPGRQPARDQTVDQGHGRRVPAAVPPALPRGPLRRVRH-------
> ----------------------------------------MQRLVALAAG**RRLR**PSASRLPRAVAALSACASWASPPSSSARARAFSSAKPLPSPSRMSVADLL
> PAYRFNHPPNCRKRWVFNNTVQQQQPAVRLRKARTTPADLPSEKSPLGTR**RRLR**FKEKENPGTPAVHRRRRRLAAHLVIKSSTRDGAHLEVLMPPEPVGDASAV
> ------------------MDLVDRLLDYVATVPASESVDEVKDQLSAYTH**RDLR**AACMHLDLQVPRRLNKKGGFIATLVNYWKGEALLSSTKLSSPSTPDPSSA
> -----MKSQLLHRERDEFYVAYKRMRTSYENAAEVMNDLHREISAQETEF**RELR**QARSAQDDQDDQDNQDDQDGQEDQDEPRQPQPYGTTIADLFGDTSSESSE
> -----------------------------------------MKKWGEAMD**RLLR**ASTLVELDALRRIEEEQDMEILVAELARVTYQADFAFNSLATIPGAQEPR
> ---------------MHVGEGYRGQIPFRPLQLTFEEQERCQELTLQLLD**RTLR**SYVRRAGHHYQQHAAPPLESRQYALEAPEDPHYVRDMRARAASIASHDVS
> ----------------------------------MSRTAQCKISGHAPAA**RILR**RVREDATDMALLVTSTPADSWRFVEESNNCRLYELTGDALPSCISTVETF
> ------------------------------MGKGAGDRTSAEPKNTAEFL**RHLR**ATLLPDANNSRHVGSSAPPPPTNVSAAAPKAAAPRRSQASAPRDSKHGVK
> -------------------------------------------------M**RPLR**QVGARVRLLRAPRATPRLAAPLFTTTDAPSTHLTPKISTNVRFLHAEQLE
> -----MAARVTKLFLQNCAPKKEDTSKTGKRPATKGKAAVTQASTSKNAK**RKLR**QKNKKVSAKVRVLEEARRELEKADRTAENLRKLKSKAPTKSQRLMTKPCN
> -----------------MATPSLGRRSETFWTRVFLFGASCVAGLIIARR**RKLR**RFHLHILNAKSADCGRLFGLDIGGTLAKMVYFQSADGAAAAQRRQMERQL
> ----------------MATNHPRQAFLVSAESSSDSSDSSRLERRAQSRT**RQLR**YLERKRQYERDLQASVETLQSEVSHLQSELHGLRNAELQRCDCPISASAA
> ------MCRNIFGTGYRELLSKLLRYVRVCDAVYASTVAGFCEEAGVPRE**RVLR**AHPGGVVSPKCVIVADHEHRELVLTVRGTASLLDFCTDLCLQNEPFLAGQ
> TPGEHARFLEAVELYPHGPWKLVAAYIGTRSTRQAMTHAQKYRQKLERRR**RGLR**TPHSRRPKAVSVEFSHEQSPPSFEALMMAEAFVLGDALRGPDAWCTVEPM
> LRGPLLASASHATARSPASSATASQTNINSSVRSPQSAFWGLQHRHYANI**RGLR**GKISRVPRFWLWPRFLNNSFFARRLMLSGQWLVEFKERKPDAYDHCGMVV
> DRFGYYLATYEGKKDAALACLMWHCFGRSSDIGYISKQHITVSADNVFYV**RMLR**VKTAEEQGLTLVPDRDDFLTCPLHTLAVALVMQEAPCVSLLSQLPALATT
> ----------------------------------------MEAKVSFHVR**RYLR**ECEERGARPSEATVTAHLRDTYAEYQRKPQGALRKVVAKVLKKAQPAVEP
> AGGARAGRHQDVRGQGGVQGQEHVRLQEQGGLQERARHVPPRGRHVPRQE**RPLR**GVRRLQRHERRPPGRRGRARRLPRRRAGQAAQGRARAHREVQLAPRRRAD
> -------------------------MGWVDVFRRGRAAVPRHSWSVFGPK**RRLR**SVQPVEMLRVIRFPHLNLVQTHVQPSKFQTQLVRWGTVLAVTSATYQFLT
> ------------------------------------------------ML**RRLR**PDARALACASHLLQSVRVSAAPSARALSLLPSARTQRPLDLKLQRALRVA
> EKLSREVHQMQITGAIHTDWLENPKGFLLKPDFKRKAQHVGAGGKKLNSS**RLLR**LHLPGNTYRVRWFVLDGMILRYFKTSTEEQELGAIHLTSVNAVLPSSVAD
> DDDKEQLQARQREDVDMNSRTPIAFEADLLTERTLFLSVNLFMKRRSYQH**RRLR**EGEEQRRLRRHTPLGAEKYELGPTYTFDNLALGSKTDDYIGVVSTMKGNP
> DLVLEMTATFDADGFQLPSSVTRSEVKHALQACDGDVSRAVDDVFSLAAI**RSLR**QQERQTLSERRQQHATLQLSELCSGLGLDESSSFVAALQQLPAEERDALL
> -----MSSIVLQDGATQVEVPCPRVIKDYHACMGGVDAHDQLRLQRYSLQ**RALR**FKKYYKSLVLGLIDLAIVNGYIVHKAYHKNKSSRPLMHVKYMKKLHLQLC
> -------------MFEKVVESVLEEYVSEWVEGLDSEKMKVALFAGKVEF**RDLR**MRGAALDKFQLPMKMKSGSVGRLSIKVPWKRLTSQAVKIKVEDVFLVVEP
> -------------------------------------MKRGRVATRGSTR**RVLR**RKNVVVDEEDEEEEAEDVSQEGEEEEDDDEEEDDGEQEQEEKEKTPPPKL
> ------------------MQMQQQQPAVRLRKARTTSADLPSEKSPLGTR**RRLR**FKEKEDADTPAVYRRRRRLAAHLVIKSSTRGGAHLEVLMPPEPVGDASAV
> ----------------------------------------------MLRL**RRLR**RLWRPQIDGPSMLPPGAATPAAPADSECAAVLAQIVDVVAAAGEDSDADS

> ---------------------------MPPSSLTILAYSTSERRLVDCHS**RSLR**NRVYVSKPEGSSASLTYSIGNSSPQQCAGTIDRSRTPIEQRLATQDLQKT
> CIVDTSSDDENVPPNLHRGGVQAPAESAKSSSPASTSSTGTSTPPWMSPQ**RGLR**VPKEVSNPPPAKPSTAGAAAFTPSTTDPRPPATPPVTRNPRSPTTSSPVL
> FSAQLQCAICLCAYDNPVSLPCNHCFCEECIHRALELKAVCPICKTPAKK**RRLR**YDTTVQELLRATEMLCAAPDASAAPAAEAPAPIKTEKTAAAPAPAPAPAA
> ---MNVNSNTQYGKVIEIEAVTPVACSIQEAAAILWKDMKTVHEIPDKRY**RYLR**GSKPHSLEKNFVMKLRGQQSDIEVNGSHFSRKFVEDTRVVIAKADTIILQ
> GDKQPADGNLTAATQLEQLFERTVQCGENQSGLLLGSVGSDRRSIVSRAM**RNLR**DRFGGFTVVYLNGVILQNELEAFKELIAQLTRATGVKHPVLSYWNMYEYL
> VRPGRGRVLPEGGQHRGAAARGARRGRAAQDRRGRAQGARVHALARGRAV**RRLR**EHAVGRRAGRGPHDGCVRARQEGRGAGQRGRRGRRLVHGDQHLLLEAPQR
> ---------MRSLDDFHVQVQQQQQAAARRRKSRATSGSSSPAKSPLAAR**RRLR**SKEQEDVGAPAVWIAPDWATHPVIESFYGHARRARASSFYEVMCSPMNAT
> LASYATSVKGFLRANSRAEWEDSLYDKGEKKQVRRAVAQLKNQHESQLHS**RRLR**LAELYNREMEEWEDACLANVETPEDRKQNMIRRALELKERRERERQAIVE
> ----------------------------MRESQPATRMSMSLMNQSDPNG**RELR**LWLLSATTHRGVTYYRIAGRFTSTGQTWEVSHRYSEFLKLRDQLVKFLST
> ----------------------------MRESQPATRMSMSLMNQSDPNG**RELR**LWLLSATTHRGVTYYRIAGRFTSTGQTWEVSHRYSEFLKLRDQLVKFLST
> -------------------------------------MAKSRDENKAVII**RRLR**SCLDRKTRGASAEADARLRAQVDFYARLRSSIFVNGDSLGVTALDDVAVA
> ----------MVSLMALTASASASGSGSVSFAEAFKMLSPGSSRPSFGSA**RTLR**SSRESSVADVSVSVSSSASSNSSATVTVGSGASYELATPLTAKSSVVPVY
> --------------------------MASTSTSELRKALSKYALPSASEL**RHLR**AADPTTSYRASFFRLQIDELLAAAGRKPGAKAAAALQTLLFQVKEVLQGL
> ASIHLDASGKRLAATMDRFGFYLATNEGKNDLGYIQNQHVSLSADGTFYL**RLLR**VNTSEEQGLTLIPDKSDFLTCPLHALALAQATQDAPCAALLSQLPDHVAE
> -------------------------------------------MLAKQVT**RALR**RPSAVRFLATADESAAKAPAAAPKFRKKKTFQFSGKDVEQAAPLEEFAYP
> ---------------MPPLELHDFGRDKSKSPKNPTTIAKSRDDNKAAII**RRLR**SRLDRKTRGVSAETDARLRAQVAFYAQLRSSIFVNGDSLGVTALGNVAVA
> IGSPPAKPDNSALRMKDGKQVEVANGSTGETLGLQVDHVYQQATEADVME**RSLR**PLVAACVEGVNASVLAVGSAKTQKSRVLFSPMPEQSLAAAVFQTLLDTLQ
> -----------------------MTSEISQVIAPYTTRERPAGQSAMTGK**RFLR**TIDATDNAAAEERMFSVSAINGVLGKVGSVVETGGAKVVKEAPKLEKYQQ
> -MSFEGSTSSECAMSFEGSTSSECTTSSECDMSNTRTTETTSTTSSKSIT**RSLR**VYLDLRESESTDEFASTTESTSTTEFIPTIISMSLSSSVYTTSELRDHHE
> ----------------MASDKQKNALAVAACAAALAGLGLWARSKQQQRT**RLLR**KQLTHLRFDLSVAEIEAETQRILAQMKRVDDEIAALAPSAVTFENTAQKL
> ---------------------------------------------MPLLR**RLLR**PEELGDAPAAATATAEQLFASSIYSQELRAQRQKAAALCSAPLVAISSLA
> ----------------MSHRFPIQEKVFPPLQLSDSNREKLVNTALMQLS**RALR**DYDKYQKWQASRPPSLRYQLHPAMWKPVKTCEQLTVYRRVPADDAVVTAP
> LDKSVMAAMGVQHGNVLLLTHGGRKTVVIARLDESDEAQSEAARLSTIMR**RNLR**LDIDNEVAVSTEWRKVASVAEKAALRRLLASTSHVHEAASSFKERRPSSK
> -------------------------------MATPAPGSNQRASADDEAA**RKLR**IRAAFDMFDKEKKGSVIQEEVSTIMRYLGAYPTEKDIIKKILPEMQEDEP
> -------------------------------MATPAAGSNQRTSADDEAA**RKLR**IRAAFDMFDKEKKGSVIQEEVSTIMRYLGAYPTEKDIIKKILPEMQEDEP
> -------------------MFYDLLGNALTNSDGDVWQFQRKIFAKLFSA**RALR**ESMTSTIQKHGRTMHTLFENGAASGASFDLFRLLSRFAMESFAEIGFGIQ
> -------------MTLDVAVKAPIPSASDAPLDAVALVLLHHDAHYRLLE**RSLR**DFQRLERQLQQEPRPKASTALPSIIPAIEAYCDVNLSFYPAEQQAARLAI
> ----------------------------------------MEGLLPAPLL**RSLR**RFAAPEDSEDAAPYSALTGGALLGTQKSSADGVLKQVAVWGLLRLSPDDA
> ---------------MNAGEGYRGGIPFQPLQLTYEEQERCQELTLRLLD**RTLR**SYDERDATPHGTPRHHSNLDKTSTCPEITGRILLLLAVGNIQASLDDVMF
> PRLQQREYADAQVARWRSRGLGVMSPPDPYYPERRHWPMRNWLMEIKETI**RYLR**EINLSGDPERPWVANFRGECLCLPVVSDHQARQVDFNILWLEDGSRDYIS
> PAQSVLRPIHFEHDEDDTAERPFRIVGVRDVEVQDWEYPTRVESDQKNKN**RVLR**NRFGHPNFISLWVDKLGEVEGYLKQEKVPYSSEGVVKGPEGHDVLVIPPS
> RVSSSPSPPRDATLRIVTQTLRDENRQLQQEREALEQDKRALGERLARSQ**RSLR**DLEEDVARVYRQKQQELEAQRRAFEQKLQARPTPAETQASKETSSAALHS
> -----------------------------------------MARLRRQHR**RELR**HGDRTQRKMRARKDAVARLDARLQASLCLQEQQEVSAVLRDLVGRVELSV
> ------------------------------------------MTKEEDKI**RLLR**PEADGGATEEVPGVYRSLNFRSLQGPYHHSDTMTSRYSILRADNLDTMLN
> -----------------------------------------MAPEIREFG**RDLR**RARTILIKIGTEIVHSPEGLLAMGQIGNIVEQIAALHMRGHNIILVSSGS
> -----------------------------------------------MWR**RELR**WMRHVVRAQDQDQRLDRWLRRQFPSLPQSFLQTQLRKRKIRLQPPPGVTD
> -----MSEWRPINSKALKADMFTVAGKLGIVISDEMGILFATEPCTSSGL**RSLR**GGLDVVGGRLQRTLLALSPMDDGSQDADAHIELFRNVLAVYNKTVDMIQF
> -----------------------------------------------MAG**RHLR**LEEYASSGLAVESEDVGDALSRVKVRPMHGGRGGAGRGASETQEASGGAH
> IARLELENFKSYGGVHVVGPFQRFTAVVGPNGSGKSNVMDAIAFVLGVHS**RHLR**STQLKDLVHRAPSDDDTTSARTAAVTLVYELADGEAPPSASRPAQQQQPQ
> WLSEPSRSRAPRAQFSCALPKPFRHFARCATEGGAAAAAADSQSVSPALL**RALR**AARAHGAAVFAARAEDVPLDPVISNPLDEAQSEQVAAQMALMRSQRSELR
> PMLAEWIYAAFKVRVSRATLARLRNLPSSYFSHVDLSATKRRRVKFPQFE**RELR**EFFFQNEAKTAMADDVLLLKAHELAERLGIDATQLRPSNGWLQSFKKRNG
> TVESSELFMNTLAKVINAKAGTLSGKKGFQTLFETLDSKREVILSVDGDE**RRLR**EQKLDAWKFLAVTHAQLFTDETYQFNKAAKVVKARFEAMVSNEEEQDEEE
> ------REHFTPPPPTPCRPSHADFGHARDQGARHRGLVRRRGAGRQEAG**RALR**SGQGRARDQVQEARRPHRRARDAPRHERPRRRAAHHRAAAGEGQEAARDD
> -------------------------------------MLSGRVASRSVAS**RGLR**SSPLAPHWLAASQVGGNAVRSFAAPSGGSNAGGGVPGDFVCHLRDVGVTI
> RGGGGRGPDQGGAHQHPQGRRPPGAQGQVRVPGLAVHAAGPQAHAGRTGA**RGLR**QPGDGGAAPLPRAAQPPDGQGQQRRQGQEVSWTTDRAQ------------
> -----------------------MVAPVGKTPRHTLTPAEKKCLEALRRE**RRLR**RAEEKEARARAAAAVPPQKKAAAKTVAATAANTAPAANLASAEAKQTVAE
> ------------------MSTPLKALIQERLRAKMVCRRPLDDDFEGDSG**RLLR**QPVDVTAYDVSSRGDDEDEALANTGARGSTASRRSSSTQPSYPIRLHTHI
> GFGGGDGGPLHLTPAPSRDNSTLLREQEAERERLRMDNFNQALRINFLEE**RLLR**MKQGTDFCSEDLESELAQLRITLEERDHELRQRNFSMIRATEAIDMLNAQ
> -----------------------------MASAADSAPPSADEQRVRQLL**RDLR**VDTGDVVLFDRKCSSMGLYGGAICVCAKFFGQTQWDHNGIVVRAPSLAPD
> ----------------------------------------MPNISEFRSI**RVLR**PLRSLKMFPVRDRSLWSVNGRKSNWKLRQSAHSAHPSTEQLNAKDPEHQK
> -MGLSRRAKAAESGAAASSSSSSSSSASRLLAFFSHRKQDAQVPEQQDKR**RHLR**IKTKHKRPKQRSRSRSRSFSGAEDNNDDGDDAPGSPHANVRLRQSPGGRA
> -------DSPLLLHCQHAQGAGPRKEPLHRVQGPGPRRARLPQVDVRPAA**RGLR**PRLHHVVPGHGRAARRRAAQGPRHGGRHRRHRLAGALLPGDPRRAERAQD
> ----------GAQLVPGVQQRSPRRARRRDRARDGDQDLDLLGRRPRRRQ**RVLR**QLHLHSHPFSLRGGHHAGPRLQRLPEVGRIRRGLPFERGRHSRCRLACAL
> MDEEDVRALAALQVSGGALSERAQRRRVATCPHCHQGFGSASLPIHVPRC**RALR**QTLEDEDEERDKQRSTPIRRGQVPPLADLCLRLVTKHFESICMEKIVAFP
> SIIELDRKDLLIVKDGTVRLAESLDREQQQLGANGEVVKASGTWTKAEHE**RFLR**AMETFPKGPWKAIAEMVATRTVRQTQTHAQKYREKLARRMRGLRNRNGTL
> DRVYDGCVGGVLAEKSRFHLRIASNGSLHGEVRRKAQRGANSDTVTNRKK**RKLR**VIADHPDGIVIAHVIGNAQVVAVEEAVVSSSASTVNCDGSTAAAEYCCAF
> LYEYPATKRPVGGLPNAFVMCWENVLVPTKWMHQQLGLRSSLQALQAAKQ**RALR**TPNLQAALAAVEQDLIQLLTSVSSYGPLFIVSEESVQFVEAMCKTFFPRL
> ------------------------MDNTTTSTNATTAAADNVAPPAASSG**RMLR**PRKTVVAPTARAGQAKSNRGKGKKPARKARVSEPSEPNTDSTPVGLGGGL
> -----MSWLDVAKDLNTGTIGGVAGIIAGHPLDTIKVQLQTSRETGSGVL**RTLR**RVVRSEGAAGLYRGLLSPILSNAPINAVIFGVQGQMVRALQSRENHSEPL
> TSHAALVQWRKERKVYEDIMRARCQTSGEDYAAVTRSVKDSFDRKLLETW**RRLR**WQVAVTEVDDDRLRSEIDNIINSVKNHTLPDVQALFKKQLHFNLKESDVS
> --------------LLHALYSLPTSRHGLRQHPANLPPRRVCRARGARDG**RRLR**HRPGDRAHAGRVRRQGRGLRPPRDGAAADHGAHAPARRGLGRVHVRAGRR
> -MTRTLTVKMCLVCPPTDIVVSTEMLLTPKQVERYRKLEKKQLEVWQVVV**RQLR**VWSASSAGAEEGQDAVPCRPYCILVNNLYPLGQVVSKKICDPPEVYPSPQ
> AASAEEEETKPETGDVNDDNSVSSQEGEAEPQPEPYRKKLKEGEPGTLTG**RPLR**LYADGIFDLFHFGHAKALQQCKEAYPNTYLIVGCCSDEITHKLKGRTVMT
> ----------------------------------------MADRAESADE**RALR**RELQELSKKREALDNSKRRGSGAAAEGVAGRRSVFARLGGEPARGGGAGR
> --------------------------------------MTAARLLGRSTE**RKLR**LLCLHGMYQDAATFSAKTKPLCGADASVDFVFLDGPFTIVPPILARQSKR
> AQLKEAADESFKADAGTQYAELWMGTHPNGPSRVVREDEEGESPLLSDWI**RALR**ANETGDLPYLFKVLSVRKALSIQAHPDLKVARELHAKFPQIYKDPNHKPE
> ------------------------------MVAFHSDLAVASALPLPDFV**RLLR**GQTETDPRPNKDLYELPLPRDPALHEAARRWNEVVRYRVVPEWLPSRPAR
> LEAAGCVVCASGAHRPHPPARLPVTLPAPGTPVPSVAALVADARALAQEN**RTLR**AQYELVSVNNAGLAAHASVLHDRNLALLRHAREGYRAGMVMLEQARLSCE
> GRLGAVADGGLAVPDGGLRALAGAGGCDAGGARVLVLLPARLPVRVAAPE**RQLR**AAHCLLLASDRRGIRPRGARGGRAAAQQPRHRALLEPRGAQGLAGPGRGA
> ----MAAPLHPETYETLGQAWIWPNKQQQVEASASAAARTASLKRRSGAQ**RALR**KSCSERPLSSPVRAMELLASAGSASALDVEDSTASITSSSSGGNNNNDAD
> ----------MATVSCPRECPSVPTLLADCGGTSSASPSTLFLASSARRR**RRLR**PESDAESCSQVSEVETSTLSGLNIQAVELQNSTLTELNLTDNSVADASAL
> ----------MATVSCPRECPSVPTLLADCGGTSSASPSTLFLASSARRR**RRLR**PESDAESCSQVSEVETSTLSGLNIQAVELQNSTLTELYRRCGSCENQLTT
> QCIGPSQHLSLSAACSFRSTTLLDWIWACSCPTQHDSPASSGRTGGWTLA**RFLR**CDEFYNRWQFVEATHVVVEHGDMELLQWLFDHFQDTVVPCMVVALAFLVA
> -------------------------------------MAKSRDVNREAVL**RRLR**ARLDRRSRGADADADAQLRGQVDFYVRLRAALFANSGSTNVDAVDAVAVA
> EIRCLCVSKWKKGYSYSRIARELEMARSTVRDIIAHFNSAPRPPPAHYYR**RILR**DTETNRFSSAAVLAAAVSKDTGRVVSPQVVRNRLNAAGLNGRSARKKPYL
> ETNNTAEYSALLLGSRAAADHGATSLRVEGDSTLVIQQVRGIFAARNAIL**RRLR**DQVKVELARVGSFSLHHIDRQANAHADRLANRALDLRRTMMECGAHTAGD
> ------MGRRGAGRRLNDQERMEILEIVQREAKVKNVDLAKQYGVSEGAI**RKLR**QMKDTVRNRYYMGNEQNRDKRKRGGFKRNAPFEQELYQWIVRMRESQPYQ
> -------------------------------------------MMQVSQF**RALR**RAQSVAHARPVQSALARAFSAQSDVDVEVCEEEMLRALDKFQKDAASNTV
> --------------------------------------MDASDERENDYL**RFLR**GMRPDDANIQASMPDLSTAQSSDLDFRTSFVGPDGLPEIDHDGAEFARML
> PTASDVTMSDGALFEFEEEATRQPYAVQTWTSYIRALSDAPSATRYAVYE**RGLR**ALPRSYKLWKLYLDNVYESDARGKRVDSPAFTQLVALYERALAQLSTMPR
> VVERRYSDFLWLHHQLSKQCAGYVIPPLPAKVVGLLQGPEFLEHRRAGLE**RFLR**KVVNHDELRNSNFFRSFLECSIVELTALKAEAQRANSVNATSMSSVVQRT
> ---------------------MATPDAERWLYLDAATGQQKGPVAAPILK**RLLR**KGIVQPQQLAWTQSLSEWTALADVEPFAAYCRVWAAAWFYMAENVAEPSQ
> YENTMFENDLVMIFPLREGGEIKKPDAFTAQAFVDLLLGKDTKHSRHPFQ**RVLR**TPRCFLDEQGNDMVVNPRRSGAGDTSLEVERQLLEAEYEKFVGSTEPTTE
> SADLGSSDDEEDEEEVQVLSIPSRGRNQDQHECNNGSDDTKDAEISSSGK**RSLR**LWKILAPNCKGESNRATVMYSISFYPWKMEPHGYYSHPEAAANWVPDTVS
> -------------------------------MRGLVRAIFDEQRPPTPPR**RKLR**GASTRRSWDVNDRAPGYSNNTSNNNSRESEPSYVPQPLEGEYNVCFRRKP
> PMLAEWIYATFKARVSRATLARLRNLPSSYFSHVNLSATKRRRVKFPQFE**RELR**KIFFQNEAKTAMADAVLLLKAHELAERLGIDATQLRLSNGWLQSFKKRNG
> DRRASDGAAPAAAVARDDDAHALVQGLGAAGAPRQDRPRRDQPHHGARRR**RVLR**AGRVPRALHVAHPDAQDDAGLLRQRPARLHAPEALQRLRRPAPPGVELRA
> ILAEIWAMRSPRLDGASGDDLPEVDDELSETAPMEFLFFSGCPDDKNLRV**LDVG**YRRLRSNPRATRYPVTPSYATSVPIKPTLVSNMPPLFAPRFG--------
> ----------------------------------------------MLGR**RMLR**AAARSSAEARRFATSAAQTDTKRSSANESSFSRYAGMAFFGAVAATTAYL
> ----------------------------ARRPHLQSLARSIHPTELRYNG**RFLR**SAYSSLRQGCVPWLQAWPAQPVLAHRAGEDPGPDGQEGRGLLPGQEDRLH
> -----MKSQLLRRERDEFYVAYKRMRTSYENAVEVMNDLHREISAQETEI**RELR**QARSAQDDQDVQDNQDDQDGQEDQDEPRQPQPYGTAIADLFGDTSSESSE
> HLGDEFIRRGGRVYHPARVASAKPRPFGQSESLTDEQDEELDSGALRLLK**RTLR**SARSFLMDDELKLLNTALEAAGGAAQRLLSAATGAEDEELPEPSELVADS
> NTGDAISSLNQLPTQRRQETSVSLSNEVAIGGAVGPLSREEKRRRQAEKK**RLLR**KAGIYSDPNRARNEQTREIAFLREQMDKLQLNLKVLQARKHQNGAKALVP
> GCTTKRRATKPGLKSLAGGIQEEHPSTAGPGRGQEPGGAGLRKTRRRRMR**RSLR**KSRPGAETRQEVSAGQTGAEKPDVETLNVLTRTCAGFRYREMALANAPTQ
> RHRHQPHCRRLHRHAAAPVRLRPGGCRHRVGHRRGRDHRKGGRRRRVPEV**RRLR**RVRGGAGGQPDQDAGAVAGSADHDGGRHLGLHRAGADRRDEVERDGVRVG
> ----------------------------------MPRKAKTAAANSSDSA**RALR**PVSLEAWVTSTRRLLSLEQQEEVQTMRDELATLADSENPNVLTHLTLARY
> -----------------------------------------METRLSLRR**RLLR**IDDSDTAAASAALDPVVASSSSGQVFNRSNQSRGFAGRRQQPQQEFEDEE
> ---------------------------------------------MAFES**RELR**VLSRLRSLCGSDVHIVYRSLAPRLEALHRALYGPNDGDESASDDEMDLLA
> QDVQTLKMKAARKHDNDYERRRSSTLRLEPPQSDSRKATSFVRARTSGVN**RLLR**SNTSSTGVLSNGNSNRSSNRHKSKSKSAPTSKTPHEEYDTALAEPVNESA
> STSSKCAMSWESSTSSKCAMSWEGSTSSECDMSYTSTTEATSTTSSKNIA**RPLR**VYLDLRESESTDEFASTTEITPTTIYMSPSSSVYTASELRDHHGHH----
> KGYSYSRIARELEMARSTVRDIIAHFKLNDHCVVAPLQGRPRLTTARDDR**RILR**DTETNRFSSAAVLAAAVSRDTGCVVSPQVVRNRLNAAGLNGRSARKKPYL
> -------------------------MKKRTESATFGPIGSNERVYCTDCT**RRLR**LFKKFKSNANSNYPAPSYGPPDFGRASNFEFECLNDKCAGKGHCQRLHHD
> ---------------------MATTASSAKPKDVAPFLKSLRQILDTEDP**RILR**WTPDGKAFEIHDMAAMMQLVLPKYFKHSKYTSFQRQLNYFNFRKWTKSKA
> --------MTRTAATKQPDPRAQDPADGTPVKEVAPFLSSLRVMLDTESP**RILR**WTPDGKAFEIHDMAAMTSYVLPKYFKHRKYASFQRQLNYFHFRKWTKSRA
> -------------------------------------------MNSDQAE**RHLR**TRAHRTHITPTTRISIGALAGAVSKKPSNQGEVDKLFLKLKLYVANTEVL
> RPLEVDVDGRRCRERCNQLVDAYEAKQKASEKRSGPCEKYTPKDECLAEL**RELR**EIEALLKNDKKQPKDQQQQEDEQASAMRDAALEGMSRGGGKGGSQQQQLV
> ----------------------------MCGRELERDTALNGQKFLADQN**RELR**ATLSQVQAARATTAPQPTTGINTETSAVPQSTTLNQVSSGAINDGVAPGT
> ---MQLEGEPFEGVQIEAAGGPNREVQLKRAVSSQTMLTSSFSPAPVVPP**RLLR**HQASEQSISVMAASAPPPEPILPTVYSSRHPDLNVITPETVVRVLRGEYE
> PPSQRPKAPPPPSPAGAAAPDPVEEGLRSLERWRKAAEYAEEGVSVSDVM**RELR**EDEDDEDYEEKEAPVTSAGMKTETVAEAKAEPAVKGEPEPPVSSLMGRSS
> QAPPQELGTFQGLVLQAMDSGDRPEQWESGIGSADGGDESRVAREPMTPS**RGLR**RDHDAWSPQRVVPMRQLLERSLEEDKPPATRSRTRNTPQPAALSIELPMD
> HALNLCIGYGIGLKENVRNEYKKDPKSEAYVKTRTVVTKGGAFPEGGKVI**RKLR**ALNNFFASSKSPERVARLKDVQKFHKLPQLAALVDIDVRVASTIKLFQRL
> QPDSLRPKLVAKLLNVKPSPRVGTPTQYVLQCTLDRTDLEWTVERRYSDF**RELR**KVLHAFFLRRGGGARPQCFGCAWFAQTLRGYWFPRRHLLCSRSPDVVNQR
> ---------------------------MYSGNAARIYNPQPDPLERGPPK**RVLR**EEQHIGEMVAASKKRVTWRFTLGESDRTHEVVLIHSVMSYKKWTTADDWA
> NCVTFIDVQRVRRKADQLAASMQPLNDAGEREDTTLLYEQVPKCPGCGST**RYLR**AGVVSFQEQIEEERNAIKEFERRRVPATALLQRIARGFLGRLEFRRRLVE

> QRFDSADYEMARRAAGPCAGCTCGKCAPSSDGTDLEREPEPGSAEHREQL**RCLR**LQRFDSADWAMQVQHAQQSPQTDSPPTEPANVAQMIMDRHALKTCALVGP
> -MVQTRGRQQQQIARKELMLGLAKDSMSLLLELGYVINQCYGNRPPHEMQ**RALR**RKNELVSRLETFVGSQQSSREEDNTAQEREQLQASVERLQKQLLDQSAEF
> -----------------------------------------------MPG**RRLR**HAPPVDEVLLPPLRNPARCGESNQLDADAGAQWDDGRHRRARRRRPQLAY
> -------------------------------------MAKSRDVNKEAVI**RRLR**ARLDRRSRGADAEADAPLRAQVDFYVRLRAALFANSGSTNMDAVDAVAVA
> YRTSYHEALGDLPFFSLYGHDPVLPLDVAFLNLGKKWKSNEVAAYRRELY**RTLR**DSRHLVERQLLKAQDRHEQRLGDRVAVQYEIGDPVWVYQLFRNKRGEART
> -------------------------------------MAKSRDENKAAII**RRLR**SRLDRKTRGANAEADARLRRNSIFVNGDSLGVTALDDVAVATLADDTQLP
> ---------------------------------------------MLGSL**RALR**ATRPSLRQPQSLRRLSLAPLRRRAPLPAVLPPRRSRESMLLTQPPSFRWL
> SRASDFVSWLGLGANADSAAKPSPSPLQQPAQAKRKPSPPPPPSNRQAAK**RVLR**PQPPTDASFPAFQLSVDQSKRKAAAYALGRFSQKVKHAPTSKQLLEAWQV
> --------------------------------MELSSLSMLFGVPPSTLA**RSLR**RAEEALSKTLNNYAPAHIAVPSPSRQNELPKLVEAQEPLLKHTFDFIDGK
> ----------------------------------------MPDKACSRPR**RRLR**VRMELSAHAGSRAGSRSNSSENMFANITPPVYDEEKNGPQSCPSTFNSTK
> SVSDASSADLSSPSRRHQQQRPRFDSKASWADSTRFDMGRADSRASDMSA**RDLR**LTGLGGRVSSSGHNRSRADSRASRLSRTSSRSSMSGVRGRQSSFVSNFGV
> --------------------------------------MDVMMNMTTEDV**RRLR**GQFTGSLELEAFVRLMKKSLRDRIHSELDFVVSVIELFHTIDVNGDAVLD
> YSEDADSIESDNDNTGFNQSRGLPALNRSAFEHSLNRSGFDQSFNRSGFE**RGLR**IDNAQVSAIPILSRVDNPLMSSVVIMNGAESGIDSPGWRKAEAENEGGGG
> TYEMLERFVALHPHVKMLEFKDLEKIRIVDLLFIPHEFAQAEELLAQLAN**LEEA**TRELRKEELTLAAARDLFGLLSSATRA-----------------------
> ---------------------MINRCFELLPFIDAEDDELAELLPPAASK**RRLR**DLLGELKDVESVSKALQGADANLLDVRVWFDGLIAAKPSYARYLAPRADI
> ARVHKLLGSRAELEATKALLRTLVTDKAASGAPLVQLEATTRTPTLATLR**RNLR**SSLEQQQLALAQRALDGLERTLEQVSNLAAQVDALDDKCDQVHKFLETTK
> TNDGKKARVNSDYQDAALTCLMWHCFGRSSDLGCLRKQHVSVSADGVFYL**RLLR**VKTAEEQGLTLIPDKEDFLTCPLHSLEVALVMQAAPCAALLSQLPELVAT
> ASISRTHALIRVGSLTLEQLGDPSTRPALELVDMKSRFGSFVNQEQCFGT**RQLR**HGDQISFGAKKTVLRVRYQVFVLVASRIQRGSRPQVNDACQRVGMHLIPS
> ------------------------------VDCYDGAPPDVHNGRPAGGG**RRLR**LPQDQERPVAGSGRDAGRRLRCGGLPAAEGRDDQRPRRGAAHVLHHHGRY
> ---------------------------------MDSDDEARVELELAAEV**RALR**AKLRARDEELSALQDQVAAVTTQLSETSDELECRMSFVSDAMTQIESLEA
> -------MSPTGAAVAAATAASVASRATRAALGSVAPPGALACQRDSSL**RSLR**TAVHACREHVVPATKKTKAQRFLELELEDTVLFPEGGGQPWDTGNIDGVP
> GAPRRRKQQLEQHAALVAQLAAVKQQQQSLREDMLAFAKAPAAPEKAAAQ**RVLR**SLRQLTPEVTVLCAQTGAVVERVANANDVAEKMTREVRRLDVIQSRLGVA
> --------------------------------------MFGLIHQVVVSR**RVLR**RIVIEAVEDFAAENVKYLELRSTPRDLPQDGATRADYVDEVVSALDECRA
> ------------------MNATAPALIPPFRFSTVQQGLYRGAYPTLKNF**RFLR**RMGLKTVVSVIPEPPTSDLAGFCANEKITLHHFYAEKFTSDNVTVSPATA
> QEASSAELTRSFLSFRMLKEALESTNIKDDEEAVMVTRMCVDPRHVEEFE**RLLR**MILKTTHTVGHVSTCVVRPPPGEFTYTSIVKYSGLQAMRQIYPSTVENGV
> ---------------------------------------------MVDTS**RSLR**HDAETFRVAEELIMLSSRAAAASPLGESGDSPSNISGFCEHTKKRSSRGA
> VDEASVLSFKEELSLQAELTTLDAFQLLFRTLQPLVATTPQLLHHLKKVV**RALR**TELQQAKSGESDPVVLRQRLVPVLKLVTALARELGKEFYPHFASVLPHVV
> --------------------------MDNGTTSTDATPAVAATQQAASTG**RTLR**PRKTAAASTKQAGQARPTRGKGKKSVRTAGASRARQQETESVPVGLGGGS
> -------------------------------------MPKASVKEKGFSW**RSLR**KDVEMFVSKCMHCMATASGKIPRPLDETLRATKPNEQLHFDFLSMAEGAG
> RRGEGVQVRAGAQLRHPGRERDVVVDGQRGSAGRPRHQVADHHPDVERRR**RVLR**RQGHRQQEPERLDPGRHRGRVPRARHGRALRRARDPPLGPLRQEAAAVVR
> GRGEGVQVRAGAQLRHPGRERDVVVDGQRGSAGRPRHQVADHHPDVERRR**RVLR**RQGHRQQEPERLDPGRHRGRVPRARHGRALRRARDPPLGPLRQEAAAVVR
> ---------------------MWHCFGRSSDLGYIQKQHVSVSAVGAFYL**RLLR**VKTAEEQGLTLVPDKADFLTCPLRALAVALATQDAPCGSLLSQLPELSWM
> -------------------------MALRQLLLAAERDRQPLDGAFLAFA**RALR**LAPPAAVEDDEEAPVQPSQEDAAALRAQFLAVHAQGELLLRVWPEQHGLG

**END OF 3rd SET of 382**

> PMLAEWIYATFKARVSRATLTRLRNLPSSYFSHVDLSATKRRRVKFPQFE**RELR**EFFFQNEAKTAMADDVLLLKAHELAERLGINATQLRLSNGWLQSFKKRNE
> LHERLRRFLDMLSTIDPATIEANGLLSSLSKLVNEFSAAVNEYADLDCVS**RWLR**PNTFPDNVKVFNERLDGLCLMGLKEATNLLEWRSKYEQDVANALSSSTHG
> --------------MLVPTFSSLSTASTDAQCALLRRLSTLGLVYAKLPV**RQLR**ELAPPVPAFNTEPRELALKGSLLDARTLLGPFPADMLESLATWLDQGIDK
> ----MSSSLENESEELAPLEPMIARSLDSEPDIGQLINDFPQDGTDEAFD**RFLR**THHSKKLRAACTQLGLHPQADPSTNHKDGYIELLIQYRRARLRGEVFSGN
> FPFNRLRPAHLNWPRAPWSVLPAALPAGSETGSGGMPAPRSGVSSVGIVS**RGLR**RGDDVRLGDLYDSAVAFLGARPSTYTYEPDDVTKIDVLHGEAGGLMSVNA
> -------------------------------------------MSLSLYR**RILR**VARTWEGGAVEQDWIRAEARRRFEENRSLRDPTAIEEAVRQGHNQVDVAL
> ------GENDPSVRGAGAGHGNADGLLCGREEEGGGGRRAGAGAVHAQVL**RGLR**DAPRLRQRRVYQGCGQQGAELRHHHGQPDPQAAADPQDPGRQGRHGAHAE
> GPQLAPTTQGRRPLHQLVHARARTTADARHLGRLRQDERAAGFPGREGLV**RHLR**RGLREADPGGAARVPRRRAGLRQAGQLPHVGREPGRQGAEQDRHLERRLR
> DGYYQILMRESDIPLTAVSTPSGMLWEWLVMPQGLSNAPATFNRLVTQLF**RPLR**AFTQTYFDDIFVHSRAEAGQTAMEVHLQHLRRVFEVMRANKLYANIDKCV
> APHAPLAAPRRAVAAAGARAGHVRGRRQDPEGGHGGARPHGPRHRADRGE**RGLR**RGGAGRQPQGPRERHEAHRGLAGQAAEPRRQEGHAHAAAGGRAGRRHAGA
> -------------------------------------MAKSRAENKAVII**RRLR**SRLDRTTRGASAEADARLRAQVDFYARLRSSIFVNGDSLGITALDDVVVD
> ------------MELRRRHAAGAAGLSAAPEPRVVLSESLLRRRRPLVLA**RPLR**SAFQRLAKPKKVSTTEDQAAISSNSSASAHTLSGKRRRGSEEVAAPASGS
> ------------------------------MEALVLKSLGKLRKACTRSH**RELR**DMIDAAQAKVTSASAEGTLEVPFEPFLLACLTRYAKLVAVALDCMEKFLA
> IQKHVRVLQRVLTDHPFERAFVDAQHIAASRLVVPTWYWKLKRSLNVGSE**RRLR**EALTTVDQFVMDVISKTVDKRNAPISDAEDKVHTRGRDIVSLILANETVD
> VLVAGTTLVKPTAEHPFRSVTVKWVEKGQPLHVRALIKNRDFVYMESTGT**RTLR**NGERVGYQLVHSVQFPETPARDSAIRGNMSMTAVYQQRDSNIVDVFIKGF
> ----------------------------------MLTRENLPPQVAQRVA**RELR**KLVLQPLDGIRYLPQDEEQLSEIHAEIRGPEDTPYEGGYFTVKLTLTESF
> -----MWHTASRINTPNSSYSTPLNLYLYLSLYLSPLFRTARKVHAADFQ**RTLR**ELRSQYLQQMETRLRAGGEENDEANREAPGSDHMHVYVRKRPLLPHELRK
> GFKVTNSSVATDYEDVALACLMWHCFGRSSDLGYINKQHITVSADIVFYV**RMLR**VKTAEEQGLTLVPDRDDFLTCPLHTLAVALVMQEAPCVSLLSQLPALATT
> HRVAKVLRIDRKDDEFPIRVDTQEMLPLTIMLKRKRDRNDVDISSDDAKW**RKLR**TFRLVDGEVEAETRADRLNAGLKKSLEDAMKTTKKMLAKEKADKANAGSK
> ----------------------------------------MQSRDRSTSA**RALR**YIGVAVIAAAAVSGVSGRQMHAGIDYHRYLAERDVTKQELADWKSNFGDM
> ----------------------------MASGVQVEGEGAATALSADAEL**RPLR**IRVSTPVSDSADTPTPDGGGGDGSGSARGTKRRREEAALFLEKTYELLER
> ----------------------------------------------MAAK**RFLR**RNKKEEDSDSDEDRTVHLKLLDDVAAKAAQAEHKKAVDHMLAKAMQAQLS
> LFEHFTEFWVTESIVEAAVSVGDMDILRYFAEHDESGADASDRRVAADQQ**RPLR**LVCWGYQDMAKAAEARHIDVVRWLRDKAVENERNAVEAMEAAVANGDLEL
> ----MSSTSPKCAMSFEGSTSSECTTSSECDMSYTRTTEATSTTSSKSIT**RPLR**VYLDLPESESTDEFASTTESTSTTEFTPTIISMSLSSSVYTTSELRDHHE
> GHLLAGSAAVSSRPQAESRDAAATDSWASFLQCYDSLLARELAQEHPQFV**RALR**QLERDSRQQQERQSAQVLATLRLKDARISELQRLVSEQREQLDVVLDELE
> CHGCGKTALCEEFEAQGYDILDEAFMDMPSYALHPQSLLMETSWVCSWFE**RVLR**LAERVKPGRKQVFIADRSPFSAVLYSAKGHLLEPVIREQMKEVQDFARVQ
> ----------------------------MSTAADSKETKTGGEKSESSLR**RSLR**VAGLPAEVVPTESGTPRARRSTAGARTPSAEGEAASSGEAAPSAEPEVPP
> -----------------------LLDLLHGAHVLARMPHPDAQGRRRGRV**RRLR**GHAHRRRYRRGRRHVRHAWPPHAAVA-YQVLAGRGHVRQPVARVQRRHRD
> ------------------------------MPQVTPRDGRLSQKALSSVG**RTLR**PPEGSPFSRFSRLAHQLAVVAVSILYVEISLNSFSTAMIILHGSVSHNLP
> -----------------MTAGIMAGPAGATYRYGYRCNSVIWSRGGAMAS**RDLR**AFGSPLPLLRPESRVGYASQAVKAEAQQRAPARSSVNHREARLKSSCGGD
> PSTETADVPPNHTLYLNNLNDKIKADRMKATLYASLSQHGKILEIVMGRA**RRLR**GQAWVTFDDIPSASNALRAVNGSVLFDKPVVIHFAKEKADVIARREGSFV
> -----------------------MDLGEAPREAIHEKEEELRRAKKLWRT**RILR**DWEENKDTPLAHSLWRQGIPPSIRARVWPMAIGNKLKVTPEMFKIYRRRA
> ---------------------------------VRVRALVGARTGLPRHA**RRLR**LRLLEREPHADGVRRQEGVQGHRHGQQQLHARHGRGRHPQRHRRLRGLRK
> KKSNKGEHFDDVDKRIQIPMATRMKMQMNLKMNSASASAAPASSSSSEDT**RLLR**SGIDQYRSLQHLNTSAANPRRARGSAERAAERGVALMERKQFGAAVPYFT
> -------------------------------------------MASLALA**RSLR**TCARRSAPLMSARAFASVPTPEAPKFSVVDHPVTADAIKPEDYFAVVKLA
> LVRLAGALHGPAPPHGRAQHDAAGRGRRLQRLPVHQGQDGRAGGLRLQPG**RWLR**YDGARDLPLPAAPALPPPQQVRPALEHGRGGRPQPGDGQALPGPGHHDRP
> ---------------------------------------------MDEVS**RILR**CGSHYEVLKLAEPGAVAVFVAVQQVRRRYKELAILVHPDKNRAPDAEAVF
> -------------------------------------MAKSRDVNKEAVM**RRLR**ARLDRRSRGADAEADAQLRAQVGFYVRLRAALFANSGSTNVDAVDTVALA
> GEHIPHLNKGLSTIGVVVCRSGSAATAGSVSHQEEHLPRVLFSDVNVAGA**RQLR**GNEDVDPEERALIPASIKRFAEKVMLYAKTAVGIIRAGGRDQFFDRVFMN
> VAGPVGGRRVPPLRGALLRREPGLRRLGPPARASGQDHGAVRGHDHRQVP**RALR**RPRARAAGPDALRARPGGHVDRLRARHSALPPGGLARALAGAGGLAAAEN
> FVARQPKQTAASSRVSQDKVQLRSKPARDKVFELDTYLDRRDFVGALTLL**RVLR**REDSEQDRARRKKGAPDDNDLGSWRVNTWWVAYCHFHSGDFGEALSHFEQ
> TVQYKDSAPVIVEPTIPADAKEYCGKVQFCDTVSPPAVSLLHRSLSEWGH**RTLR**WLSSLVSSVMSTVSGDSGSSSSSCSTTPRPCVFVAGLGQKTDYGLSTTDS
> ----------------------------------------------MAAP**RPLR**AVEQVFQDKAVFAGLQEVVASTSEFLDCSVELTLAAACKIGSRKLLDRIW
> LPQLQHYNVPQHLHRQVYDCVHEIEGEWSDFDAVASTEAASEASRGGAIH**RKLR**AVSTMPLPAASQVLHVGHDSTQQDADDGQGVIEEDKHELDSEEESDVEKE
> PNFDPLAVDDASTDLPDASATSNAASAPQQQPNCDASEASTGGAALLTAS**RGLR**RVSSSSGMVASRGVGFGISAAVGRRSRSRSGGDNDDHASSELVLQLQRLC
> RKSKLDSTGRICYELKNDPLLLDSFPMDRLMKAMRRLVCDSDTSTRAGAL**RALR**YAISSSASIKHFVDLNLPVFVVRSLEREQKHLAERVQALKVVRRVMEIDA
> -----------------------------------------------MSA**RRLR**EVTKSTPRSIPGTPRKAAESEHEYLRRCVDPVLMPLIESLLLYQPESVYH
> EVSNPSAKASTAGVTVATAPEADLATPATPPPTRNPRSTTTSSPVLLEDR**RPLR**ERHSSRSERRAAAGPCQRRERRHEATPPTVAPNGVMLAARGSASLVPAHP
> AGSTRPKPLMLSVKPFEDRNGFNRSTSAFPTWDELKRQLSRVFSPPNHAY**RELR**TLIAGMFAEPLPESVTVTVFMDGLRTGVARTEVFRSRPTSFEEAVAVALS
> ---------------------------------MSMGDRGGISLLVRNIS**RRLR**PEDIRKEFERYGEVRDVYIPKDYYTKEPKGFAFVEFRSEREADDARRNLD
> --------------------------------------MATANTNPLAER**RRLR**KLENQRLEEAARQRQEELRRQQEAERKRLEDERIAEEQRRLQEREEIRRR
> PEANPNTIEAVREKNKALEIDMKEKNRRIAYLTKKCEALYRSRGVTGASF**RCLR**RQWLQLQDELLAATKTVDPSAVSDEASTEAWQAALDAVDGFGQVRVRAEE
> ---------------------MAARTQQLRQHIEALIRRDAVKRSLAVDE**RALR**RRVDDYYLPMFRWTTEVVEAAQKKQGDAKRCVCIGLSCPQGGGKTTASMY
> ---------------------------------------------MQRGR**RRLR**QRAERDRESIGSSSDSDVELLAQLKDTQELKKEKKETEKEEKKEKNDAWQ
> KGYSYSRIARELEMARSTVRDIIAHFKLNDHCVVAPRQGRPRLTTARDDR**RILR**DTETNRFSSAAVLAAAVSKDTGRVVSPQVVRNRLNSAGLNGRSARKRPYL
> PPPIKWKDAITAMVAAVGKQGEYEDGVTTPKARLFLLPTETTGIAAELNA**RSLR**FGVRENDRFVVCLQGEDYQQPSERQASTSNPAPEAVATTTAAVNGSSARP
> --------------------------MLQPLGRAEQFPARAKELLSPSME**RILR**RGSSSAAGREPAAGDGVALKLPQNVEDTLAKVLPSEDVLDRPEFDAREFI
> PRKSSGAPSAVVSSRTIPTMQRSRSAGQENAALVANATATAADADSRAAA**RRLR**RQYAQQPIAQLEHHYHRQHQRPQLVHHQQQQVQHNSRGHFDGPPRLVASH
> -----------------------------------MLYRLGRRAAATSVQ**RSLR**RGAMTQPCGQRRSFRFSSAAGKPAASDGNSRVASAASFFLDAKAACKGKR
> --------------MAWVNRTTRDNCPGGLQVKASTSASLGPGCYETSPT**RKLR**PNATGFGSGEKLVRGANAGSTKGLTMITPGPGSYATNNQTSWDSPTNSKA
> STFTQTPAYTARVSSPKEVAAEEALVYEELKRTVALLETAVKTNAKEKLP**RVLR**ATSKLRKTLSVAQLQQAVEQLLPSQNASKPALLALLAKIDAKAQAAAADA
> STFTQTPAYTARVSSPKEVAAEEALVYEELKRTVALLETAVKTNAKEKLP**RVLR**ATSKLRKTLSVAQLQQAVEQLLPSQNASKPALLALLAKIDAKAQAAAADA
> ---------------------------------------------MRRSS**RRLR**GEDAADGPDTPVKRQKSGEKEDSPAVKTTTTTSQQRVAWDAFLQSKCFLK
> MEGEMVLLAMLPRDDRVMLVQTPRLRTIANLEDFIVDEFARVFPHLPALS**RDLR**IQKCVSLELVVDRRAHAKVRQQSPHCFVDLAKNVQAGNVFTNMEQIYVVM
> ------MAGHAEDFNLRPVHTQTFHINLTGSSNSGGVAMDIEHLPITMQY**RELR**RRPFDERMLVRKSSIHGYGLCLKEAVSKGQMIVEYQGQMINQAVADERER
> DRFGYYLATYEGKKDAALACLMWHCFGRSSDLGYISKQHITVSADNVFYV**RMLR**VKTAEDQDLTLVPDRDDFLTCPLHTLAVALVMQEAPCVSLLSQLPALATT
> --------------------MAPSFPLSRLLLPKVKVSAEEHRLAKQMMT**RMLR**HTLLAFENFAYDAQGVVDAARWKHIDSQDDLKLYRERVAGATSAGLAAEL
> --------------------MQAQQVGTDEASSSAHPPMQHAEADVPLGQ**RTLR**AAQLDDDLAPWYLYDLSGALQPDTLDVMKDNFRRFHATRQKGIEGYPYEA
> RKLAAKKKKNPAPKSPLRCGCCGGKEDKEGKCGCTVEPRVNPKHAADIYE**RQLR**WKNANDVRHCQQKQLQETLAMTECTFRNPYYQKAWNTIGDPVARLGKKEG
> ------------MERPGARGLSPAEQQKRRALLAKIAQFQARGAILQADA**RTLR**SQLHDSTSGQWRAVELRLQQLARSWNPYQDGGAADKAELKLKEKEKKKLD
> ---------------------------MINRSNTSKSPSSTSAVSMSAML**RSLR**RVWLGSRGADGLEGGLRRLKPPPPLPPHSSKPDESKASLSNEQRLGDVVW
> AGSLLGSTAVFYPGFDLSNLIPQFSFWSSLIDSTAAAPDDTAGNSATALG**RLLR**ANRGAPDAPPPQTPRRPPTHSPSPAKASAMRKNGWETPFHVLQLATWVVF
> ARASLPLRQHPPQRHQPVAPVRPGLRQGGEQERLQISVVAARTIYSQPVC**R-LR**RVLLFGARFCKH--------------------------------------
> ARASLPLRQHPPQRHQPVAPVRPGLRQGGEQERLQISVVAARTIYSQPVC**R-LR**RVLLFGARFCKH--------------------------------------
> RGRREPGRRHPPQLPGAAAVGQGRLHRGVRAVRRRVRPAQAQAPLPRLRA**RVLR**PLHGALRPRGQVRLRGARAALQQLRGHGADRERLLREVPAAARGRRPLQQ
> VVGEEISRIIQEQRQLEGKYEKLIAQRAVLKGLANKSKFKENQREIQEVS**RLLR**ESTKSLCRNLKENPNFAGNLLKIQQEREGLLELLGHTLTEMKKHGTFETL
> -------MAVAPSFEFDPLPPSPRASMLPTTNESAVGIRNGHFVDAQGRV**RLLR**GVNLGGSSKLPSGYQHTDRHSRSSFFDGAASVSFVNRPFPLSEAPAHFAR
> LGPRRGDQEDGDHDHQQDVRVHVRVGRGDRAGPQGRGHHAPARAQGQRLR**RDLR**VHAELVHLRVRVRIRGLGGAHRDLRAVRPLPGRLPRHSVRRPLRHREARG

> -----------------------------------------MSLDPLKSL**RPLR**SNASANLPPKAHAHSTAVPNAYRLPATTRTMLRSLAENSFSVMSYNVAGL
> NSLVSDHEDMVLLREKLTRIAVGRHRRQFDAAPGAAQAAIDEEEREEEEE**RELR**RRRSSGAAHEHFKVFPLLPPERPSTPPSSPQRDVLRKQLWLKQQVAKTR
> ------------------------MLSPLFEAAPPARLTLWLPHSHSQSS**RLLR**KPPATLRLPVMSTASPYQYHLDDESLFAMDTVLKVTSEQALCVEWCIVVG
> YRRQLNELINRMERLVEEEDNLETDSDAAELEWNGAQLAALPGQTVWHNL**RALR**DCRVLLEDAAAQFTLKIRESAELVAAEPQLLALERTPSSSSMPVSEYSSD
> -------------------------MVKPRGKIVRDLEITARDLSFKSVW**RELR**EQGRTRKPPPRRGLDNRYFSLRRDGDASGADSVDILCGEEAVLVYYANDC
> DLRDVTLAVDFVGFLFHLCDELFRASGASASWLLLGGCPLRLERHVDAWL**RRLR**ARNVRLVFVTDPPQCFGGESHRKGHCLQDRAQQKRAQIEQLGRSLETTIV
> ---------------------------------MARKKARQSVAISDKCF**RLLR**LAHEIQTDVKVLKAVVDEVDVLRLCRSLGMSVSSESNSTHDFEFGMFNAS
> ---------------MEVSRVSSPPPPRARPGRYWKYSHSAVSLPRLLQE**RELR**GNARVIDAACQGHGSLVRRLQCESVLRGHSGCVNTLQWNESGRLLASGSD
> GPVATLVQHLPTALVYTYNLHPRMRALQVKDAFHEAEIQPPITVDDVAAR**RLLR**VRATSAAEEETAMWKRHVQEECERVVDRYEDFASFLEVWVDEDNSLARLF
> SVQGTDADKTKKKKKKNGSVQGTDTAKTNAEAALGQACDATSHVDRDAR**RGLR**GLSDLSPQDRHLLLQSGELPALMLFHSSDSPLERWAKVNPRLTIPGACSH
> QRLQREKGEADGMLQLLRSKLHQVEEDNFDLHSSMAAFEAETRFESDKKE**RELR**AQITALESKLSFMSSKLQNAERVKLRAINEVEELQQKQLVERKRRDAERR
> -------------------------------------MAKSRDENKAVII**RRLR**SRLDRKTRGASAEADARLRTQVDFYPRLRSSIFVNGDLLGVTALDDVAVA
> PGDQAGLEVARPRDGPAGGTEEEHPSTAGPGRGQEPSGAGLPKTRRRRMR**RSLR**KSQPEAETLQEMSAGQTEAEKPDVETLNVLTRTCAGFRYREMTLANPPTQ
> --------------MLRRARVARSLQPRRRLAAASISTSARPGSLLAYGA**RILR**CTDPLEKVSLTFECQNRWNSDHLPVFCEAQWGSHFPPDLPARGPKPELLP
> -------------------------------MAYRDLTSRFQERRANLRK**RNLR**FQMRPRLDNSPLLSALFLSKQRSKSHAMEKALLQDANEFPGGEHGDIHKP
> -----------------------MGETQLVQVVPARDACKCAATGAVCKK**RALR**KRVVDSRWLFNNIHVGEIIVDCRSREQFISNTVVGAINVPPPPEDDDTSD
> -----------MINRPGLPGFQRFPLNNNAVVPPAVSTACDWRLNSDDEL**RRLR**SNYVVVIERMLRNHQPHDVLNHVPENTDWRSKTFKLALKIERLMFKAAKS
> LIPRCPHGRRRRRRPEGEAGLGRLFLRRGAHRPRWPVPGSVRGAGGLRPQ**RILR**EQQLQRQLARERRRAGPAHAQRRADRADAEQPRATRRAHAGARERGLAL
> MTIQQPWVAVKNAVQQGRPVPRSIPALYRGLGVSLSSIAPVSAIRFAING**RLLR**VLNGTDSAPTAQTKVLCSTISGATAAAITSPAELVMTLQQINGRSIGATV
> VMLQQPTVAIKNAVQQGRPIPWSVPALYRGVGVSLASIAPVSAIQFSVNG**RLLR**ALTAPGTAPSDQTKVLCGTLSGVASAVLSAPAELVMTLQQNNGKSFGATV
> -------------------MAPATLHARHRCSAPARSWPAQLRARVRDPL**RPLR**PLRDRGPGPLAAKPRPGPPLQPRVASRRRRADAGPRTVRRAGPGPVHLAP
> ----------------------------------------MGLQFSESLF**RRLR**CFRAFASSADAATDDDENAASTNNATAGGPATPTTASSSSSSSSSSSSTV
> LSSFASAADAEPLAVRARVWSSRSCSAASSSAAGGDDALTLVATSDVALL**RRLR**VVHDSFVTLQFQGRRHVGRLRLLPAGSIVDASEDEDGDVTLSPLLAFNLG
> ----------------------------MSPKKTTTPPPKRKQPSAATAA**RKLR**KSCSLPSCSSAQGTCMHCTCDGRCGRHPAGRCGSRREGSGRGCKREGCTR
> KELPQRLRRMPECEGMSRRLHDRIKAVHDAVAAHDEQWKAEYCNVLKRFI**RVLR**QTPLLERLAGGETIAYTFQEFNSKMDKICVGVGVMDSSATGQWQGAWASD
> CRRVYERLRFVYDELQKCSDASSLRESRVLAMYGSAIAQFLSFLESHSRK**RLLR**RLIESARMADRIQDFHKEIDELFKVLSIVHMTEMSAWRHEWEQGRTMRRN
> --------------------MRVMGSEDAHVTRAQRDSAESERSRSRQRQ**RELR**HETDRTLRDLPLLPVYNRGMLEAQRASNVARARRDEQEIRLAMDGRHKRT
> PGDQAGLEVARPRDEPAGGTKEEHPSTTGPGRGQEPSGAGLRRTRRRRMR**RSLR**KSRPGAETLQGMSAGQTEAEKPDVETLNVLTRTCAGFRYREMTLANPPTQ
> --------------------------------------MSAGRMDLEARL**RALR**EGKRLPAAAPAPPPPVAPAASFSWPEVPSSPVQSAVPVRQRVPVAVGSDY
> IFSEAGSVVNFRLVTDRETGKPKGYGFCEYADGATALSAMRNLNGYEING**RNLR**VDFADGGDKSGGADRKRHDNGSHARHGGTSGSNFRNGGSDGGPPTMVTGE
> TEAEAKLERATLAAQRLRVTSQEDAFDTDRLMRFLNQGGAATRGRWPRLV**RLLR**HFQRGSTPPAHWVTNITVDAANRPFKMIPPYPGPVDPDMEDDYDNYQAGD
> -------------------MVHLSQKKSKRAVRAFIRREFPLAYVTDEAL**RYLR**HVLREQVTEAPSPRGPLFDVLSLLQQQTGRRIEGHHVLAWRSSRRPSLRQ
> TNDGKKARVNSDYQDAALACLMRHCFGRSSDLGYLRKQHVSVSADGVFYL**RLLR**VKTAEEQGLTLTPDKEDFLTCPLHSLAVALVMQTAPCAALLSQLPELVAT
> --------------------------------------MSSSSSARAKQT**RLLR**QASVLHELQETQEKDSLDGRYARLMAELAALCSDRSSVITAESLQFFAAS
> PREFIPRSSSQRSNSTPRDANRTPKLRYKDKYVNTLLSRKTTPDSFVFAC**RALR**CKDEIALLAATDPRAWNEALQRRLEARDERGAAELLGLVASFAPENASEP
> -------------------MLTVKLANKEKEVETLRCKQNAVDTEFAGQD**RQLR**QQQEEHARVVSELTTTNQELQRQVQHLQIQSERLQADSETQRTAALEQRR
> QRELMKLENRLSACRTRESKMVAYNAELRHRVDELRANRVLSQTVFEKNQ**RRLR**EIQKQMQETFRQSTQIVAERDRILSQANSLSGQNMDEQEAFDQVYQSLAS
> --------------------------------------MGEEGPCRGRLF**RLLR**LVRETQCELEVVKPVLDELEVLRLCCSFGMMPTVNPMEFDFDVLHASEFD
> GGEPPPFDYRAAIGALMYLATSTRPDIAFSIGYLSRFVRNPSKKHCGAVK**RILR**YLVQTRNLGIQYSASDEIADKIVVTRYCDADWGNDPETRKSITGYVMTIA
> -----------------------------------MSTALATARAKTKGK**RMLR**NALDSYGDEDTSLSKWNARKKKAVEEGKWVWKESVANANAAIKSRFDKEE
> ------------------------------------MKHISVSADGAFYL**RLLR**VKTAEEQGLTLVPDKADFFTCPLPALTVALTTQDAPCGSLLSQLPELVST
> ---------------------------------------------MATPP**RSLR**FVDVVAWDPSSPEWHRLLRQLPAHEQQQVTRFMFAKDQKLALASRLMQRQ
> -----------------------MEQFAHLPPNQQDVLKTSMSLLGPEGG**RNLR**SKAPMRAPPAEDGDCRGAPSSSDAGTRPKPLMLSVKTFEGKEGENLMLWT
> ----MLWPREEFHAAYGQIKHDALQSGGSCSVLVLVALDVDALCAAEILT**RLLR**ADMLSYSLLAVRGYEDVLQARETRIRGADGTLRGGGELRSVVMLNCGAIV
> ------------------------------------------------MA**RHLR**QPAHRLAECEGTTKDGQEREGRRRHQVRGGQERGGGRPTISLRLAADVAY
> KEQQQPSAKAEQRAKLVRRNSRVTVDQLLREAFARVDVDMDGKVDALDIQ**RGLR**VAGVKLSADDVGELMKRLDPKNQGQLTYEDFHAVHEELILQTFRSFDKEN
> QTNGVIPMELRCTYRSKPCPNERARKRNGTLHKLCQTHRIKANKTQRDMQ**RRLR**EQRRQEQLERQLAAMQFNGEEFQAIELLDEFLKTDIPLELDTDTELDKWD
> VDMETGCVLPMEEQEMMNRQQSVYEYDPKRTAASIAAGPQGRDGAGKKQK**RHLR**LAGGQIWEDPTLEDWPDNDFRLFCGDLGNDSTDASDRDWAVILTQVNNWK
> --------------------------------MAQPQKTQVVIWTTKKKM**RVLR**NSLETKLPKRQCLADYTESMDEALSHFETCSLGDIVNGADDRSQFSVNEL
> GDEAEEDAPKHPEAGSIRKIDNALTNPDGCADMVSLNYLHEPAILFNLKH**RFLR**QIPYTYTGAICIAVNPYSWLDIYTKELQEQYLERDRSELPPHVYATSAGA
> -------------------MGNSPLSREFSGRRLSSEEREAMKRFGDEEM**RLLR**ETFKGLANGKDGVSVDKETFLKCFPMRGLLGERLFEVIDKDGSGTIHYNE
> APGPALAHQVQHRVGRLHLPLLDPGGRVLLDAGGPRLPEAPGLLVPGGGA**RRLR**GGAGARQRQQLARRGHDRRETVRVHQVRQVYPEEAQGVRGPQLVAEHAPP
> -----------------------------------------MQGPPSGSL**RFLR**RQPHAKIPAPKQLSMASASRRRGSLGDRRGFFRLCGSPIRTHKMELDYVH
> ---MQRVAVLSHVKASSVPPSLKSSGVRRIAWQGGKISSNKEEALQKFYQ**RKLR**AGDHLTAQQIEAMQATLGVKLDQDQAQKNPKKQHETHRQHAKVTAGAHRT
> GMWTEEEHARFLEGVKLFASGPWKRVAAYVGTRNVRQTMTHAQKYRLKAA**RRLR**EAQRKQAASRHGLHNAHRGLIGSTDDAALAHRSLQSPSAGGLGLGSYSKM
> SSTSSSAPSAAPARRRPRTASTAGPLKGVVAMVDVRVGADAQIDCSDVVA**RKLR**ELGASTVKRFTPKLTHMVLSHFTPVWKTKIAKWQAGGGSMAAAATRYELK
> ------------------------------------------------MR**RALR**HTRSLLTASHRVARMKFAVNHVKLNGDGQYYFDPMFDVVHIDEKWFYVKK
> MQQKLLRDAQSTDEMARLLSQEISLFQRNMYKNHSQHRRAFFFQNLQQVK**RRLR**DFKVETLTGCFAEAAGVLKQLELDAGAHHISWKLLASDLKVGTDAVLRKL
> ---------------------------SEGQGAAREEQNQGEAGEPRVRP**RHLR**PDVVDPTASGHSDRHH----------------------------------
> GFVPGPGSEKKLVISYSTSKTAKDPRGWLYVEDVSGIYCRREMIEIVSPS**RTLR**LKGETAVEHRLWSDSLYKLCNPPPKSATAVVQTTPAVAEPRQERTPRRSQ
> -----------------------------MLYSYEPKPSLQRRLPQTRRS**RQLR**RLEGSRGRSPRLLGFVTKPEYDGVSEDDSEDTGSDMSDADDPLKTTPAKT
> PGDQAGLEVARPRDEPAGGTEEERLSTAGPGRGQEPSGAGLRKTRRRRMR**RSLR**KSRPGAEKLQEMSAGQTEAEKPDIEALNILTRTCAGFRYREMTLANPPTQ
> LRLEDGDVADLLRVLHFVLLDSSRAVAQFLVDRGYDLYGKTDARFVESAF**RLLR**DEFRCFPSLSVPQFLSKQFVARRLAIVADAAAAVAQKRQELQRLKRREEA
> DGALYDAASSSKPDVVHVITSYWADCTPAATDALLRPVTRFWLQETLRLG**RWLR**TDSHPFFEPPPRPADLWLQYPRDYQDATGQEQVDGDGDVNMETPTATAEH
> ------------------MQMQQQQPAVRLRKARTTSADLPSEKSPLGTR**RRLR**FKEKEDAGTLAVHCRRRRLAAPLVIKSSTRGGAHLEVLMPPDPVGDASAV
> VTDAAGDKKKKVFRCTFPGCGREFQLKGNLKRHVNIHNGDKKFACKFCGK**RFLR**KADMEVHYRVHTGEKPYRCKYQDCGKCFARRSDLLSHERTHTGRKPFSCA
> --------------------------MAEKRRKATVTKRKEEISDLKAEI**RKLR**SELGLQLELKELHVVPARTPPLGQVVSDNEALSSAIHGQQLDIARVHSTL
> TDEATQWLASMDEMRDDDATDRAPSPKAAAASPGFVSPSTDSSPVACSKK**RELR**ENHDSEPEVPKKRPKRKFRKSTINVRKEEKANLLEELAELHTKMEELKAR
> -----------------------------------------------VGG**RQLR**QGQEQHRPPAVHRRADALQLHDAARHLPGHGAVRFLARQQRAGHGGRGAE
> ------------------------------------------------MP**RFLR**ETSRERAPDVAPSAPASASASPSPASPSAGDAEPSMVEELQRRKAAFLAH
> PLSQRPRAPPPPSQAGAGAPGPVEEGLRSLERWRKTAEYAEEGASVSDVM**RELR**EDEEDDDYEEKEAVKTETATGVQPVTSAGMKTETVAEAKSEPAVKGEPEP
> VVQLVIRPKRARYSVHELGPERFQVAELSVDPTQDDVTEARRRDFDLVND**RGLR**VCCSHWQLFARGSSTPAVTPCLIYLHSNVGSRLDALRVRDAALKRGFSVL
> ----------------MQLLEVAATRKATRMTIARANPANSSDVGAATAT**RLLR**DPPKAANCALPAELLLLDLSKTPAQAADTNLLLLRLLKTHVRVAVKQSPM
> PMLDEWIYATFRARVSRATLARLRNLPSSYFSHVDQSTTKRRRVKFPQFE**RELR**EFFFQNEAKTAMADDVLLLKAHELGSV-----------------------
> QRAAASVRASMDFSGPLNALSTSPNNRLIAVGGRDVLKVVALEAAGFSEK**RNLR**VAGKPSLNFSTNDIRWHPQSDYLLATAATNGAVVIWNLEREGYKHVQERV
> ---------------------------------------GDSNQRGPGAA**RGLR**GRPDALADRRGRVLPRGSARGAQRAAAELQDVVLRLRFDHGAARVPRTWR
> --------------------------------------------MEGVPR**RTLR**HWLDDADKINSFDGPDSRKSIGRSGRREILPFARELTAFLEEGRQEGREM
> GGGPANFSGDVGAYAAQLPGGQRFSGQPSPLQALASAVALPDVFTEAEMA**RTLR**RNNGDVMKAALQLKEKHAFRSEMHYLELNVGLPTLQEEVRKGYAHLLEGV
> ---------------------------MNLFGRKSAAPARTGPVATAETI**RKLR**EQLESLEKRELHIVKKIALQLQEAKQKSAAKDKRGAIFALKRKKMYEAEV
> ENDMKRLRVAYNYDGAHGVRWAGEATAMDTEMEVPRARAVRNDYAEANAM**LREF**HYLRQLRKFQAERGRARASYLERHEEDVTMAQEPRV--------------
> SIVDTSSDDENVPPNLQRGGVHAPAESAKSSSPASTSSTGTSTPPWMAPQ**RGLR**VPKEVSNPPPAKPSTAGAAASTPPTTDPRPPATPPVTRNPRSPTTLPRRS
> -----------------------------------------MDFFSLSNA**RFLR**DAPPLNGSVIRPSCAFLRRAALIGPKRPPGLRRHRRLHSKSCANAKKDAA
> FTSGNVLMHTPDGGVGANSTAASSGLIKLTTGWARRAAQVTCLTAGAKV**ARRL**RCRKPVSSVFELLLSPSLVFIANLDSIFGTGGNYSSSSEAATGTIMYTGLD
> -------------------------------------------MTHVVMC**RFLR**LGCSEGEHPLFRREYARSNREKGVKLLRCFPHCCPEHVKRSYCGCSVHLL
> -------------------------------------------MTHVVMC**RFLR**LGCSEGEHPLFRREYARSNREKGVKLLRCFPHCCPEHVKRSYCGCSVHLL
> -----------------------------MEMDTPVPNASVETPHVERIS**RYLR**EVDRRAILLRIEGGEKQAVLAKEYQVSRAAICNLNKHRDEVLARKDGNPL
> PMLAEWIYATFKARVSRATLARLRNLPSSYFSHVDLSATKRRRVKLPQFE**RELR**EFFFQNEAKTAMADDVLLMKAHELAERLGIDATQLRLSNGWLPSFKKRNG
> -----MKSQLLRRERDEFYVAYKRMRTSYENAVEVMNDLHREISAQETEI**RELR**QARSAQDDQDVQDNQDDQDGQEDQDEPRQPQPYGTTIADLFGDTSSESSE
> MKKSFPLTRESDIPLTAVSTPSGMLWEWLVMPQGLSNAPATFNRLVTQLF**RPLR**AFAQTYFDDIFVHSRAEAGLSVMEMHLQHLRRVFKVMRANKLYANIDKCV
> QNEPDVLLARFKSNVRIARRHSTQLAPGVVYPIPEGTGEAAPNGTIGQAR**RLLR**RSSTLRGLTPTSKPWAPMLAGISRAGASLKAYNDNTEDAKEAICETPYEE
> AVAQALSAAYQAAVPDDEKDRTNLLLTHACSCDDAQCRHPEFRALCSHMK**RFLR**AACWASHSERWRSYPIATAVVELFTYHALHCQALQCDVPMCRQLRGQVLL
> -----------MRSRHYLTTQCLDAPCASGWMTLYEYGTDINFLNATSLT**RTLR**KAEEALARALNGYAPARISWPSPSCQVELARMVNKRYWNDSDKSLEFRGK
> -----------------MAAASSASSSPKKLRVLCLHGFRTNVQVMQSQT**RGLR**EALGPDAEFVFLNGPFEARGRTDRIIERMFGDTAPFYEWWGARSLEKEER
> ------------------------MGATKKLRVLCMHGYRTNAKIMEDQT**RGLR**KALAPHAEFVFLNGPIEARGPSDDVIEKLYAEHKPFCEWGSFKERERPHR
> GGLADATRRAKKAELQQLKDEHEKKEQVAQEKKNAEKYKRPRFFERVKVM**RKLR**QAKNRIEQRCLLQANDDSERAEHEKKFKTYREQMMYIYYYPKSEPYISLF
> -----------------------------------MVATAGDNGFDKGYL**RFLR**EAVLEFEDVFRIDLLADPPADVEPLRITLQPGAQPFRARTRRYGAAQSAV
> -----------MVGKLKLCCTKVAAVAVGDLPSMKKDGSSVAEGDADSRA**RELR**RRVANRRHTGAAILGSLPPVQSLSLPQLFYAKQEAHEIRMQLIDMQSQHN
> SETSSTSSSSSARSSKKGKAKHKKHHNDAAASASPQAPEPVREPVDPTKA**RFLR**EIKYTELLRSLMRAYFQLFNALEREGLVQVKPPMYSAPTIRFQHRFAAFQ
> -----------MQVKPASYSASVGASSVVTESNVATDHAIMNGPNHMVTK**RLLR**TETLPEEEEERANFEFVKKLKNLLKPKSVAQGVPAVIAKESQQLQAVAKE
> VPRPGSTTSNYGLTIQEQSEAGHGASDDQEGYEEQFAVPDVAPDEGAAKD**RDLR**RGPTGWKPAKADDQQQPTAKAATKRKSPKRKKKLHAPDSAEESLSKPQGK
> KFVFNRLPMGVSTAPDAFQAVMNEALGNLEFVWVYLDDFIVMSAFFEEHL**RHLR**VIFERLRERDLTIHPRKSRLCTDSVSYLGYLISTSGIQPLQDKAVYRYAA
> ---------------------MPAETQLKRRHYKQHEDVIKDVISEQDRL**RELR**RLRQIRYRKKKAEYANTLDAENKQLQEEIDTLEQRRRSFAAAVPAKENVW
> --------------------------------------MAELATAEKLRL**RELR**RERQIRYRKKKENYMHSLEDETRQLRDEIEQLEQRRRSVSAAVPAIESAW
> DEAPGDQAGLEVARPRDEPAGGTKEEHPSTAGPGRGQDPSGAGLRKTRRR**RSLR**KSRPGAETLQGMSAGQTEAEKPDVETLNVLTRTCARFRYREMTLANPPTQ
> FVLDEDVTGGSSSSEQLPGTASMQPRSSASVVVDPNELKQRRRQQINERR**RLLR**KAGVYADPNRARNGQRAEIACLREQLEKLQLDLQVLQKVKTKPARRPQRQ
> EEAAGDGLLPSPLDNAPAETARATQSKALTKRSSSSEEKRWRQNEINERR**RLLR**KVGIYGDSNRVRNERTREIAFLREQMERLQLDLKVLQARRAQEQREAKRA
> ----------------------------MSFLQSSAGRGRRARLRANAKK**RLLR**KAGLYDDPNRARKERKLEMTYLQRKVEQLQLELKTLQAQHPQRHRRQEEN
> TQQQRNLISGKVVVEADSIAHLSEVHTRVVLQITTLCRQYTRDFGESSFT**RRLR**ALTRRLVEWKFTRMSEVVAALSEAKSVAMQEEKQRNLEFYHVHNVNKD--
> DLTVHHYITRLDDTFERDKVSAARELGNLAKSSSKARAEVTSNAGCCSLL**RLLR**FGDDEGKQWAAYALAYTALDNEENAATIAEDGAIEPLVGLLSGTDGQKEY
> LTEIHVELQLLNRNGPLQDNATLRRTATVKKFALAVTNFVAYLQKYNDMN**RFLR**FFKRSDMEAERLEIVAEIDQLFRMLGLATSVTVMNGDASAAKNAAKFLSK
> LTEIHVELQLLNRNGPLQDNATLRRTATVKKFALAVTNFVAYLQKYNDMN**RFLR**FFKRSDMEAERLEIVAEIDQLFRMLGLATSVTVMNGDASAAKNAAKFLSK
> ------MSSSSAPLPDRVSYVEAHRLFMALENPSAAVSEPHSQAFQDELT**RALR**YMKQCVAQRQLDGVLSANERFFELQNTQLYAFCLEYYLGMLTPKQSFFQQ
> YQGRTDQEGKDEGETSPEELSPLKQAPLELEEKSATESVADQLKRLQAEN**RQLR**ERNEALETDVDNVVNQYVVVVIAATEMREMQERMRAGELRAMLRCYKKWR
> -------------------------MASLEAHIKKQVELYFSDSNLRRD**RFLR**KETKKREGGCVPFTFKKLAALTVDGAVLQAAIVDSDVVEINETKNALRRR

> QRRHRQALRDTAESLQMGRSTAHTLSMQAEQLGRSERVLDETQATVDMSK**RVLR**GMTWSGWLYNKFSAAPQLSPNKDAAEIAMGFICPECKVMFQSPEQLGTHY
> PALQSLNLSSNALADVRHLQLVPRSLTQLDLGANRLAALPPELGAWLPNL**RLLR**LRGNALESLRPLSACLRLQTLDAGANRVALLSELRFLQPLAQLRHLALDG
> -------------------------------------MTKSRDVNKETVM**RRLR**ARLDRRSRGADAEADARLRAQVDFYVRLRAALFANGGSTNVGAVDAVAVA
> --------------------------------------------MLSRSV**RALR**AGAAQLSARPAAASTTAGFHSSRAAGSSFVQHRDTEDNNADTPFDFTPEN
> ----------------------------------MPLCPVDEAQALDAAK**RFLR**SDDILDDAEDSEERAKLPAGLLKPENLKVDDLLNKNMLDRARNGNKKQRN
> GPGESLVATTRCVLKITDNTLRYGFPHLVGELAPLAGKLLGKRITLQGSV**RFLR**DNAKGRVTQLHWRVDMLSALLTLLGNLEDVERVFDGARVTPEGMVRGSWD
> TLDESRALTQQLDPVRDRDPSPSKLKHSRTDSEQNADNPSPEVHNPQKST**RKLR**HQRKKDELHYLRSQVTELEKDLERLKNSSTTHTEEQTATPPSEADVTLLW
> SSVVSALFGSYDLRNAKQWRDEDMAFREQEVQWLNDDIVRAHQWRVADME**RSLR**REKLQSEHVVCEARAEQLSTVSEQCTLLCGFVVSAMCNVGVPEDVHSYAL
> SSVVSALFGSYDLRNAKQWRDEDMAFREQEVQWLNDDIVRAHQWRVADME**RSLR**REKLQSEHVVCEARAEQLSTVSEQCTLLCGFVVSAMCNVGVPEDVHSYAL
> ----------------------------------------------MAQP**RALR**SHSYSNLDEVTFTHLHWVVALDFAAQQLKGYAEYTFHHAASAASAVVVLD
> QDYDSRMEEQVEDLRDKMRLLQVDRKSNIDLLESSKQTNKDYIRQLKNEN**RDLR**KALADLKRSTSSAGGPPGLGPSTAVGGGGLADEAEEIATAMQHLNKVRKQ
> QEYSDIVQCRVNSMLADEHRHAERRAQQQPVVQETEWKFFRRMGGLKVYQ**RRLR**GRSKQEVAAEEDFPEAAVAVERGNPSMLADGAVSGTIEDMLYGMSATTQE
> -------------------------------------MAKPRDENKTAII**RRLR**SRLDRKTLGASAEADARLRAQVDFYARLRSSIFVNGDSLGVTAFDDVAVA
> ------------------------------MALAVDAAPPARWAFLYPWC**RVLR**GKLHLEGRTAENSARWEYHWVAVRLPHAPDQADSRALRRIREALDLLARV
> RLNMLLDIVCADYITACYASRENGKRVVFKALEKLYISVHAGAPLTGADT**RALR**PAQDPLFQERARRFYDELMAEFRTRKRAAEAAPDANTPSASSSTRGLDDS
> VAREHIPKDTLVFQAGEDVWYPFSAEHALETAQQKAPGFLNQLNQLLASS**RVLR**EGSSFVPSALVLGVHMLVNFPHSENPETSLMAMASMNTPPLDELYVNALP
> --------------------------------MVMSPNQRGLDGGRVDTV**RSLR**FDGKNVIAYKEGLKAALMARKCWEILIGKRTKPERDGTPEAAARRKKWKQ
> --------------MPGSVVPPKLPISEMTVARTPQVMRLAKMTYDERSP**RFLR**HLQALDHPSKKNWTTVERICSEMLATERDYVSDIAGLVDRFLDPFAAFAD
> NRTRWAARNPRLRTSSPRIRRRPRPLPRPRRSRSPSRSPRPRSLQLLLRP**RRLR**PLGPSLKRRPPKSLRPQRHPPRSCSRSRATRSTRPAWSSTLWRPWRATPP
> ----------------MASSSAALQQSLGESEVFDLVCDYLRTRQFFEAE**RTLR**SEREQAEATGSPRAASPVAANQQGAPPSEDAAAPSLASRLEFMLERSYVT
> FELYQAELELYLGDRDAWEVVTGAVVRHGTDQAEQATFDRRDRLARATIL**RGLR**GCKNDDASKVCGMTTAKEMWDTLVGDHTQRDFSYAMLLKRQLYQCSHTPD
> ------MSEMVFAVAWKQVYQQGIEGVVEVTSYDLPSENSKVSDLVQALD**RKLR**SERVCDGAIRKVRFLQLYGTRLDDDNRLLETLVPDLLHGCPRLLASTHLV
> GGGGGNNKRKRKRVGKAKEAAAAPPSAASVSVDAQLQTLENWHRALTVDM**RELR**REGARVRVELEKVDDTAEAAVVRAEQCAKAAVQARDAALQDAQTAHARHA
> TKRRAASRKPLKTKKPRVRPAPSADATTLPDLVTLLHLQLVPFLTPRDLS**RLLR**SLARLVSAELRESLASTGLRRLYERESAHFGQRCSSDWHLLVPTEAEGGE
> RYVYSTASVATDYEDAALACLMWHCFGRSSDLGYISKQHITVSADNVFYV**RMLR**VKTAEEQGLTLVPDRDDFLTCPLHTLAVALVMQEAPCVSLLSQLPALATT
> DRFGYYLATYEGKKDAALACLMWHCFGRSSDLGYISKQHITVSADNVFYV**RMLR**VKTAEEQGLTLVPDRDDFLTCPLHTLAVALVMQEAPCVSLLSQLPALATT
> --------------------------------------------MTNLMA**RSLR**VGRSRIYTYPVASGESFDCRASGYLVRSTMSAQCAKHDSVVLWVPHGELP
> ----------------------------------MQSLVEGQTLLRDGRY**RFLR**RIGSGTFAVIMRALDVQQQRHVAIKCVRQRGLNALGEREAAALRRVNERD
> LTQRDVEAEFGKFGPIEQCAVKKGYAFVHYEQLEDAELAVQEMNDKELGG**RRLR**VAFAVSHGTQRRYDGPPPPLQAPAGQPQQQQPPLLHSPRFPVNASPNLFV
> ----------------------------MSPYDEGGQVFVCKRKSDNEVD**RELR**YAYDRVFNEKADQGDVFDYLRDGVQQVAQGFNCTIFAYGQTGTGKTHTML
> ----------------------------------------MFGARVTKVE**RALR**RRVLGAVRPLTGNGSPRSNVEMAVQHARAFSGLSAVPGRQAQHVSATKWL
> ---------------------------------------MGEDQKPRDEA**RELR**LITWYLVDRFREAFASGFCCSVVRSTEVVNHALQTLTMRAIRLFDILSEG
> QAAPDPAATPSAVAIARHRALQEQLLHFDQNAWLTKHAKQPRFQFSGEQK**RMLR**QWFDALDSDGSGKISVEELEDPMLSIGIVSDTREIAQIVSKLDKDANGQI
> -----------------MFAITCEGLADVPSGNQVVGSAIALDNNDGLTE**RKLR**SSATTTPVEDGPDDEERAAFSIKEGFSKLNNIFKKNPDVTKTLQKAESTK
> ------------------------MEALGGGVSHSTAMCWAASEGNLEAM**RRLR**EEHGADVNAADYDKRTPLHIAVSDQQLQMVDYLLQCGANAEALDRWGRSP
> AQAGQARRAVPGQPRRGHAHRPRGLQAGAHPRARGGEALRRELGHRGLHR**RALR**RRARPQAGLGPRGPGQVDRQCHAAAGQALPAALPRGAAARVHAARVARLL
> DRFGYYLDTYEGKKDAALACLMWHCFGRSSDLGYISKQHITVSVDNVFYV**RMLR**VKTAEEQSLTLVPDRDDFLTCPLYTLAVALVMQEAPCVSLLSQLPALATT
> ------------------AGPTRSSEREAVPQPRQALPPLDPQQHHGRPR**RSLR**HGRRARGLRTLLRRRGRAEPAAAHQARHVPAAAHGGLQELGRESSEALCQ
> -------------------------------------MTPAGAEKAAFQM**RSLR**PVSARIVSYGRENQSRRRPTLYVLRVSRSGAADWYVAKRYSEFRALHDAL
> ------------MIGDFEYLQQRVALRVDVMRRHVAGVAEVVLAPRAPEL**RVLR**LHARQLRVCSVRIEGVEAAFEQLNFLGDVVDENYRDLATFDLFYRGAIVA
> FRLVMDFVDGVPGFVVALVTDFERAHRGVPWSATGALPRSAIQHGDVETL**RHLR**RLSTTKRFQARPELAFDGATRCAIQFGQLAVLEYLGDTGMLLGDDNSGEN
> ----------------------GRHGPSLRYPDRRPQGGRGRHHHRQVGS**RLLR**LLHRPSAQLPHGDLLPVRLAGPSALASGHDELHHGAPHVAAAGRARRLHR
> KNLHGVVQPGCTTKRRATKPGLKSLARVMNPPAEYKKNTRPRLGLDERMR**RSLR**KSRTGAETLQEMSAGQTGAEKPDVETLNVLTRTCAGSRYREMTLANPPTQ
> PMLAEWIYATFKARVSRATLARLRNLPSSYFSHVDLGATKRCRVKFPQFE**RELR**EFFFQNEAKTAMADDVLLLKAHELAERLGIDATQLRLSNGWLQSFKKRNG
> -----------------------------------------MQRGGTRKE**RLLR**AKMMKKKLDDDNSPLGAGASTFEDILQMSDLSEQSLLANLRKRYEHELIY
> ----MEEETLRLQFGSAATTIGTQWLALQQANESSKTPHVLAFEAKGRVR**RQLR**PTPQQQEDPATWGGGVQVYDQSGVEPVTGSQQDGLWSWGSLNERNLVEVD
> --------------------------------------MPIKTRQEQYEG**RELR**NTVIAHERHEVFVEVQGAITNGTNGPLEHSDGSVCSNCPESDEYVEAFDV
> HPSAVRAHHEPRLRAARGAPHGERQVHGTRAAGLPLHRDVRAGGRPHQIR**RLLR**VGVQEGLRHVAGQQGVPGVHAADQRAAGRPRTAHAHLQDPQGRRVPPLSS
> --------MHHELFLALLGHTGDVVERRADGFFVRKEATFLSQAQRSVLD**RLLR**LGFACWTLERFVRQAPTLPGVFLHALAQGVERLLQRYADAVVALEAKTLR
> --------------------------------------MATGSMMKDYVR**RQLR**YWVDGFKDDMLPRDFMSKSYSKTQLLIPDIRLKPHRVAAVLHLPAPLLVT
> VQREEEERVEALAKLRKQSVKDKYELASAATRLDQELKEQQLQWKRKTEM**RRLR**QLPTDEREMAAKVAEANSNNNRKKGSIYAMENLRLAMLMKDTKLRQRVKR
> -----MKSQLQRRERDEFYVAYKRMRTSYENAVEVMNDLHRQISAQETEI**RELR**QARSAQDDQDVQDNQDDQDGQEDQDEPRQPQPYGTTIADLFGDTSSESSE
> --------------------------------------MRNWLMEAKETV**RYLR**EVNVSGDPERPWVASFWGECLCLPVVSDHQARLIDFNVLWLEDGSRDYVS
> --------------------MGLCDLPSDLLAATLQFADHAALQRLEAVN**RHLR**EVIVQCDTWRILAQRLALYANDCASRSSVQWKHLALMTEIPVGEELSLLR
> -----------MPWNANNNPAGDIRAPAMAAALRLKQQQQGATDVAAAWT**RSLR**VEELDGLELAGDQLSALRHCALLHPDDFPAAAAGPDGAAQIHPRLHAQFV
> -------------------------------------------MVRVVSC**RYLR**WSCSEDDHPLFRRHYTRSNRDRGVKFLRCFPHCCPEHVRRGYCGSSIHVQ
> ---------------------------------------MATSRTQEDRR**RRLR**QLVLEAARARANDALTLKSEDARQELFAPFSPSPVGVIDAVWTKLEDAQL
> ---------------------------------------MPADWFATSGA**RELR**DNSALDYREYVRRSEQCASPQFEEDLRQIELDLPRTGESMRLFLLRPEER
> -----MAPPTAPMKQFSYELEGTATATHGAVRMGHLKSGFTPDMTLFKNL**RELR**RKFDPSGTNKLFGWRKADPGAGKRGAYEWVTLNDFLPVVEAAASGMSHIL
> -----------------------MTLFPLTKKALSVRKDPQHVIWWTRTR**RRLR**FWSGLRLTSRRWARICGRTTSVVTFSVRPQNIENVSVHNAYIALYPNIYT
> GRGSGGKRRGRNGRSGRGRNVTSLGHGCFGHLKGSLDPIEAAARKLALLP**RNLR**SEIRNVRRGEIELVVQSITIGVSSREIGLSACQLAIQILDLGGMQGYLIP
> YHFNQLAKLKHWNAEDKFLNTTILLEGDLREAFEDASITDDDVRMDEEFT**RALR**KASVVVLPTDYSEKLQEELWEIKKARNELLSEYSKRFRALVRMEHTLALL
> --------------------------MEKLLTKIGAPSYLPTPAEVLDGV**RWLR**ENPAIAAGVVVGLTSYSVAKYYEYGEDSDESDEENDENVDHQDNAPRASP
> -------------------------------------MAKSRDVNKEAVM**RRLR**ARLDRRSRGADAEADAQLRAQVEFYVRLRATLFANSGSANVDAVDAVAVG
> INMDPDVDYQCIAGSLQYLVSCSRPDIANAVRTLGKFLTCYTKKHFVLAK**RVLR**YLQGTREYGLVWNVTVPWGLQLAAYADADLGNEKDDSRSITGYVLQLNGC
> -------------MLRAARQLSRAVSVAPLRVRQPSLQLLAAAASQLRGA**RCLR**APSCCSAPFRVNRPLQARGFSAAAAAEDARDAAAEQQQQQRVKWPSDVAR
> -------------------------------------MAKEGINGVFGKP**RPLR**GKVGKTSDRLEEDAQAMEARLLQLRVSMMEEKQKRDAELPLKHAGNRWRS
> WCRGLVERIEHPMKKLRKLMEREEAKEVYKVYSTIMASMQAQSKLKRAPL**RQLR**PSAREIKYLRLLNVEVPQSTLQTSAKEEMFHKCRGEIMYTVETNNAMIEG
> -------------------------------MRAVAKKLAHKLREAQARR**RNLR**FPFHALGQRFLRDLVSLTSRIGWDSVDVPIYSCWNRVMEQLWPYYGTDTC
> ----------------------MDELKINEESTRAGRPRRLGSNAVTHEI**RMLR**AQAASMEDELQALQSKWRDQLQDGRILATAQESAREKHATSQAEKVREDL
> ----------------------------------------------MHEI**RILR**TMVTSMEDELRVLQSKWASQLPDKRTLASAQRCAVEKHAVSQAEAIHREL
> SRAPKKRRRLSEVPETNEELIQALTGATCSRSRGRPRDPTKAPLTATHEL**RMLR**AQVANMEEELEGLKSKWSKELPEQRTLSTARHSAREKYAADLSERAHNEL
> TDPDSYTKKRARPVQTHDELIRCFTKAPETRRRGRPRVSNAAPLSATQEI**RRLR**AQVATMEDELHGLQSKWAEELPDSRTLAVAQRSAREKHDVAQTEAAHNEL
> SNANVMDRDVKKRRRSSHSDNDAIVNFNGANPQSKPKTAKKQAVSAANEL**RMLR**VEVASMEEQLRNLKLKWSSTQPDERTLAAAAIQTEATRSELKRTLLEQQI
> MSRDPEPASKKRRRSQEANDDVISALKGARRPRGRPRVANKTPLSATQEI**RMLR**AQVACMEAELHGLETKWTRELPDQRTLATAQHTARKKREVALTEAAHMEL
> TKKRRRLSPTKDSNSTNNNEELICVLTGATPAPPRPRAANGAPLTATHEI**RLLR**AQVAEMEDELRALNNKWAAQLPDQRTLAIAQRSAREKHGAIQTEAAHNEL
> APAPPAREVEARHLASRANEAPQQQQEEDFFLSGLSKTQRKPAPSATHEL**RQLR**ADAAAMETQLASLCAKWQEHLPDPSVRESACVAAKAKWVSSQAEMVNRSL
> --------------------------MRVKTDDGMASPNRNVLEATRPPG**RSLR**AAKSSKALACLLRAGKLERSLLVSVMRSQSRREAILESPAADEDDTKVPV
> -----------------------------------MSANDDAKGMIAQEE**RELR**RVFDHLSSYRQKKRLAHTISDCKERRQRLESSRNNPEVSALLNEKGAKMT
> ---------------------------------------------MSGVQ**RFLR**LSAAEAEAAMKPRLVDGKWKQPLISGRKIAMVKKHAFRNGLVGTWEEGKG
> PAADGREDAQGEPQGRDRLPDVAHGLRAHHQAGLHQRQQARDGGARQERA**RDLR**GAGLPLLADRDGVREAGQVHRIDSRQTEDNATEVACATRVDDRIYRGAD-
> MGEAGATAAAPPPAVVTSPSLTPAVAADAMRVPSSSPAFTSSGGGGGRSG**RNLR**QKVVDEIKNVRARKSPASKEKAAAKEKEKTVAPPPLELNHEDADEEEDDV
> ---------------------------------MLEVRLVATGAVVKTEK**RMLR**VRFTYEDQEYVETFIVLDLDDKFDIVLGMPWLARHDPVIDWEKRTLVRLG
> PMLAEWIYATFKARVSRATLARLRNLPSSYFSHVDLSSTKRRRVKFPQFE**RELR**EFFFQNEAKTAMADDVLLLTAHELAERLGIDATQLRLSNGWLESFKKRNG
> -----MAPSLASPPLSPSDESVDLECGLNAPHARVDRRHMERDLVLAFDS**RELR**RHEAWFLVETAWLDSWMSYVLGDPDDANAPKRPGPLTNDSLFDREEFRLR
> QRSIGRALPAPAAPHGGGRRPVRRGRGLQRPQPAAHAPGRGAPPRAHGRR**RRLR**AARAARRDVRAQGAAAADAARGLVAARAQPPAAGVRPGGRHRRGRLRPRA
> ---------PRPHIKRAPRTQTATPHNQHTSHQTQNHGGRVRYARVLPVR**RALR**RLLRLGRRLGRRPPPRRPLLGRVPQRPARVRAARRGAAAAGLRPTASGAA
> NGRTQVLARSCSHNQICLPRDTRPRARQTALQYYDGGQAGRLRDPAAAGA**RALR**GRAAVPAQAVGRPRGRQARAAERGERAADARQQQEGARERGARARAVPPA
> GSEEYDHNGLSSPIPQEVRFMNAELRRCPVEGVGMVLAIFSGSGAVYEFV**RTLR**KCIYGKVKHAVRLQMTPNGTFVRTQFEVAIKVMSKVIIEEGNLQENPLVE
> -------------------------MGKVVFWDDTVMMRAQSDEDVTSLA**RYLR**DGREFTAALEVLFEKHQFAEAARMEELRSKHRRRQRGPDELRSSLRAVFA
> AAVAAVAAHDGQGGDGLLRGPVRAHVARVLPEVQVQVQRRPAQRGRDELH**RPLR**RQVHAGLQLAGRQDGAGGEGDHGPGGRGRRRAAVEHVSTHPVTHPPRQEI
> DLRESRCLNSAELLREHWRWEQLYLQAAQAMDSRQDDIADQVRVTARALC**RDLR**ESPVAVEVLYHKGTTAHDRSEDLQMLVKALSELTDLTHAQLDKTLEDAKS
> DEALAGYFRVASTASTSSKRGRGRGRRGMMAPSAENDNSDTPETCLKHLK**RLLR**DSAAPVQSHCIKKLHLTVLCQLQRGSRPPTVFSRAVALCDEIFKRSVVFR
> KGYSYSRIAREQEMARSTVRDIIAHFKLNDHCVVAPRQGRPRLTTACDDR**RILR**DTETNRFSSAAVLAVAVSKDTGRVVSPQVVRNRLNAAGLNGCSARTKPYL
> DRFGYYLATYEGKKDAALACLMWHCFGRSSDLGYISKQHITVSADNVFYV**RMLR**VKTAEDQDLTLVPDRDDFLTCPLHTLAVALVMQEAPCVSLLSQLPALATT
> RVAGSGRAASVATSSRNRTPPNNSSARGAMNGTAPPPPSSSSGDAARRRE**RALR**RWNLLRTHVRVTRRLSQELLLKHARRFSSAQQTLVDACVGCISAPASKDC
> FDALWGSLEALARDVKRPVSSSSQDEEKKPSLLAKWLAGVVDHYAFRQII**RDLR**AQEKETMSTLNVLLQFLVARVSALFVERRLLHCGSCQIGRQLVKLVRDFL
> -----MKSQLLRRERDEFYVAYKRMQTSYENAVEVMNDLHREISAQETEI**RELR**QARSAQDDQDVQDNQDDQDGQDDQDEPRQPQPYGSTIGDLFGDTSSESSE
> DLLMALQSMEPCGNCGAEGAKIPCMSGCGEVFYCSRECNMHHASAHRLRC**RNLR**CSKFNDSESESDSDDSDEYETDSSAEYETDSDEDEEEEEEEEEEKPKKKK
> ---------------------------------------MKDFKVTHQQL**RELR**VTQWQTASELASQFAAKQSETLVPTGTIGLVDHREHVYECISLTNNTMRR
> ----------------MYVAFATCACNPVTLAKYLDKYTHENYVVAKHVL**RYLR**GTTDYGLAWEKVETPDLHLVAYADAELGIEMDGRYSITGCVLQLNGCTFA
> HAILKEEKEALLLEIDTLTARLEDLKTKTFGSPDGEEERDRRCKDKMVEN**RLLR**RAVQDQQDQFTSVHALTNEYSLTDTEVGSALHRFIHLDKGESSRRTALRA
> PPPRLNVGVFSKRKGVRYHRDGQVRSCRFCEILRTKAEPFLYEDEHVVVF**RPLR**PIVPSHVLIVPRAHIRNVNRLAARHRGLLARMHRVAEAVMAKGSLEECAE
> ---------------------------------------MAGAREKRRTA**RILR**GETTMWKAIWTELKAIHSKEGAQALSAACVAKAPEEDCQVDGACVLVPQA
> -----------------------MAEPKTEAEPVSESTTGKAIANSSEKI**RSLR**AAIDAKLHEQGIYEQIRDLVHLKTKASTDGNEDNDGQPNSGEDRVADDHA
> ----------NDVHDGRQRQRADGLDPAPALEPQEQPLPLQHARGGRAGR**RRLR**AAAGVRARGLAQRVEAAGQGAPPPGGDGARQPPHPRRGELDASARDRQGA
> -------------------------------------------MGSRQIG**RTLR**RPRRRSAVNGSASTRSAAGAGPLQVIGENTVVELTADDALPAPTLTSPSP
> SPPPMLSRLTLLRALSAHGVAAGGNARLAPAAIPSALSASFSSPAANRRA**RRLR**RAKASAKAATPDPRTPSLLADIEQLSRNGLAQRVEMPSELLDRLTSVVRS
> RYLPPGSSHEARQRRELDGLRRPGHDDSPVLALGLHVALEAAARVRQALP**RPLR**ARRRAEPHDVGGRDQRAPQEAQQHRQALLRARVCQGQGRGHVGRLAVPLR
> ILEAHSELISRNKLSDNFYSKLHAALDAMKVNVILGERLTERLPGNSFEK**RTLR**TDKGTEIESDIQLLCGGFHPVSDLVQDMDPSLVTEQGSIKQPRDAQDGVL
> LDGVDFMGGVGEEVGLWGEVQTQNLKSTSGRQVVLGERLTERLNGNSFEK**RTLR**TDKGTEIESDVQLLCGGFSPTTELIQKLDASLVTPEGFIKVNSKLQLDSA
> -----------------------------MPGERGAKKSGRGDGGRDEAS**RQLR**RVQGMEPEEHKDLETVVRDARKVKKAARESEAVQGGDVEASAVQGQLAAG
> SKRKFAAKDAQRVWTRTAPISIPTSGRRHPHPHLCASVVESPDLSYHADM**RLLR**AGRYLHEHEDDLDGSEQELDEDDPAFYGNLAFFRSQCGAQPRVVRRSSHP
> DGLQAHSGNADQSPPPSEEGEEDREDIHGPPLDNMNPSGRYVDEELAEAT**RALR**NSPAIKLTKLQIKADYKAWKSEVPLQIETCTLGDITYGTERVDELRDSMK

> ------------------------------------------MAWNRKCR**RNLR**SATINGEDEDEEWTAGGGSTGNSYSYSYSYSYSYSSDRARCVTLLQLTRP
> PEEEDEEEGEEMDSTAERRGSKLLEQNEGEQEDDDVDAELDEEEAARQEA**RRLR**TAIAEATEARDELQQRNTKLQRRIAALLQKNQENSATTSSSGTATAPGDA
> ---------------------------------------------MTSKN**RRLR**STSVSFGVGSIPRPNFDLGPAASNANNAPLRRRSSSPRVPPPWRRADSVM
> -----------------------------MYSMFPMVEKSYRVNITDEIE**RDLR**FLGQSRVDALTSPFAMKLLKEKGGVKLYELCEKEFYTMKAVTTVHAPIQE
> -----------------------------------------MPRVPEELA**RKLR**AAGQDHVLKFDDADKLSSTEAQELTTELEALDLQLLHDIFQASTHAQTTE
> -----MTDEHHVEEKLRALVRSWGVSTFSERIALQWLERFLGSDKDCDDF**RRLR**KWIVLQLNQRRVPDDCSKWQRGCPEVIPLLRATPVWDCSEFPWVQTLESN
> ----------------------------------------------MASQ**RRLR**YINKVKAERVALKQQEQALSAQLRDLQQAQTVAKANVVENVALGAWKAVT
> KGSSSSEEEEPEAVDDGADGNADEDADEAAGDEWGLPLQRLTWEMLKTNL**RVLR**FSAMYMTKTK----------------------------------------
> QPLLQHGRQTPAARARQGPRAPAQGDAHAREALGGAAGGAGAAGGRARRQ**RLLR**RRQRPRAAHRLGQEHGLGAGQEARVRVARGLWRHPGHALCHQAPGHDQRG
> ---------------------MSWLQTHEVAVPFYEVRAGKQVFAIQLHE**RALR**QEELARNWSSITSTSSQFLLEEEDDDENNSMTGNTSGVRVSAMRSYSQFR
> -------------------------------------------MLAADKL**RELR**ALMSRTNVRLLPLRHHLGKTEKGEAAELSGRALQAFLVDTADAHQSEYVG
> WCRGLVERIEHPMKKLRKLMEREEAKEVYKVYSTIMASMQAQSKLKRAPL**RQLR**PSAREIKYLRLLNVEVPQSTLQTSAKEEMFHKCRGEIMYTVEPNNAMIEG
> SAAAKLKQNERERLEIERLEARIAEQTPARGSQLGEAASFDLFPLSEASR**RGLR**TCGFTAPTKIQVGALPHALAGRDVLAAAKTGSGKTLAFLLPVLEKLFRLR
> GIWSLEEHDRFLEAMKLYPKGPWKSIADHIATRSVRQVQTHAQKYQEKVS**RRLR**GLRKSKKKLVRPEHRIDDDTMELCKLVDCAAEKLVAASKKSSSRASGPKL
> SEQEHEQFLHAMKMFPTGPWRSIAAFIGTRSIKQVQTHAQKYQQKINRRR**RGLR**KQKKKFVRPEHRVDAHATGCIQRVKNFTMQGSPRSPSAFPVGAMSPLSGS
> -------------------MAPATFDEDPDALYRGVPPPVPRVRPISSSD**RLLR**PTKASLAASREKVLTGKDKELAAAREVARKRRRLARVAKENGRLTIPKSP
> GRGLLLQRALVPAVPPVHALPERRVRRHDRGAPGVRAHLHLVGPRPGPVQ**RVLR**RDAVPGAALRGARRQPGHEHQVRRHGHPHAGLRGRRGQGHHHGRPQRRRQ
> -----------------MDDRIHFGVAPIWISERSLSKVPLRIEDFFSKR**RRLR**SGPSMGNVWSYYAGDSDAFDKAACLPTGDACSAFYTLFRRSAAKVVLQQE
> -------------------------MSLTVRSLWPGRRKPRKETPEEARQ**RYLR**EILRRELEDKKRVEREMHNLVKQINALILKDRKSLVAYQRRVKDEDPRSV
> -----------------------------------------------MPA**RSLR**NLPAPAQASPSIPQQTKPPAYASALPSNVGVGIIGAGLVGTWAGFRVGVA
> --------------------------------------MGEEGPCRGRLF**RLLR**LVRETQCELEVVKPVLDELEVLRLCCSFGMMPTVNPMEFDFDVLHASEFD
> GFGCHLEILTSGKEHPFEVAFDEANRISSERFTKPTWLWKFQRFLNIGNE**RRLR**EAISVMNEFSVDLIMEAMEQMKNSKPDEADVESPAHKNIMAILLSKKEAV
> ----------------------------------------MWRACRVAAL**RHLR**TEAARMPVARAPLALALQQQLGSATCHFTSIAGVDRELLLTRSARDLRDE
> ----------------------------------------MRGLGRLALG**RALR**RPEALARHGGAAVNALQVQTRAFSRFRAMDALYEGQDYSRQVEKKFSELS
> GLWSGEEHDRFLDGLKLYPHGPWKKIASYVGTRSPRQVQTHAQKYYEKVG**RRLR**GLRKDRKKLVRPEHRLDEDMVQLCQLAKTKKDPSGGVDPVLASRLRAPLS
> SEDEHDRFLAAIREFPRGPWFSIAKAVGTRSVRQVQTHAQKYYEKIMRRG**RGLR**KDRKTWPRVEHRIGDDVLEFCELMKQFNKTKSEPLPSTPKRSQRRSKKSS
> ALVKCRNLQKLTMVNCNLEVISNLEPVGASLTHLCLSNQNLTRMSGLSSL**RQLR**HLYLQQNRISRIEGLESCRKLKTLWLYDNCITRVENLGFCTDLRELWLQT
> --------------------------------------MGEEGPCRGHLF**RLLR**LVRETQCELEVVKPVLDELEVLRLCCSFGMMPTVNPMEFDFDVLHASEFD
> QLPAEDTACTFCGVSYFVFAEVQTLQSTVKQYKKVFRDFVRFMERERSVA**RDLR**QQITELKDSFEEVVATCSASTKQLGDESEIQRAAKREALDQLQRVQRELM
> ---------------------------------------MVADKKTQRHW**RVLR**EALLRSTSSGAVNGATASSISTEFFPLFAECAVDATRVRIHSPAQENFVW
> QPMVIVMVKTMQLQAQCYEAMLRLPGASVKVFDAATECWEGIAKGSGSSR**RVLR**ATLPSANKAESESIRQQLAKLALAQQKRVVETIPELKNLFIQSSSKKQSM
> DATPLNVLEFEEYAREYLPKASMDYFASGSDSMETLQENREAFKRLVLHP**RVLR**DVSNMNTSTTLLGHNVSSPVCVAPSSTHRLAHPDGEIASSSATAKADTCF
> YLSKYIEDIQLEGLGLFGGDLVLNDLEIKRHVLRESLEIPSSFDFSRGFI**RELR**IHIPWTQLLSQPIEVKLYTIELILTAKSGDEQATPRAATRAGADVDAPPS
> ------------MPLIRADKTKTITPKDFDEFGQHLDPPRVGRTVKKVLT**RYLR**GDLWDQFGPAAKPEHEPVRVNREIRRLRVERPEITLPEIAKALRIHPSTF
> -------------------------HAAKARNDDGRTRWKREDHVHDGRG**RLLR**RRHRLRRVRLGHPQAWRQAAQAVEPAQAPRHALARVRCRRRHLHDGRVRD
> ESVRVVRIPGVRRAAAVPSADAGRQRAHDQLLRQGAARDGLAHGHRHERR**RELR**AVGRWRLLLLPGAAARSVVRGGGCHHGGTRLPPARRQGRRQEDQGGVTC-
> ALFQYRRRQYPAVAPKEPFVEMESDDEIAIDSEDLKVMRDSVDTMQQQLR**RGLR**DIAEIRELVKQAAAQREVEKRELSTRRRSPYELDSAVSLAAGAQRTRKKK
> SVLSAMTVRHHDENLVTQRPAATLKEILEKIRANKDQIRELDLKDMAAKK**RKLR**PAGGDLVGRVFQLNRTVLRLLLPDHDIEDVGAKAMGNMLRANNTLQHLDL
> RPQARDADGNALMSLEELKEGMFQSIRDNGTVELIRAQLRRRFIEKLQQQ**RRLR**DDQDGENNASRANRSTQSLTPDEKLVHGLVAEYLASKGLENTLAVFVPEI
> -------------------------------------------MADERLD**RCLR**RGDVPPPWGNLDDHMVYPDEDADSTVDDIENDPDYQPPTQEPEESASSGS
> -MLALQRHAARSGAKQVARFSAAAAATAAQDAFADVLQLALRETEGSRAS**RRLR**KQGFLPGVLYGEGEDGKDERVLVSFPTRTFERMHRKLWTSIENQVFQVQV
> EVVIEVMDELQAAREFGFQPLTEQDLTQIIMDNERYVSHVKNRLLSVGAQ**RELR**TRCMQAFGWDIVQRVEDSVLEFVYTKRFHGLNVLDVMKKSWTNGLILEEF
> --------------------------------------MGEEGPCRGRLF**RLLR**LVRETQCELEVVKPVLDELEVLRLCCSFGMMPTVNPMEFDFDVLHASEFD
> PVESTSVTQVTEDTVSLLRAMGCEPRTRPSEVSIKSWTPGLAGKELHKWK**RKLR**LSFGESDLTLGRPPSARPAREQADPSKIPLPQTPKKSAERSNQSQATDGV
> ---------------------------------GAARRAGELAAVLRQHP**RGLR**PAGRALLRGGGQARAAGDPGAQRRLRVARGHPRDGHAVADHPAAQDVAVG
> -----------MAKRFWGLKKVDYLGHRVSAGGLEAYPKDLQFLTDLPFP**RSLR**AMQAFPGSLNYYGRFSENLAVYAAVLYELREVDFAELRQQSIEKQSDEIF
> ---------------------MDDSATAGDRRGMWSMIKEGYEGLVSTVI**RPLR**AQYAPSELGPKRAQIGDVSVQRVDLKLKNPAGYTLECSWWKPCAPKNGQT
> TAREHSERPVASWSSEGTITTGHGASDDQEGYEEQFAVPDVAPDEGAAKG**RDLR**RGPTATKRKSHKRNKKKKLRAPDSAEASLSKPQGKDAGRQYTVEEVRYVV
> ----------------------------MSSPMITSSAVEEADITELIEM**RMLR**PELDVGLDPRDSSSYGAVSDHGNNVAAVTFESKKALLQRCREFARARGFS
> ----------MTTSRDGEPETGANIHRKRPREAPPQWSPDASQELLRLRF**RQLR**ALFDDAETVADVHEAWGVVSARLNDTDGLLDAQVDAVKCSDQLTRLRQQW
> --------------------------------------------MRSALS**RPLR**RALRAQQPRWISDVKITRDTGLPDLIITPVAAKKLIEAAERQKTENLMLR
> GPQLAPTTQGRCPLHQLVHVRARTTADARHLGRLRQDERATGLPGREGLV**RPLR**CGLREADPGGAARVPGRRAGLRQAGQLPHVQREPRCQGAEQDRS------
> IVLSLHGDLLDRFKSDIETANPAQVWAALLRTYDGTRGTNAVYLKQDLYA**RRLR**QGEGVTEYVGDLQRMRRELERTEITLAEGEMASTVLSTAVAVYLSIANDH
> PPSQRPRAPPPPSQAGAGAPDPVEEGLRSLERWRKTAEYAEEGASVSDVM**RELR**EDEEDEDYEEKEAVKTETATGVQPVTSAGMKTETVAEAKAEPAVKGEPEP
> AVPDAVLPHARGRVAVAREVPARGPVPPVEGGLVPPRGRGDGGRGGGGRA**RLLR**GPVGHARHAQRAAGQPAAARHGRRPRRGLSAAPDGRLPGGQRGQEAAKEN
> --MAKGAKAEDAPAASAEVLSVPILETVKAAQLQNGLRHRDFQRYRQYCT**RRLR**RIRKSVKFTHGKGKQFVNKKVDAESATENRLLYLPLYNAERAWGYAMQLK
> VVWQLKSESCAELRAFEARRAAQIDKFQARDRAYWRQFQDEIRRAGDENA**RLLR**FFALRMQADLAYAEALRQTRAALDAPASEGSAGSDTEQLSVQSSVAKALH
> --------------------------SNSNAGSARGRRVPVVGVVFRLHG**RRLR**ARFRQPGRRVRHGQVGRGHRVDGRDAAGAGHAQHHPRGHGRCAGHLRADR
> ------------------QSSDLPISSNSNAGSARGRRVPVVGVVFRLHG**RRLR**ARFRQPGRRVRHGQVGRGHRVDGRDAAGAGHAQHHPRGHGRCAGHLRADR
> --------------------MELQRHGLTSAAPGASVHRYLNEAGEKEVL**RVLR**EQHQRHKRVTSDDVRLAARAVASQGGSVAIPPDFPPSRWVLEFKRVHGFV
> -------------------MTDPRRLHASSAGAPVPVRRYLSEAGEKEVL**RVLR**EQQQRHKRVTSDDVRLAVRAVASQGGSVAIPPDFPPSRWVLEFKRVHGFV
> EAPAKKSKHRQQREPESERETQVVCKWQERWHGICADCFEQVQHCVHYPE**RRLR**LLIVGHNPSDHAWKTGYSYSNPTNRMWMLLTGTLSPHSWEGVVPATASIM
> ---MGLENFKSYGGAHVVGPFQRFTALVGPNGSGKSNLIDAVSFVLGVHS**RHLR**STQLKHLIHKAPSDDDTTSARAAAVTLVYELADGEAPPSASQKKVRFTRL
> --------------MTPANSDSGDSSSATSMEQVHAAWLAAGAQGDVETM**RRLR**RLHPQWLDLQRLVNTPASGAPTRRQFCSWESFHLSTSGASTLLTAAWGHS
> ---------------------------------------MLSGSAATAAR**RRLR**PAACLARGVASSAAADAPEEEGVLTREFIYASLYAKEAGYFTTAQREVLH
> SKHFTPTTDIAARIARALFTDLLVAEHQLLRSQQRSAWKVVNATARSARR**RQLR**AAFDRLASSSSAASRAGKRRFLTPKHVANARAFVDKLTSVLLRSGFQRWK
> ----------------------------MSTAADSKETKTGGEKSESSLR**RSLR**VAGLPAEVVPTESETPMARRSTAGSRTTSAEGEAASSGEAASSAEPEVPP
> SEDEHDRFLLAIKEYPRGPWGSIASAVGTRSVRQVQTHTQKYYEKIMRRV**RGLR**KDRKTWARVEHRIDDDILQFCNFMKSMGSNKVMVTSPSALKINAQVAQAV
> LAPASAAFYARLIPQTSDEEQVAAALAALETPGEGMSPTKRSRNHAVSAA**RTLR**NAVLKEARFYRVLEEAKLYSIDFGGLQAGDGDQDGGGDDQDAEIDDMRRR
> LVRLAGALHGPAPPHGRAQHDAAGRGRRLQRLPVHQGQDGRAGGLRLQPG**RWLR**YDGARDLPLPAAPALPPPQQVRPALEHGRGGRPQPGDGQALPGPGHHDRP
> SALQARQVTPVPYRPALLGLVERFHRTWKDMVALARHSGTGFSPNELMMG**RRLR**APNELLRSSGVTQVGPFADYHRALVDSMQRATEAAKAALAKDQRRRAKYY
> DVALAPNSSVILEPADSLSFHLQPQTAPQAVLTIRNVADDRQIAFKVKTT**RPLR**YLVRPNQGLLGPNGSASIMVILQQKDCDELLRLDPAERQLANDKFLVQSI
> KLLKKIIKDCAQIKKEEKLQGDKLVKIKIKPSAKEDNDSIRQSQDEMNFF**RTLR**MEIKKIADFFIKEQARHTSQVAAIDASFQQLKTNPDSAEAKTALMKSCVA
> GTRQGFAIYNCEPFGKCFQEDIGGIGIAEMLYCTSLVALVGAGDQPAFSP**RRLR**VWNTKTGAAICDLNFVTAVLAVRMNRQRLVAVLERKIYIFDISTMKILET
> YPDATLSTITRQPGISIIKYTRAQVTGSDALRVIESAPLGNDLGGHPELV**RLLR**YLGAAVFINGTFEMVPKPSTQCLIVMVRDPGVDLYVPAMYVLMDSKQQDA
> --------------------MEQKVQELEAEKERERESSENIQRFHAGQV**RDLR**ATLAQVQARRATVAPDMATNVRSSVAQNPEVATRPQESSGVINGNASVTG

**END OF 4th SET of 382**

> ---------------MSKKKSEEYLKQRESGFNLSGVHQERLPQYNALLD**RNLR**HHFESRPLQSHLNELGLIDQRGRIVDLDKQKSKLFIIDQEFKLAEEAERK
> ------------------------------------MLSPGSSRPSFGSA**RTLR**SSRESSVADVSISVSSSASSNSSATVTVGSGASNELATPLTAKSSVVLVY
> ------------------------------------MLSPGSSRPSFGSA**RTLR**SSRESSVADVSISVSSSASSNSSATVTVGSGASNELATPLTAKSSVVLVY
> -----------------------------------MALENASLPALLNAW**RTLR**RHGVQVRLHRDAERNSQPGGALVTIFSFQYLNGCSVQRHVLSVVTEPCGS
> PMLAEWIYATFKARVSRATLARLRNLPSSYFSHVDLSATKRRRVKFPQFE**RELR**DFFFENEAKTAMADDVLLAERLGGRGGTGRNPQFNLPLL-----------
> KRQLASTLNHLVQQKAVAALYTPTEVERIKQMTDMIEQATSDFEADEDWD**RILR**VVDALSNVSNHAVLKESIRYLKLRLGDPSSRVVILALTLTESIVKNCGDL
> AGERGAAPQAAASTGSTSSNNSVVGLNGPKGYTREELINFWRELSLVAKR**RLLR**IRRETYLTALDQFLVGQNLCCECHDNVIAEWEDHERKRSGSRSLQDVFSV
> -----------------------MQLPARDDEPVDLWACARTLEATPALA**RLLR**DAAPPDDRNDLGETALHVAAARGNDEAVQLLLRHGADLLAADWTRFGKKF
> -------------------------------------LPVQDARRSDHDV**RHLR**RLHSRSQG-CTRTRAT-RLRVRHVLRLRAVVVRHRQASSQRAVPTPGWQT
> TAAPRAVLQSSGWQDDHRYSYDGYDPEVPVMDPQGEELLPRPSLLAFRPS**RMLR**VSTQQHQNAIYKQQLLRAIGRPSDDSSGRPTGTSDMSDDDWYERSEAGSS
> -----------------------------------------------ELR**RRLR**GVRQRRVPGQWRRRLHVEPAERQPGHAHQAQRRRAEHPAREHQAAADAGR
> QGGNSTEKMSSGATTLKRAIDSNKGADTENEDNESGRQSPPTQEGDNFTQ**RVLR**KQRSISGRILSTQEALAMPVSRQRPTSVRQLTTDHIGSNHFVREGYGGLE
> TAREHSERPVASWSSEGTFMTGHGASDDQEGYEEQFAVPDAAPDEGAAKD**RDLR**RGRKPAKADDQQQPTAKAAAKRKSPKKRRKKKLHAPDSAEASLSKPQGKE
> -------MENHVEHLRAVLECMRQNKLYANIDKCNFGAEEIPFLGCFIGK**RGLR**ADPVKVKAIVDWPVPKNQKDLRKWLGLANYLHRYSENYAEMARPLSNLLK
> -----------------------------------------MSMVELRDS**RFLR**DVAPPLRAPVGKAAAESPEGTTASSDDEESNNHSHGSSSGCGRPPLGAKA
> GSPTACDECGKKFKVFGSKKKCKHCMDVVCKSCLAAHLELKHTHLNNPAR**RGLR**RRKSLELFDAAGRHPDEQEELQFLSPIGSPDLELEYADSGSPDVLEGVDG
> AKAAAGTTQPEIVLEGSSVVNDVEMEEGEASSSKRKESIDSDSLDPACGS**RRLR**EGDDSDASSSKRSRGESDTPLSAAVALSSPRDVATSSSPRAASVARDPWM
> --------------------------MSRSATLLFAHGGGFCKDTWEPIV**RRLR**DSSLLQSVSTRFVSFDFKYHGSNRDESVAPQLDLKNPRGPRVHHPAADLT
> ---MATIDETVALPKYQCERYEASLKASDSLLAKDRELFKSGLQEYNRNM**RQLR**SLLVNQARSQPEARELVRRMGDNVLPGAIDAFLEVFDSAYSAPILVNPRL
> YQGRTDQEGKDEGETSPEELSPLKQAPLELEEKSATESVADQLKRLQAEN**RQLR**ERNEALETDVDNVVNQYVVVVIAATEMREMQERMRAGELRAMLRCYKKWR
> ----------------------------------------MRTSVHVSSC**RFLR**LGCRNDEHELFAPSYARSNKKRSSKLLRCFPHCCPDHVTRSYCGCSLHLL
> DCVSSLVQEFNGEPFLLSAPGRPDILVVSTPEAFEGVAKRQFDTFVKGDV**RALR**ESITSTIQKHDRTLHTLFENAAVSGESFDLFRLLSRFAMESFAKIGFGIQ
> SVADVKDLLDSQSTLNGLQNAMKQVESAAAGEFATKPQVESINRQLQAIT**RQLR**SEIYQARYIWKDGPPSTKQTIQWSSQVVNTNADIFLWQFGSDEVRLLLPG
> -------------------------------------------GLRFPYP**RPLR**RRRTRHRCRRVPGRCVGCPHYHQGQQVLQLGDRHGVPHGVLPAPQQRRDG
> PMLAEWIYATFKARVSRATLARLRNLPSSYFSHVDLSATKRRRVKFPQFE**RELR**GFFFQNEAKTAMADDVLLLKAHELAERLGIDATQLRLSNGWLQSFKKRNG
> ----------------------------------------MSNQLEDDLR**RVLR**LARAEAKANKLKNGKKNGARSTNQQKEQRHRHDRYLQGAKGDRSTLLTAK
> -------------------------------------------MARVVSC**RFLR**LSCSEEDHSLFHREYARSNRERGVKLLRCFPHCCPEHARRSYCGCSVHVL
> AVPDAAPDEGAAKGRDASRGPTGRKPAKADDQQQPTAKANTKRKSPKRKN**RKLR**APDSAEESLSKPQGKNTGRQYTVEEVRYVVARTVLFRLLELDPISVFLKP
> -------------------------------------------MADERLD**RCLR**RGDVPPPWGNLDDHMVYPDEDADSTVDDIENDPDYQPPTQEPEESASSGS
> -------------------------------------------MALSSAP**RKLR**LADPHAELPIKNHKYVKNCTELYMANKRIDKLANFDAFVNLEVLWINGNQ
> EVAGEIKAKYPSKTVTILEGKDKLVASYDGVKVVLGERLTERLNVNSFEK**RTLR**TDKGTEIESDIQLLCGGFRPTSELIQKLDASLVTPEGFIKVNSKLQLDSA
> QLLLEEEWRVAMRSRHYLTTQCLDAPCASGWMTLYEYGTDINFLNATSLT**RTLR**KAEEALARALNGYAPARISWPSPSCQVELARMVNKREPLLTHTFGFINGK
> -------------------------------------------MADERLD**RCLR**RGDVPPPWGNLDDHMVYPDEDADSTVDDIENDPDYQPPTQEPEESASSGS
> ----------------------------MIPERSRSRRRHAVLVVRAPGH**RQLR**PLERESWTPQGQRVLTLPAEQAPQPEVSNRPDPSTARRLLLLAYRARHFL
> --------------------MESEAKMRELERKIKESKSLVLRDDLRRRR**RVLR**RLEFVDKEGVIQRKGRTACEVSTTDELLVTEMIFNGQFNDLSVNDTVALL
> AKSAMSLNEIYRVEISSTYETDGVTMYVVSVFLRTTQKGLPTMETLDQRR**RRLR**REYQGQTPDYHVEHRYSDFRAMVKSISNVVNDHDHFRFCAYCSRVGTTES

> ------------------------------------MAETVVSRLKAELK**RSLR**SHVAQAVNDVCESFTEELKPQEPALSEIRPQVGASAFETDYDCCRRLVEY
> LKRTISRGKRTREISVKASDTVVVPGVVDEARLKIDNALDAAGLRDEVAI**RYLR**RGIKYDKHEGVDEIVFDSLDEVVASHRASSSDKYCKASTYLLLNDVLVTP
> ---------------MATNHPRQAFLVSAESSSDSSDSSRLERRRAQSRT**RQLR**YLERKRQYERDLQASVETLQSEVSHLQSELHGLRNAELQRCDCPISASAA
> ------------------------HDGPGPTTRALLARRAGVAAPGKRLE**RRLR**ARHARAVPESGGARVPRVGAAREHGAGGGAGRGRRGAGRQVRAAGVGGVQ
> ---------------MADKLAGWTLLISDLEAKYMELEKLGKETLQEFAT**RFLR**VSSSPSSSPPLLFFFSNKIFERFGTPNCIIFSVVAYGVRMIVYTYAHNPW
> -------------------------------------------------M**RVLR**PTRLALQRLRAHSARQQRVQLIARAFSDAADPAKRALDPSAAAPSSAAVP
> ------------------------------------------------MR**RSLR**KSRPGAETLQGMSAGQTEAEKPDVETLNVLTRTCAGFRYREMTLANPPTQ
> ESVDQLKRTGEDIRTQGNNASDSAVDLAKRSMDQAGSALDDLGKSAQDYD**RRLR**DSINSSVDDAKDNGSQTLSSWQDSISSLVNSTREATFHGFEALQNQITAT
> ---------------------------------------MAGAREKRRTA**RILR**GETTTWKAIWRELKASGSAAGTEGKDYFLGENAVLAHYARVHSKEGAQAL
> ---------MARTTGTPGSSKRPPGPEDAHVTRAQRDSAESERSRSRQRQ**RELR**HETDRTLRDLPPLPVYNRGVLEAQRAANVARARRDEQEIRLAMDGRYKRT
> IARLELENFKSYGGAHVVGPFQRFTALVGPNGSGKSNLIDAISFVLGVHS**RHLR**STQLKHLIHKAPSDDDTTSARAAAVTLVYELADGEAPPSASQKKVRFTRL
> ---------------------------MAAAALSGSSFVPLCTSSEGSDG**RGLR**LHNAGVDVLNGAKLAARKIALVGVLNVAEDEKTEPERGLFLSSSAASVAE
> ------------------------------------PPLQHHGRYRPHHV**RRLR**RHQQASVAMEHPQGGQAHQGRHRSR-HGLLQEVPERGVRELCRGVPPLPE
> ----------------------------------------------MALD**RELR**RLLEYARLPETDNASSKDGNPPRPTARGILDRIIGIHRQTCVPSMGVSAD
> PMLAEWIYATFKARVSRATLARLRNLPSSYFSHVNLSATKRRRVKFPQFE**RELR**KIFFQNEAKTAMADAVLLLKAHELAERLGIDATQLRLSNGWLQSFKKRNG
> ------------------------------------------MGVVKRRR**RGLR**CDAGDYGGCPGPGRNVSDGGDKHAMTAGISGSGRNGYGSAVSTVNSMVSE
> CFRCGLAGELICCDGRLRCLLKVSKTTDAGIVAANGTRSSNRAKLTDYAS**RHLR**THAAPQVPSLKKIFHSTVPHAKRRYKKRHKATDRSYRELEHMPLPVWNES
> RAKTNPWRHLHLHLHLHLHQRRAPTLRFISPFSRLTRRHALRRSRHRRPP**RRLR**GPQVRAQQHQQPQHQQRRLLHGRRGRRGRPARAAGLGPGRARLPPRAAGQ
> -----------------------------------------------MWR**RELR**WMRHVVRAQDQDQRLDRWLRRQFPSLPQSFLQTQLRKRKIRLQPPPGVTD
> QRRVPAAGRRAQVLVLSSPSHAELGGHRHRQRVQGADAGAPVHDAAVALA**RGLR**AGQAEAGQDGGGRDHAAGVHRRGPAARGAAQDAHAHGQGAAVQHQGLRPE
> SERARWVSKTYAGWKEQMSTMEQKVQALEAGRERERESCGNIQRFHAGQV**RDLR**ATLAQVQARRATAAPHLATNVRSIARGKRGHGAAETKVTQDIKLDSDDSS
> --------------------MNSTERGNLSMEQVHAAWLAAGARGDVDTM**RRLR**EQHPQWLGLQRRVGTAADAAASDPARPQRQQFCGWNGFHLSSIGASTLHT
> --------------MPSTPAQTLEDGLDVNMVAFHFDLAVASALPLPDFV**RLLR**GQTEADPRPNKDLYELPLPRDPALHEAARRWNEVVRHGVVPEWLPSRPAR
> -------------------MSWEGSTSSECDMSYTSTTEATSTTSSKNIA**RPLR**VYLDLRESESTDEFASTTESTSTNESTSTTEITPTTIHMSPSSSVYTTSE
> -MGLSRRAKAAESGAAASSSSSSSSSASRLLAFFSHRKQDAQVPEQQDKR**RHLR**IKTKHKRPKQRSRSRSRSFSGAEDNNDDGDDAPGSPHANVRLRQSPGGRA
> --------------------------------MELSSLSMLFGVPPSTLA**RSLR**RAEEALSKTLNNYAPAHIAVPSPSRQNELPKLVEAQEPLLKHTFDFIDGK
> MLKLVQKYGTRHWTIIGTKLPGRNGKQCRERWHNQLDPAIRKEPWSPEEE**RILR**ELHDKFGNKWAEIAKMLPGRTDNAIKNHWNSSKRRLKRGVTPTAAAQRKR
> HDRRGVYGDHRDHHDGVRQARARQELRGRGAAHKAEHGQEVRLVVALLHR**RGLR**LRHQLPAATHALPLRGRYWRARLQV-------------------------
> ----------MIYPMPLIDDLLEDFDSLLWFCSLDMASGFWVPIEPERSP**RSLR**RSACSSGCECRLV-------------------------------------
> GAKRFSIEGGESLIPGLCELLESASDHGVEVVQMGMAHRGRLNVLANVLQ**RPLR**SIISQFQPYLPDEPDYPNNSDDVRYHLGTSSGIEMRNGKRLEVNLAANPS
> -------------------------------------------------M**RLLR**THCMGDCAVHFQVVSDQSLNFPNFGVVNFEDPNYNVELPVFSIHGNHDDP
> RGVRRRHEDVPALERQVRRPGRERLADRRLHGGAGRHAQERHHRQGPNGG**RALR**AERLHHPERLVGRRLRGRAEHQGRLGQQRLQGGRRRRALGRRQGGAAQRA
> ----------------------------------------------MLEI**RELR**PLLAILNEDDKPLEQVASVFLRTFTKNDHFKVGCTICILLQDNLLTSSQR
> KRIEILGTDGNLYPFLCKREKTGDLRKDARMMEFNSMINKLLQKDQEGRK**RKLR**LRTYAVVVLNEESGLMEWVRNTKAMRQLIGQIHKTERGYIQPIRMTHEVK
> RHVPDGHKHLRGPRGVRRGARDAAPHQPGAAEGRRQGERGAQHGRGRAQQ**RPLR**AGPRGRYGRHPVLHARGRLALGAHLGGARDPARRGAQGLHRHRRHEPHRV
> -MVLAELGSKLQGALAKLNRTTVVDDEVFSALLKEICGALLESDVQVKLV**RQLR**DNVKLAVSLEDASSGVNRRRLIQKSVVDQLVQMLEPEAQPYKMKKGQSNV
> ------------------------------------MFTKWMCLQRSSTE**RYLR**ISICNNLKGFGT--------------------------------------
> -----------------------------------------MARCSSSAF**RALR**RGFSSSASSAPLKKTPLYDLHVALGGKMVPFAGYAMPVQYKAGVLQSHLH
> ------MSGELRRDKINEWLDGLGGSDEPLEDEDQVNVGIEDDESRRMIV**RLLR**VYRQLTADKSDCPPATVLSTEHHIDTGDAAPIMLKRRRQAQAEDEVIETN
> ------------------------HDGPGPTTRALLARRAGVAAPGKRLE**RRLR**ARHARAVPESGGARVPRVGAAREHGAGGGAGRGRRGAGRQVRAAGVGGVQ
> ----MQIRVAEEDRDKTAFMTKRGLYRFRRTPFGLTNAPATFQRLMNGVL**RGLR**WLTCLVYLDDIIIFTKGGIERHVIERAGVFERLRAAGLSLKLKKCTF---
> RYLLKPTALELFIQSTRKNYFFNLVSEDVALFHEALMTRRPLLLKRDPSV**RRLR**HPSSLFRNSPMSNRWVQHEISTFEYLMWLNTIAGRTYNDLTQYPVFPWVI
> -----------------------------MASLSLWKVVELAVSGDASWG**RVLR**KKAKKEQYLAGFPGLVGNTPMVELTSLSKATGCTILAKAEFLNPGGSSKD
> -----------------------------------MKQPEFFDDGSGRVC**RLLR**SLYGLKKAPRIWYKTLDKYLRQCGFQRM----------------------
> -MDVAAGEKVGICGRTGAGKSSLIVALFRICDFDSGRVLIDDVDISSVNL**RELR**RSLAIIPQDPVLFSGPLRENLDPFHEYADERIWRVLQQVHMAESLRRWGA
> ---------------------------------------MLPAKSLRQAS**RLLR**PLGARSFALGQHIDDSKFSAFSLKLSDEQREFQQLARKFAREEMIPKEKH
> QRPRQRGDNPIVFLDVSIGEKNVGRLLIELRADVVPRTAENFRRLCTGET**RELR**TGRRRHYANPFHRVVKDKFCQSGDYANHDGSGGECTFGHKVPPPPSTFDD
> ----------------------------------MDTHSFCTALGKEEAN**RQLR**IHYANWVTEADIKEMAEAGVNSLRVPVGDWMFQPYEPYVGCTDGAVEELD
> -----------------------------------------MASQEPQIH**RALR**ADVHEISAMIQELADFEKESASNKATPEKLLETVNFADDVAPEHIPGFPL
> DGVGAKMDSMELEREKGITIQSAATYCSWKDSNINIIDTPGHVDFTIEVE**RALR**VLDGGVLVLCGVSGVQSQSLTVDKQMKRYGVPRIAFINKLDRMGANPWKV
> LRADRRPRGREQGRGQALDAPGRRARHVQGAHVHRHQARRRAARPHQRGD**RPLR**EEGLQARGAQAHDGDRGARQEPLRRPVGAPLLPRSRQVLHLGPHRLHGLG
> -------------------MPLVNSIFWSPVLRTGALAPSIKRIRQAVTK**RWLR**GSISNFEYLIHLNTLAGRSFNDITQYPVFPWVIADYSSEFLDLNARDTFR
> ISLIVNEDAAHDCVQKLGDLGVLEFTDLNPELTPFQRRYVNYVKRCDEME**RKLR**YFEVELAKFSIAPKPAGSDTAARALDTLERLLEDKEQELLQLNSMHEKLT
> --------------------------------------MLVEGEPGTLTG**RPLR**LYADGIFDLFHFGHAKALQQCKEAYPNTYLLVGCCNDEITHKLKGRTVMT
> ------------------------------------------------PG**RLLR**QVRQDHAQEDQAHRRLPSLRAGHGRAPIYLLPAGGQLPLQLVPGRLRVHR
> ----------------MYLMLGTRPDLAYPVRECSQFLENPGLLHWRAAK**RGLR**YLKDTVDWGIQLGGLALLVQEVVHLRQKLKELQVQQIQPSQVFVDNESAK
> ------LEIGVQGEHLLLRRDTHTGLGVLAHTLLEEVGLALQRDHVHPVE**RVLR**VVDLRHAQRVQQAVGHELNVLAHQRRVHADQLHRQRVAHELVLDGHGIAH
> EMLDRVGDGAFGEVSRARSLKTHEIVAVKKIKALFPTWEECLQLRELKSL**RVLR**HENIVLLKEVIRDKEELYFVFEFMQTSLFRVMRNFKCQGGSSSPVRSIMY
> ----------------------------------MDTYTFCTALGAEEAN**RQLR**IHWATWVTEADIKELAEAGVNSLRVPVGDWMFNPYEPYIGCTDGAVKELD
> AEVLAGVASLGAKLLLDTQKLVVLGQTLRTARRASLDLASAQTHSQVSDE**RVLR**LTAAVARHDGPASLLRHRHGLNRLRDAADLVHLQQKSVASLLLDGLLHTR
> SSVETNDGGLVVNREVIHVFAARSPSPVGQRRRHVCVRVDGRVRDDGQGP**RPLR**GRLLEGGELGAAQELHAYVHDGREPQGVQVINDQ----------------
> ------------------------------------------MRAALKLS**RRLR**VPAAHITRAQSTAAAPASLAASWDRPFPSILVSADGVSAQGSFAEAQANY
> ---------MEAILEETAAIYSTEYDVSLTASFVEIYQERLRDLLLPHSS**RPLR**LREDKDHNVHVEGASEISISTVAGGMAVLSRGTAQRSTGSTLMNADSSRS
> --------------------------------MLATKTLQTIGLTRAIAS**RGLR**FASSASTTETPYYDVVVAGGGVMGFSTAYHLAIAAPKLKIAVVEKDPCYK
> -----------DPQLDPGPRGQHAARAAQHDRAQGRPRVRAAGQVRVLQR**RRLR**QGPHRQAHDRGRHGLGPHQAGRHAHRAHVGQHGHRPGAGRRALRLPHHHH
> --------------------------------------ADQGPCQAQRGR**RALR**LECDPDGAADTRAEPHAAAPGRLQPGGPHGHARGRRGRRHAGYVPDDGDD
> -ERRTKLWVSCCWPLASSSSSTTAFGCCLRRSLTRTTSSSSSSCRTTTPS**RSLR**CCSCCSLLEPPPSSEWS---------------------------------
> ----------------------------------MDMYTFCTALGKEEAN**RQLR**IHYEAWVTESDLKEMAEAGVNSLRVPVGDWMFNPYEPYIGCTDGAVEALD
> PADVISINMTSALSIYSLIFMRFAWMVKPRNYLLLACHASNETAQLYQLQ**RGLR**YQASKKSAEKQA--------------------------------------
> EHKVMVTSGPQSDRLAFGEYIQKNMKLYELRNGVTLNGPATANYVRGELA**RFLR**RAPYQVNVLIGAVDATPEQKAEPSLHWIDYLGSMVKVNFGAHGYGAHFCL
> --------------------------------------AARAHAAPERVR**RVLR**LSRAARRREPGPGLPVLRDAAVCQAGGHRRHHGRQQPVHAPRRPRELRGD
> -------------------------------MPGWSALPPHVFSIAEGAY**RSLR**RRLHEPGESKKNQTILVSGESGAGKTETTKFIMNYLAESSRSAAIAGSSS
> ------------------------------------------MAAASEAP**RKLR**RATYVKVQDLTPGSQGHNLVLQVVSIAPTVEKKRYDGTISRIAEAVMADE
> STFKIPLKLHEHSICSISKQSHLRKFIERASLVIWDEAPMTHRHAFEAVD**RTLR**DVMNNDREPFGGKVFVISGDFRQILPVVKRGTPAETIDACLKMSPLWRHF
> -----------------------------------MEMPDYFNVGSGRVC**RLLR**SLYGLNQAPLIWYRMLDEHLRSCDFKRSKMDNGVCWRVVGGRPIFLPVYV
> VSFPSDCSPFTVRNVVGSSDVGFRIRLEGLARDHLRFSTYEPEMFPGLIY**RMLR**PKCTLLIFISGKIVITGCEVQQK---------------------------
> --------------------------------------MLDGVDIKQLNV**RWLR**RQIGLVGQEPTLFIGTIAENIAYGQDTMPDMEEIEAVAKMSNAHEFIMQF
> DLHQNHGAPKFTVFDDLDPVVTTYDCFDSMLVPKDHVSRKVTDTYYVDEN**RVLR**AHTSAHEVATMKKGFTAFLVSGDVYRRDEIDASHYPVFHQMEGVRIFSEL
> ----------------------------------------LLRGPGPPGR**RHLR**VHARGQDGVPAEAGGRGRAQHRDGGALHGRLLPQAPHPRRRRLRDPAEPP
> -----------------------------------------------MSF**RELR**NLTEMMRALGYPRPISMENFRTPNFELVSDLLYWMVKKYDPSSSIAEEID
> LQPAELLARLRGAVQDRVRADHRAHAAGHNALDEALGLASRLLQSRQHQH**RHLR**AVLSNKAGRVARLRQHHQHLRVDVDGRLHGRRRHGLRRLHRAVHLQHGVA
> ASAARSRLSVSAWLAWSPCEKLKRATFMPASMSSPSPSSDQQDGPSVQKM**RVLR**STPSTLRMMLSSSTPC----------------------------------
> VEKTKSSKDDLVTVVDKQCQDLVFATIKQSFPTHEFLGEENVAPGSEASA**RALR**ELASKEWLWIVDPIDGTTNFVHHRPASVVSVACAHLGDVVVGVIYDPYRD
> ----------------------------------------MLALRRRELQ**RALR**APAVRARCLATVSREVKEDAALTPATVGPYMSERLEEFKPERIRNFSIVA
> ------------------MTPLFRSVTRARAAHAPRLLSTAAEHATPPTR**RRLR**ELPPPLNLTDAAAARIKELLASKPDAIGIRLGVRTRGCNGLSYTMNYADK
> ----------------------------------------------MFAS**RVLR**MAVTKTSTGLVGLKVNPNARQDLIKIYRRTLEEVKILPPEAKNYRNAVEQ
> PHHRAQGQEVQVPPGTLGHPEPERHLHHRQRCRGAPSQLPRGAGGAQEDG**RGLR**RPHSHLGPRAHGARPAPGSGRHQRAAPRPQQDRHDQEGHRSGLLVQDAAQ
> RQQDVEAGTETSTPAADRLPLGIRVEAQRERASHSAQGPYRGQGVFATTR**RGLR**RDVSAGGLPELDPCQVGEVRRGWIRD------------------------
> -------------------MAAPTKELKEWLDEWPVVRDLVDELLLSLKR**RQLR**GSYETAKMTTSVLSKVLETVEWSTAGEILEKIRQLGHMLTKAHAHELAIG
> ------------------------PARADAAAAAHHEDGRGRAHDQRRGA**RALR**QGLRDVHPRAESARLDPHGGEQAPHAAAQRHRHGHHQDRRLRLPHRHRPA
> ASAARSRLSVSAWLAWSPCEKLKRATFMPASMSSPSPSSDQQDGPSVQKM**RVLR**STPSTLRMMLSSSTPC----------------------------------
> LQPAELLARLRGAVQDRVRADHRAHAAGHNALDEALGLASRLLQSRQHQH**RHLR**AVLSNKAGRVARLRQHHQHLRVDVDGRLHGRRRHGLRRLHRAVHLQHGVA
> ----------------MYMATGTRPDLAFPVGYLSRFVENPTVQHGGALK**RVLR**YLVATADHARSTSRLDAIKIEEYCDSDWGNCPETRNSVTGYTMLVAGEPV
> ----------MHLNTLAGRSYNDLTQYPVFPWVLADYDSEILDLSDSGVY**RDLR**KPMGAIRREEEFRTRYEGLMESVGTVDGDESADNPLGSRPFHYGTHYSSA
> TLQSHFDGGAEPTPRFDVLQEYPLFVTWEIEEQGGTHLRGCIGTLAPTRL**RNLR**DFTFKSALRDRRFDPIGPQELHRLHCSVSLLIDYQDAESFDDWEIGTHGI
> -------------------QYGRRSDPCSRRGRHQRQRVHDQGPGDQVHL**RRLR**HRQLPVGARPHPLPRVRLPHHVQGAHQAPHPVRGPVSGRPRPSERPRPST
> ----------------------------AAAVLDQARAALAGGRHPELRR**RWLR**GRRGRGDGAAAGQVRGRAGRRAVLHRGLPRARLQGRVAAAGGRRGEAQQR
> ------------------------------------------------RP**RALR**AEAARRAGGLRGRQEDGQDRDKDDGAQRQRRQALDGRGAHRRAPQHLPAR
> RRKHPALRWEVMDMTAMASLVDASFDLVMDKGALDALMAEDTPEIKQDAT**RMLR**EVRRVLAPGGRYCCVTMAQDFILQHLLGFFSAKDAAEMEPTSWGVGVQEL
> IARLELENFKSYGGAHVVGPFQRFTALVGPNGSGKSNLIDAISFVLGVHS**RHLR**STQLKH--------------------------------------------
> PVTNPPPRSRKPVDKIVTIPTTLARRLQPRPRLQRSRNTPPPRPRLPRTT**RVLR**RRDRGLPGHRVPRRQLGETTRLLRPRPTKTTRVARRR-------------
> ---------------------------------------MGLSGSPGTFN**RLLR**RVFSDLRDVIRIYFDDIYVFTQDTDVRTHVEALDRVLQRCQEQQLYVKLS
> -----------------------MEDEVYPMTLLTEELKSDEVETRIKAM**RRLR**TVAQALGPERTRSDLLPFLREATEDEDEVLVALAEELGGFVDLVGGTEHA
> RQQDVEAGTETSTPAADRLPLGIRVEAQRERASHSAQGPYRGQGVFATTR**RGLR**RDVSAGGLPELDPCQVGEVRRGWIRD------------------------
> --------------MLMYLATGTRPDLAFAVGQLSRFVAKPSARHVGTLK**RVLR**YLAGTVDYGISYERPTEVPSTIVLEGFCDSDWANDSESRKSTTGFVFSLA
> -------VAHGEHGISVARVRPGQVPRQPAHRAPGRPFPLPSSQARTSAV**RHLR**AARGEQGAAAHQRVRRERDFARAHFQHDGR--------------------
> --------------------------------------------------**RNLR**SKTRWFTGFCNSHQVSHFATFFIDARAEISVAES----------------
> KWIRNLGKRIGSEGWARGSQSRTRRLSVDCSSCSRGESGSSRAGRGTGWE**RPLR**GPSPGVEQSTQNWYGQGESDCLIKTKHCDGPCGCSRNVISAQCSECQSEE
> -------------------MCKCRSHGTFPLFGLQSSHLNICYYHQDLHR**RPLR**PGSRPGFCSDRRALLLIGAWRLPRRPGIGRALQRHPFSGLVDSAGELLHT
> EESDTSFDYRKAVGMLMYLATGTRPDLAFAVGQLSRFVAKPSARHVGTLK**RVLR**YLAGTVDYGISYERPTEVPSTIVLEGFCDSDWANDSESRKSTTGFVFSLA
> YCIRLRGRVPPQPPRPVGHQGAGGPLHGLVRRDGGRGQAQTRGAQEPGGR**RRLR**DGRQHQEARYRAGRGGAGQARQEAEEQGDGQFLQVPDAREEARPYVRYAA
> AAVKYKPQLHVSFGCHPYPAVQANGSECGSERERVFGRRVRGLQLGLASV**RALR**LVQRQVGHHVRVVSAQRTIQQVPAPPLLGGCCRLDR--------------
> ------------------------------MPQGLSNAPATFNRLVTQLF**RPLR**AFTQTYFDDIFVHSRAEAGQTAMEVHLQHLRHVFEVMRANTLYA------
> CCLMLRTYHTCSVCRLQDEVAPALHRTFQGCGQEGPCVHAQSSEEDADAP**RLLR**GLAQAVP-------------------------------------------

> KQLMRSKPYRAVVGSLMYLMLGTRPDLAYLVRECSQFLENPGLLHWRAAK**RGLR**YLKDTADWWIQLGGLAWSDQKLDEHLRAYADADFANRVDDRKSVAGYVTQ
> AEVLAGVASLGAKLLLDTQKLVVLGQTLRTARRASLDLASAQTHSQVSDE**RVLR**LTAAVARHDGPASLLRHRHGLNRLRDAADLVHLQQKSVASLLLDGLLHTR
> AEVLAGVASLGAKLLLDTQKLVVLGQTLRTARRASLDLASAQTHSQVSDE**RVLR**LTAAVARHDGPASLLRHRHGLNRLRDAADLVHLQQKSVASLLLDGLLHTR
