# Supplementary material for: The Malarial Host-Targeting Signal Is Conserved in the Irish Potato Famine Pathogen
Source: PLoS Pathog. 2006 May 26;2(5):e50. doi: 10.1371/journal.ppat.0020050 (PMC1464399; doi:10.1371/journal.ppat.0020050)
Supplement: Table S1 — (19 KB PDF) [file ppat.0020050.st001.pdf]

Supporting Table 1: List of protein IDs in the predicted *Phytophthora sp.* RxLR HT-secretomes

P.ramorum **(source: JGI)**	***P.sojae*** **(source: JGI)**	***P.infestans*** **(source: Randall *et al*.); subset with at least 100aa after the SS cleavage site.**
44614	108861	Contig2560_1
51422	109021	Contig257_1
73159	109104	Contig3711_1
73482	109418	Contig3739_1
73572	109434	Contig6919_1
73625	119323	E7.1230.C1
73649	127092	E7.2698.C1
73707	127119	E7.339.C1
73724	127824	E7.3882.C1
74039	127875	E7.4221.C1
74075	127883	E7.4380.C1
74178	128358	E7.4993.C1
74299	128372	E7.5531.C1
74336	128394	E7.6222.C1
74348	128439	E7.623.C1
74367	128911	E7.7045.C1
74378	129113	E7.7226.C1
74393	129513	E7.8117.C1
74395	129531	E7.829.C1
74469	129601	E7.8963.C1
74521	130409	E7.9126.C1
74580	130529	E7.9208.C1
75380	130530	E7.9348.C1
75732	130725	PEX-R1 E7.6301.C1
75759	130789	PEX-R12 E7.4706.C1
75817	130892	PEX-R14 E7.5930.C1
75845	131011	PEX-R15 E7.6019.C1
76149	131013	PEX-R16 E7.6418.C1
76309	131119	PEX-R17 E7.7223.C1
76324	131125	PEX-R18 E7.8373.C1
76339	131847	PEX-R2 E7.4015.C1
76429	131852	PEX-R20 rpvb_3739.y1.abd
76892	132044	PEX-R24 rpvb_12884.y1.abd
76894	132150	PEX-R25 rpch_15494.y1.abd
77464	132253	PEX-R27 PV004C8.XT7
77763	132282	PEX-R28 E7.6199.C1
77765	132305	PEX-R29 Contig1828_1 site=1011:1397
77786	132405	PEX-R3 E7.9705.C1
77933	132650	PEX-R32 Contig5688_1 site=358:736,780:788,900:974,997:1094
77945	133003	PEX-R33 gi|56121797|gb|CV970784.1
77948	133017	PEX-R42 R-dEER42 contig 4167
78050	133354	PEX-R43 rpcm_4494.y1.abd E7.cl.7454.singlet
78054	133362	PEX-R44 rpvb_13318.y1.abd
78157	133390	PEX-R49 E7.6112.C1
78163	133675	PEX-R5 E7.8848.C1

78246	133752	PEX-R50 PG001C8.XT7
78267	133799	Pex-R52 E7.6097.c1
78294	133810	PEX-R6a IpiO1 Genbank L23939
78400	133811	PEX-R6b IpiO2 Genbank L23938
78539	133874	PEX-R7 MY-15-C-09
78544	133875	PEX-R9 E7.589.C2
78646	133876	RD7b pex147-2 156c
78748	133894	RD7c pex147-3 169c
78800	133912	rpcd_1706.y1.abd
78978	133925	rpcm_2725.y1.abd
78979	133930	rpcm_2772.y1.abd
78980	134000	rpct_1002.y1.abd
79106	134001	rpcy_0655.y1.abd
79107	134020	rpvb_5774.y1.abd
79108	134166	
79110	134204	MY-14-C-10 (sequence is shorter then 100 after SS)
79119	134359	
79329	134429	
79397	134706	
79705	134711	
79763	134909	
79820	135133	
80241	135137	
80471	135162	
80523	135175	
80526	135182	
80530	135206	
80531	135209	
80533	135341	
80550	135445	
80775	135585	
80828	135626	
81610	135627	
81822	135689	
81823	135837	
81825	135840	
81907	135843	
81908	135847	
81911	136045	
82188	136046	
82227	136207	
82244	136214	
82374	136215	
82380	136243	
82381	136266	
82382	136280	
82658	136286	
82736	136868	

82793	136869	
82794	136877	
82803	137090	
82880	137322	
83060	137361	
83086	137398	
83274	137399	
83372	137455	
83373	137461	
83417	137514	
83582	137608	
83583	138312	
83593	138565	
83619	138592	
83943	138594	
84010	138595	
84117	138921	
84198	138954	
84626	139045	
84627	139178	
84708	139179	
84992	139182	
85042	139188	
85073	139205	
85154	139206	
85155	139209	
85377	139216	
85379	139217	
85382	139250	
85589	139437	
85688	139438	
85830	139461	
85835	139921	
85876	139923	
86023	139988	
86033	139995	
86034	140000	
86166	140196	
86187	140204	
86191	140210	
86248	140524	
86463	140568	
86600	140616	
86841	140648	
86871	140661	
86872	140683	
86936	140716	
87084	140717	

87086	140719	
87087	140885	
87139	140904	
87141	140939	
87596	141324	
87903	141401	
	141697	
	141756	
	142101	
	142112	
	142136	
	142308	
	142463	
	142529	
	142795	
	142859	
	142972	
	143116	
	143170	
	143225	
	143254	
	143795	
	144029	
	144030	
	144031	
	144038	
	144167	
	144427	
	144434	
	144507	
	144578	
	144844	
	145361	
	145414	
	145598	
