# Supplementary material for: The Malarial Host-Targeting Signal Is Conserved in the Irish Potato Famine Pathogen
Source: PLoS Pathog. 2006 May 26;2(5):e50. doi: 10.1371/journal.ppat.0020050 (PMC1464399; doi:10.1371/journal.ppat.0020050)
Supplement: Table S2 — (28 KB PDF) [file ppat.0020050.st002.pdf]

Supporting Table 2:
Detailed annotation of the *Phytophthora sp* HT-secretome.
Proteins were annotated using the Pfam HMM library of global alignment models with an E-value cutoff of $1e^{-10}$, and the NCBI BLASTP tool to search the non-redundant protein sequence database with an E-value cutoff of $1e^{-25}$.

	In silico annotation of *Phytophthora* proteins containing a SS and RxLR	No. of proteins	ID	Pfam description	Pfam entry accession number	Pfam hit E-value
	P. infestans	**P.infestans**				
1	Kinase	2	E7.623.C1	kinase	PF00069	3.30E-47
2	Kinase		E7.7226.C1	-	-	-
3	Acyltransferase	1	E7.2698.C1	Acyltransferase	PF01553	1.1 e-13
4	peptidase family M3	1	PEX-R17 E7.7223.C1	peptidase family M3	PF01432	6.00E-172
5	transglutaminase elicitor	1	E7.8963.C1	-	-	-
6	ATP binding cassette	1	E7.9348.C1	-	-	-
7	phosphoribosylpyrophosphate synthetase	1	E7.4993.C1	-	-	-
8	sugar transporter	1	rpvb_5774	-	-	-
9	phosphatidylinositol glycan biosynthesis protein	1	Contig3739_1	-	-	-
10	grpE	1	E7.6222.C1	grpE	PF01025	7.30E-16
11	small cystene rich protein SCR58	1	MY-14-C-10			
12	*in planta* induced genes (ipi0), ipi01	1	PEX-R6a	-	-	-
13	*in planta* induced genes (ipi0), ipi02	1	PEX-R6b	-	-	-
14	avirulence protein 3a (AVR3a), alleles	3	PEX-R7	-	-	-
15	avirulence protein 3a (AVR3a), alleles		PEX-RD7 b	-	-	-
16	avirulence protein 3a (AVR3a), alleles		PEX-RD7 c	-	-	-
17	Unknowns	44				
	P. sojae	**P.sojae**				
1	Acyltransferase	1	134204	acyltransferase		6.30E-13
2	Proteases	2	134166	papain family cysteine protease	PF00112	1.90E-23
3			138921	-	-	-
4	transglutaminase elicitor	1	132405	-	-	-
5	tetratricopeptide repeat	1	135341	TPR-2 Tetratricopeptide repeat	PF07719	2.60E-14
6	ubiquitin carboxyl-terminal hydrolase	1	128911	UCH Ubiquitin carboxyl-terminal hydrolase	PF00443	4.70E-38
7	ABC-transporter	1	139988	ABC-2 type transporter	PF01061	5.00E-20
8	extracellular dioxygenase	2	133354	-	-	-
9			133390	-	-	-
10	DNA repair	1	140939	REX1 DNA Repair	PF06331	1.60E-20
11	homology to *P.sojae* necrosis inducing factor	1	138312	-	-	-
12	very glycine rich protein	1	142112	-	-	-
13	elicitor avirulence protein 1b (AVR1b); secreted protein that triggers HR response in plants	1	108861	-	-	-
14	elicitor AVH 1b	1	109104	-	-	
15	Unknowns	162				

	Top annotated Blast hit NCBI gi number	Top annotated BLASTP hit description	Blast hit E-value	Annotation Source
	**P.infestans**			
1	66824999 66812872	rasGEF domain-containing protein [Dictyostelium discoideum] and putative LISK family protein kinase [Dictyostelium discoideum]	2E-43 and 5E-42	this paper
2	67603708	mitogen-activated protein kinase 2[Cryptosporidium hominin]	6.00E-32	this paper
3	-	-		this paper
4	47523776	endopeptidase 24.16 type M1 [Sus scrofa]	1.00E-179	this paper
5	37930526	elicitor-like transglutaminase M81B[Phytophthora infestans]	2.00E-62	this paper
6	60302844	ATP-binding cassette, sub-family D(ALD), member 3 [Gallus gallus]	3.00E-27	this paper
7	23619201	phosphoribosylpyrophosphate synthetase [Plasmodium falciparum 3D7]	7.00E-36	this paper
8	168402264	similar to glucose transporter 1A [Danio rerio]	4.00E-24	this paper
9	62531278	phosphatidylinositol glycan, class O[Mus musculus]	3.00E-76	this paper
10	-	-		this paper
11		small cystene rich protein SCR58		Torto T.A. and Kamoun S.
12	-	ipi01 gene (AAA21422.1)		
13	-	ipi02		Pieterse C.M. *et al.*
14	-	AVR3a		Armstrong M.R. *et al.*
15	-	AVR3a		Armstrong M.R. *et al.*
16	-	AVR3a		Armstrong M.R. *et al.*
17				
	**P.sojae**			
1	-	-		this paper
2	-	-		this paper
3	68209970	peptidase S8 and S53, subtilisin,kexin, sedolisin:PKD:Fibronectin, type III[Methanococcoides burtonii DSM 6242]	3.00E-34	
4	37930526	elicitor-like transglutaminase M81B[Phytophthora infestans]	1.00E-141	this paper
5	-	-		this paper
6	-	-		this paper
7	50881997	pleiotropic drug resistance transporter [Phytophthora sojae]	2.00E-29	this paper
8	6684539	extracellular dioxygenase, putative [Aspergillus fumigatus Af293]	1.00E-61	this paper
9	66845396	extracellular dioxygenase, putative [Aspergillus fumigatus Af293]	7.00E-51	
10	-	-		this paper
11	2132710	necrosis-inducing-like protein [Phytophthora sojae]	4.00E-26	this paper
12	13561980	flagelliform silk protein [Argiope trifasciata]	4.00E-30	this paper
13	-	elicitor AVR1b		Shan W. *et al.*
14	-	elicitor AVH1b		Shan W. *et al.*
15				

	In silico annotation of *Phytophthora* proteins containing a SS and RxLR	No. of proteins	ID	Pfam description	Pfam entry accession number	Pfam hit E-value
	P. ramorum	**P.ramorum**				
1	Acyltransferase	2	76149	acyltransferase	PF01553	9.50E-15
2			76309	acyltransferase	PF01553	8.50E-20
3	transglutaminase elicitor	1	84198	-	-	-
4	tetratricopeptide repeat	1	75817	tetratricopeptide repeat	PF07719	3.40E-13
5	SCP-like extracellular proteins	2	78267	SCP-like extracellular protein	PF00188	1.90E-26
6			80241	SCP-like extracellular protein	PF00188	1.90E-25
7	MatE efflux protein	1	86034	MatE	PF01554	9.00E-73
8	NUDIX domain	3	86166	NUDIX domain	PF00293	2.50E-11
9			73707	NUDIX domain	PF00293	2.10E-11
10			73724	NUDIX domain	PF00293	1.30E-11
11	ubiquitin carboxyl-terminal hydrolase	1	76429	UCH Ubiquitin carboxyl-terminal hydrolase	PF00443	1.20E-18
12	phosphoribosylpyrophosphate synthetase	1	73159	-	-	-
13	Helicase	1	44614	DUF889 eukaryotic protein of unknown function	PF05970	1.40E-15
14	cytochrome P450	1	75732	cytochrome P450	PF00067	6.30E-39
15	very glycine rich protein	1	76894	-	-	-
16	similarity to dynein, axonemal, heavy polypeptide 5	1	81823	-	-	-
17	Unknowns	131				

	Top annotated Blast hit NCBI gi number	Top annotated BLASTP hit description	Blast hit E-value	Annotation Source
	**P.ramorum**			
1	-	-		this paper
2	62650362	similar to lysocardiolipin acyltransferase isoform 1[Rattus norvegicus]	3.00E-41	
3	37930526	elicitor-like transglutaminase M81B[Phytophthora infestans]	1.00E-121	this paper
4	-	-		this paper
5	-	-		this paper
6	-	-		
7	15219524	MATE efflux family protein [Arabidopsis thaliana]	3.00E-60	this paper
8	-	-		this paper
9	-	-		
10	-	-		
11	-	-	-	this paper
12	1289360	phosphoribosylpyrophosphate synthetase [Plasmodium falciparum]	1.00E-60	this paper
13	4895169	putative helicase [Arabidopsis thaliana]	2.00E-48	this paper
14	15222515	Cytochrome P450, putative [Arabidopsis thaliana]	4.00E-54	this paper
15	34898126	putative glycine-rich cell wall protein precursor [Oryza sativa (japonica cultivar-group)]	6.00E-33	this paper
16	68361834	similar to dynein axonemal, heavy polypeptide 5, partial [Danio rerio]	7.00E-45	this paper
17				
